# Cloning of the Human *MORG1* Promoter: Differential Regulation by Hypoxia and Prolyl-Hydroxylase Inhibitors

**DOI:** 10.3390/genes13030427

**Published:** 2022-02-25

**Authors:** Tzvetanka Bondeva, Gunter Wolf

**Affiliations:** Department of Internal Medicine III, Jena University Hospital, D-07740 Jena, Germany; tzvetanka.bondeva@med.uni-jena.de

**Keywords:** MORG1, hypoxia, HIFs, PHD inhibitors

## Abstract

MAPK-organizer 1 (MORG1) is a molecular scaffold for prolyl-hydroxylase-3 containing a domain (PHD3) protein linking MORG1 to mechanisms of adaptation in hypoxic conditions. In this paper, we report the cloning of the promoter region of the murine and human *MORG1* gene. Among other transcriptional factors binding sites, we identified that both (mouse and human) promoter regions contained several putative hypoxia-inducible factor binding motifs. Analyses of the human *MORG1* promoter by reporter assays revealed that hypoxia and pharmacological inhibitors of prolyl-hydroxylases under in vitro conditions in HEK 293 cells differentially regulate the *MORG1* promoter reporter activity. The exposure of the cells to 10% hypoxia showed inhibition of MORG1 promotor activity at 6 and 12 h, but stimulation after 24 h while treated with prolyl-hydroxylase inhibitors led to a time-independent MORG1 promoter activation. Mutational analyses of the individual HIF binding sites on human *MORG1* promoter suggest that the binding sites work in a complex corporation because single mutations were not sufficient to abolish completely the *MORG1* reporter activation by PHD inhibitors. Our data provide the first evidence that not only MORG1 regulate HIF stabilization through a PHD complex, but also that, vice versa, HIFs control MORG1 expression directly or indirectly by a complex regulatory mechanism.

## 1. Introduction

The MAPK organizer-1 (MORG1) protein belongs to the family of the WD40 repeat domain-containing proteins [1]. The WD40 domain-containing proteins are devoid of enzymatic activities, but due to their multiple WD-repeat structure, play an important role in assembling functional protein complexes [2] and are involved in different cellular processes, such as transcriptional regulation [3], ubiquitin-dependent protein degradation [4] and chromatin modification [5]. MORG1 was first discovered as a binding partner of the MP1-protein, that is, a part of the extracellular signal regulated kinases (ERKs) module, and facilitates ERK activation [1]. Moreover, it was shown that MP1 is not the solely protein that interacts with MORG1 from the ERK-signaling path. In addition, it was shown that MEK-1, RAF-1, and ERK1,2 are binding partners of MORG1 [1]. Simultaneously, MORG1 was identified by us in a yeast two hybrid system as a protein that associates with the HIF-prolyl hydroxylase domain containing protein 3 (PHD3) [6]. PHDs are pivotal cellular regulators of the HIF transcription factors [7,8]. Those findings suggest an important function of MORG1 as a scaffold protein for two signal-transduction pathways regulating proliferation via a MAPK-signaling module and hypoxia/normoxia regulation and adaptation to hypoxia via its complex with PHD3.

Hypoxia often occurs because of a microcirculation injury and hypoperfusion in tissues and organs, including the kidney [9]. Hypoxia contributes to renal injury in acute and chronic kidney diseases [10] and as well as in CKD-related pathological processes as anaemia or inflammation [11]. There is increasing evidence that HIF activation may protect against renal damage [12,13,14]. Hypoxia Inducible Factors (HIFs) are the major transcription factors that are activated under hypoxic conditions and have an evident role in kidney injury and repair by inducing the expression of numerous HIF target genes involved in the cellular adaptation to hypoxia [15]. HIFs are heterodimers, which are degraded under normoxic conditions due to prolyl-hydroxylation-dependent ubiquitination and subsequent proteasomal degradation [16]. Our recent data revealed that the reduced expression of MORG1 in *MORG1*^+/−^ mice improved renal damage in animal models of type 1 and type 2 diabetes mellitus [17,18] and ameliorated renal injury during systemic hypoxia [19]. Thus, our findings depicted that a better understanding of the potential mechanism of MORG1 regulation may eventually lead to the generation of novel therapeutic tools to treat hypoxic renal damage. 

## 2. Materials and Methods

### 2.1. Cells and Treatments

The regulation of the human MORG1 expression was studied in parental human HEK 293 cells (kindly provided by J. Mueller, Center of Molecular Biomedicine, Jena, Germany). HEK 293 cells were cultured in DMEM (Invitrogen, Schwerte, Germany) containing 4.5 g/L glucose, supplemented with 10% heat inactivated fetal bovine serum (Invitrogen, Germany), 100 U/mL penicillin and 0.1 mg/mL streptomycin (Sigma-Aldrich, Darmstadt Taufkirchen, Germany). For analyses, 3 × 10^5^ cells per well were seeded into 12 well plates. On the next day, the medium was replaced with DMEM, containing 1 g/L glucose, supplemented with 5% heat inactivated fetal bovine serum, (Invitrogen, Germany) and penicillin/ streptomycin. To study the effect of hypoxia on the *MORG1* expression in HEK 293 cells, they were exposed for 6, 12 or 24 h to 10% O_2_ in hypoxia cell culture incubator from TermoScientific (Schwerte, Germany), containing 5% CO_2_, 10% oxygen and 90% nitrogen gas. In addition to hypoxia, we studied the effect of two HIF-prolyl-hydroxylase (PHDs) inhibitors: 500 µM 3,4-dihydroxybenzoic acid (3,4-DHB) and 500 µM L-mimosine, both purchased from Sigma-Aldrich (Taufenkirchen, Germany). 

### 2.2. Reverse-Transcription and Quantitative Real-Time PCR Analyses

Total RNA was isolated from HEK 293 cells using the RNeasy kit (Qiagen GmbH, Hilden, Germany), according to the manufacturers’ recommendation. To assess the quality of the RNA, gel electrophoresis with RNA stained with SYBR Green dye (Molecular Probes) was performed on samples. RNA concentrations were determined, and one µg total RNA was reverse transcribed using an M-MLV-RT kit (Promega, Mannheim, Germany) for 1 h at 37 °C. The gene expression was estimated by real-time PCR (RT-PCR) in a multiplex assay as previously described [20] using 0.5 µM of forward reverse gene specific primers and a LightCycler^®^ 480 SYBR Green I Master Kit (Roche Diagnostics, Mannheim, Germany). The amplifications were performed using a Q-Tower thermocycler (Analytik Jena Bio Solutions, Jena, Germany). The sequences of the primers used for analyses were as follows: human *gapdh* forward 5′-GGAGTCAACGGATTTGGTCG-3′, reverse: 5′-CAGTGGACTCCACGACGTAC-3′, human *MORG1* forward 5′-AGGAATACAAGCTGGACTG-3′, reverse: 5′-AGCTACGTCTCTAGAATAAGATGG-3′, human *VEGFA* forward 5′-TTGCCTTGCTGCTCTACCTCCA-3′, reverse 5′-GATGGCAGTAGCTGCGCTGATA. The relative gene expression was quantified by ΔΔCT method, and the relative expression ratio was calculated according to Livak et al. [21], where *Ratio*  =  2^−ΔΔCT^ [21]. The primers sequences used for the analyses of the gene expression in HEK 293 cells were purchased from Invitrogen (ThermoFisher, Invitrogen, Life technologies, Darmstadt, Germany). Annealing temperatures and amplification conditions were as previously described [17,18]

### 2.3. Cloning of the Murine MORG1 Promoter and Generation of the Luciferase Reporter Constructs

A fragment corresponding to 1473 bp of the 5′ untranslated region of the murine MORG*1* genomic sequence upstream of the ATG codon was amplified from a DNA isolated from a BAC clone pMQ155E2 purchased from Source Bioscience, Nottingham, U.K., containing the murine genomic *MORG1* clone with the following primers: forward primer sequence 5′ GCAGCTGAGGGAGGGCGGA3′ and reverse primer sequence 5′ TGCATCAGTCCGCTGCCT 3′. The fragment was amplified with Phusion^TM^ High-Fidelity DNA polymerase (TermoFisher, Schwerte, Germany). After that, the PCR product was digested with *HindIII* endonuclease enzyme; the location of the internal *HindIII* restriction side is underlined on the murine promoter sequence. Next, the fragment was gel purified and cloned into the digested with *SmaI* and *HindIII* promoter-less pGL2-basic vector (Promega), containing the firefly luciferase gene as a reporter gene. The corresponding sequence of the murine *MORG1* promoter was confirmed by sequencing. 

Subsequently, we performed a specific analysis of the hypoxia-inducible factor binding sites on the h*MORG1* promoter by the generation of point mutations in the putative HRE binding sites, where the A/GCGTG was mutated to A/GAAAG in the corresponding constructs. The point mutations were generated using 100 ng of the WT *MORG1* promoter reporter construct as a template, 5 U/reaction of Pfu-Turbo DNA polymerase (Agilent Technologies, Waldbronn, Germany) and 125 ng of each forward and reverse primer, caring the mutated site of the putative human *MORG1* hypoxia-responsible element binding site (HBS). The reactions were performed in a final volume of 50 µL. The primer sequences used for the mutagenesis are presented in Table S1. The cycling conditions were as follows: 95 °C denaturation for 5 min, followed by 18 cycles of 95 °C for 30 s, 60 °C annealing for 45 s and 16 min elongation step. Next, the PCR reactions were digested with 5 U *DpnI* restriction endonuclease (TermoFisher, Schwerte, Germany) for 3 h at 37 °C and the enzyme activity was inactivated for 15 min at 75 °C. 5 µls of the *DpnI* treated reactions were transformed into *E. coli* XL-1 Blue competent cells. Plasmid DNA was isolated from several colonies and subjected to sequencing using the sequencing primers pGL2 forward: 5′ CAATGTATCTTATGGTACTG 3′ and pGL2 reverse: 5′ CCTTATGCAGTTGCTCTCCA 3′. For simplicity, the constructs of the human *MORG1* promoter caring the corresponding single mutations were termed HBS A, HBS B, HBS C and HBS D, respectively. For the generation of the double HBS-binding site mutations of the h*MORG1* promoter, the plasmids caring the single HBS A, HBS B and HBS C mutants were digested with *XhoI* and *HindIII* endonucleases (Analytic Jena, Jena, Germany). The corresponding 496 bp fragment, containing the mutated sites, was gel purified and ligated into *XhoI* and *HindIII* digested HBS D vector plasmid containing the HBS D single mutation. Thus, were generated the *MORG1* promoter constructs caring double mutations as follows: HBS A + D, HBS B + D and HBS C + D; the corresponding reporter constructs of the human *MORG1* promoter were named thereafter as the double sites HBS A + B, HBS B + D and HBS C + D. All mutations were confirmed by sequencing analyses.

### 2.4. Transfections and Reporter Analysis

To analyse the transcriptional activation of the h*MORG1* promoter 3 × 10^5^ HEK 293 cells were seeded into 12 well plates as described above and transiently transfected on the next day in DMEM medium, containing 1 g/L glucose, 5% heat inactivated serum and antibiotics. For transient transfections, 2 µg of the corresponding human WT *MORG1* promoter reporter constructs or mutants of the h*MORG1* promoter reporter plasmid were co-transfected with 0.5 µg of β-galactosidase control plasmid (TAKARA Bio, San Jose, CA, USA), using TurboFect transfection reagent (ThermoScientific, Schwerte, Germany) for 12 h. Thereafter, a fresh DMEM medium, supplemented with 1 g/L glucose, 5% heat inactivated serum, antibiotics and the corresponding stimuli, was added and the cells were incubated for different time periods at the absence (control, respectively normoxia samples) or at the presents of stimuli as shown under the figure legends. Finally, the cells were washed with sterile PBS and lysed in 100 µL of cell lysis reagent (Promega). The cell lysates were vortexed and centrifuged at 13,000 rpm for 15 min. The luciferase activity was determined on a Tecan Infinite M200 (Tecan, Austria GmbH, Grödig, Austria) with a luminometer mode using 20 µL of the cells lysates and 100 µL firefly luciferase substrate (Promega). The ability of 3,4-DHB, L-mimosine and hypoxic conditions to induce HIFs activation was assessed by transient transfection of the *pHIF-Luc* reporter plasmid (Signosis, Sunnyvale, CA, USA) [20] and 0.5 µg of a β-galactosidase control plasmid (TAKARA Bio). The corresponding *pHIF-Luc* reporter activity was assayed as described above.

### 2.5. Western Blot Analyses

The protein expression was analyzed using Western blots after separation of proteins on 10 or 12% SDS-PAGE gels. After stimulations as appropriate, the cells were washed with PBS and lysed in complete lysis M buffer, supplemented with protease inhibitors (Roche, Mannheim, Germany) and 100 mM of Na_3_VaO_4_ (Sigma-Aldrich). The protein concentration was detected in DeNovix, DS-11FX Spectrophotometer/Fluorometer (DeNovix Inc., Wilmington, DE, USA) under UV 280 mode. Protein expression was detected with ECL reagent (PerkinElmer, Waltham, MA, USA) and documented with a Fusion FX7 EDGE imaging system (Vilber Lourmat GmbH, Eberhardzell, Germany). Protein expressions were measured via ImageJ software and normalized to the corresponding loading control protein. For the detection of MORG1 protein, a rabbit polyclonal antibody generated in Biotrend (Berlin, Germany) was used in dilution 1: 1000. The equal loading of total protein or cytosolic fraction protein was monitored by the detection of vinculin expression using a mouse antibody to vinculin 1: 1000 dilution (Santa Cruz Biotechnology Inc., Heidelberg, Germany). For detection of HIF-1α protein (Abcam, Cambridge, UK, 1:1000) and for HIF-2α, an antibody from Novus Biologicals (Wiesbaden, Germany, 1:500) was used. The PCNA (Proliferating cell nuclear antigen) antibody was purchased from Santa Cruz Biotechnology Inc. and it was used in dilution 1:500. The corresponding secondary antibodies were used in dilution 1:2000 purchased from KPL (Sera Care, Gaithersburg, MD, USA). 

### 2.6. Biotin-11 UTP Labelling of ssDNA Oligonucleotides 

The primers for electrophoretic mobility assay were labelled using BIO 3′-END oligonucleotide labelling kit with Biotin 11-UTP (Jena Bioscience, Jena, Germany), according to the manufacturer’s instructions. In brief, 5 pmol ssDNA oligonucleotides were labelled with 50 pmol Biotin 11-UTP by terminal deoxynucleotidyl transferase (TdT) for 30 min at 37 °C. We evaluated the Biotin 11-UTP labelling by dot blot analyses. Afterwards, two complementary ssDNA oligonucleotides (sense and anti-sense) were annealed in annealing buffer containing: 10 mM TRIS-HCl pH 7.5; 1 mM EDTA and 50 mM KCl in a heating block at 95 °C for 5 min, then the mixture was allowed to cool down to 25 °C. 

### 2.7. Electrophoretic Mobility Shift Assay

We further analyzed the *MORG1* putative HIF binding sites by electrophoretic mobility shift assay (EMSA). Nuclear protein extracts for EMSA were prepared using a mammalian nuclear and cytoplasmic protein extraction kit (Serva Electrophoresis GmbH, Heidelberg, Germany), as recommended by the manufacturer. The protein concentration was measured in DeNovixDS-11FX Spectrophotometer (DeNovix Inc., Wilmington, DE, U.S.A) and the proteins were kept frozen at −20 °C until the analyses. The EMSA binning reactions were performed for all four putative HBS of the h*MORG1* promoter region by the aid of an active motif non-radioactive assay kit (BIOZOL Diagnostica, Eching, Germany). The reactions were performed in final volume of 20 µL and usually contained 10 µg of nuclear extracts, 1 µL of 10× reaction binding buffer, 1 µL of 2.5 mg/mL BSA, 1 µL of 50% glycerol, 2 µL of 0.1 mg/mL dI-dC, and 2 µL of 3′-end Biotin-11 UTP labelled dsDNA oligonucleotides. After that, the reactions were incubated for 30 min at room temperature and loaded on a 6% non-denaturing PAGE. The gel was transferred via semi-dry transfer system (Bio-Rad Laboratories Inc., Feldkirchen, Germany) on a positively charged membrane for 1 h. Next, the membrane was removed and placed side down on a UV-transiluminator Vilber (Vilber Lourmat GmbH, Eberhardzell, Germany) equipped with 4 × 8 W 365 nm UV-lamp and cross-linked for 15 min. The membrane was blocked for 1 h in blocking buffer, followed by 1 h incubation with a stabilized Streptavidin–HRP conjugate in 1:330 dilution in blocking buffer. The membrane was washed 4 times each 5 min with washing buffer. Finally, the membrane was equilibrated for 10 min in equilibration buffer and incubated with an EMSA-ECL solution for 10 min. The signals were visualized on a Fusion FX7 EDGE imaging system (Vilber Lourmat GmbH, Eberhardzell, Germany). All buffers, the streptavidin–HRP conjugate and ECL reagents were provided with the active motif assay kit (BIOZOL Diagnostica, Eching, Germany). For competitor studies to the binding reactions, either 100-fold excess of the corresponding unlabeled dsDNA oligonucleotides or 10-fold excess of unlabeled mutated dsDNA oligonucleotides were added. For super shift analyses, 1 µL of HIF-1α of HIF-2α antibodies from R&D System (Wiesbaden, Germany) were added for 15 min, prior to addition of the 3′-end Biotin-11 UTP labelled dsDNA oligonucleotides, followed by incubation for an additional 30 min at RT. The sequences of the oligonucleotides used in the EMSA analyses are shown in Supplementary Table S1. 

### 2.8. Statistics

Statistical analyses were performed with the statistical package SigmaPlot 14.5 software (SYSTAT Software, San Jose, CA, USA). The results were evaluated by Analysis of Variance (ANOVA) followed by a t-test to analyze the differences between the two groups. The data are graphically presented as a box plot, where the values are shown as the median and percentiles, and to the box plot was added additional a vertical point plot of all sample’s value. In the bar graphs, the values are presented as mean  ±  standard error of mean (SEM). Differences were considered significant when *p*  <  0.05. * *p*  <  0.05; ** *p*  <  0.01. *** *p*  <  0.001. 

## 3. Results

### 3.1. Cloning of the Murine and Human MORG1 Promoter and Identification of the Putative Transcription Factors Binding Sites on the Promoter Sequence

**Figure 1:**
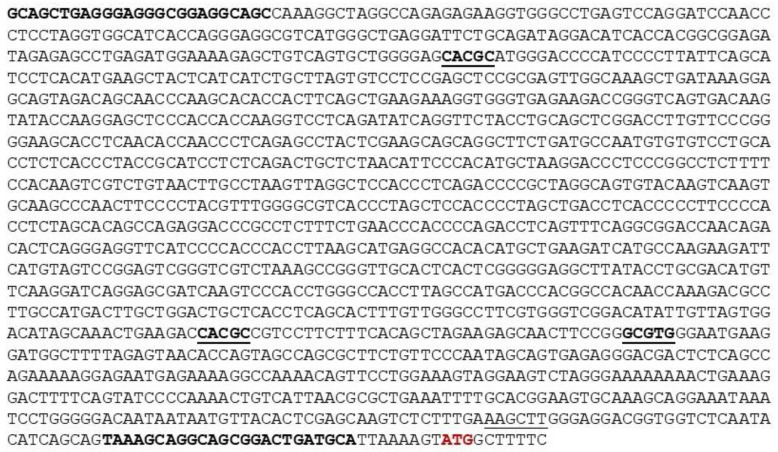
Sequence of the murine *MORG1 (WDR83)* promoter region. The putative HRE binding sites are underlined in bold. The primers used for amplification of the promoter region are shown in bold. The ATG start codon is shown in red. The internal *HindIII* restriction site is underlined.

To study the regulation of the *MORG1* promoter, we cloned the murine and human promoter regions of *MORG1* genes. We amplified 1473 bp fragment of the 5′ untranslated region of the murine *MORG1* gene using a BAC clone pMQ155E containing the murine *MORG1* genomic clone. The DNA sequence of the murine *MORG1* promoter is shown in Figure 1. 

**Figure 2:**
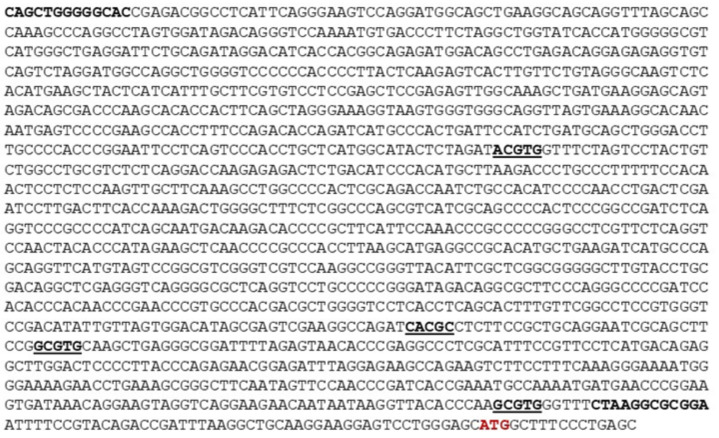
Sequence of the human MORG1 (WDR83) promoter region. The putative HRE binding sites are underlined in bold. The primers used for amplification of the promoter region are shown in bold. The ATG start codon is shown in red.

Furthermore, a 1512 bp fragment containing the 5′ untranslated region up stream of the ATG codon of the human *MORG1* promoter region was amplified from genomic DNA originated from human HEK 293 cell line. The DNA sequence of the human *MORG1* promoter is present in Figure 2.

**Figure 3:**
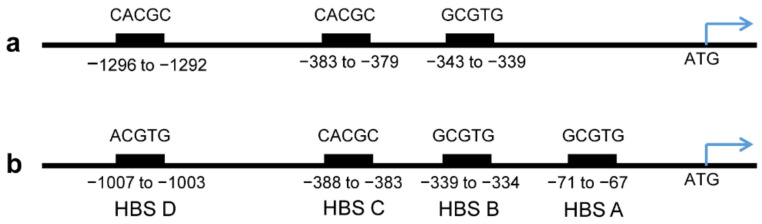
Schematic presentation of the distribution of the hypoxia-responsible element binding site on the promoter sequence of the murine and human MORG1 promoters. The positions of the HIF binding sites on the promoter regions are shown. (**a**) Murine MORG1 promoter. (**b**) Human MORG1 promoter. HBS: Hypoxia Binding Site.

To understand the regulatory network, which control the expression of the murine and human *MORG1* genes, we searched for a reliable identification of the transcription factor binding sites in the DNA sequence of the murine and human promoters. To predict binding sites of the putative transcription factors in murine and human *MORG1* promoter regions, we used the ALGGEN-PROMO link [22], based on TRANSFAC [23] database for potential binding sites in sequences. The results of the search for specific transcription factor binding sites in murine and human *MORG1* promoters DNA sequences are shown in Supplementary Table S2 and in Supplementary Table S3, respectively. Based on the predicted putative transcription factor (TF) binding sites in the promoter sequences, we found that both promoters contain multiple putative Sp1 (Specificity protein 1) TF binding sites, several potential NF-κB and AP-1 binding sites (see Supplementary Tables S2 and S3). Among other predicted TF binding sites, both the murine and the human *MORG1* promoters contain several hypoxia-responsible element (HRE) binding sites. The murine promoter contains three HRE binding motives characterized with the canonical hypoxia-inducible factor consensus sequence 5′-GCGTG-3′, which are shown in Figure 3a. The HREs in the murine *MORG1* promoter DNA sequence are located at positions −339 to −343, −379 to −383 and −1292 to −1296 relative to the ATG codon. The analyses of the putative HRE binding elements on the human *MORG1* promoter region revealed four consensus hypoxia-inducible transcription factors binding sites. Three of them are located in the proximal promoter region corresponding to positions −67 to −71, −334 to −339 and −383 to −388 relative to the ATG codon, and one HRE is located in the distal promoter at position −1003 to −1007 (Figure 3b). The comparison between the distribution of the HRE on the murine and human *MORG1* promoter sequences depicted a similarity in the locations of the putative HRE on both promoters. As it is shown on the schematic presentations of the HRE site for the murine (Figure 3a) and for the human (Figure 3b) MORG1 promoter regions, more sites are located in the proximal 400 bp of the promoter sequences. Even the HBS A on the human MORG1 promoter is in near proximity to the start codon, but this site is absent in the murine MORG1 promoter sequence. Interestingly, to this proximal HREs of the human promoter was identified the binding as well of ARNT- the aryl hydrocarbon receptor nuclear translocator, also known as HIF-1β and the AHR (aryl hydrocarbon receptor) (see Supplementary Tables S1 and S2).

### 3.2. Influence of Hypoxia and HIF Prolyl-Hydroxylase Inhibitors on hMORG1 mRNA and Protein Expression

**Figure 4:**
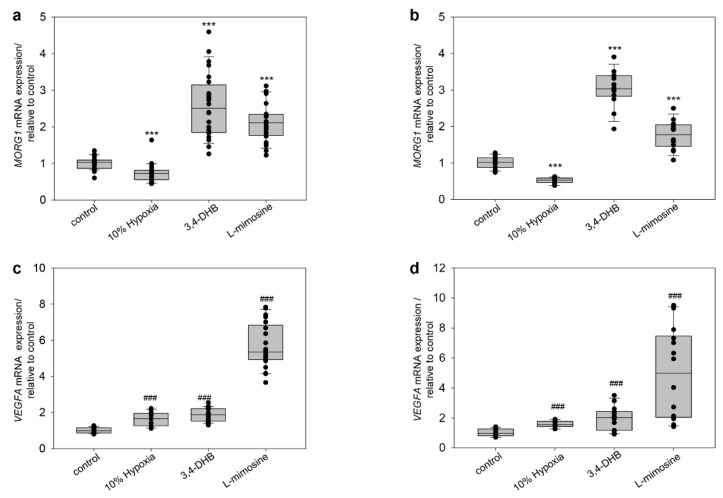
Influence of hypoxia, 3,4-DHB and L-mimosine on the *MORG1* and *VEGFA* mRNA expression in HEK 293 cells. Cells were left untreated or stimulated for the corresponding time period with 10% hypoxia, 500 µM 3,4-DHB or 500 µM L-mimosine. The expression of h*MORG1* and *VEGFA* mRNA were determined by real-time PCR analyses. The relative expression was normalized to *GAPDH* expression for each time point and presented as a fold expression relative to the untreated control, which was set as 1. (**a**) Analyses of the *MORG1* mRNA expression at 12 h treatments. N = 26/24/24/24. *** *p* < 0.001 vs. control. (**b**) Analyses of the *MORG1* mRNA expression at 24 h treatments. N = 14/12/14/14. *** *p* < 0.001 vs. control. (**c**) Analyses of the *VEGF A* mRNA expression at 12 h treatments. N = 21/12/21/21. ^###^ *p* < 0.001 vs. control. (**d**) Analyses of the *VEGF A* mRNA expression at 24 h treatments. N = 14/12/14/14. ^###^ *p* < 0.001 vs. control.

In this study, we further investigated the effect of hypoxic conditions and hypoxia mimetics only on human MORG1 in HEK 293 cells. We first assayed whether h*MORG1* mRNA expression is affected by reduced cellular oxygen supply and treatment with PHDs inhibitors 3,4-DHB and L-mimosine in HEK 293 by real-time PCR analyses. We exposed the cells for 12 h to 10% hypoxia, as well as 3,4-DHB and L-mimosine. We found that 10% hypoxia significantly reduced the levels of h*MORG1* mRNA compared to control, normoxic cells (Figure 4a, *p* < 0.001), while 3,4-DHB and L-mimosine significantly rose h*MORG1* expression (Figure 4a, *p* < 0.001). The exposure of the HEK 293 cells for 24 h at 10% hypoxia O2 supply also inhibited MORG1 gene expression levels relative to control (Figure 4b, *p* < 0.001). On the other hand, both PHD inhibitors significantly induced h*MORG1* expression in HEK 293 cells after 24 h treatment (Figure 4b, *p* < 0.001). To assess whether HIF target gene transcription was affected under the same experimental conditions, we examined the mRNA expression of the human *VEGFA* from the same cDNAs at 12 h and 24 h periods. Our data revealed that h*VEGFA* expression was significantly elevated under 10% hypoxia after 12 h (Figure 4c, *p* < 0.001) as well as after 24 h 10% hypoxia treatment relative to control, normoxic cells exposed to 21% oxygen (Figure 4c and Figure 4d, respectively; *p* < 0.001). Furthermore, the application of both PHD inhibitors 3,4-DHB and L-mimosine also significantly elevated the expression of h*VEGFA* mRNA in HEK 293 cells (Figure 4d, *p*< 0.001). Likewise, L-mimosine was a more potent inducer of h*VEGFA* mRNA expression, while 3,4-DHB produced a stronger h*MORG1* mRNA expression.

**Figure 5:**
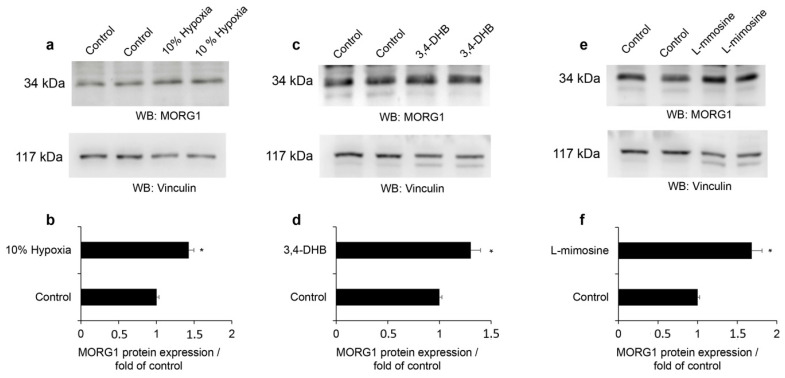
Influence of hypoxia, 3,4-DHB and L-mimosine on human MORG1 protein expression. Cells were left untreated or stimulated for 24 h with 10% hypoxia, 500 µM 3,4-DHB or 500 µM L-mimosine. MORG1 protein expression of was evaluated by Western blot analysis. The relative expression was normalized to vinculin expression and is graphically presented as a fold expression relative to untreated control, which was set as 1. (**a**) Analyses of the MORG1 protein expression in HEK 293 cells exposed to 10% hypoxia. Representative images are shown. (**b**) The graph represents the densitometry analyses of the MORG1 protein expression using Image J software. N = 4/4. * *p* < 0.05 vs. control. (**c**) Western blots for MORG1 protein in cells treated with 500 µM 3,4-DHB. Representative images are shown. (**d**) The graph represents the densitometry measurements of the MORG1 protein expression using Image J software. N = 4/4. * *p* < 0.05 vs. control. (**e**) Expression of MORG1 protein in cells treated with 500 µM L-mimosine. Representative images are shown. (**f**) The graph represents the densitometry measurements of the MORG1 protein expression in L-mimosine treated cells relative to control. N = 4/4. * *p* < 0.05 vs. control.

We further evaluated the protein expression of human MORG1 in HEK 293 cells using Western blots. The analyses of the protein expression in cells treated for 24 h with 10% hypoxia, 3,4-DHB or L-mimosine revealed that 10% hypoxia elevated the protein expression of MORG1 (Figure 5a,b, *p* < 0.05). The exposure of the cells to the PHD inhibitors 3,4-DHB (Figure 5c,d, *p* < 0.05) and L-mimosine (Figure 5e,f, *p* < 0.05) also significantly increased the expression of the MORG1 protein.

### 3.3. Influence of Hypoxia on the Reporter Activity of the Wild Type Human MORG1 Promoter

**Figure 6:**
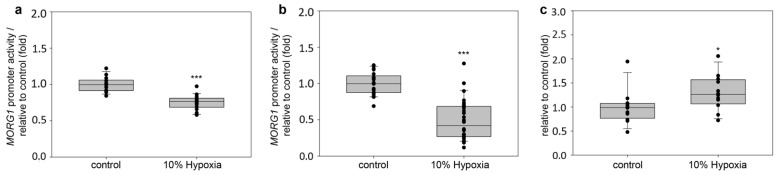
Influence of 10% hypoxia on the human *MORG1* promoter reporter activity. Relative *MORG1* promoter reporter activity in HEK 293 cells treated with 10% hypoxia (10% O_2_ supply) for different time periods. The corresponding controls were grown at 21% oxygen supply (normoxia conditions). (**a**) Relative *MORG1* promoter reporter activity at 6 h treatment. N = 14/18. *** *p* < 0.001 vs. control. (**b**) Relative *MORG1* promoter reporter activity at 12 h treatment. N = 21/28. *** *p* < 0.001 vs. control. (**c**) Relative *MORG1* promoter reporter activity at 24 h treatment. N = 12/12. * *p* < 0.05 vs. control.

We further continue our evaluation of the *MORG1* promoter regulation only on the human *MORG1* promoter region. It was already shown by Hopfer et al. that *MORG1* is expressed in human embryonic kidney cells (HEK 293 cells) [6]. The finding that four putative HRE binding sites are present on the sequence of the human *MORG1* promoter and its involvement in the hypoxia pathway through interaction with PHD3 [6] prompted us to investigate the regulation of the wild type of human *MORG1* promoter under hypoxic conditions. It was shown recently that the binding of hypoxia-inducible transcription factors HIF-1α and HIF-2α necessarily correlates with the degree of hypoxia [24]. Therefore, we assayed the h*MORG1* promoter reporter activity and transiently transfected the wild-type h*MORG1* reported construct in HEK 293 cells, which were left untreated or exposed to 10% hypoxia, in a hypoxia-cell culture incubator for 6 h, 12 h or 24 h. Our results reveal that, at 6 h, 10% hypoxia significantly inhibits h*MORG1* reporter activity compared with controls exposed to 21% O_2_ (Figure 6a, *p* < 0.001). Similarly, the exposure of the cells for 12 h to 10% hypoxia also decreased hMORG1 promoter reporter activity (Figure 6b, *p* < 0.001) relatively to normoxia-treated cells. Interestingly, 10% hypoxia upon 24 h significantly elevated MORG1 promoter activity in HEK 293 cells (Figure 6c, *p* < 0.05).

### 3.4. Influence of PHD Inhibitors on the Reporter Activity of the Wild-Type Human MORG1 Promoter

**Figure 7:**
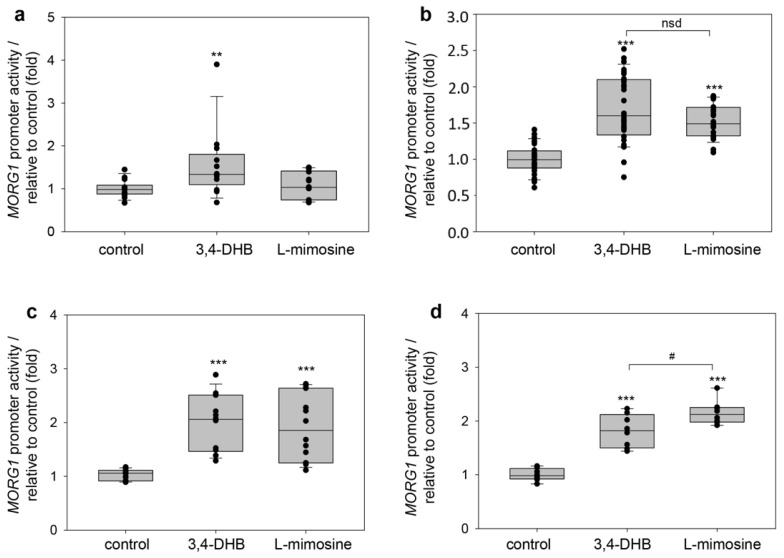
Influence of 3,4 DHB or L-mimosine on the human *MORG1* promoter reporter activity. Relative *MORG1* promoter reporter activity in HEK 293 treated with 3,4 DHB or L-mimosine) for different time periods. The corresponding controls were grown without the stimulators (controls). (**a**) Relative *MORG1* promoter reporter activity at 6 h treatment. N = 14/13/14. ** *p* < 0.01 vs. control. (**b**) Relative *MORG1* promoter reporter activity at 12 h treatment. N = 32/32/24. *** *p* < 0.001 vs. control. (**c**) Relative *MORG1* promoter reporter activity at 24 h treatment. N = 14/14/14. *** *p* < 0.001 vs. control. (**d**) Relative MORG1 promoter reporter activity at 48 h treatment. N = 8/8/8. *** *p* < 0.001 vs. control. ^#^ *p* < 0.05.

We evaluated the application of two small-molecules, 3,4-dihydroxybenzoic acid (3,4-DHB) and L-mimosine, which were already shown to effectively induce HIF accumulation and activation [25]. The application of the PHD inhibitors to the cells stimulated h*MORG1* promoter reported activity (Figure 7a–d). 3,4-DHB elevated h*MORG1* activity starting with 6 h time point (Figure 7a, *p* < 0.01), while L-mimosine was less effective at the early time point. A longer incubation with the stimuli for 12 h revealed that 500 µM L-mimosine similarly to 500 µM 3,4-DHB significantly increased h*MORG1* reporter activity (Figure 7b, *p* < 0.001) relative to untreated cells. We detected a sustained activation of the PHD inhibitors from 24 h up to 48 h, with a single dose applied to the cells (Figure 7c,d, respectively, *p* < 0.001) when compared to controls. In contrast to hypoxic conditions, the application of the PHD inhibitors significantly elevated the wild-type h*MORG1* reporter activity. Although the 3,4-DHB application was stimulated at earlier time points than L-mimosine *MORG1* promoter activation, with longer stimulation periods, L-mimosine had an equally enhancing effect as 3,4-DHB on the wild-type h*MORG1* reporter activity. We did not observe a significant difference in h*MORG1* promoter driven luciferase activity after 12 h, 24 h or 48 h, when the cells were stimulated with either PHD inhibitor.

### 3.5. Inhibitory Effect of 10% Hypoxia Is Reversible at the Early Time Point via the Application of 3,4 DHB 

**Figure 8:**
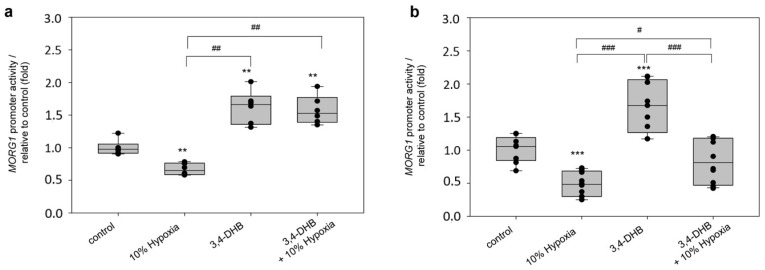
Application of 3,4-DHB reversed the hypoxia inhibitory effect on the *MORG1* promoter reporter activity at an early time point. HEK 293 cells were transiently transfected with the wild-type *MORG1* promoter construct and were left untreated (control) or treated with 500 µM 3,4-DHB or expose to 10% hypoxic conditions. To check the ability of 3,4-DHB to reverse the inhibitory effect of hypoxia on *MORG1* promoter activity in parallel experiments in addition to 10% hypoxia treatments was added as well 500 µM 3,4-DHB for different time periods and the promoter reported activity was assayed. (**a**). Relative *MORG1* promoter reporter activity at 6 h treatment of control, 10% hypoxia, 3,4-DHB and 10% hypoxia + 3,4-DHB. N = 6/6/6/6. ** *p* < 0.01 vs. control. ^##^ *p* < 0.01 vs. 10% hypoxia. (**b**). Relative *MORG1* promoter reporter activity in 12 h treated HEK 293 cells of control, 10% hypoxia, 3,4-DHB and 10% hypoxia + 3,4 DHB. N = 9/12/9/12. *** *p* < 0.01 vs. control. ^###^ *p* < 0.001 vs. 10% hypoxia. ^#^ *p* < 0.05 vs. 10% hypoxia.

The observation that PHD inhibitors activated h*MORG1* reporter activity, and especially, 3,4-DHB was more effective than L-mimosine in inducing h*MORG1* promoter reporter activity at an early time point, raised the question of whether an application of 3,4-DHB during hypoxic conditions could reverse the inhibitory effect of 10% hypoxia on h*MORG1* promoter reporter activity. First, we tested the ability of co-treatment of the cells with 500 µM 3,4-DHB and 10% hypoxia for 6 h period. We observed that the simultaneous application of 3,4-DHB and 10% hypoxia was able to reverse the inhibitory effect of hypoxia on human *MORG1* promoter activity (Figure 8a; *p* < 0.01, 10% hypoxia vs. 3,4-DHB +10% hypoxia). We next tested whether hypoxic inhibition on the *MORG1* reporter activity could be reversed after 12 h co-treatment with 3,4-DHB and 10% hypoxia. We measured a small, but significant, increase in the hMORG1 promoter activation when 3,4-DHB was added to 10% hypoxic conditions in HEK 293 cells (Figure 8b, *p* < 0.05, 10% hypoxia vs. 3,4-DHB +10% hypoxia). Nevertheless, 3,4-DHB was not able to reverse the hypoxia inhibitory effect after 12 h to levels comparable with 3,4-DHB alone (Figure 8b). Based on this original experiment, it could be hypothesized that there are differences between the hypoxic activation and pharmacological activation of HIFs in normoxic conditions, which need to be studied further in future experiments.

### 3.6. DHB, L-Mimosine and Hypoxic Conditions Induced HIF-Reporter Activity

To verify that under our experimental conditions HIFs are activated, we assayed the ability of a commercially available p*HIF-Luc*–reporter construct in transiently transfected HEK 293 cells to induce luciferase activity. We found that 3,4-DHB and L-mimosine significantly increased *pHIF-Luc* reporter activity at 6 h (Supplementary Figure S1a, *p* < 0.05). Similarly, 10% hypoxia also notably elevates *pHIF-Luc* reporter activity (Supplementary Figure S1c, *p* < 0.001). Considerably induced *pHIF-Luc* reporter activity was also detected in HEK 293 cells exposed for 12 h to 3,4-DHB or L-mimosine (Supplementary Figure S1b, *p* < 0.001). Furthermore, the exposure of the cells to 10% hypoxia for 12 h was also able to significantly induce HIF-driven luciferase activity (Supplementary Figure S1d, *p* < 0.001). Thus, the 10% hypoxia and PHD inhibitors used in our experimental settings can induce the activity of HIFs in HEK 293 cells. We also measured a stronger activation of the *pHIF-Luc* activity in L-mimosine treated cells compared with 3,4-DHB application or hypoxic conditions. Therefore, HIFs are activated from both mild hypoxia as well as by exposure to pharmacological inhibitors of PHD enzymes, although we measured a higher activation by hypoxia mimetics, compared to hypoxic conditions. Thus, our results support previous reports that 3,4-DHB and L-mimosine are capable to activate HIF reporter activity [25].

### 3.7. Evaluation of the HIF Binding Sites on the Wild-Type Human MORG1 Promoter by Mutational Analyses

**Figure 9:**
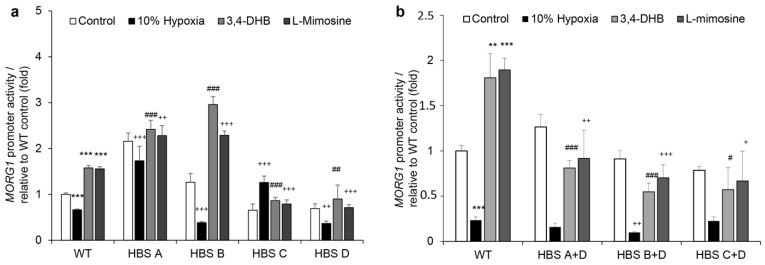
Mutational analyses of the human *MORG1* promoter reporter constructs. The influence of the individual HBS on the h*MORG1* promoter reporter activity was analyzed by the introduction of mutations in each individual site (single mutants) or at two HBS simultaneously (double mutants). (**a**) Influence of control, 10% hypoxia, 500 µM 3,4-DHB and 500 µM L-mimosine on the WT h*MORG1* and h*MORG1* single mutant reporter activity: HBS A, HBS B, HBS C and HBS D on the promoter activity after 12 h treatments. N = 9/9/9/9 for all treatments of WT h*MORG1* and all single mutants. *** *p* < 0.001 vs. control. ^##^ *p* < 0.01 vs. WT 3,4-DHB. ^###^ *p* < 0.001 vs. WT 3,4-DHB. ^+++^ *p* < 0.001 vs. WT 10% hypoxia. ^++^ *p*<0.01 vs. WT 10% hypoxia. ^+++^ *p* < 0.001 vs. WT L-mimosine. ^++^ *p* < 0.01 vs. WT L-mimosine. (**b**) Influence of control, 10% hypoxia, 500 µM 3,4-DHB and 500 µM L-mimosine on the WT h*MORG1* and h*MORG1* double mutants: HBS A + D, HBS B + D and HBS C + D on the promoter activity after 12 h treatments. N_WT_ = 9/9/9/9. N_HBS A+D_ = 9/9/8/9. N_HBS B+D_ = 9/9/9/9. N_HBS C+D_ = 6/6/6/6. ** *p* < 0.01 vs. control. *** *p* < 0.001 vs. control. ^###^ *p* < 0.001 vs. WT 3,4-DHB. ^#^ *p* < 0.05 vs. *MORG1* 3,4-DHB. ^+^ *p* < 0.05 vs. WT *MORG1* L-mimosine. ^++^ *p* < 0.01 vs. WT *MORG1* L-mimosine. ^+++^ *p* < 0.001 vs. WT *MORG1* L-mimosine.^++^ *p* < 0.01 vs. WT *MORG1* 10% hypoxia. HBS: Hypoxia Binding Site; WT; wild type.

We next performed a site-directed mutagenesis of the four putative HBS in the wild-type h*MORG1* promoter to investigate the functional significance of the HBS A, HBS B, HBS C and HBS D on h*MORG1* promoter activity. First, we performed single mutations of all four sites and analyzed their reporter activity in transiently transfected HEK 293 cells. The ability of the single mutants to affect h*MORG1* promoter activity was assayed for 12 h in control, 10% hypoxia-, 3,4-DHB- and L-mimosine-treated cells. Single mutants HBS A and HBS B significantly increased the 3,4-DHB- and L-mimosine-dependent activation of the h*MORG1* promoter in HEK 293 cells, compared to wild-type h*MORG1* promoter activity under the same experimental conditions (Figure 9a, *p* < 0.001). 

In contrast, HBS C mutation significantly reduced the h*MORG1* promoter-dependent reporter activity in the presence of 3,4-DHB or L-mimosine (Figure 9a, *p* < 0.001). Similar effects were observed when the HBS D mutation was transiently expressed (Figure 9a).

On the other hand, HBS A and HBS C h*MORG1* promoter mutants reversed the inhibitory effect of 10% hypoxia at 12 h treatment, while HBS B and HBS D single mutants further suppressed h*MORG1* promoter reporter activity (Figure 9a). These data suggest a possible inhibitory effect of HBS A and HBS B putative HIF binding sites during PHD inhibitor treatment at 12 h, while the HBS C and HBS D putative HIF binding sites presumably positively regulate hypoxia mimetic-dependent h*MORG1* promoter-induced reporter gene activity. 

We further generated double mutants of the h*MORG1* promoter caring at least two mutated putative HBS. We assayed the effect of the double mutants HBS A + D, HBS B + D and HBS C + D for their ability to influence the h*MORG1* promoter response after 12 h treatments when cells were exposed to 10% hypoxia, 3,4-DHB or L-mimosine and compared with the non-treated controls and wild-type h*MORG1* reporter activity under the same experimental settings. Reporter assays analyses revealed that all double mutants specifically reversed the 3,4-DHB- and L-mimosine-dependent activation of the h*MORG1* promoter, while being ineffective to reverse the inhibitory effect of 10% hypoxia (Figure 9b). The double mutant distribution on the wild-type h*MORG1* promoter is shown in Supplementary Figure S2. 

The observation that double mutations of putative hypoxia binding sites of the h*MORG1* promoter region could suppress the PHD inhibitor-dependent activation demonstrates that the detected stimulatory effect during normoxic PHD suppression could be related to HIF-1α binding to the putative HIF binding site(s) in the h*MORG1* promoter sequence. Our data revealed that the presence of the mutated HBS D site in all double mutants was able to inhibit the ability of the single HBS A and HBS B mutations to activate h*MORG1* reporter activity in 3,4-DHB- or L-mimosine-stimulated HEK 293 cells. Therefore, the putative HBS D in the *MORG1* promoter presumably positively regulates the human *MORG1* promoter reporter activity by hypoxia mimetics. Our results support the hypothesis that HIF binding sites in the promoter collectively affect its reporter capacity. Similarly, the presence of the mutated HBS D in the double mutants HBS A + D and HBS C + D was able to inhibit the ability of the single HBS A and HBS C mutations to activate h*MORG1* reporter activity in HEK 293 cells exposed to 10% hypoxia for 12 h. 

### 3.8. Evaluation of the Putative HRE Binding Sites of the hMORG1 Promoter by Electrophoretic Mobility Shift Assays after 12 h Treatments 

**Figure 10:**
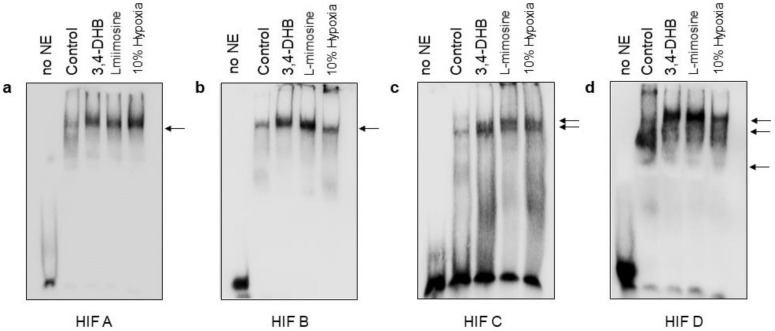
Evaluation of the putative HRE binding sites of the *MORG1* promoter region by EMSA. HEK 293 cells were exposed to 10% hypoxia or treated with 3,4-DHB and L-mimosine for 12 h followed by isolation of nuclear protein extracts (NE). The binding reactions between NE and double stranded 3′-Biotin 11–UTP labeled specific sequences corresponding to the putative *MORG1* promoter HRE binding motifs termed HBS A, HBS B, HBS C and HBS D were performed and separated on a 6% non-denaturing PAGEs. Representative images are shown. (**a**) HBS A. (**b**) HBS B. (**c**) HBS C. (**d**) HBS D. At least two experiments with qualitative similar results per binding site were performed. The development of the membranes revealed that in all HBS of the h*MORG1* promoter shifted bands were detected compared with the labeled oligos without NE (no NE lane on the images), thus shown a binding between nuclear proteins (transcription factors that bind to ds DNA sequences) and the analyzed ds sequences. The detected bands are shown with arrows on the images.

To analyze whether nuclear proteins bind the putative HRE-binding sequences of the h*MORG1* promoter, we assayed their binding ability by EMSA from HEK293 cells nuclear extracts prepared of untreated or stimulated cells with 3,4-DHB or L-mimosine or exposed to 10% oxygen supply for 12 h. We used 3′ 11-UTP Biotin labeled dsDNA oligonucleotides corresponding to HBS A (Figure 10a), HBS B (Figure 10b), HBS C (Figure 10c) and HBS D (Figure 10d) of the h*MORG1* promoter and performed an EMSA. We observed specific retarded complexes for all assayed HIFs’ putative binding sequences shown with an arrow in the images (Figure 10a–d). Our EMSA analyses depicted that the binding of the HBS A oligonucleotides was stronger in NE originated from cells exposed to 10% oxygen (Figure 10a) than 3,4-DHB- or L-mimosine-treated cells. We also detected that the HBS B and HBS D oligonucleotides bind more effectively to 3,4-DHB or L-mimosine NE and showed a weaker signal in NE from 10% hypoxia treated HEK 293 cells (Figure 10b and Figure 10d, respectively). The HBS C showed a lower binding ability to the NEthan the other HBS of the hMORG1 promoter at 12 h (Figure 10c). These results support our data from hMORG1 reporter assays, which showed a reduced activity when the cells were exposed to 10% hypoxia. EMSAs revealed that all HBS of the hMORG1 promoter form complexes with nuclear proteins, at our experimental conditions in HEK 293 cells, and could also have a direct inhibitory or stimulatory effect on the hMORG1 promoter activation. Furthermore, to confirm the specificity of the detected complexes on our EMSA gels, we analyzed the binding capacity of a well-investigated HIF-1α consensus site of *IL-8* promoter [26] (commercially available). We performed binding reactions with nuclear extracts (NE) from 10% hypoxia or 3,4-DHB treated HEK 293 cells, using labeled HIF-1α *MORG1* promoter putative HIF binding site or unlabeled competitor oligonucleotides (Supplementary Figure S3), and run all reactions together. The detected specific retarded complexes in binding reactions of the *IL-8* HIF-1α consensus site oligonucleotides, formed with NE from 10% hypoxia and 3,4-DHB treated HEK 293 cells, migrated similarly to the complexes, which were detected from the binding of the same 10% hypoxia or 3,4-DHB treated nuclear extracts with the HBS D binding site of the h*MORG1* promoter (Supplementary Figure S3). Moreover, competitor studies revealed no binding of the labeled oligos to the NE in *IL-8* HIF-1α consensus site binding reactions. Interestingly, the addition of the HIF-1α antibodies to the reaction did not show a super shift band, but instead reduced the binding ability of the labeled dsDNA oligos to the reaction. Thus, the super shift was presented as a competition assay between the corresponding antibody and the labelled dsDNA oligos, showing that HIF-1α was present in the complex with the *IL-8* HIF-1α consensus oligos. This analysis proved the equal migration capacity of the specific HIF-1α consensus and HBS D oligos. Thus, the migrated complexes in HBS D reactions are a result of the HIFs TF binding to the HBS D sequence. Therefore, all complexes detected in Figure 10a–d presumable correspond to HIFs TF binding.

### 3.9. Evaluation of the Putative HRE Binding Sites of the hMORG1 Promoter by Electrophoretic Mobility Shift Assay after 24 h Treatments

**Figure 11:**
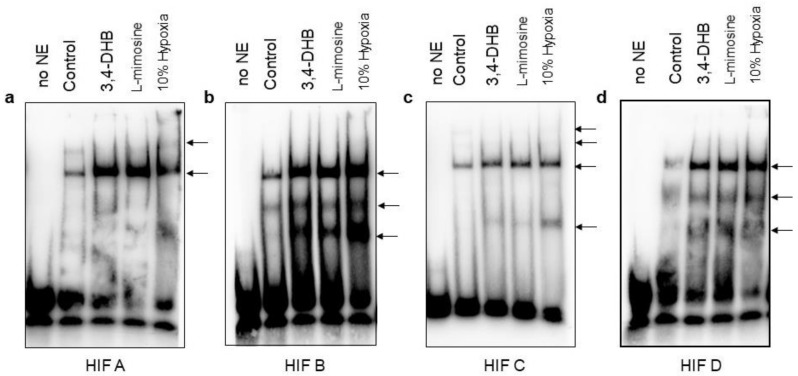
Influence of 10% hypoxia, 3,4-DHB and L-mimosine on the association between nuclear transcription factors and the putative HRE binding sites of the h*MORG1* promoter region by EMSA after 24 h treatments. The binding of the nuclear transcription factors to the h*MORG1* promoter putative HRE sequences termed HBS A, HBS B, HBS C and HBS D was investigated by EMSA assays using NE purified from HEK 293 cells exposed to 10% hypoxia, 3,4-DHB or L-mimosine for 24 h. Representative images are shown. The detected bands are shown with arrow on the images. (**a**) HBS A. (**b**) HBS B. (**c**) HBS C. (**d**) HBS D. The development of the EMSA gels revealed that the bands observed at 10% hypoxia NE were much stronger at HBS B and HBS D compared with the other putative *MORG1* HBS. A weak association was detected in HBS C, there was not a difference in the intensity of HBS C bands between 3,4-DHB, L-mimosine and 10% hypoxia.

Next, we assayed by EMSA the ability of putative HBS of the hMORG1 to bind to nuclear extracts from HEK 293 cells treated with 3,4-DHB or L-mimosine or exposed to 10% oxygen supply for 24 h (Figure 11a–d). We found specific retarded complexes in all assayed HBS. Furthermore, we found, in comparison to the NE from the 12 h treated cells, some additional bands in HBS B and HBS C (Figure 11b and Figure 11c, respectively), and the nuclear proteins binding ability to HBS C from 10% hypoxia NE increased (Figure 11c) relative to the 12 h treated cells. We also estimated the protein expression of HIF-1α and HIF-2α proteins in NE used for EMSA from 24 h treated cells. We detected both HIF-1α and HIF-2α proteins in the NE (Figure S4a and S4b, respectively) by Western blotting. We found more HIF-α protein in the NE from 3,4-DHB, L-mimosine and 10% hypoxia treated cells than HIF-2α, although the data are not yet significant due to the low number of NE lysates that were subjected to analyses. To evaluate the purity of the NE, we examined the protein expression of the cytoplasmic protein vinculin, as well as the TBP (TATA box binding protein) and PCNA (proliferating cell nuclear antigen) protein expression in the NE. We observed vinculin expression only in the lane of the cytoplasmic proteins and not in NE protein fractions (Supplementary Figure S4, cytoplasmic fraction lane). In addition, the expression of the TBP was detected only in the NE fractions and not in the cytoplasmic fractions, which confirms the purity of the NE used for analyses.

We also performed additional EMSA assays to analyze the binding specificity of the HBS A, HBS B and HBS D in NE from 3,4-DHB-treated cells (Supplementary Figure S5). We found that the binding reactions are specific, as the mutated oligos did not influence the binding of the labelled specific oligonucleotides to the reactions much. Moreover, we found that HBS A binds more to HIF-2α. We found that the binding reactions of HBS D oligos EMSAs show that both HIF-1α and HIF-2α TF could associate with this hMORG1 promoter-binding site, as the addition of HIF-1α or HIF-2α antibodies to the binding reaction, showed a competitor-like effect, thus reducing the binding of the HBS D oligonucleotides to the NE. We also tested the specificity of the formed DNA protein complexes of HBS A, HBS B and HBS D to the NE from the 12 h 10% hypoxia-treated cells. The resulting EMSAs are shown in Supplementary Figure S6. The binding reactions between the NE and HBS A, HBS B and HBS D are specific, as the mutated ds DNA oligonucleotides did not influence the binding of the labelled specific dsDNA oligonucleotides to the reactions much. Interestingly, we also detected a strong similarity between the supper shift analyses. Under hypoxic conditions, HIF-2α TF showed a higher affinity than HIF-1α TF to HBS A binding site. Both HIF-1α and HIF-2α transcription factors can bind to HBS D as observed in the supper shift assays, as less binding of the labeled ds HBS D DNA oligonucleotides were detected in the HIF-1α, and HIF-2α antibodies were added to the reactions. The HBS B showed a weak affinity to both HIF-1α and HIF-2α TFs. 

A detailed analysis using a CHIP assay could be useful for the precise detection of the binding ability of HIF-1α and HIF-2α transcription complexes under hypoxia conditions and the normoxic activation of HIFs by PHD inhibitors to each putative HIF binding site on the human MORG1 promoter sequence.

## 4. Discussion

The identification of the MAPK-organizer protein 1 (MORG1) as a binding partner of several binding modules provoked an interest in the function and regulation of the MORG1 gene and protein expression in health and diseased conditions. Although studies from MORG1^+/−^ mice revealed that reduced MORG1 expression is renoprotective and diminished the development of diabetic nephropathy in a mouse model of type 2 diabetes mellitus [18], it reduced renal injury in systemic hypoxia [19] and shows an anti-inflammatory function in septic AKI models [27]; the mechanisms regulating the MORG1 expression are not investigated. With this study, we cloned murine and human MORG1 promoter regions. Exploring the ALGGEN-PROMO link [23], we identified putative regulatory elements in both promoter sequences. The numerous predicted transcription factor binding sites in the murine and human MORG1 promoter regions need to be further investigated in more detail. Surprisingly, among other depicted putative TF binding site, several predicted hypoxia-responsible elements (HRE) binding motives were identified in both murine and human MORG1 promoter sequences corresponding to the canonical HRE binding sequence 5′-A/GCGTG-3. HIF binding motifs are located similarly in the murine and human MORG1 promoter sequences, except for the most proximal putative HRE binding site on the human promoter, the HBS A, which is situated just 67 bp upstream of the ATG codon. To this sequence has been also predicted the binding of ARNT, aryl hydrocarbon receptor nuclear translocator, also known as HIF-1β and the AHR (aryl hydrocarbon receptor). The heterodimer ARNT: AHR binds to the core DNA sequence 5′-TGCGTG-3′ within the dioxin response element (DRE) of the promoter sequences and activates their transcription [28]. Furthermore, environmental factors act through AHR receptor activation and regulate energy metabolism, and the disruption of energy metabolism [29] can result in metabolic diseases such as obesity [30], diabetes and hypertension [31]. This complex is closely related to hypoxia response elements as ARNT interacts with HIF-1α, HIF-2α in hypoxic conditions [32,33,34]. The interrelated roles of AHR and HIF-1α signaling play an important role in the coordination of such processes as infection and inflammatory diseases [33]. Thus, some of the predicted transcription factors binding sites correlate with our current knowledge well for the role of MORG1 in physiological and diseases conditions. Although we have more data supporting the involvement of MORG1 in the HIFs activation from animal studies, we selected to focus initially on the h*MORG1* promoter for our analyses, because understanding the mechanisms of *MORG1* regulation could be of potential therapeutic interest and, second, there are no data about the regulation and function of human MORG1. We explored the human HEK 293 cells for our studies as it was already shown that MORG1 is expressed in those cells [6]. It is well documented that murine MORG1 protein plays an important role in the stabilization and accumulation of HIFs via MORG1/PHD3 complex [6,17,20], but is unknown whether h*MORG1* expression is affected by hypoxic conditions. The finding that four putative hypoxia-inducible binding sites are present in the h*MORG1* promoter sequence raised our interest to study in more detail the ability of hypoxic conditions to affect the transcriptional regulation of the h*MORG1* promoter. Recent studies in HKC-8 cells have reported that HIF-1α and HIF-2α binding sites are independent of the duration and severity of hypoxia [24]. Our data revealed that mild hypoxic conditions, as 10% hypoxia in a time-dependent manner, affected the hMORG1 reporter activity. While early time points were associated with a significant inhibition of the wild-type hMORG1 promoter activity, 24 h exposure to mild hypoxia showed low, but significant, enchantment of the reporter gene activation. Therefore, we observed a biphasic effect of hypoxic conditions on the regulation of the hMORG1 promoter. We have to point at this point that we were able to confirm that HIFs were activated at 12 h of 10% hypoxia as we observed a significantly increased expression of the hVEGFA mRNA under above conditions. Furthermore, exploring HIF-reporter assays, we measured an increased luciferase activation after 6 h and 12 h in HEK 293 cells exposed to 10% hypoxia or treated with 3,4-DHB or L-mimosine. 

Although, in general, it is expected that the genes that are HIF targets in hypoxic conditions are positively regulated, characterized with an increased transcriptional activation [35], it was also reported that hypoxia can inhibit TNF-dependent VCAM1 induction in endothelial cells [36]. One explanation for the inhibitory effect of hypoxic conditions could be that at the first 6-to-12 h mild hypoxic conditions could induce the hypoxia-dependent activation of other transcription factor(s) regulated by HIFs, which in turn negatively regulate h*MORG1* transcriptional activity. Indeed, 10% hypoxia is approximately a 50% reduction of the normal oxygen supply. A very recent report demonstrated that even mild hypoxia can enhance HIF stabilization and the expression of VEGF and may induce destructive changes in kidney tissues [37]. On the other hand, hypoxia could lead to the reduced expression of several genes, regulated by transcription factors activated by hypoxic stress (for example HIF-1α activation is shown to induce an NF-κB activation [38]). It is also possible that the elevated *MORG1* reporter activation after 24 h exposure to mild hypoxia could be a result of the direct binding of HIFs to the putative HREs on the *MORG1* promoter. This binding may enhance the promoter reporter activity at the latter time point because the association is likely time dependent, or it is a result of competitive binding. 

In contrast to hypoxia-dependent HIF activation, the suppression of HIF hydroxylation by PHD enzymes under normoxic conditions, by the application of pharmacological inhibitors of PHDs, was associated with an enhanced hMORG*1* promoter activity starting at 6 h for the 3,4-DHB inhibitor. We detected a sustained h*MORG1* promoter reporter activation at 24 h and 48 h with a single treatment of the reagent. Furthermore, the application of L-mimosine, another PHD unspecific inhibitor, was less effective at the early time point, but its effect was undistinguishable from the 3,4-DHB action at 12 h, 24 h or 48 h treatments. Interestingly, these data suggest that small PHD inhibitor molecules probably need more initial time until HIF transcription factors accumulate and promote the h*MORG1* transcriptional activity. Therefore, HIFs could have a slower kinetic of accumulation at the presence of L-mimosine compared with 3,4-DHB treatment, which could be due to the slower effect of L-mimonsine on the suppression of the PHD enzymatic activity. Such a stronger, but slower, inhibitory action of L-mimosine on tyrosinase inhibition has already been reported [39]. Although both PHD inhibitors have common effects on the suppression of PHDs as well as the stabilization and activation of HIFs in normal conditions, it was shown that 3,4-DHB could suppress trophic factor deprivation-induced apoptosis partially in a HIF-independent manner in neuronal cells [40]. Consequently, the mechanisms used from both pharmacological PHD inhibitors to induce HIF activation could be slightly different and cell-type specific. Several studies reported that hypoxia mimetics induced preferentially HIF-2α activation, thus acting less on HIF-1α or even suppressing HIF-1α activation showing a mutual antagonism of HIF isoforms in cardiac, vascular and renal disorders [41]. Our data demonstrated that hypoxia and hypoxia mimetics induce HIF activation via different mechanisms, which may explain the differential effects observed on the regulation of h*MORG1* transcriptional regulation. This hypothesis is also supported from our experiments to reverse the hypoxic inhibition of h*MORG1* promoter by simultaneous exposure of the HEK 293 cells to 10% hypoxia and 3,4 –DHB. Although 3,4-DHB very potently reversed the h*MORG1* promoter reporter activation after 6 h, it was not able to overcome the hypoxic inhibition of the h*MORG1* promoter after 12 h co-treatment. This finding suggests that, at 12 h of mild hypoxia, different transcription factor(s) could be activated by HIFs indirectly, causing an inhibitory effect on *MORG1* transcriptional activity. Both HIF-1α and HIF-2α are expressed in HEK 293 cell, but the kinetic how they associate with the putative MORG1 HRE and how are activated from hypoxia or 3,4-DHB alone or in simultaneous treatment is unknown at present.

Furthermore, mutational analyses of the putative HIF binding sites on the h*MORG1* promoter revealed that single HBS A or HBS C mutants were able to reverse the inhibitory effect of 10% hypoxia compared with the wild-type h*MORG1* promoter reporter activity, suggesting that these sites have an inhibitory function on the h*MORG1* promoter at 12 h treatments. We detected opposite results when h*MORG1* reporter constructs carrying single mutation at the HBS B or HBS D were used. Interestingly, h*MORG1* reporter constructs carrying mutation in the HBS A and HBS B further increased the activity of the mutated promoter’s reporter constructs, compared with the wild-type h*MORG1* reporter activity, while HBS C and HBS D mutations were able to suppress the 3,4-DHB- or L-mimosine-dependent hMORG1 promoter activation at 12 h treatments. Therefore, these HIFs putative binding sites in the h*MORG1* promoter could be more sensitive to the pharmacological activation of HIFs. The analyses of the h*MORG1* promoter reporter constructs carrying two mutations revealed that the presence of the mutated HBS D binding site was sufficient to reduce the reverse the effects observed in the single mutants’ analyses. These data suggest that the distal putative HIF binding site in the h*MORG1* promoter sequence HBS D could play an essential role for the HIF-dependent regulation of the h*MORG1* promoter.

Moreover, HIFα subunits can also be hydroxylated at asparagine 851 blocking interactions with coactivators, suggesting an additional mechanism of regulation [42].

EMSA assays confirmed the complex specificity between the hMORG1 promoter putative HIF binding sites and HIF-1α and/or HIF-2α TFs, but is not yet clear how the exact complex formation occurs in hypoxic conditions and the pharmacological activation of HIFs via PHD suppression under normoxic conditions. Because the h*MORG1* promoter region contains four HIF binding sites, it is difficult to exactly determine whether there are preferential binding sites. It is expected that the proximal TF binding sites are probably positive regulators of the promoter activity, although we observed here that the HBS A presumable negatively regulate the h*MORG1* promoter activity in hypoxic conditions and by the pharmacological activation of HIFs in normoxic conditions through the suppression of PHD enzymes. In addition, *cis*-active elements may build complexes with other *trans*-factor and/or coactivators, further adding to the complexity of the regulation [43]. Further approaches, as a CHIP assay, bioinformatic approaches as well as functional motif analysis of the promotor, are necessary to be performed to learn more regarding the complex regulation of the MORG1 promoter under hypoxic conditions. In addition, experiments with other cell types will be of advantage to find out whether this complex regulation is cell-type specific. Nevertheless, our data provide for the first time evidence that not only *MORG1* regulates HIF stabilization through a PHD complex, but also, vice versa, HIFs control MORG1 expression directly or indirectly by a complicated regulatory mechanism, which needs to be further elucidated.

## Data Availability

The data are present in the manuscript and in Supplementary Materials. If more data are needed, it is available from the corresponding author upon request.

## Supplementary Materials

The following supporting information can be downloaded at: https://www.mdpi.com/article/10.3390/genes13030427/s1, Figure S1: Influence of hypoxic conditions and PHD inhibitors 3,4-DHB and L-mimosine on *pHIF-Luc* reporter activity; Figure S2: Graphic presentation of the double mutants on the human *MORG1* promoter sequence; Figure S3: Analyses of the specificity of the detected bands relative to IL-8 HIF-1α consensus sequence; Figure S4: Protein expression of HIF-1α and HIF-α in the nuclear fractions; Figure S5: Electrophoretic Mobility Shift Assay analyses of the specificity of the binding reactions in 3,4-DHB-treated HEK 293 cells; Figure S6: Electrophoretic Mobility Shift Assay analyses of the specificity of the binding reactions in 10% hypoxia treated HEK 293 cells; Table S1: Primer sequences used for EMSA assays; Table S2: Predicted TFBS based on the murine *MORG1* promoter sequence; Table S3: Predicted TFBS based on the human *MORG1* promoter sequence.
