# Supplementary material for: Cloning of the Human MORG1 Promoter: Differential Regulation by Hypoxia and Prolyl-Hydroxylase Inhibitors
Source: Genes (Basel). 2022 Feb 25;13(3):427. doi: 10.3390/genes13030427 (PMC8954370; doi:10.3390/genes13030427)
Supplement: Supplementary file 1 [file genes-13-00427-s001.zip › genes-1568895-supplementary/Supplementary Figures.pptx]

## Slide 1
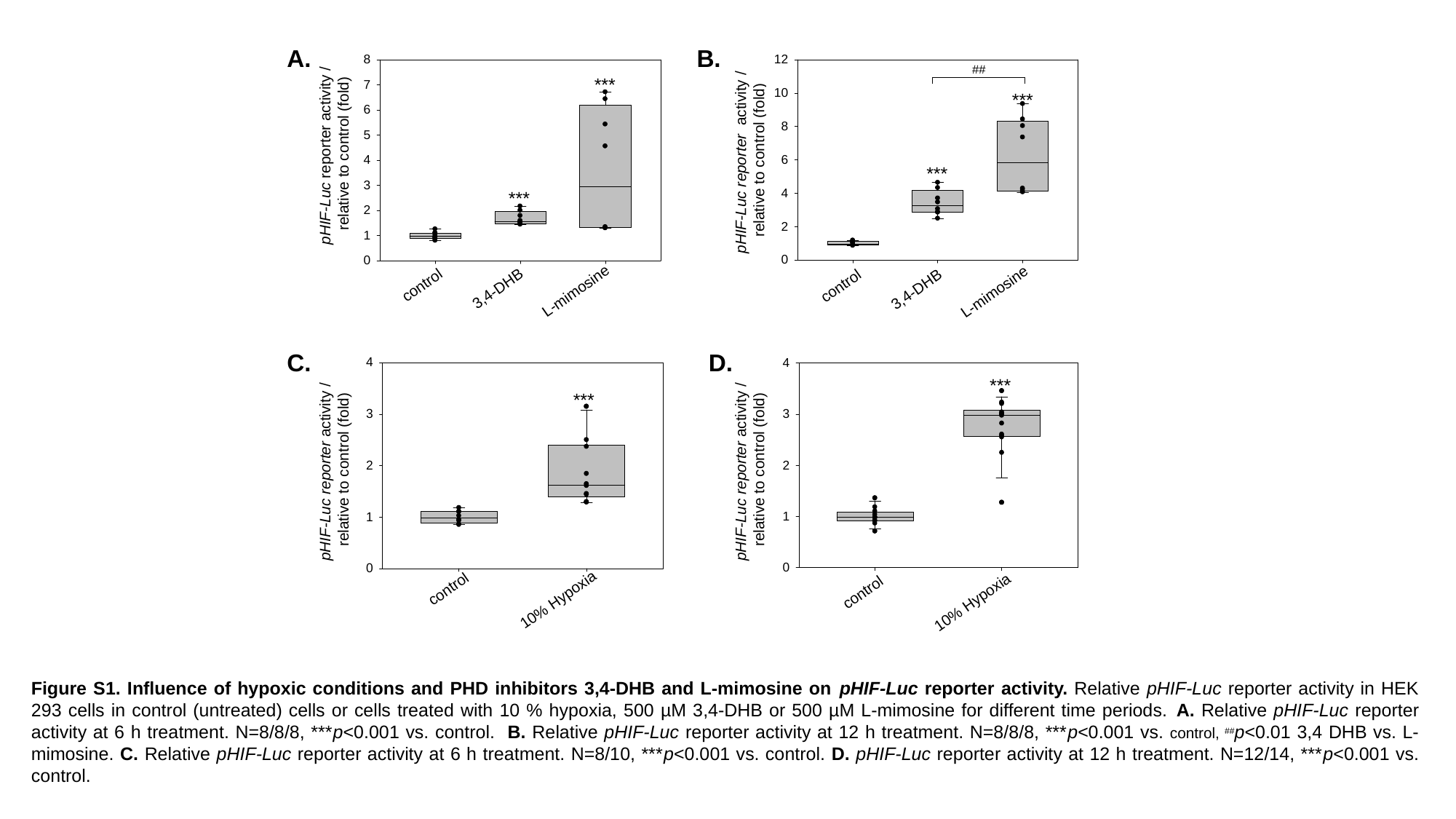

A.
B.
##
***
***
pHIF-Luc reporter activity /
relative to control (fold)
pHIF-Luc reporter activity /
relative to control (fold)
***
***
control
control
3,4-DHB
3,4-DHB
L-mimosine
L-mimosine
C.
D.
***
***
pHIF-Luc reporter activity /
relative to control (fold)
pHIF-Luc reporter activity /
relative to control (fold)
control
control
10% Hypoxia
10% Hypoxia
Figure S1. Influence of hypoxic conditions and PHD inhibitors 3,4-DHB and L-mimosine on pHIF-Luc reporter activity. Relative pHIF-Luc reporter activity in HEK 293 cells in control (untreated) cells or cells treated with 10 % hypoxia, 500 µM 3,4-DHB or 500 µM L-mimosine for different time periods. A. Relative pHIF-Luc reporter activity at 6 h treatment. N=8/8/8, ***p<0.001 vs. control. B. Relative pHIF-Luc reporter activity at 12 h treatment. N=8/8/8, ***p<0.001 vs. control, ##p<0.01 3,4 DHB vs. L-mimosine. C. Relative pHIF-Luc reporter activity at 6 h treatment. N=8/10, ***p<0.001 vs. control. D. pHIF-Luc reporter activity at 12 h treatment. N=12/14, ***p<0.001 vs. control.

## Slide 2
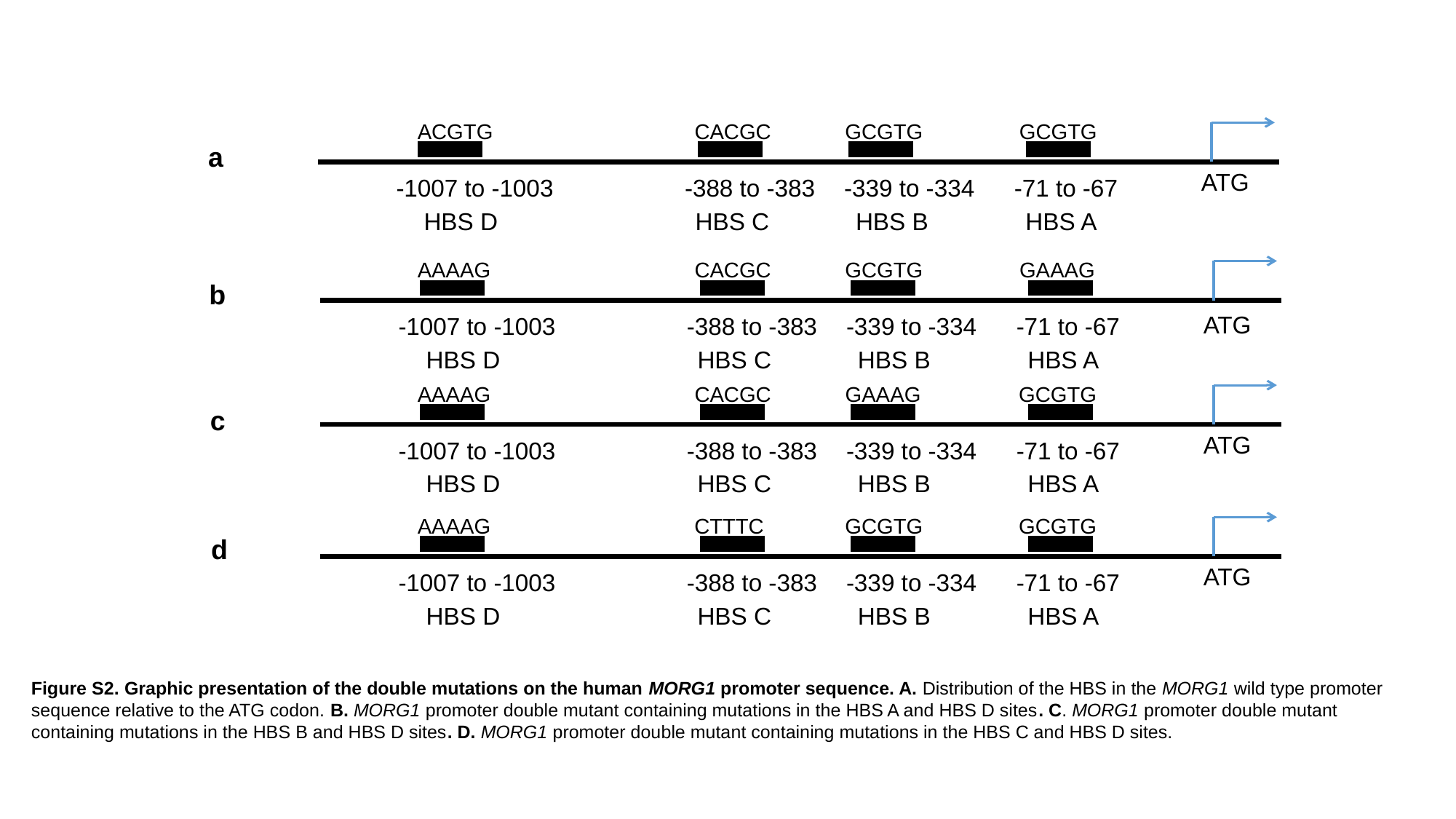

ACGTG
CACGC
 GCGTG
GCGTG
ATG
-1007 to -1003
-388 to -383
-339 to -334
-71 to -67
HBS D
HBS C
HBS B
HBS A
a
 AAAAG
CACGC
 GCGTG
GAAAG
ATG
-1007 to -1003
-388 to -383
-339 to -334
-71 to -67
HBS D
HBS C
HBS B
HBS A
b
 AAAAG
CACGC
 GAAAG
GCGTG
ATG
-1007 to -1003
-388 to -383
-339 to -334
-71 to -67
HBS D
HBS C
HBS B
HBS A
c
 AAAAG
CTTTC
 GCGTG
GCGTG
ATG
-1007 to -1003
-388 to -383
-339 to -334
-71 to -67
HBS D
HBS C
HBS B
HBS A
d
Figure S2. Graphic presentation of the double mutations on the human MORG1 promoter sequence. A. Distribution of the HBS in the MORG1 wild type promoter sequence relative to the ATG codon. B. MORG1 promoter double mutant containing mutations in the HBS A and HBS D sites. C. MORG1 promoter double mutant containing mutations in the HBS B and HBS D sites. D. MORG1 promoter double mutant containing mutations in the HBS C and HBS D sites.

## Slide 3
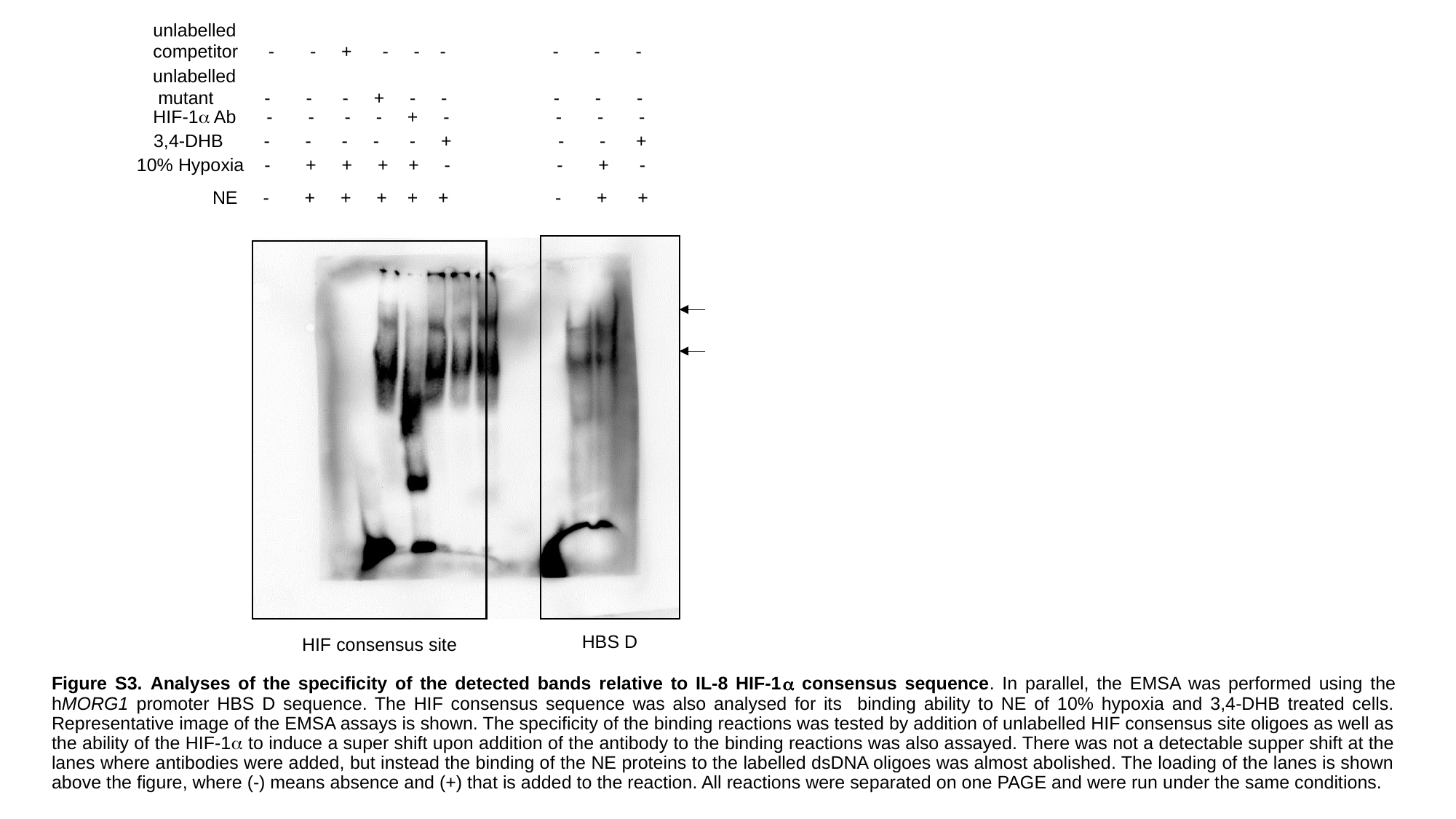

unlabelled
competitor - - + - - - - - -
unlabelled
 mutant - - - + - - - - -
HIF-1a Ab - - - - + - - - -
3,4-DHB - - - - - + - - +
10% Hypoxia - + + + + - - + -
NE - + + + + + - + +
HBS D
HIF consensus site
Figure S3. Analyses of the specificity of the detected bands relative to IL-8 HIF-1a consensus sequence. In parallel, the EMSA was performed using the hMORG1 promoter HBS D sequence. The HIF consensus sequence was also analysed for its binding ability to NE of 10% hypoxia and 3,4-DHB treated cells. Representative image of the EMSA assays is shown. The specificity of the binding reactions was tested by addition of unlabelled HIF consensus site oligoes as well as the ability of the HIF-1a to induce a super shift upon addition of the antibody to the binding reactions was also assayed. There was not a detectable supper shift at the lanes where antibodies were added, but instead the binding of the NE proteins to the labelled dsDNA oligoes was almost abolished. The loading of the lanes is shown above the figure, where (-) means absence and (+) that is added to the reaction. All reactions were separated on one PAGE and were run under the same conditions.

## Slide 4
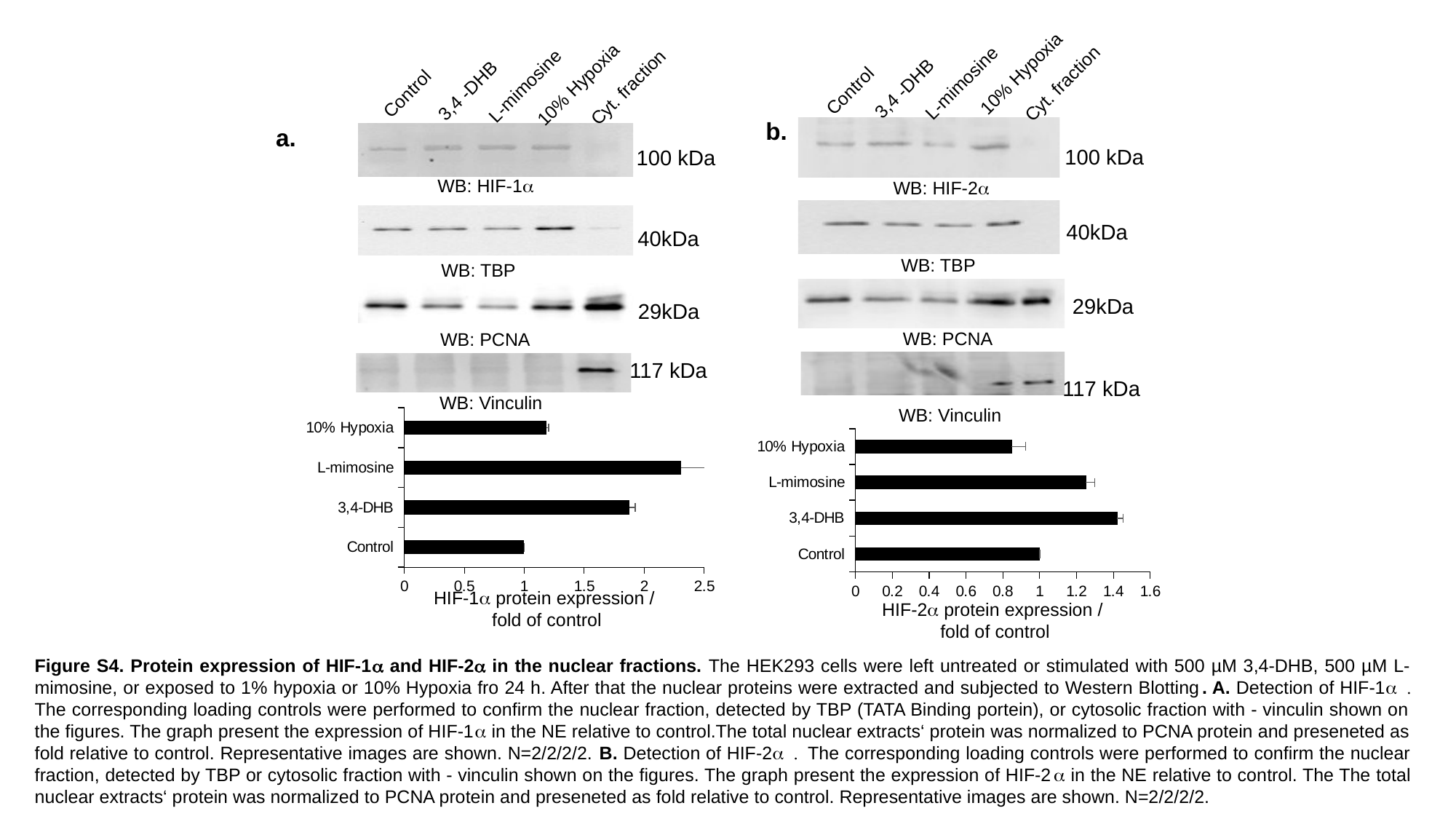

10% Hypoxia
L-mimosine
Cyt. fraction
3,4 -DHB
Control
b.
100 kDa
WB: HIF-2a
40kDa
WB: TBP
29kDa
WB: PCNA
117 kDa
WB: Vinculin
10% Hypoxia
L-mimosine
Cyt. fraction
3,4 -DHB
Control
a.
100 kDa
WB: HIF-1a
40kDa
WB: TBP
29kDa
WB: PCNA
117 kDa
WB: Vinculin
### Chart
| Category | |
|---|---|
| Control | 1.0 |
| 3,4-DHB | 1.878 |
| L-mimosine | 2.309 |
| 10% Hypoxia | 1.186 |
### Chart
| Category | |
|---|---|
| Control | 1.0 |
| 3,4-DHB | 1.42 |
| L-mimosine | 1.25 |
| 10% Hypoxia | 0.847 |HIF-1a protein expression /
fold of control
HIF-2a protein expression /
fold of control
Figure S4. Protein expression of HIF-1a and HIF-2a in the nuclear fractions. The HEK293 cells were left untreated or stimulated with 500 µM 3,4-DHB, 500 µM L-mimosine, or exposed to 1% hypoxia or 10% Hypoxia fro 24 h. After that the nuclear proteins were extracted and subjected to Western Blotting. A. Detection of HIF-1a . The corresponding loading controls were performed to confirm the nuclear fraction, detected by TBP (TATA Binding portein), or cytosolic fraction with - vinculin shown on the figures. The graph present the expression of HIF-1a in the NE relative to control.The total nuclear extracts‘ protein was normalized to PCNA protein and preseneted as fold relative to control. Representative images are shown. N=2/2/2/2. B. Detection of HIF-2a . The corresponding loading controls were performed to confirm the nuclear fraction, detected by TBP or cytosolic fraction with - vinculin shown on the figures. The graph present the expression of HIF-2a in the NE relative to control. The The total nuclear extracts‘ protein was normalized to PCNA protein and preseneted as fold relative to control. Representative images are shown. N=2/2/2/2.

## Slide 5
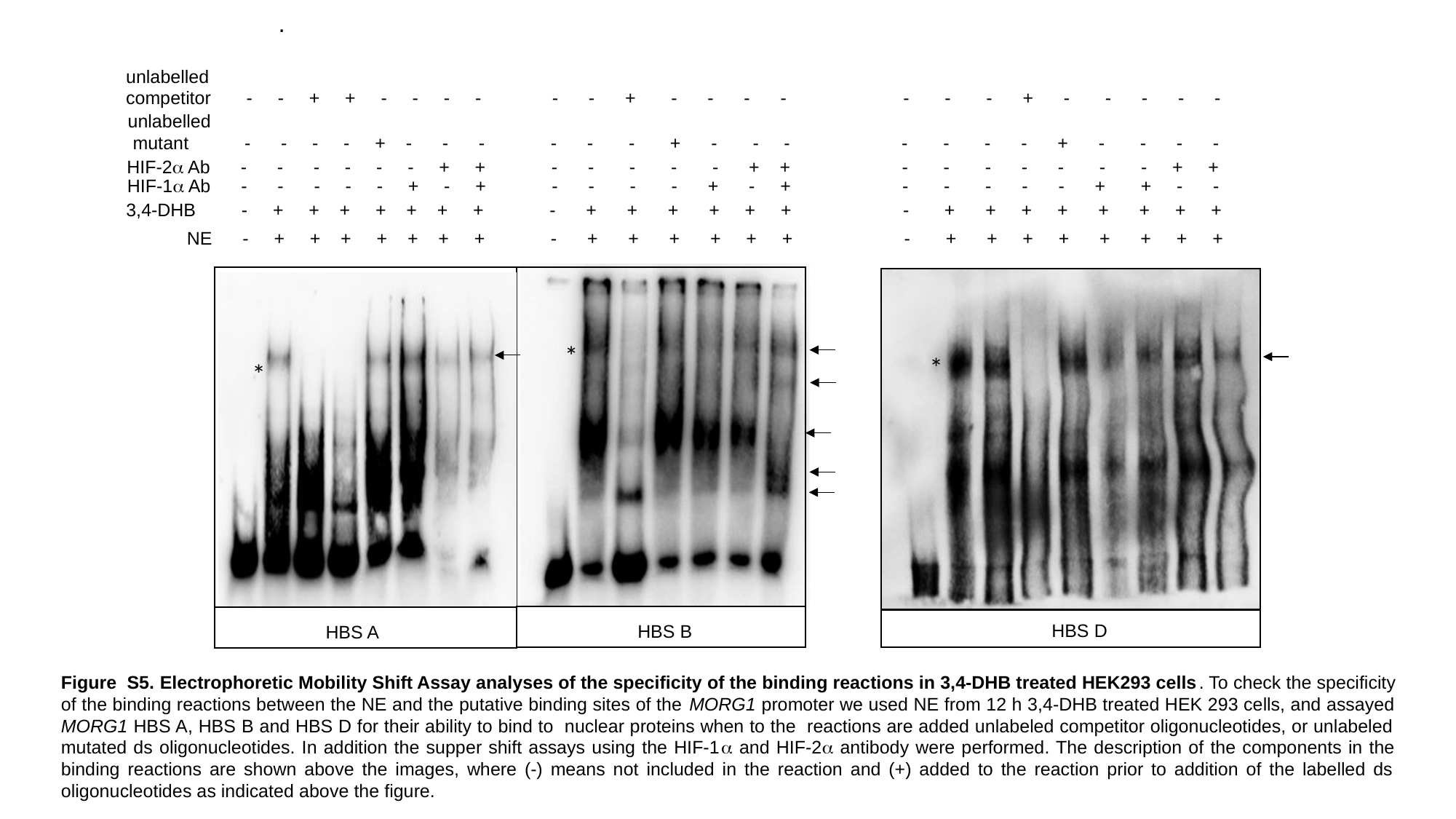

.
unlabelled
competitor - - + + - - - - - - + - - - - - - - + - - - - -
unlabelled
 mutant - - - - + - - - - - - + - - - - - - - + - - - -
HIF-2a Ab - - - - - - + + - - - - - + + - - - - - - - + +
HIF-1a Ab - - - - - + - + - - - - + - + - - - - - + + - -
3,4-DHB - + + + + + + + - + + + + + + - + + + + + + + +
NE - + + + + + + + - + + + + + + - + + + + + + + +
*
*
*
HBS D
HBS B
HBS A
Figure S5. Electrophoretic Mobility Shift Assay analyses of the specificity of the binding reactions in 3,4-DHB treated HEK293 cells. To check the specificity of the binding reactions between the NE and the putative binding sites of the MORG1 promoter we used NE from 12 h 3,4-DHB treated HEK 293 cells, and assayed MORG1 HBS A, HBS B and HBS D for their ability to bind to nuclear proteins when to the reactions are added unlabeled competitor oligonucleotides, or unlabeled mutated ds oligonucleotides. In addition the supper shift assays using the HIF-1a and HIF-2a antibody were performed. The description of the components in the binding reactions are shown above the images, where (-) means not included in the reaction and (+) added to the reaction prior to addition of the labelled ds oligonucleotides as indicated above the figure.

## Slide 6
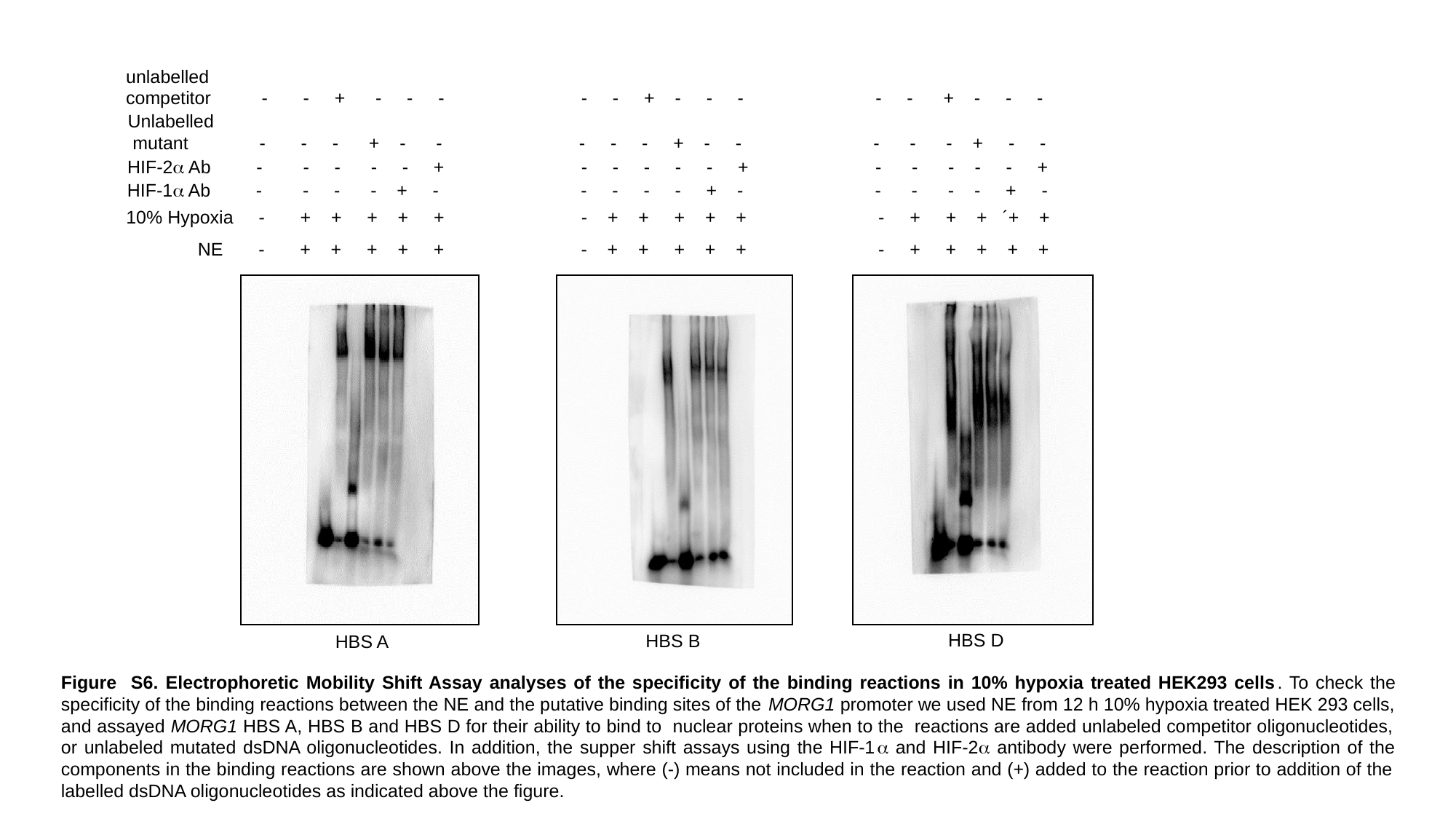

unlabelled
competitor - - + - - - - - + - - - - - + - - -
Unlabelled
 mutant - - - + - - - - - + - - - - - + - -
HIF-2a Ab - - - - - + - - - - - + - - - - - +
HIF-1a Ab - - - - + - - - - - + - - - - - + -
10% Hypoxia - + + + + + - + + + + + - + + + ´+ +
NE - + + + + + - + + + + + - + + + + +
HBS D
HBS B
HBS A
Figure S6. Electrophoretic Mobility Shift Assay analyses of the specificity of the binding reactions in 10% hypoxia treated HEK293 cells. To check the specificity of the binding reactions between the NE and the putative binding sites of the MORG1 promoter we used NE from 12 h 10% hypoxia treated HEK 293 cells, and assayed MORG1 HBS A, HBS B and HBS D for their ability to bind to nuclear proteins when to the reactions are added unlabeled competitor oligonucleotides, or unlabeled mutated dsDNA oligonucleotides. In addition, the supper shift assays using the HIF-1a and HIF-2a antibody were performed. The description of the components in the binding reactions are shown above the images, where (-) means not included in the reaction and (+) added to the reaction prior to addition of the labelled dsDNA oligonucleotides as indicated above the figure.
