# Supplementary material for: Cloning of the Human MORG1 Promoter: Differential Regulation by Hypoxia and Prolyl-Hydroxylase Inhibitors
Source: Genes (Basel). 2022 Feb 25;13(3):427. doi: 10.3390/genes13030427 (PMC8954370; doi:10.3390/genes13030427)
Supplement: Supplementary file 1 [file genes-13-00427-s001.zip › genes-1568895-supplementary/Table S1. Primers for EMSA assays.pdf]

Table S1. Primers' sequences for Electrophoretic mobility shift analyses of the human *MORG1* promoter putative HIF binding sites.

Name	Forward 5'- 3'	Reverse 5'- 3'
HIF A	TACACCCAAGCGTGGGTTTCTAAGGCGCG	CGCGCCTTAGAAACCCACGCTTGGGTGTA
HIF A mut	TACACCCAAGAAAGGGTTTCTAAGGCGCGGA	TCCGCGCCTTAGAAACCCTTTCTTGGGTGTA
HIF B	CAGCTTCCGGCGTGCAAGCTGAGGGCGGAT	ATCCGCCCTCAGCTTGCACGCCGGAAGCTG
HIF B mut	CAGCTTCCGGAAAGCAAGCTGAGGGCGGAT	ATCCGCCCTCAGCTTGCTTTCCGGAAGCTG
HIF C	GCCAGATCACGCCTCTTCCGCTGC	GCAGCGGAAGAGGCGTGATCTGGC
HIF C mut	GCCAGATCTTTCCTCTTCCGCTGC	GCAGCGGAAGAGGAAAGATCTGGC
HIF D	TACTCTAGATACGTGGTTTCTAGTCCTACT	AGTAGGACTAGAAACCACGTATCTAGAGTA
HIF D mut	TACTCTAGATAAAAGGTTTCTAGTCCTACT	AGTAGGACTAGAAACCTTTTATCTAGAGTA
HIF-1α IL8	TCTGTACGTGACCACACTCACCTC	GAGGTGAGTGGTCACGTACAGA
HIF-1α IL8 mut	TCTGTAAAAGACCACACTCACCTC	GAGGTGAGTGGTCTTTTACAGA

* The mutated primer sequences were used as well for generation of the corresponding mutations on the wild type *MORG1* promoter reporter constructs. The HIF binding sites and the mutated HRE binding sites on the sequences are underlined. The HIF-1α consensus binding sequence is based on the IL-8 promoter.
