# Supplementary material for: Cloning of the Human MORG1 Promoter: Differential Regulation by Hypoxia and Prolyl-Hydroxylase Inhibitors
Source: Genes (Basel). 2022 Feb 25;13(3):427. doi: 10.3390/genes13030427 (PMC8954370; doi:10.3390/genes13030427)

Table S2. Putative transcription factor binding sites on the murine *MORG1* promoter sequence.

Putative transcription factor binding sites on the murine *MORG1* promoter sequence identified by the aid of the ALGGEN-PROMO link (http://alggen.lsi.upc.es/cgi-bin/promo_v3/promo/promoinit.cgi?dirDB=TF_8.3), which use the TFBS defined in the TRANSFAC database to construct specific binding site weight matrices for TFBS prediction.

Factors predicted within a dissimilarity margin less or equal than 15 % :

Data (txt)

-- Input sequence --
>Sequence
GCAGCTGAGGGAGGGCGGAGGCAGCCAAAGGCTAGGCCAGAGAGAAGGTGGGCCTGAGTCCAGGATCCAA
CCCTCCTAGGTGGCATCACCAGGGAGGCGTCATGGGCTGAGGATTCTGCAGATAGGACATCACCACGGCGGA
GATAGAGAGCCTGAGATGGAAAAGAGCTGTCAGTGCTGGGGAGCACGCATGGGACCCCATCCCCTTATTCAG
CATCCTCACATGAAGCTACTCATCATCTGCTTAGTGTCCTCCGAGCTCCGCGAGTTGGCAAAGCTGATAAAGGA
GCAGTAGACAGCAACCCAAGCACACCACTTCAGCTGAAGAAAGGTGGGTGAGAAGACCGGGTCAGTGACAA
GTATACCAAGGAGCTCCCACCACCAAGGTCCTCAGATATCAGGTTCTACCTGCAGCTCGGACCTTGTTCCCGGG
GAAGCACCTCAACACCAACCCTCAGAGCCTACTCGAAGCAGCAGGCTTCTGATGCCAATGTGTGTCCTGCACCT
CTCACCCTACCGCATCCTCTCAGACTGCTCTAACATTCCCACATGCTAAGGACCCTCCCGGCCTCTTTTCCACAA
GTCGTCTGTAACTTGCCTAAGTTAGGCTCCACCCTCAGACCCCGCTAGGCAGTGTACAAGTCAAGTGCAAGCCC
AACTTCCCCTACGTTTGGGGCGTCACCCTAGCTCCACCCCTAGCTGACCTCACCCCCTTCCCCACCTCTAGCACA
GCCAGAGGACCCGCCTCTTTCTGAACCCACCCCAGACCTCAGTTTCAGGCGGACCAACAGACACTCAGGGAGG
TTCATCCCCACCCACCTTAAGCATGAGGCCACACATGCTGAAGATCATGCCAAGAAGATTCATGTAGTCCGGAG
TCGGGTCGTCTAAAGCCGGGTTGCACTCACTCGGGGGAGGCTTATACCTGCGACATGTTCAAGGATCAGGAGC
GATCAAGTCCCACCTGGGCCACCTTAGCCATGACCCACGGCCACAACCAAAGACGCCTTGCCATGACTTGCT
GGACTGCTCACCTCAGCACTTTGTTGGGCCTTCGTGGGTCGGACATATTGTTAGTGGACATAGCAAACTGAAG
ACCACGCCGTCCTTCTTTCACAGCTAGAAGAGCAACTTCCGGGCGTGGGAATGAAGGATGGCTTTTAGAGTAA
CACCAGTAGCCAGCGCTTCTGTTCCCAATAGCAGTGAGAGGGACGACTCTCAGCCAGAAAAAGGAGAATGAG
AAAAGGCCAAAACAGTTCCTGGAAAGTAGGAAGTCTAGGGAAAAAAAACTGAAAGGACTTTTCAGTATCCCC
AAAACTGTCATTAACGCGCTGAAATTTTGCACGGAAGTGCAAAGCAGGAAATAAATCCTGGGGGACAATAATA
ATGTTACACTCGAGCAAGTCTCTTTGAAAGCTTGGGAGGACGGTGGTCTCAATACATCAGCAGTAAAGCAGGC
AGCGGACTGATGCA

-- Factors predicted by PROMO in this sequence ---------------------
NAME; MATRIX_WIDTH;
Myf-3 [T00519]; 8
MyoD [T00525]; 8
MyoD [T01128]; 8
myogenin [T00528]; 7

MyoD [T00526]; 8
Nkx2-1 [T00857]; 7
COE1 [T01112]; 7
Elk-1 [T00250]; 5
WT1 I -KTS [T00900]; 10
WT1 -KTS [T01839]; 10
Sp1 [T00759]; 9
Sp1 [T00755]; 9
AP-2alphaA [T00035]; 6
GAL4 [T00302]; 8
CREMtau [T01309]; 7
CREMtau1 [T02108]; 7
CREMtau2 [T02109]; 7
MF3 [T00507]; 5
NF-1 [T00535]; 9
NF-1 [T00536]; 8
NF-1 [T00538]; 8
TGGCA-binding protein [T00832]; 7
LIM1 [T04817]; 4
C/EBPalpha [T00107]; 3
TCF-1A [T00999]; 9
LEF-1 [T02905]; 8
TCF-4E [T02878]; 7
HNF-3 [T02277]; 6
NFI/CTF [T00094]; 8
NF-AT4 [T01949]; 8
DEF:GLO:SQUA [T03217]; 7
SF-1 [T01147]; 9
NF-1 [T01298]; 6
GAGA factor [T00301]; 6
GA-BF [T00297]; 7
PBF [T02693]; 8
Zic1 [T04669]; 4
Zic2 [T04670]; 3
USF-1 [T00875]; 7
WT1 I [T01840]; 7
p300 [T01427]; 5
FACB [T02841]; 3
R2 [T00712]; 6
Nrf2:MafK [T05666]; 7
LVb-binding factor [T00477]; 7
HELIOS [T06012]; 6
RC2 [T00724]; 6
MYB2 [T02536]; 5
Zic3 [T04671]; 4
NF-1 [T00537]; 5
ENKTF-1 [T00255]; 8
TF68 [T00809]; 10
Msx-1 [T02072]; 3

RAR-gamma [T00720]; 8
ABF1 [T00056]; 6
ABI4 [T05743]; 6
Pax-9a [T03593]; 7
Pax-9b [T03594]; 7
XPF-1 [T00906]; 9
EBF [T05427]; 11
Sp3 [T02419]; 11
USF2 [T02115]; 6
c-Fos [T00123]; 8
MafG [T01437]; 5
YY1 [T00915]; 7
Pax-2 [T01823]; 7
Staf [T02247]; 12
YY1 [T00865]; 6
Mitf [T01554]; 13
NF-1 (-like proteins) [T00601]; 8
C/EBP [T01386]; 6
GATA-1 [T05705]; 7
NF-X3 [T01514]; 8
GATA-1 [T00306]; 6
GATA-3 [T00311]; 8
Pax-6 [T00682]; 5
HOXA3 [T00378]; 5
NHP-1 [T00621]; 7
Alfin1 [T04733]; 7
Vpr [T02399]; 8
Pax-2a [T00678]; 7
HES-1 [T01649]; 7
TCF-1(P) [T01109]; 10
STAT5A [T04683]; 4
IRF-1 [T00423]; 9
POU2F2C [T00665]; 8
POU2F2 [T01032]; 8
DEF:GLO [T03216]; 8
IRF-3 [T04673]; 7
MNB1a [T01059]; 6
NF-AT1 [T00550]; 9
Dl [T00196]; 9
TGIF [T04076]; 8
PKNOX1 [T04122]; 11
YY1 [T04970]; 9
E47 [T00207]; 7
MZF-1 [T00529]; 7
CP2 [T00152]; 8
Pax-5 [T00070]; 7
DBP [T00183]; 7
ZF5 [T02349]; 3
AhR [T00018]; 9

AhR:Arnt [T05394]; 9
PTF1-beta [T00701]; 10
PTF1 [T01227]; 11
MYBAS1 [T05553]; 5
AP-2alpha [T00033]; 9
YY1 [T00278]; 7
MCB1 [T06035]; 9
MCB2 [T06036]; 9
EIIaE-A [T00246]; 7
FOXO3a [T02938]; 8
USF [T00873]; 9
muEBP-C2 [T00215]; 6
USF-1 [T00877]; 11
c-Myc [T00143]; 7
LCR-F1 [T01599]; 6
Stra13 [T02327]; 11
STE12 [T00772]; 8
Pax-8 [T01828]; 6
FOXN2 [T04206]; 11
E2F-1 [T01542]; 7
f(alpha)-f(epsilon) [T00287]; 6
NF-1 [T00539]; 5
C/EBPbeta [T00017]; 8
HNF-4alpha2 [T02422]; 11
HNF-4alpha1 [T02429]; 11
GATA-1 [T00305]; 8
Ncx [T04368]; 3
GATA-1 [T00267]; 9
Cdx-1 [T01484]; 4
HOXD8 [T01426]; 7
HOXD8 [T01754]; 7
POU1F1b [T01516]; 8
POU1F1c [T01902]; 8
HNF-3beta [T02513]; 7
RFX1 [T01673]; 9
Nkx2-1 [T00856]; 6
DEC2 [T05845]; 10
AP-4 [T00036]; 11
Spz1 [T04668]; 7
VDR [T00885]; 4
E2 [T00205]; 6
GAMYB [T02679]; 7
T3R-alpha [T00841]; 9
ER-beta [T04651]; 9
RAR-beta [T00721]; 8
PPAR-alpha [T00694]; 7
ER-alpha [T00261]; 8
USF2b [T02377]; 8
c-Jun [T00131]; 6

JunB [T00436]; 6
c-Fos [T00124]; 7
c-Jun [T00132]; 7
c-Jun [T00133]; 7
JunD [T00437]; 7
SRY [T00997]; 7
c-Fos [T00122]; 8
TMF [T00835]; 7
MEDEA (MED) [T04379]; 11
CAC-binding protein [T00076]; 8
Yi [T00913]; 10
Ik-1 [T02702]; 13
HNF-4alpha [T05287]; 12
FXR:RXR-alpha [T05318]; 12
TFIIB [T00818]; 8
GATA-2 [T01302]; 8
Tal-1 [T01799]; 9
AREB6 [T00625]; 8
DSXF [T00955]; 7
DSXM [T00956]; 7
LVc [T00478]; 5
NF-1/L [T00599]; 13
En-1 [T02016]; 6
PR B [T00696]; 7
PR A [T01661]; 7
Sox2 [T01836]; 7
HMG I(Y) [T02368]; 7
STAT4 [T01577]; 6
GR-alpha [T00337]; 8
PR B [T00697]; 7
COE2 [T05006]; 9
GR-beta [T01920]; 8
c-Ets-1 [T00112]; 7
E2F [T01547]; 8
STAT6 [T01581]; 9
C1 (long form) [T01592]; 8
C1 (short form) [T01593]; 8
C1-I [T02946]; 8
c-Ets-1 [T00111]; 10
c-Ets-1 68 [T00115]; 10
NF-kappaB1 [T00593]; 12
HNF-3beta [T02344]; 7
MEF1 [T00506]; 10
LBM1 [T05245]; 12
RelA [T00595]; 11
c-Rel [T01154]; 10
XBP1 [T03707]; 10
Myf-5 [T00521]; 13
NF-E4 [T00560]; 10

NF-Y [T00150]; 8
POU2F1a [T00644]; 8
POU5F1 [T00651]; 8
LF-A1 [T00467]; 7
Cutl1 [T02042]; 6
POU1F1a [T00691]; 5
C/EBPdelta [T00109]; 3
unc-86 [T01882]; 5
POU2F1 [T00641]; 10
BR-C Z2 [T01478]; 5
POU2F2B [T00964]; 12
POU2F2 [T00647]; 11
POU2F2 [T00648]; 11
POU2F2 (Oct-2.1) [T01864]; 11
POU2F2 (Oct-2.3) [T01865]; 11
POU2F2 (Oct-2.4) [T01866]; 11
POU2F2 (Oct-2.6) [T01867]; 11
POU2F2 (Oct-2.1) [T01870]; 11
NF-Y [T00613]; 9
MATalpha2 [T00487]; 6
OSH15 [T05493]; 8
PEBP2alphaA1 [T01062]; 10
abaA [T01085]; 10
RelA [T00594]; 10
HSF1 (long) [T01042]; 8
HSF1 (short) [T02104]; 8
Zta [T00923]; 8
COE3 [T05008]; 13
AML1 [T01067]; 13
Nrf2 [T01443]; 11
MAZ [T00490]; 11
T-Ag [T00788]; 9
NF-AT3 [T02462]; 10
NF-AT2 [T01945]; 9
NF-AT1 [T01948]; 8
TRM1 [T05311]; 8
NF-AT1 [T01944]; 7
STAT1beta [T01573]; 10
DPBF-1 [T04363]; 10
DPBF-2 [T04364]; 10
TAF [T00778]; 7
c-Myb [T00137]; 8
C/EBPalpha [T00104]; 6
C/EBPalpha [T00105]; 6
C/EBPalpha [T00108]; 6
C/EBPbeta [T00459]; 6
C/EBPbeta [T00581]; 7
AP-3 (2) [T00039]; 7
BTEB3 [T05051]; 9

R1 [T00711]; 12
E2F-1 [T01543]; 9
AP-1 [T00029]; 8
WRKY2 [T03974]; 12
Nkx2-5 [T01675]; 9
WIZZ [T03807]; 11
C/EBPbeta(p20) [T01420]; 8
TCF-2 [T01110]; 10
p53 [T00671]; 7
GCR1 [T00322]; 11
TGA1a [T00829]; 6
E2F-5 [T01607]; 8
ETF [T00270]; 9
GBF [T00315]; 11
USF2 [T00878]; 9
CREMalpha [T01803]; 12
T3R-beta1 [T00851]; 11
CRE-BP2 [T01017]; 9
T3R-beta2 [T01350]; 9
ATF3 [T01095]; 8
RAR-alpha1 [T00719]; 10
ARP-1 [T00045]; 9
RXR-alpha [T01345]; 9
ATF-1 [T00968]; 11
CREBbeta [T02361]; 11
ER-alpha [T00264]; 10
CREB [T00989]; 10
ANT [T02639]; 11
AP-2 [T00034]; 10
MIG1 [T00509]; 11
VSF-1 [T02976]; 12
DRF1.1 [T05835]; 10
DRF1.3 [T05837]; 10
E2F-1:DP-1 [T05204]; 9
c-Ets-1 54 [T00114]; 6
Sp1 [T01228]; 11
Pu box binding factor [T00704]; 10
Smad3 [T04096]; 10
Smad4 [T04292]; 11
Zeste [T00918]; 7
Zeste [T02100]; 7
TFII-I [T00824]; 11
Egr-3 [T00243]; 13
Egr-3 [T05889]; 13
Pbx1 [T06000]; 12
RF2a [T02811]; 10
ALF1B [T01496]; 8
GCM [T02302]; 9
GCMa [T02306]; 9

GCMa [T02307]; 9
GCMb [T02308]; 9
octamer-binding factor [T01225]; 11
SXR:RXR-alpha [T05670]; 9
TBP [T00794]; 8
FXR [T04494]; 11
Pax-5 [T01201]; 7
E47 [T05421]; 10
Clock:Bmal1 [T05866]; 12
LIP15 [T02803]; 10
ROM1 [T02809]; 9
ROM2 [T02810]; 9
T3R-alpha [T00838]; 12
AP-1 [T01140]; 7
ERRalpha1 [T05682]; 13
Xvent-1 [T04665]; 8
mat1-Mc [T01275]; 13
LEF-1 [T00930]; 12
CUTL1 [T00100]; 6
ROX1 [T01286]; 10
POU6F1 [T04470]; 11
c-Myb [T00138]; 9
BR-C Z3 [T01479]; 10
Pbx1b [T02087]; 10
Fli-1 [T01408]; 13
NF-AT2 [T01947]; 13
Arnt [T01346]; 9
AhR [T01795]; 8
LyF-1 [T00479]; 9
GT-1 [T00339]; 8
Su(H) [T01615]; 13
PEA3 [T00685]; 8
MIF-1 [T01047]; 13
E12 [T01786]; 9
AP-3 [T01150]; 9
SPF1 [T03975]; 7
HOXD9 [T01424]; 10
HOXD10 [T01425]; 10
HOXD9 [T01755]; 10
HOXD9 [T01756]; 10
HOXD10 [T01757]; 10
HOXD10 [T01758]; 10
ANF [T00025]; 12
ANF [T01183]; 12
ANF [T01184]; 12
CIZ6-1 [T05137]; 10
FOXM1 [T04208]; 11
NBF [T00951]; 9
NBF [T01190]; 9

PPAR-gamma:RXR-alpha [T05236]; 12
ERR1 [T04849]; 11
GR [T00333]; 7
HNF-3alpha [T00371]; 8
c-Ets-2 [T01397]; 9
ELF-1 [T01113]; 13
STAT3 [T01493]; 12
IRF-2 [T00425]; 13
PEA3 [T00684]; 8
c-Ets-2 [T00113]; 8
Elk-1 [T05013]; 9
Hb [T00395]; 7
GATA-2 [T00308]; 9
AP-2beta [T02469]; 12
HNF-1B [T01950]; 8
Ubx [T00863]; 6
Crx [T03461]; 6
HNF-1A [T00368]; 8
HOX11 [T02054]; 7
SUT1 [T04582]; 10
POU3F1 [T00969]; 7
AGL3 [T03025]; 6
Pax-4a [T02983]; 6
POU3F2 [T00630]; 7
RAR-beta2 [T01326]; 12
HNF-3gamma [T01050]; 10
EFII [T00239]; 11
MAC1 [T01265]; 7
POU2F1 [T01031]; 10
E74A [T00208]; 10
GABP [T00268]; 12
Nkx6-2 [T02050]; 8
HNF-3alpha [T02512]; 10
NERF-1a [T05021]; 12
Croc [T02291]; 10
Olf-1 [T01040]; 13
Sox13 [T02420]; 8
Tll [T00789]; 6
MBF1 [T00492]; 11
Antp [T00026]; 6
Gt [T00328]; 11
Prd [T00699]; 7
IPF1 [T02057]; 8
C/EBPgamma [T00216]; 8
MRF-2 [T04675]; 10
TSAP [T00855]; 10
Adf-1 [T00008]; 8
MATa1 [T00488]; 10
PUR alpha [T05167]; 10

PUR beta [T05172]; 10
ATHB-5 [T04066]; 11
HNF-1C [T01951]; 9
POU4F1(l) [T01877]; 10
ARF1 [T04514]; 13
POU2F2 (Oct-2.1) [T00646]; 8
TBP [T00798]; 8
MED8 [T03491]; 9
FOXP3 [T04280]; 9
SBF-1 [T00739]; 9
HNF-1 [T00369]; 12
Meis-1a [T03388]; 9
Meis-1b [T03389]; 9
Elf-1 [T01019]; 10
HOXA9 [T01710]; 14
NF-AT4 [T01946]; 12
FOXD3 [T02290]; 8
HNF-3beta [T01049]; 8
AR [T00040]; 8
R [T00710]; 9
Hlf [T01071]; 9
Elf-1 [T05012]; 9
NRF-2 [T01199]; 11
Pax-6 [T01122]; 11
POU2F1 [T01466]; 8
HNF-3 [T00370]; 9
AIRE [T05990]; 10
REB [T02808]; 10
PPAR-alpha:RXR-alpha [T05221]; 11
Lmo2 [T02251]; 8
Tal-1 [T00790]; 11
Tal-1 [T01800]; 11
DTF-1 [T00201]; 11
MEF-2C/delta8 [T01769]; 11
B factor [T00061]; 8
TFIID [T00820]; 8
DEAF-1 [T05885]; 9
UME6 [T01247]; 11
dri [T04679]; 11
ADR1 [T00011]; 8
Sry-delta [T00767]; 13
IA-1 [T05887]; 13
T3R-alpha1 [T01152]; 11
T3R-beta1 [T00853]; 10
RORalpha1 [T01527]; 10
SF-1 [T04014]; 10
COUP-TF1 [T00149]; 9
T3R-alpha [T01351]; 9
SF-1 [T02769]; 11

RXR-beta [T01332]; 10
E4F1 [T00223]; 9
Sp3 [T02338]; 9
BTEB4 [T05053]; 9
Pbx1b [T02088]; 11
PHO4 [T00690]; 12
Ttk 69K [T00843]; 8
CTF [T00174]; 12
Oct-B1 [T00545]; 8
POU2F2B [T00662]; 8
POU2F1 [T00959]; 8
POU2F2B [T01871]; 8
POU5F1 (Oct-5) [T00653]; 9
POU3F1 [T00656]; 11
POU2F1b [T01862]; 11
POU2F1c [T01863]; 11
NF-kappaB [T00588]; 10
AR [T00042]; 7
NIT2 [T00627]; 8
NFe [T00565]; 12
mtTFA [T04783]; 10
RPN4 [T04539]; 9
StuAp [T02248]; 8
POU2F1 [T00643]; 11
Sp1 [T00753]; 6
Sp1 [T00752]; 10
Sp1 [T00754]; 10
Egr-1 [T01200]; 13
GKLF [T02450]; 13

-- PROMO predictions detail --

Sequence name; Factor name; Start position; End position; Dissimilarity; String; RE equally; RE query
Sequence; Myf-3 [T00519]; 0; 7; 1.122441; GCAGCTGA; 0.40457; 0.40913;
Sequence; Myf-3 [T00519]; 236; 243; 2.453540; TCATCTGC; 0.31467; 0.28208;
Sequence; Myf-3 [T00519]; 317; 324; 1.122441; TCAGCTGA; 0.40457; 0.40913;
Sequence; Myf-3 [T00519]; 960; 967; 0.000000; CCACCTGG; 0.40457; 0.47842;
Sequence; MyoD [T00525]; 0; 7; 1.122441; GCAGCTGA; 0.40457; 0.40913;
Sequence; MyoD [T00525]; 236; 243; 2.453540; TCATCTGC; 0.31467; 0.28208;
Sequence; MyoD [T00525]; 317; 324; 1.122441; TCAGCTGA; 0.40457; 0.40913;
Sequence; MyoD [T00525]; 960; 967; 0.000000; CCACCTGG; 0.40457; 0.47842;
Sequence; MyoD [T01128]; 0; 7; 1.122441; GCAGCTGA; 0.40457; 0.44102;
Sequence; MyoD [T01128]; 236; 243; 2.453540; TCATCTGC; 0.31467; 0.29935;
Sequence; MyoD [T01128]; 317; 324; 1.122441; TCAGCTGA; 0.40457; 0.44102;
Sequence; MyoD [T01128]; 960; 967; 1.122441; CCACCTGG; 0.40457; 0.44102;
Sequence; myogenin [T00528]; 0; 6; 0.000000; GCAGCTG; 0.17981; 0.22297;
Sequence; myogenin [T00528]; 1; 7; 2.411907; CAGCTGA; 0.26971; 0.32202;
Sequence; myogenin [T00528]; 21; 27; 11.564188; CAGCCAA; 0.17981; 0.20320;
Sequence; myogenin [T00528]; 102; 108; 9.044578; TGGGCTG; 0.44952; 0.57191;

Sequence; myogenin [T00528]; 164; 170; 5.289006; AGAGCTG; 0.26971; 0.30091;
Sequence; myogenin [T00528]; 173; 179; 5.289006; AGTGCTG; 0.26971; 0.30091;
Sequence; myogenin [T00528]; 211; 217; 5.879091; CAGCATC; 0.62933; 0.72409;
Sequence; myogenin [T00528]; 273; 279; 7.808616; AAAGCTG; 0.44952; 0.42325;
Sequence; myogenin [T00528]; 296; 302; 5.879091; CAGCAAC; 0.62933; 0.72409;
Sequence; myogenin [T00528]; 317; 323; 2.411907; TCAGCTG; 0.26971; 0.32202;
Sequence; myogenin [T00528]; 318; 324; 2.411907; CAGCTGA; 0.26971; 0.32202;
Sequence; myogenin [T00528]; 411; 417; 3.841862; CAGCTCG; 0.26971; 0.35143;
Sequence; myogenin [T00528]; 471; 477; 0.482381; CAGCAGG; 0.17981; 0.21833;
Sequence; myogenin [T00528]; 695; 701; 6.361472; CTAGCTG; 0.53943; 0.64423;
Sequence; myogenin [T00528]; 729; 735; 9.634663; CAGCCAG; 0.17981; 0.22297;
Sequence; myogenin [T00528]; 837; 843; 6.361472; CATGCTG; 0.53943; 0.64423;
Sequence; myogenin [T00528]; 1017; 1023; 6.361472; CTTGCTG; 0.53943; 0.64423;
Sequence; myogenin [T00528]; 1036; 1042; 5.289006; CAGCACT; 0.26971; 0.30091;
Sequence; myogenin [T00528]; 1116; 1122; 6.361472; CAGCTAG; 0.53943; 0.64423;
Sequence; myogenin [T00528]; 1179; 1185; 7.907558; CAGCGCT; 0.44952; 0.42325;
Sequence; myogenin [T00528]; 1219; 1225; 9.634663; CAGCCAG; 0.17981; 0.22297;
Sequence; myogenin [T00528]; 1327; 1333; 6.460414; CGCGCTG; 0.53943; 0.64423;
Sequence; myogenin [T00528]; 1443; 1449; 1.929525; CAGCAGT; 0.17981; 0.18383;
Sequence; myogenin [T00528]; 1458; 1464; 5.030459; CAGCGGA; 0.17981; 0.21833;
Sequence; MyoD [T00526]; 1; 8; 3.094314; CAGCTGAG; 0.15733; 0.18001;
Sequence; MyoD [T00526]; 163; 170; 10.472091; AAGAGCTG; 0.20229; 0.21406;
Sequence; MyoD [T00526]; 172; 179; 12.731190; CAGTGCTG; 0.38210; 0.38801;
Sequence; MyoD [T00526]; 211; 218; 12.751607; CAGCATCC; 0.22476; 0.22410;
Sequence; MyoD [T00526]; 272; 279; 13.500287; CAAAGCTG; 0.20229; 0.19598;
Sequence; MyoD [T00526]; 296; 303; 12.101233; CAGCAACC; 0.42705; 0.46685;
Sequence; MyoD [T00526]; 316; 323; 2.710437; TTCAGCTG; 0.17981; 0.21023;
Sequence; MyoD [T00526]; 318; 325; 2.710437; CAGCTGAA; 0.17981; 0.21023;
Sequence; MyoD [T00526]; 411; 418; 10.832676; CAGCTCGG; 0.24724; 0.25936;
Sequence; MyoD [T00526]; 471; 478; 1.491344; CAGCAGGC; 0.13486; 0.15138;
Sequence; MyoD [T00526]; 694; 701; 11.483048; CCTAGCTG; 0.31467; 0.34968;
Sequence; MyoD [T00526]; 836; 843; 10.937622; ACATGCTG; 0.24724; 0.25936;
Sequence; MyoD [T00526]; 1016; 1023; 10.287249; ACTTGCTG; 0.20229; 0.21406;
Sequence; MyoD [T00526]; 1036; 1043; 11.003740; CAGCACTT; 0.24724; 0.25936;
Sequence; MyoD [T00526]; 1116; 1123; 11.099171; CAGCTAGA; 0.24724; 0.25936;
Sequence; MyoD [T00526]; 1326; 1333; 14.421725; ACGCGCTG; 0.04495; 0.05794;
Sequence; MyoD [T00526]; 1443; 1450; 3.242086; CAGCAGTA; 0.15733; 0.18001;
Sequence; MyoD [T00526]; 1458; 1465; 7.643058; CAGCGGAC; 0.13486; 0.18244;
Sequence; Nkx2-1 [T00857]; 3; 9; 5.851350; GCTGAGG; 0.53943; 0.62196;
Sequence; Nkx2-1 [T00857]; 7; 13; 3.365659; AGGGAGG; 0.53943; 0.62196;
Sequence; Nkx2-1 [T00857]; 14; 20; 4.033796; GCGGAGG; 0.62933; 0.68611;
Sequence; Nkx2-1 [T00857]; 71; 77; 3.365659; CCTCCTA; 0.53943; 0.62196;
Sequence; Nkx2-1 [T00857]; 90; 96; 3.365659; AGGGAGG; 0.53943; 0.62196;
Sequence; Nkx2-1 [T00857]; 105; 111; 5.851350; GCTGAGG; 0.53943; 0.62196;
Sequence; Nkx2-1 [T00857]; 217; 223; 5.183213; CCTCACA; 1.25867; 1.25010;
Sequence; Nkx2-1 [T00857]; 251; 257; 3.365659; CCTCCGA; 0.53943; 0.62196;
Sequence; Nkx2-1 [T00857]; 388; 394; 5.183213; CCTCAGA; 1.25867; 1.25010;
Sequence; Nkx2-1 [T00857]; 439; 445; 4.490101; CCTCAAC; 0.44952; 0.55636;
Sequence; Nkx2-1 [T00857]; 452; 458; 5.183213; CCTCAGA; 1.25867; 1.25010;

Sequence; Nkx2-1 [T00857]; 504; 510; 5.183213; CCTCTCA; 1.25867; 1.25010;
Sequence; Nkx2-1 [T00857]; 522; 528; 5.183213; CCTCTCA; 1.25867; 1.25010;
Sequence; Nkx2-1 [T00857]; 560; 566; 1.361249; CCTCCCG; 0.26971; 0.36241;
Sequence; Nkx2-1 [T00857]; 568; 574; 5.183213; CCTCTTT; 1.25867; 1.25010;
Sequence; Nkx2-1 [T00857]; 614; 620; 5.183213; CCTCAGA; 1.25867; 1.25010;
Sequence; Nkx2-1 [T00857]; 703; 709; 5.851350; CCTCACC; 0.53943; 0.62196;
Sequence; Nkx2-1 [T00857]; 720; 726; 1.817554; CCTCTAG; 0.35962; 0.37561;
Sequence; Nkx2-1 [T00857]; 732; 738; 3.178803; CCAGAGG; 0.62933; 0.73053;
Sequence; Nkx2-1 [T00857]; 744; 750; 5.183213; CCTCTTT; 1.25867; 1.25010;
Sequence; Nkx2-1 [T00857]; 767; 773; 5.183213; CCTCAGT; 1.25867; 1.25010;
Sequence; Nkx2-1 [T00857]; 797; 803; 3.365659; AGGGAGG; 0.53943; 0.62196;
Sequence; Nkx2-1 [T00857]; 825; 831; 3.178803; CATGAGG; 0.62933; 0.73053;
Sequence; Nkx2-1 [T00857]; 911; 917; 4.033796; GGGGAGG; 0.62933; 0.68611;
Sequence; Nkx2-1 [T00857]; 1033; 1039; 5.851350; CCTCAGC; 0.53943; 0.62196;
Sequence; Nkx2-1 [T00857]; 1203; 1209; 5.183213; TGAGAGG; 1.25867; 1.25010;
Sequence; Nkx2-1 [T00857]; 1418; 1424; 3.365659; TGGGAGG; 0.53943; 0.62196;
Sequence; COE1 [T01112]; 5; 11; 2.523907; TGAGGGA; 0.53943; 0.57717;
Sequence; COE1 [T01112]; 88; 94; 0.459822; CCAGGGA; 0.08990; 0.11085;
Sequence; COE1 [T01112]; 177; 183; 1.751598; CTGGGGA; 0.44952; 0.51337;
Sequence; COE1 [T01112]; 189; 195; 1.247931; CATGGGA; 0.35962; 0.39778;
Sequence; COE1 [T01112]; 201; 207; 5.642184; TCCCCTT; 0.53943; 0.57341;
Sequence; COE1 [T01112]; 373; 379; 7.207716; TCCCACC; 0.53943; 0.61896;
Sequence; COE1 [T01112]; 426; 432; 2.955683; TCCCGGG; 0.26971; 0.32212;
Sequence; COE1 [T01112]; 428; 434; 1.291776; CCGGGGA; 0.26971; 0.31170;
Sequence; COE1 [T01112]; 543; 549; 3.771837; TCCCACA; 0.17981; 0.18978;
Sequence; COE1 [T01112]; 562; 568; 7.765914; TCCCGGC; 0.35962; 0.43781;
Sequence; COE1 [T01112]; 660; 666; 2.206305; TCCCCTA; 0.44952; 0.51337;
Sequence; COE1 [T01112]; 714; 720; 6.561828; TCCCCAC; 0.35962; 0.40436;
Sequence; COE1 [T01112]; 795; 801; 1.834174; TCAGGGA; 0.44952; 0.51337;
Sequence; COE1 [T01112]; 808; 814; 6.561828; TCCCCAC; 0.35962; 0.40436;
Sequence; COE1 [T01112]; 909; 915; 1.981509; CGGGGGA; 0.44952; 0.51337;
Sequence; COE1 [T01112]; 958; 964; 7.207716; TCCCACC; 0.53943; 0.61896;
Sequence; COE1 [T01112]; 1139; 1145; 2.397486; CGTGGGA; 0.53943; 0.57717;
Sequence; COE1 [T01112]; 1191; 1197; 6.977805; TCCCAAT; 0.53943; 0.61896;
Sequence; COE1 [T01112]; 1205; 1211; 5.959785; AGAGGGA; 0.53943; 0.60435;
Sequence; COE1 [T01112]; 1275; 1281; 0.919644; CTAGGGA; 0.35962; 0.39778;
Sequence; COE1 [T01112]; 1308; 1314; 3.125949; TCCCCAA; 0.26971; 0.32212;
Sequence; COE1 [T01112]; 1371; 1377; 3.355860; TGGGGGA; 0.35962; 0.43479;
Sequence; COE1 [T01112]; 1416; 1422; 2.167575; CTTGGGA; 0.44952; 0.51337;
Sequence; Elk-1 [T00250]; 9; 13; 5.092035; GGAGG; 1.43848; 1.76187;
Sequence; Elk-1 [T00250]; 16; 20; 5.092035; GGAGG; 1.43848; 1.76187;
Sequence; Elk-1 [T00250]; 56; 60; 6.853012; AGTCC; 4.31543; 4.28952;
Sequence; Elk-1 [T00250]; 62; 66; 6.274504; GGATC; 2.87695; 3.26211;
Sequence; Elk-1 [T00250]; 63; 67; 6.274504; GATCC; 2.87695; 3.26211;
Sequence; Elk-1 [T00250]; 71; 75; 5.092035; CCTCC; 1.43848; 1.76187;
Sequence; Elk-1 [T00250]; 92; 96; 5.092035; GGAGG; 1.43848; 1.76187;
Sequence; Elk-1 [T00250]; 110; 114; 6.853012; GGATT; 4.31543; 4.28952;
Sequence; Elk-1 [T00250]; 124; 128; 6.081668; GGACA; 2.87695; 2.78753;
Sequence; Elk-1 [T00250]; 139; 143; 7.213232; GGAGA; 4.31543; 4.28952;

Sequence; Elk-1 [T00250]; 159; 163; 2.121196; GGAAA; 1.43848; 1.33404;
Sequence; Elk-1 [T00250]; 181; 185; 7.406067; GGAGC; 1.43848; 1.75385;
Sequence; Elk-1 [T00250]; 193; 197; 6.274504; GGACC; 2.87695; 3.26211;
Sequence; Elk-1 [T00250]; 199; 203; 3.960472; CATCC; 2.87695; 3.26551;
Sequence; Elk-1 [T00250]; 214; 218; 3.960472; CATCC; 2.87695; 3.26551;
Sequence; Elk-1 [T00250]; 248; 252; 6.081668; TGTCC; 2.87695; 2.78753;
Sequence; Elk-1 [T00250]; 251; 255; 5.092035; CCTCC; 1.43848; 1.76187;
Sequence; Elk-1 [T00250]; 258; 262; 7.406067; GCTCC; 1.43848; 1.75385;
Sequence; Elk-1 [T00250]; 285; 289; 7.406067; GGAGC; 1.43848; 1.75385;
Sequence; Elk-1 [T00250]; 368; 372; 7.406067; GGAGC; 1.43848; 1.75385;
Sequence; Elk-1 [T00250]; 371; 375; 7.406067; GCTCC; 1.43848; 1.75385;
Sequence; Elk-1 [T00250]; 385; 389; 6.274504; GGTCC; 2.87695; 3.26211;
Sequence; Elk-1 [T00250]; 417; 421; 6.274504; GGACC; 2.87695; 3.26211;
Sequence; Elk-1 [T00250]; 424; 428; 2.314032; GTTCC; 1.43848; 1.53260;
Sequence; Elk-1 [T00250]; 432; 436; 0.000000; GGAAG; 1.43848; 1.49486;
Sequence; Elk-1 [T00250]; 495; 499; 6.081668; TGTCC; 2.87695; 2.78753;
Sequence; Elk-1 [T00250]; 519; 523; 3.960472; CATCC; 2.87695; 3.26551;
Sequence; Elk-1 [T00250]; 541; 545; 2.892540; ATTCC; 1.43848; 1.25493;
Sequence; Elk-1 [T00250]; 556; 560; 6.274504; GGACC; 2.87695; 3.26211;
Sequence; Elk-1 [T00250]; 560; 564; 5.092035; CCTCC; 1.43848; 1.76187;
Sequence; Elk-1 [T00250]; 573; 577; 2.121196; TTTCC; 1.43848; 1.33404;
Sequence; Elk-1 [T00250]; 607; 611; 7.406067; GCTCC; 1.43848; 1.75385;
Sequence; Elk-1 [T00250]; 658; 662; 0.000000; CTTCC; 1.43848; 1.49486;
Sequence; Elk-1 [T00250]; 686; 690; 7.406067; GCTCC; 1.43848; 1.75385;
Sequence; Elk-1 [T00250]; 712; 716; 0.000000; CTTCC; 1.43848; 1.49486;
Sequence; Elk-1 [T00250]; 737; 741; 6.274504; GGACC; 2.87695; 3.26211;
Sequence; Elk-1 [T00250]; 781; 785; 6.274504; GGACC; 2.87695; 3.26211;
Sequence; Elk-1 [T00250]; 799; 803; 5.092035; GGAGG; 1.43848; 1.76187;
Sequence; Elk-1 [T00250]; 806; 810; 3.960472; CATCC; 2.87695; 3.26551;
Sequence; Elk-1 [T00250]; 869; 873; 6.853012; AGTCC; 4.31543; 4.28952;
Sequence; Elk-1 [T00250]; 874; 878; 7.984576; GGAGT; 1.43848; 1.51166;
Sequence; Elk-1 [T00250]; 913; 917; 5.092035; GGAGG; 1.43848; 1.76187;
Sequence; Elk-1 [T00250]; 940; 944; 6.274504; GGATC; 2.87695; 3.26211;
Sequence; Elk-1 [T00250]; 946; 950; 7.406067; GGAGC; 1.43848; 1.75385;
Sequence; Elk-1 [T00250]; 956; 960; 6.853012; AGTCC; 4.31543; 4.28952;
Sequence; Elk-1 [T00250]; 1023; 1027; 6.853012; GGACT; 4.31543; 4.28952;
Sequence; Elk-1 [T00250]; 1063; 1067; 6.081668; GGACA; 2.87695; 2.78753;
Sequence; Elk-1 [T00250]; 1078; 1082; 6.081668; GGACA; 2.87695; 2.78753;
Sequence; Elk-1 [T00250]; 1103; 1107; 3.960472; CGTCC; 2.87695; 3.26551;
Sequence; Elk-1 [T00250]; 1131; 1135; 0.000000; CTTCC; 1.43848; 1.49486;
Sequence; Elk-1 [T00250]; 1143; 1147; 2.892540; GGAAT; 1.43848; 1.25493;
Sequence; Elk-1 [T00250]; 1151; 1155; 3.960472; GGATG; 2.87695; 3.26551;
Sequence; Elk-1 [T00250]; 1189; 1193; 2.314032; GTTCC; 1.43848; 1.53260;
Sequence; Elk-1 [T00250]; 1209; 1213; 3.960472; GGACG; 2.87695; 3.26551;
Sequence; Elk-1 [T00250]; 1231; 1235; 7.213232; GGAGA; 4.31543; 4.28952;
Sequence; Elk-1 [T00250]; 1255; 1259; 2.314032; GTTCC; 1.43848; 1.53260;
Sequence; Elk-1 [T00250]; 1261; 1265; 2.121196; GGAAA; 1.43848; 1.33404;
Sequence; Elk-1 [T00250]; 1269; 1273; 0.000000; GGAAG; 1.43848; 1.49486;
Sequence; Elk-1 [T00250]; 1279; 1283; 2.121196; GGAAA; 1.43848; 1.33404;

Sequence; Elk-1 [T00250]; 1295; 1299; 6.853012; GGACT; 4.31543; 4.28952;
Sequence; Elk-1 [T00250]; 1306; 1310; 6.081668; TATCC; 2.87695; 2.78753;
Sequence; Elk-1 [T00250]; 1345; 1349; 0.000000; GGAAG; 1.43848; 1.49486;
Sequence; Elk-1 [T00250]; 1359; 1363; 2.121196; GGAAA; 1.43848; 1.33404;
Sequence; Elk-1 [T00250]; 1366; 1370; 6.853012; AATCC; 4.31543; 4.28952;
Sequence; Elk-1 [T00250]; 1375; 1379; 6.081668; GGACA; 2.87695; 2.78753;
Sequence; Elk-1 [T00250]; 1420; 1424; 5.092035; GGAGG; 1.43848; 1.76187;
Sequence; Elk-1 [T00250]; 1423; 1427; 3.960472; GGACG; 2.87695; 3.26551;
Sequence; Elk-1 [T00250]; 1462; 1466; 6.853012; GGACT; 4.31543; 4.28952;
Sequence; WT1 I -KTS [T00900]; 8; 17; 14.354266; GGGAGGGCGG; 0.13205; 0.22678;
Sequence; WT1 -KTS [T01839]; 8; 17; 14.354266; GGGAGGGCGG; 0.13205; 0.22678;
Sequence; Sp1 [T00759]; 10; 18; 3.173774; GAGGGCGGA; 0.01124; 0.01594;
Sequence; Sp1 [T00759]; 133; 141; 11.743652; CACGGCGGA; 0.12362; 0.16144;
Sequence; Sp1 [T00759]; 740; 748; 7.457793; CCCGCCTCT; 0.02248; 0.03457;
Sequence; Sp1 [T00759]; 775; 783; 10.711423; TCAGGCGGA; 0.11800; 0.15319;
Sequence; Sp1 [T00755]; 11; 19; 1.308689; AGGGCGGAG; 0.02810; 0.04866;
Sequence; Sp1 [T00755]; 672; 680; 7.450611; GGGGCGTCA; 0.14610; 0.21262;
Sequence; Sp1 [T00755]; 1135; 1143; 7.450705; CGGGCGTGG; 0.14610; 0.21262;
Sequence; AP-2alphaA [T00035]; 16; 21; 2.003202; GGAGGC; 0.71924; 0.94539;
Sequence; AP-2alphaA [T00035]; 26; 31; 4.442019; AAAGGC; 0.71924; 0.70647;
Sequence; AP-2alphaA [T00035]; 31; 36; 3.414894; CTAGGC; 1.07886; 1.20726;
Sequence; AP-2alphaA [T00035]; 51; 56; 0.798453; GCCTGA; 0.35962; 0.39880;
Sequence; AP-2alphaA [T00035]; 92; 97; 2.003202; GGAGGC; 0.71924; 0.94539;
Sequence; AP-2alphaA [T00035]; 150; 155; 0.798453; GCCTGA; 0.35962; 0.39880;
Sequence; AP-2alphaA [T00035]; 459; 464; 3.255204; GCCTAC; 1.07886; 1.20726;
Sequence; AP-2alphaA [T00035]; 473; 478; 0.000000; GCAGGC; 0.71924; 0.95736;
Sequence; AP-2alphaA [T00035]; 567; 572; 3.440418; GCCTCT; 1.07886; 1.20726;
Sequence; AP-2alphaA [T00035]; 596; 601; 4.053657; GCCTAA; 0.71924; 0.68502;
Sequence; AP-2alphaA [T00035]; 603; 608; 4.053657; TTAGGC; 0.71924; 0.68502;
Sequence; AP-2alphaA [T00035]; 626; 631; 3.414894; CTAGGC; 1.07886; 1.20726;
Sequence; AP-2alphaA [T00035]; 743; 748; 3.440418; GCCTCT; 1.07886; 1.20726;
Sequence; AP-2alphaA [T00035]; 775; 780; 0.798453; TCAGGC; 0.35962; 0.39880;
Sequence; AP-2alphaA [T00035]; 827; 832; 2.801655; TGAGGC; 1.07886; 1.22836;
Sequence; AP-2alphaA [T00035]; 913; 918; 2.003202; GGAGGC; 0.71924; 0.94539;
Sequence; AP-2alphaA [T00035]; 1005; 1010; 3.164494; GCCTTG; 1.07886; 1.22836;
Sequence; AP-2alphaA [T00035]; 1050; 1055; 3.004803; GCCTTC; 1.07886; 1.22836;
Sequence; AP-2alphaA [T00035]; 1242; 1247; 4.442019; AAAGGC; 0.71924; 0.70647;
Sequence; AP-2alphaA [T00035]; 1453; 1458; 0.000000; GCAGGC; 0.71924; 0.95736;
Sequence; GAL4 [T00302]; 15; 22; 10.837618; CGGAGGCA; 0.26971; 0.33986;
Sequence; GAL4 [T00302]; 138; 145; 12.318609; CGGAGATA; 0.33714; 0.40458;
Sequence; GAL4 [T00302]; 249; 256; 0.000000; GTCCTCCG; 0.02248; 0.02938;
Sequence; GAL4 [T00302]; 256; 263; 9.893188; GAGCTCCG; 0.31467; 0.39131;
Sequence; GAL4 [T00302]; 780; 787; 5.712782; CGGACCAA; 0.20229; 0.22809;
Sequence; GAL4 [T00302]; 867; 874; 3.583560; GTAGTCCG; 0.11238; 0.12798;
Sequence; GAL4 [T00302]; 873; 880; 11.490106; CGGAGTCG; 0.38210; 0.44696;
Sequence; GAL4 [T00302]; 1062; 1069; 14.717376; CGGACATA; 0.44952; 0.50128;
Sequence; GAL4 [T00302]; 1129; 1136; 14.780748; AACTTCCG; 0.26971; 0.26660;
Sequence; GAL4 [T00302]; 1344; 1351; 13.183832; CGGAAGTG; 0.53943; 0.60537;
Sequence; GAL4 [T00302]; 1461; 1468; 11.155529; CGGACTGA; 0.26971; 0.33986;

Sequence; CREMtau [T01309]; 3; 9; 1.537547; GCTGAGG; 0.53943; 0.69868;
Sequence; CREMtau [T01309]; 18; 24; 8.014558; AGGCAGC; 1.61829; 1.70691;
Sequence; CREMtau [T01309]; 105; 111; 1.537547; GCTGAGG; 0.53943; 0.69868;
Sequence; CREMtau [T01309]; 167; 173; 9.552105; GCTGTCA; 1.07886; 1.17269;
Sequence; CREMtau [T01309]; 176; 182; 1.537547; GCTGGGG; 0.53943; 0.69868;
Sequence; CREMtau [T01309]; 208; 214; 8.014558; ATTCAGC; 1.61829; 1.70691;
Sequence; CREMtau [T01309]; 276; 282; 8.014558; GCTGATA; 1.61829; 1.70691;
Sequence; CREMtau [T01309]; 293; 299; 9.552105; AGACAGC; 1.07886; 1.17269;
Sequence; CREMtau [T01309]; 315; 321; 0.000000; CTTCAGC; 0.80914; 1.00807;
Sequence; CREMtau [T01309]; 320; 326; 0.000000; GCTGAAG; 0.80914; 1.00807;
Sequence; CREMtau [T01309]; 408; 414; 0.000000; CTGCAGC; 0.80914; 1.00807;
Sequence; CREMtau [T01309]; 468; 474; 8.014558; AAGCAGC; 1.61829; 1.70691;
Sequence; CREMtau [T01309]; 698; 704; 4.007279; GCTGACC; 0.80914; 0.98794;
Sequence; CREMtau [T01309]; 726; 732; 7.082373; GCACAGC; 0.08990; 0.11549;
Sequence; CREMtau [T01309]; 840; 846; 0.000000; GCTGAAG; 0.80914; 1.00807;
Sequence; CREMtau [T01309]; 1020; 1026; 4.007279; GCTGGAC; 0.80914; 0.98794;
Sequence; CREMtau [T01309]; 1033; 1039; 1.537547; CCTCAGC; 0.53943; 0.69868;
Sequence; CREMtau [T01309]; 1113; 1119; 11.089652; TCACAGC; 0.17981; 0.20320;
Sequence; CREMtau [T01309]; 1176; 1182; 8.014558; AGCCAGC; 1.61829; 1.70691;
Sequence; CREMtau [T01309]; 1216; 1222; 9.552105; TCTCAGC; 1.07886; 1.17269;
Sequence; CREMtau [T01309]; 1330; 1336; 8.014558; GCTGAAA; 1.61829; 1.70691;
Sequence; CREMtau [T01309]; 1440; 1446; 0.000000; CATCAGC; 0.80914; 1.00807;
Sequence; CREMtau [T01309]; 1455; 1461; 8.014558; AGGCAGC; 1.61829; 1.70691;
Sequence; CREMtau1 [T02108]; 3; 9; 1.537547; GCTGAGG; 0.53943; 0.69868;
Sequence; CREMtau1 [T02108]; 18; 24; 8.014558; AGGCAGC; 1.61829; 1.70691;
Sequence; CREMtau1 [T02108]; 105; 111; 1.537547; GCTGAGG; 0.53943; 0.69868;
Sequence; CREMtau1 [T02108]; 167; 173; 9.552105; GCTGTCA; 1.07886; 1.17269;
Sequence; CREMtau1 [T02108]; 176; 182; 1.537547; GCTGGGG; 0.53943; 0.69868;
Sequence; CREMtau1 [T02108]; 208; 214; 8.014558; ATTCAGC; 1.61829; 1.70691;
Sequence; CREMtau1 [T02108]; 276; 282; 8.014558; GCTGATA; 1.61829; 1.70691;
Sequence; CREMtau1 [T02108]; 293; 299; 9.552105; AGACAGC; 1.07886; 1.17269;
Sequence; CREMtau1 [T02108]; 315; 321; 0.000000; CTTCAGC; 0.80914; 1.00807;
Sequence; CREMtau1 [T02108]; 320; 326; 0.000000; GCTGAAG; 0.80914; 1.00807;
Sequence; CREMtau1 [T02108]; 408; 414; 0.000000; CTGCAGC; 0.80914; 1.00807;
Sequence; CREMtau1 [T02108]; 468; 474; 8.014558; AAGCAGC; 1.61829; 1.70691;
Sequence; CREMtau1 [T02108]; 698; 704; 4.007279; GCTGACC; 0.80914; 0.98794;
Sequence; CREMtau1 [T02108]; 726; 732; 7.082373; GCACAGC; 0.08990; 0.11549;
Sequence; CREMtau1 [T02108]; 840; 846; 0.000000; GCTGAAG; 0.80914; 1.00807;
Sequence; CREMtau1 [T02108]; 1020; 1026; 4.007279; GCTGGAC; 0.80914; 0.98794;
Sequence; CREMtau1 [T02108]; 1033; 1039; 1.537547; CCTCAGC; 0.53943; 0.69868;
Sequence; CREMtau1 [T02108]; 1113; 1119; 11.089652; TCACAGC; 0.17981; 0.20320;
Sequence; CREMtau1 [T02108]; 1176; 1182; 8.014558; AGCCAGC; 1.61829; 1.70691;
Sequence; CREMtau1 [T02108]; 1216; 1222; 9.552105; TCTCAGC; 1.07886; 1.17269;
Sequence; CREMtau1 [T02108]; 1330; 1336; 8.014558; GCTGAAA; 1.61829; 1.70691;
Sequence; CREMtau1 [T02108]; 1440; 1446; 0.000000; CATCAGC; 0.80914; 1.00807;
Sequence; CREMtau1 [T02108]; 1455; 1461; 8.014558; AGGCAGC; 1.61829; 1.70691;
Sequence; CREMtau2 [T02109]; 3; 9; 1.537547; GCTGAGG; 0.53943; 0.69868;
Sequence; CREMtau2 [T02109]; 18; 24; 8.014558; AGGCAGC; 1.61829; 1.70691;
Sequence; CREMtau2 [T02109]; 105; 111; 1.537547; GCTGAGG; 0.53943; 0.69868;

Sequence; CREMtau2 [T02109]; 167; 173; 9.552105; GCTGTCA; 1.07886; 1.17269;
Sequence; CREMtau2 [T02109]; 176; 182; 1.537547; GCTGGGG; 0.53943; 0.69868;
Sequence; CREMtau2 [T02109]; 208; 214; 8.014558; ATTCAGC; 1.61829; 1.70691;
Sequence; CREMtau2 [T02109]; 276; 282; 8.014558; GCTGATA; 1.61829; 1.70691;
Sequence; CREMtau2 [T02109]; 293; 299; 9.552105; AGACAGC; 1.07886; 1.17269;
Sequence; CREMtau2 [T02109]; 315; 321; 0.000000; CTTCAGC; 0.80914; 1.00807;
Sequence; CREMtau2 [T02109]; 320; 326; 0.000000; GCTGAAG; 0.80914; 1.00807;
Sequence; CREMtau2 [T02109]; 408; 414; 0.000000; CTGCAGC; 0.80914; 1.00807;
Sequence; CREMtau2 [T02109]; 468; 474; 8.014558; AAGCAGC; 1.61829; 1.70691;
Sequence; CREMtau2 [T02109]; 698; 704; 4.007279; GCTGACC; 0.80914; 0.98794;
Sequence; CREMtau2 [T02109]; 726; 732; 7.082373; GCACAGC; 0.08990; 0.11549;
Sequence; CREMtau2 [T02109]; 840; 846; 0.000000; GCTGAAG; 0.80914; 1.00807;
Sequence; CREMtau2 [T02109]; 1020; 1026; 4.007279; GCTGGAC; 0.80914; 0.98794;
Sequence; CREMtau2 [T02109]; 1033; 1039; 1.537547; CCTCAGC; 0.53943; 0.69868;
Sequence; CREMtau2 [T02109]; 1113; 1119; 11.089652; TCACAGC; 0.17981; 0.20320;
Sequence; CREMtau2 [T02109]; 1176; 1182; 8.014558; AGCCAGC; 1.61829; 1.70691;
Sequence; CREMtau2 [T02109]; 1216; 1222; 9.552105; TCTCAGC; 1.07886; 1.17269;
Sequence; CREMtau2 [T02109]; 1330; 1336; 8.014558; GCTGAAA; 1.61829; 1.70691;
Sequence; CREMtau2 [T02109]; 1440; 1446; 0.000000; CATCAGC; 0.80914; 1.00807;
Sequence; CREMtau2 [T02109]; 1455; 1461; 8.014558; AGGCAGC; 1.61829; 1.70691;
Sequence; MF3 [T00507]; 22; 26; 7.950676; AGCCA; 8.63086; 9.88779;
Sequence; MF3 [T00507]; 34; 38; 7.950676; GGCCA; 8.63086; 9.88779;
Sequence; MF3 [T00507]; 48; 52; 7.950676; TGGGC; 8.63086; 9.88779;
Sequence; MF3 [T00507]; 57; 61; 9.706275; GTCCA; 8.63086; 8.45568;
Sequence; MF3 [T00507]; 64; 68; 9.706275; ATCCA; 8.63086; 8.45568;
Sequence; MF3 [T00507]; 80; 84; 7.950676; TGGCA; 8.63086; 9.88779;
Sequence; MF3 [T00507]; 86; 90; 1.755599; CACCA; 2.87695; 3.20006;
Sequence; MF3 [T00507]; 102; 106; 7.950676; TGGGC; 8.63086; 9.88779;
Sequence; MF3 [T00507]; 130; 134; 1.755599; CACCA; 2.87695; 3.20006;
Sequence; MF3 [T00507]; 158; 162; 9.706275; TGGAA; 8.63086; 8.45568;
Sequence; MF3 [T00507]; 178; 182; 0.000000; TGGGG; 2.87695; 3.74205;
Sequence; MF3 [T00507]; 191; 195; 7.950676; TGGGA; 8.63086; 9.88779;
Sequence; MF3 [T00507]; 196; 200; 0.000000; CCCCA; 2.87695; 3.74205;
Sequence; MF3 [T00507]; 269; 273; 7.950676; TGGCA; 8.63086; 9.88779;
Sequence; MF3 [T00507]; 301; 305; 7.950676; ACCCA; 8.63086; 9.88779;
Sequence; MF3 [T00507]; 310; 314; 1.755599; CACCA; 2.87695; 3.20006;
Sequence; MF3 [T00507]; 332; 336; 7.950676; TGGGT; 8.63086; 9.88779;
Sequence; MF3 [T00507]; 362; 366; 9.706275; TACCA; 8.63086; 8.45568;
Sequence; MF3 [T00507]; 373; 377; 7.950676; TCCCA; 8.63086; 9.88779;
Sequence; MF3 [T00507]; 376; 380; 1.755599; CACCA; 2.87695; 3.20006;
Sequence; MF3 [T00507]; 379; 383; 1.755599; CACCA; 2.87695; 3.20006;
Sequence; MF3 [T00507]; 445; 449; 1.755599; CACCA; 2.87695; 3.20006;
Sequence; MF3 [T00507]; 485; 489; 7.950676; TGCCA; 8.63086; 9.88779;
Sequence; MF3 [T00507]; 543; 547; 7.950676; TCCCA; 8.63086; 9.88779;
Sequence; MF3 [T00507]; 574; 578; 9.706275; TTCCA; 8.63086; 8.45568;
Sequence; MF3 [T00507]; 608; 612; 1.755599; CTCCA; 2.87695; 3.20006;
Sequence; MF3 [T00507]; 652; 656; 7.950676; GCCCA; 8.63086; 9.88779;
Sequence; MF3 [T00507]; 671; 675; 0.000000; TGGGG; 2.87695; 3.74205;
Sequence; MF3 [T00507]; 687; 691; 1.755599; CTCCA; 2.87695; 3.20006;

Sequence; MF3 [T00507]; 715; 719; 0.000000; CCCCA; 2.87695; 3.74205;
Sequence; MF3 [T00507]; 730; 734; 7.950676; AGCCA; 8.63086; 9.88779;
Sequence; MF3 [T00507]; 755; 759; 7.950676; ACCCA; 8.63086; 9.88779;
Sequence; MF3 [T00507]; 760; 764; 0.000000; CCCCA; 2.87695; 3.74205;
Sequence; MF3 [T00507]; 782; 786; 9.706275; GACCA; 8.63086; 8.45568;
Sequence; MF3 [T00507]; 809; 813; 0.000000; CCCCA; 2.87695; 3.74205;
Sequence; MF3 [T00507]; 813; 817; 7.950676; ACCCA; 8.63086; 9.88779;
Sequence; MF3 [T00507]; 830; 834; 7.950676; GGCCA; 8.63086; 9.88779;
Sequence; MF3 [T00507]; 851; 855; 7.950676; TGCCA; 8.63086; 9.88779;
Sequence; MF3 [T00507]; 958; 962; 7.950676; TCCCA; 8.63086; 9.88779;
Sequence; MF3 [T00507]; 965; 969; 7.950676; TGGGC; 8.63086; 9.88779;
Sequence; MF3 [T00507]; 967; 971; 7.950676; GGCCA; 8.63086; 9.88779;
Sequence; MF3 [T00507]; 976; 980; 7.950676; AGCCA; 8.63086; 9.88779;
Sequence; MF3 [T00507]; 983; 987; 7.950676; ACCCA; 8.63086; 9.88779;
Sequence; MF3 [T00507]; 989; 993; 7.950676; GGCCA; 8.63086; 9.88779;
Sequence; MF3 [T00507]; 995; 999; 9.706275; AACCA; 8.63086; 8.45568;
Sequence; MF3 [T00507]; 1009; 1013; 7.950676; TGCCA; 8.63086; 9.88779;
Sequence; MF3 [T00507]; 1022; 1026; 9.706275; TGGAC; 8.63086; 8.45568;
Sequence; MF3 [T00507]; 1047; 1051; 7.950676; TGGGC; 8.63086; 9.88779;
Sequence; MF3 [T00507]; 1057; 1061; 7.950676; TGGGT; 8.63086; 9.88779;
Sequence; MF3 [T00507]; 1077; 1081; 9.706275; TGGAC; 8.63086; 8.45568;
Sequence; MF3 [T00507]; 1095; 1099; 9.706275; GACCA; 8.63086; 8.45568;
Sequence; MF3 [T00507]; 1141; 1145; 7.950676; TGGGA; 8.63086; 9.88779;
Sequence; MF3 [T00507]; 1154; 1158; 7.950676; TGGCT; 8.63086; 9.88779;
Sequence; MF3 [T00507]; 1169; 1173; 1.755599; CACCA; 2.87695; 3.20006;
Sequence; MF3 [T00507]; 1176; 1180; 7.950676; AGCCA; 8.63086; 9.88779;
Sequence; MF3 [T00507]; 1191; 1195; 7.950676; TCCCA; 8.63086; 9.88779;
Sequence; MF3 [T00507]; 1220; 1224; 7.950676; AGCCA; 8.63086; 9.88779;
Sequence; MF3 [T00507]; 1245; 1249; 7.950676; GGCCA; 8.63086; 9.88779;
Sequence; MF3 [T00507]; 1260; 1264; 9.706275; TGGAA; 8.63086; 8.45568;
Sequence; MF3 [T00507]; 1309; 1313; 0.000000; CCCCA; 2.87695; 3.74205;
Sequence; MF3 [T00507]; 1371; 1375; 0.000000; TGGGG; 2.87695; 3.74205;
Sequence; MF3 [T00507]; 1418; 1422; 7.950676; TGGGA; 8.63086; 9.88779;
Sequence; MF3 [T00507]; 1429; 1433; 9.706275; TGGTC; 8.63086; 8.45568;
Sequence; NF-1 [T00535]; 19; 27; 10.810319; GGCAGCCAA; 0.05057; 0.06431;
Sequence; NF-1 [T00535]; 268; 276; 1.188899; TTGGCAAAG; 0.01686; 0.01424;
Sequence; NF-1 [T00535]; 482; 490; 4.324828; TGATGCCAA; 0.05619; 0.05502;
Sequence; NF-1 [T00535]; 848; 856; 3.696156; TCATGCCAA; 0.06181; 0.06085;
Sequence; NF-1 [T00535]; 1242; 1250; 10.084131; AAAGGCCAA; 0.06743; 0.08472;
Sequence; NF-1 [T00536]; 20; 27; 5.237510; GCAGCCAA; 0.13486; 0.15168;
Sequence; NF-1 [T00536]; 62; 69; 12.386495; GGATCCAA; 0.67429; 0.74789;
Sequence; NF-1 [T00536]; 268; 275; 13.542995; TTGGCAAA; 0.67429; 0.63957;
Sequence; NF-1 [T00536]; 299; 306; 4.730993; CAACCCAA; 0.08990; 0.10732;
Sequence; NF-1 [T00536]; 483; 490; 13.542995; GATGCCAA; 0.67429; 0.63957;
Sequence; NF-1 [T00536]; 650; 657; 13.542995; AAGCCCAA; 0.67429; 0.63957;
Sequence; NF-1 [T00536]; 670; 677; 10.723477; TTGGGGCG; 0.17981; 0.23344;
Sequence; NF-1 [T00536]; 780; 787; 10.723477; CGGACCAA; 0.17981; 0.23344;
Sequence; NF-1 [T00536]; 849; 856; 8.305486; CATGCCAA; 0.22476; 0.24525;
Sequence; NF-1 [T00536]; 993; 1000; 12.386495; ACAACCAA; 0.67429; 0.74789;

Sequence; NF-1 [T00536]; 1046; 1053; 12.386495; TTGGGCCT; 0.67429; 0.74789;
Sequence; NF-1 [T00536]; 1243; 1250; 9.968503; AAGGCCAA; 0.26971; 0.27939;
Sequence; NF-1 [T00536]; 1417; 1424; 10.723477; TTGGGAGG; 0.17981; 0.23344;
Sequence; NF-1 [T00538]; 20; 27; 11.374018; GCAGCCAA; 0.80914; 0.89465;
Sequence; NF-1 [T00538]; 62; 69; 5.687009; GGATCCAA; 0.53943; 0.54131;
Sequence; NF-1 [T00538]; 268; 275; 12.629773; TTGGCAAA; 1.61829; 1.81125;
Sequence; NF-1 [T00538]; 299; 306; 5.687009; CAACCCAA; 0.53943; 0.54131;
Sequence; NF-1 [T00538]; 360; 367; 6.942764; TATACCAA; 1.07886; 1.09590;
Sequence; NF-1 [T00538]; 377; 384; 13.885529; ACCACCAA; 0.80914; 0.89465;
Sequence; NF-1 [T00538]; 443; 450; 8.198520; AACACCAA; 0.53943; 0.54131;
Sequence; NF-1 [T00538]; 483; 490; 5.687009; GATGCCAA; 0.53943; 0.54131;
Sequence; NF-1 [T00538]; 650; 657; 8.198520; AAGCCCAA; 0.53943; 0.54131;
Sequence; NF-1 [T00538]; 670; 677; 12.629773; TTGGGGCG; 1.61829; 1.81125;
Sequence; NF-1 [T00538]; 780; 787; 12.629773; CGGACCAA; 1.61829; 1.81125;
Sequence; NF-1 [T00538]; 849; 856; 5.687009; CATGCCAA; 0.53943; 0.54131;
Sequence; NF-1 [T00538]; 993; 1000; 12.629773; ACAACCAA; 1.61829; 1.81125;
Sequence; NF-1 [T00538]; 1046; 1053; 13.885529; TTGGGCCT; 0.80914; 0.89465;
Sequence; NF-1 [T00538]; 1189; 1196; 11.374018; GTTCCCAA; 0.80914; 0.89465;
Sequence; NF-1 [T00538]; 1243; 1250; 8.198520; AAGGCCAA; 0.53943; 0.54131;
Sequence; NF-1 [T00538]; 1307; 1314; 13.885529; ATCCCCAA; 0.80914; 0.89465;
Sequence; NF-1 [T00538]; 1417; 1424; 11.374018; TTGGGAGG; 0.80914; 0.89465;
Sequence; TGGCA-binding protein [T00832]; 21; 27; 7.256130; CAGCCAA; 1.07886; 1.32029;
Sequence; TGGCA-binding protein [T00832]; 63; 69; 5.804904; GATCCAA; 1.61829; 1.71645;
Sequence; TGGCA-binding protein [T00832]; 268; 274; 1.451226; TTGGCAA; 0.26971; 0.22154;
Sequence; TGGCA-binding protein [T00832]; 300; 306; 7.256130; AACCCAA; 1.07886; 1.32029;
Sequence; TGGCA-binding protein [T00832]; 361; 367; 4.353678; ATACCAA; 1.16876; 1.15944;
Sequence; TGGCA-binding protein [T00832]; 378; 384; 8.707355; CCACCAA; 0.71924; 0.90759;
Sequence; TGGCA-binding protein [T00832]; 444; 450; 7.256130; ACACCAA; 1.07886; 1.32029;
Sequence; TGGCA-binding protein [T00832]; 484; 490; 2.902452; ATGCCAA; 0.80914; 0.70424;
Sequence; TGGCA-binding protein [T00832]; 651; 657; 5.804904; AGCCCAA; 1.61829; 1.71645;
Sequence; TGGCA-binding protein [T00832]; 670; 676; 8.707355; TTGGGGC; 0.71924; 0.90759;
Sequence; TGGCA-binding protein [T00832]; 781; 787; 7.256130; GGACCAA; 1.07886; 1.32029;
Sequence; TGGCA-binding protein [T00832]; 850; 856; 2.902452; ATGCCAA; 0.80914; 0.70424;
Sequence; TGGCA-binding protein [T00832]; 994; 1000; 8.707355; CAACCAA; 0.71924; 0.90759;
Sequence; TGGCA-binding protein [T00832]; 1046; 1052; 7.256130; TTGGGCC; 1.07886; 1.32029;
Sequence; TGGCA-binding protein [T00832]; 1190; 1196; 2.902452; TTCCCAA; 0.80914; 0.70424;
Sequence; TGGCA-binding protein [T00832]; 1244; 1250; 4.353678; AGGCCAA; 1.16876; 1.15944;
Sequence; TGGCA-binding protein [T00832]; 1308; 1314; 5.804904; TCCCCAA; 1.61829; 1.71645;
Sequence; TGGCA-binding protein [T00832]; 1417; 1423; 5.804904; TTGGGAG; 1.61829; 1.71645;
Sequence; LIM1 [T04817]; 24; 27; 0.000000; CCAA; 5.75391; 6.09449;
Sequence; LIM1 [T04817]; 66; 69; 0.000000; CCAA; 5.75391; 6.09449;
Sequence; LIM1 [T04817]; 268; 271; 0.000000; TTGG; 5.75391; 6.09449;
Sequence; LIM1 [T04817]; 303; 306; 0.000000; CCAA; 5.75391; 6.09449;
Sequence; LIM1 [T04817]; 364; 367; 0.000000; CCAA; 5.75391; 6.09449;
Sequence; LIM1 [T04817]; 381; 384; 0.000000; CCAA; 5.75391; 6.09449;
Sequence; LIM1 [T04817]; 447; 450; 0.000000; CCAA; 5.75391; 6.09449;
Sequence; LIM1 [T04817]; 487; 490; 0.000000; CCAA; 5.75391; 6.09449;
Sequence; LIM1 [T04817]; 654; 657; 0.000000; CCAA; 5.75391; 6.09449;
Sequence; LIM1 [T04817]; 670; 673; 0.000000; TTGG; 5.75391; 6.09449;

Sequence; LIM1 [T04817]; 784; 787; 0.000000; CCAA; 5.75391; 6.09449;
Sequence; LIM1 [T04817]; 853; 856; 0.000000; CCAA; 5.75391; 6.09449;
Sequence; LIM1 [T04817]; 997; 1000; 0.000000; CCAA; 5.75391; 6.09449;
Sequence; LIM1 [T04817]; 1046; 1049; 0.000000; TTGG; 5.75391; 6.09449;
Sequence; LIM1 [T04817]; 1193; 1196; 0.000000; CCAA; 5.75391; 6.09449;
Sequence; LIM1 [T04817]; 1247; 1250; 0.000000; CCAA; 5.75391; 6.09449;
Sequence; LIM1 [T04817]; 1311; 1314; 0.000000; CCAA; 5.75391; 6.09449;
Sequence; LIM1 [T04817]; 1417; 1420; 0.000000; TTGG; 5.75391; 6.09449;
Sequence; C/EBPalpha [T00107]; 25; 27; 0.000000; CAA; 23.01562; 22.07633;
Sequence; C/EBPalpha [T00107]; 67; 69; 0.000000; CAA; 23.01562; 22.07633;
Sequence; C/EBPalpha [T00107]; 268; 270; 0.000000; TTG; 23.01562; 22.07633;
Sequence; C/EBPalpha [T00107]; 272; 274; 0.000000; CAA; 23.01562; 22.07633;
Sequence; C/EBPalpha [T00107]; 299; 301; 0.000000; CAA; 23.01562; 22.07633;
Sequence; C/EBPalpha [T00107]; 304; 306; 0.000000; CAA; 23.01562; 22.07633;
Sequence; C/EBPalpha [T00107]; 356; 358; 0.000000; CAA; 23.01562; 22.07633;
Sequence; C/EBPalpha [T00107]; 365; 367; 0.000000; CAA; 23.01562; 22.07633;
Sequence; C/EBPalpha [T00107]; 382; 384; 0.000000; CAA; 23.01562; 22.07633;
Sequence; C/EBPalpha [T00107]; 422; 424; 0.000000; TTG; 23.01562; 22.07633;
Sequence; C/EBPalpha [T00107]; 442; 444; 0.000000; CAA; 23.01562; 22.07633;
Sequence; C/EBPalpha [T00107]; 448; 450; 0.000000; CAA; 23.01562; 22.07633;
Sequence; C/EBPalpha [T00107]; 488; 490; 0.000000; CAA; 23.01562; 22.07633;
Sequence; C/EBPalpha [T00107]; 579; 581; 0.000000; CAA; 23.01562; 22.07633;
Sequence; C/EBPalpha [T00107]; 594; 596; 0.000000; TTG; 23.01562; 22.07633;
Sequence; C/EBPalpha [T00107]; 638; 640; 0.000000; CAA; 23.01562; 22.07633;
Sequence; C/EBPalpha [T00107]; 643; 645; 0.000000; CAA; 23.01562; 22.07633;
Sequence; C/EBPalpha [T00107]; 649; 651; 0.000000; CAA; 23.01562; 22.07633;
Sequence; C/EBPalpha [T00107]; 655; 657; 0.000000; CAA; 23.01562; 22.07633;
Sequence; C/EBPalpha [T00107]; 670; 672; 0.000000; TTG; 23.01562; 22.07633;
Sequence; C/EBPalpha [T00107]; 785; 787; 0.000000; CAA; 23.01562; 22.07633;
Sequence; C/EBPalpha [T00107]; 854; 856; 0.000000; CAA; 23.01562; 22.07633;
Sequence; C/EBPalpha [T00107]; 898; 900; 0.000000; TTG; 23.01562; 22.07633;
Sequence; C/EBPalpha [T00107]; 937; 939; 0.000000; CAA; 23.01562; 22.07633;
Sequence; C/EBPalpha [T00107]; 954; 956; 0.000000; CAA; 23.01562; 22.07633;
Sequence; C/EBPalpha [T00107]; 994; 996; 0.000000; CAA; 23.01562; 22.07633;
Sequence; C/EBPalpha [T00107]; 998; 1000; 0.000000; CAA; 23.01562; 22.07633;
Sequence; C/EBPalpha [T00107]; 1008; 1010; 0.000000; TTG; 23.01562; 22.07633;
Sequence; C/EBPalpha [T00107]; 1018; 1020; 0.000000; TTG; 23.01562; 22.07633;
Sequence; C/EBPalpha [T00107]; 1043; 1045; 0.000000; TTG; 23.01562; 22.07633;
Sequence; C/EBPalpha [T00107]; 1046; 1048; 0.000000; TTG; 23.01562; 22.07633;
Sequence; C/EBPalpha [T00107]; 1070; 1072; 0.000000; TTG; 23.01562; 22.07633;
Sequence; C/EBPalpha [T00107]; 1086; 1088; 0.000000; CAA; 23.01562; 22.07633;
Sequence; C/EBPalpha [T00107]; 1128; 1130; 0.000000; CAA; 23.01562; 22.07633;
Sequence; C/EBPalpha [T00107]; 1194; 1196; 0.000000; CAA; 23.01562; 22.07633;
Sequence; C/EBPalpha [T00107]; 1248; 1250; 0.000000; CAA; 23.01562; 22.07633;
Sequence; C/EBPalpha [T00107]; 1312; 1314; 0.000000; CAA; 23.01562; 22.07633;
Sequence; C/EBPalpha [T00107]; 1339; 1341; 0.000000; TTG; 23.01562; 22.07633;
Sequence; C/EBPalpha [T00107]; 1352; 1354; 0.000000; CAA; 23.01562; 22.07633;
Sequence; C/EBPalpha [T00107]; 1378; 1380; 0.000000; CAA; 23.01562; 22.07633;
Sequence; C/EBPalpha [T00107]; 1400; 1402; 0.000000; CAA; 23.01562; 22.07633;

Sequence; C/EBPalpha [T00107]; 1409; 1411; 0.000000; TTG; 23.01562; 22.07633;
Sequence; C/EBPalpha [T00107]; 1417; 1419; 0.000000; TTG; 23.01562; 22.07633;
Sequence; C/EBPalpha [T00107]; 1435; 1437; 0.000000; CAA; 23.01562; 22.07633;
Sequence; TCF-1A [T00999]; 21; 29; 13.648823; CAGCCAAAG; 0.20229; 0.22678;
Sequence; TCF-1A [T00999]; 268; 276; 13.648823; TTGGCAAAG; 0.20229; 0.22678;
Sequence; TCF-1A [T00999]; 994; 1002; 7.421728; CAACCAAAG; 0.25286; 0.24416;
Sequence; TCF-1A [T00999]; 1041; 1049; 1.194633; CTTTGTTGG; 0.05057; 0.04828;
Sequence; TCF-1A [T00999]; 1348; 1356; 9.340643; AGTGCAAAG; 0.02248; 0.02051;
Sequence; TCF-1A [T00999]; 1407; 1415; 7.421728; CTTTGAAAG; 0.25286; 0.24416;
Sequence; LEF-1 [T02905]; 22; 29; 9.741481; AGCCAAAG; 0.13486; 0.14612;
Sequence; LEF-1 [T02905]; 269; 276; 9.555277; TGGCAAAG; 0.13486; 0.14612;
Sequence; LEF-1 [T02905]; 995; 1002; 2.021471; AACCAAAG; 0.13486; 0.13865;
Sequence; LEF-1 [T02905]; 1041; 1048; 0.904249; CTTTGTTG; 0.06743; 0.06486;
Sequence; LEF-1 [T02905]; 1349; 1356; 7.479433; GTGCAAAG; 0.15733; 0.14981;
Sequence; LEF-1 [T02905]; 1407; 1414; 6.707850; CTTTGAAA; 0.08990; 0.06725;
Sequence; TCF-4E [T02878]; 23; 29; 12.604771; GCCAAAG; 0.35962; 0.39741;
Sequence; TCF-4E [T02878]; 270; 276; 6.302385; GGCAAAG; 0.44952; 0.44839;
Sequence; TCF-4E [T02878]; 996; 1002; 6.302385; ACCAAAG; 0.44952; 0.44839;
Sequence; TCF-4E [T02878]; 1041; 1047; 6.302385; CTTTGTT; 0.44952; 0.44839;
Sequence; TCF-4E [T02878]; 1350; 1356; 3.151193; TGCAAAG; 0.17981; 0.15204;
Sequence; TCF-4E [T02878]; 1407; 1413; 6.302385; CTTTGAA; 0.44952; 0.44839;
Sequence; HNF-3 [T02277]; 25; 30; 14.269340; CAAAGG; 4.31543; 3.77291;
Sequence; HNF-3 [T02277]; 161; 166; 9.512894; AAAAGA; 5.39429; 4.39500;
Sequence; HNF-3 [T02277]; 272; 277; 14.269340; CAAAGC; 4.31543; 3.77291;
Sequence; HNF-3 [T02277]; 281; 286; 9.512894; TAAAGG; 5.39429; 4.39500;
Sequence; HNF-3 [T02277]; 570; 575; 9.512894; TCTTTT; 5.39429; 4.39500;
Sequence; HNF-3 [T02277]; 667; 672; 14.269340; CGTTTG; 4.31543; 3.77291;
Sequence; HNF-3 [T02277]; 746; 751; 9.512894; TCTTTC; 5.39429; 4.39500;
Sequence; HNF-3 [T02277]; 771; 776; 14.269340; AGTTTC; 4.31543; 3.77291;
Sequence; HNF-3 [T02277]; 888; 893; 9.512894; TAAAGC; 5.39429; 4.39500;
Sequence; HNF-3 [T02277]; 998; 1003; 4.756447; CAAAGA; 2.15771; 1.69705;
Sequence; HNF-3 [T02277]; 1040; 1045; 9.512894; ACTTTG; 5.39429; 4.39500;
Sequence; HNF-3 [T02277]; 1086; 1091; 9.512894; CAAACT; 5.39429; 4.39500;
Sequence; HNF-3 [T02277]; 1109; 1114; 9.512894; TCTTTC; 5.39429; 4.39500;
Sequence; HNF-3 [T02277]; 1157; 1162; 11.337888; CTTTTA; 1.79810; 1.44073;
Sequence; HNF-3 [T02277]; 1225; 1230; 11.337888; GAAAAA; 1.79810; 1.44073;
Sequence; HNF-3 [T02277]; 1249; 1254; 9.512894; AAAACA; 5.39429; 4.39500;
Sequence; HNF-3 [T02277]; 1262; 1267; 14.269340; GAAAGT; 4.31543; 3.77291;
Sequence; HNF-3 [T02277]; 1280; 1285; 11.337888; GAAAAA; 1.79810; 1.44073;
Sequence; HNF-3 [T02277]; 1281; 1286; 11.337888; AAAAAA; 1.79810; 1.44073;
Sequence; HNF-3 [T02277]; 1282; 1287; 11.337888; AAAAAA; 1.79810; 1.44073;
Sequence; HNF-3 [T02277]; 1283; 1288; 11.337888; AAAAAA; 1.79810; 1.44073;
Sequence; HNF-3 [T02277]; 1285; 1290; 14.269340; AAAACT; 4.31543; 3.77291;
Sequence; HNF-3 [T02277]; 1297; 1302; 14.269340; ACTTTT; 4.31543; 3.77291;
Sequence; HNF-3 [T02277]; 1313; 1318; 14.269340; AAAACT; 4.31543; 3.77291;
Sequence; HNF-3 [T02277]; 1333; 1338; 14.269340; GAAATT; 4.31543; 3.77291;
Sequence; HNF-3 [T02277]; 1335; 1340; 14.269340; AATTTT; 4.31543; 3.77291;
Sequence; HNF-3 [T02277]; 1336; 1341; 11.337888; ATTTTG; 1.79810; 1.44073;
Sequence; HNF-3 [T02277]; 1352; 1357; 14.269340; CAAAGC; 4.31543; 3.77291;

Sequence; HNF-3 [T02277]; 1360; 1365; 9.512894; GAAATA; 5.39429; 4.39500;
Sequence; HNF-3 [T02277]; 1364; 1369; 9.512894; TAAATC; 5.39429; 4.39500;
Sequence; HNF-3 [T02277]; 1406; 1411; 4.756447; TCTTTG; 2.15771; 1.69705;
Sequence; HNF-3 [T02277]; 1449; 1454; 9.512894; TAAAGC; 5.39429; 4.39500;
Sequence; NFI/CTF [T00094]; 24; 31; 3.793671; CCAAAGGC; 0.13486; 0.15049;
Sequence; NFI/CTF [T00094]; 66; 73; 7.014249; CCAACCCT; 0.53943; 0.62847;
Sequence; NFI/CTF [T00094]; 303; 310; 5.558661; CCAAGCAC; 0.40457; 0.44188;
Sequence; NFI/CTF [T00094]; 364; 371; 10.579747; CCAAGGAG; 0.49448; 0.53515;
Sequence; NFI/CTF [T00094]; 381; 388; 9.352332; CCAAGGTC; 0.40457; 0.41966;
Sequence; NFI/CTF [T00094]; 447; 454; 7.014249; CCAACCCT; 0.53943; 0.62847;
Sequence; NFI/CTF [T00094]; 487; 494; 10.579747; CCAATGTG; 0.49448; 0.53515;
Sequence; NFI/CTF [T00094]; 654; 661; 14.601592; CCAACTTC; 1.07886; 1.04347;
Sequence; NFI/CTF [T00094]; 666; 673; 0.000000; ACGTTTGG; 0.13486; 0.15684;
Sequence; NFI/CTF [T00094]; 784; 791; 10.270346; CCAACAGA; 0.49448; 0.53515;
Sequence; NFI/CTF [T00094]; 853; 860; 14.373419; CCAAGAAG; 1.07886; 1.04347;
Sequence; NFI/CTF [T00094]; 997; 1004; 9.352332; CCAAAGAC; 0.40457; 0.41966;
Sequence; NFI/CTF [T00094]; 1193; 1200; 7.587343; CCAATAGC; 0.26971; 0.26282;
Sequence; NFI/CTF [T00094]; 1247; 1254; 14.373419; CCAAAACA; 1.07886; 1.04347;
Sequence; NFI/CTF [T00094]; 1311; 1318; 13.146004; CCAAAACT; 0.80914; 0.73675;
Sequence; NFI/CTF [T00094]; 1413; 1420; 5.558661; AAGCTTGG; 0.40457; 0.44188;
Sequence; NF-AT4 [T01949]; 24; 31; 11.261088; CCAAAGGC; 1.12381; 0.86060;
Sequence; DEF:GLO:SQUA [T03217]; 26; 32; 1.302806; AAAGGCT; 0.71924; 0.66295;
Sequence; DEF:GLO:SQUA [T03217]; 162; 168; 10.658991; AAAGAGC; 0.71924; 0.77034;
Sequence; DEF:GLO:SQUA [T03217]; 273; 279; 9.356185; AAAGCTG; 1.43848; 1.31754;
Sequence; DEF:GLO:SQUA [T03217]; 282; 288; 1.302806; AAAGGAG; 0.71924; 0.66295;
Sequence; DEF:GLO:SQUA [T03217]; 327; 333; 1.302806; AAAGGTG; 0.71924; 0.66295;
Sequence; DEF:GLO:SQUA [T03217]; 568; 574; 10.658991; CCTCTTT; 0.71924; 0.77034;
Sequence; DEF:GLO:SQUA [T03217]; 744; 750; 10.658991; CCTCTTT; 0.71924; 0.77034;
Sequence; DEF:GLO:SQUA [T03217]; 889; 895; 10.658991; AAAGCCG; 0.71924; 0.77034;
Sequence; DEF:GLO:SQUA [T03217]; 999; 1005; 10.658991; AAAGACG; 0.71924; 0.77034;
Sequence; DEF:GLO:SQUA [T03217]; 1038; 1044; 6.632302; GCACTTT; 0.35962; 0.31863;
Sequence; DEF:GLO:SQUA [T03217]; 1107; 1113; 9.356185; CTTCTTT; 1.43848; 1.31754;
Sequence; DEF:GLO:SQUA [T03217]; 1154; 1160; 9.356185; TGGCTTT; 1.43848; 1.31754;
Sequence; DEF:GLO:SQUA [T03217]; 1228; 1234; 1.302806; AAAGGAG; 0.71924; 0.66295;
Sequence; DEF:GLO:SQUA [T03217]; 1242; 1248; 2.605613; AAAGGCC; 0.35962; 0.38762;
Sequence; DEF:GLO:SQUA [T03217]; 1263; 1269; 5.329495; AAAGTAG; 0.71924; 0.54497;
Sequence; DEF:GLO:SQUA [T03217]; 1292; 1298; 1.302806; AAAGGAC; 0.71924; 0.66295;
Sequence; DEF:GLO:SQUA [T03217]; 1295; 1301; 6.632302; GGACTTT; 0.35962; 0.31863;
Sequence; DEF:GLO:SQUA [T03217]; 1353; 1359; 9.356185; AAAGCAG; 1.43848; 1.31754;
Sequence; DEF:GLO:SQUA [T03217]; 1404; 1410; 9.356185; TCTCTTT; 1.43848; 1.31754;
Sequence; DEF:GLO:SQUA [T03217]; 1412; 1418; 8.053378; AAAGCTT; 0.71924; 0.56335;
Sequence; DEF:GLO:SQUA [T03217]; 1450; 1456; 9.356185; AAAGCAG; 1.43848; 1.31754;
Sequence; SF-1 [T01147]; 31; 39; 14.895576; CTAGGCCAG; 0.40457; 0.44865;
Sequence; SF-1 [T01147]; 416; 424; 14.895576; CGGACCTTG; 0.40457; 0.44865;
Sequence; SF-1 [T01147]; 649; 657; 14.895576; CAAGCCCAA; 0.40457; 0.44865;
Sequence; SF-1 [T01147]; 1242; 1250; 14.895576; AAAGGCCAA; 0.40457; 0.44865;
Sequence; NF-1 [T01298]; 34; 39; 1.293177; GGCCAG; 2.15771; 2.58621;
Sequence; NF-1 [T01298]; 57; 62; 2.586354; GTCCAG; 0.35962; 0.39880;
Sequence; NF-1 [T01298]; 86; 91; 1.293177; CACCAG; 2.15771; 2.58621;

Sequence; NF-1 [T01298]; 177; 182; 1.293177; CTGGGG; 2.15771; 2.58621;
Sequence; NF-1 [T01298]; 730; 735; 0.646588; AGCCAG; 1.43848; 1.65196;
Sequence; NF-1 [T01298]; 760; 765; 1.293177; CCCCAG; 2.15771; 2.58621;
Sequence; NF-1 [T01298]; 964; 969; 1.939765; CTGGGC; 1.43848; 1.65196;
Sequence; NF-1 [T01298]; 1021; 1026; 2.586354; CTGGAC; 0.35962; 0.39880;
Sequence; NF-1 [T01298]; 1169; 1174; 1.293177; CACCAG; 2.15771; 2.58621;
Sequence; NF-1 [T01298]; 1176; 1181; 0.646588; AGCCAG; 1.43848; 1.65196;
Sequence; NF-1 [T01298]; 1220; 1225; 0.646588; AGCCAG; 1.43848; 1.65196;
Sequence; NF-1 [T01298]; 1259; 1264; 1.293177; CTGGAA; 2.15771; 2.58621;
Sequence; NF-1 [T01298]; 1370; 1375; 1.293177; CTGGGG; 2.15771; 2.58621;
Sequence; GAGA factor [T00301]; 38; 43; 0.000000; AGAGAG; 0.35962; 0.35153;
Sequence; GAGA factor [T00301]; 145; 150; 0.000000; AGAGAG; 0.35962; 0.35153;
Sequence; GAGA factor [T00301]; 505; 510; 1.292109; CTCTCA; 0.71924; 0.73710;
Sequence; GAGA factor [T00301]; 523; 528; 1.292109; CTCTCA; 0.71924; 0.73710;
Sequence; GAGA factor [T00301]; 1203; 1208; 1.292109; TGAGAG; 0.71924; 0.73710;
Sequence; GAGA factor [T00301]; 1215; 1220; 1.292109; CTCTCA; 0.71924; 0.73710;
Sequence; GA-BF [T00297]; 38; 44; 8.014558; AGAGAGA; 1.61829; 1.35490;
Sequence; GA-BF [T00297]; 141; 147; 8.014558; AGATAGA; 1.61829; 1.35490;
Sequence; GA-BF [T00297]; 155; 161; 9.552105; AGATGGA; 1.07886; 0.92204;
Sequence; GA-BF [T00297]; 746; 752; 0.000000; TCTTTCT; 0.80914; 0.69078;
Sequence; GA-BF [T00297]; 859; 865; 8.014558; AGATTCA; 1.61829; 1.35490;
Sequence; GA-BF [T00297]; 1105; 1111; 1.537547; TCCTTCT; 0.53943; 0.47473;
Sequence; GA-BF [T00297]; 1124; 1130; 9.552105; AGAGCAA; 1.07886; 0.92204;
Sequence; GA-BF [T00297]; 1162; 1168; 9.552105; AGAGTAA; 1.07886; 0.92204;
Sequence; GA-BF [T00297]; 1205; 1211; 9.552105; AGAGGGA; 1.07886; 0.92204;
Sequence; GA-BF [T00297]; 1224; 1230; 1.537547; AGAAAAA; 0.53943; 0.47473;
Sequence; GA-BF [T00297]; 1233; 1239; 0.000000; AGAATGA; 0.80914; 0.69078;
Sequence; PBF [T02693]; 40; 47; 6.570033; AGAGAAGG; 0.26971; 0.28537;
Sequence; PBF [T02693]; 204; 211; 3.285017; CCTTATTC; 0.13486; 0.11389;
Sequence; PBF [T02693]; 420; 427; 6.570033; CCTTGTTC; 0.26971; 0.28537;
Sequence; PBF [T02693]; 1106; 1113; 6.570033; CCTTCTTT; 0.26971; 0.28537;
Sequence; PBF [T02693]; 1225; 1232; 0.000000; GAAAAAGG; 0.13486; 0.13014;
Sequence; PBF [T02693]; 1239; 1246; 0.000000; AGAAAAGG; 0.13486; 0.13014;
Sequence; Zic1 [T04669]; 45; 48; 0.970728; AGGT; 17.26172; 18.34347;
Sequence; Zic1 [T04669]; 69; 72; 0.000000; ACCC; 5.75391; 6.94211;
Sequence; Zic1 [T04669]; 77; 80; 0.970728; AGGT; 17.26172; 18.34347;
Sequence; Zic1 [T04669]; 87; 90; 0.970728; ACCA; 17.26172; 18.34347;
Sequence; Zic1 [T04669]; 131; 134; 0.970728; ACCA; 17.26172; 18.34347;
Sequence; Zic1 [T04669]; 195; 198; 0.000000; ACCC; 5.75391; 6.94211;
Sequence; Zic1 [T04669]; 301; 304; 0.000000; ACCC; 5.75391; 6.94211;
Sequence; Zic1 [T04669]; 311; 314; 0.970728; ACCA; 17.26172; 18.34347;
Sequence; Zic1 [T04669]; 329; 332; 0.970728; AGGT; 17.26172; 18.34347;
Sequence; Zic1 [T04669]; 333; 336; 0.000000; GGGT; 5.75391; 6.94211;
Sequence; Zic1 [T04669]; 343; 346; 0.970728; ACCG; 17.26172; 18.34347;
Sequence; Zic1 [T04669]; 346; 349; 0.000000; GGGT; 5.75391; 6.94211;
Sequence; Zic1 [T04669]; 363; 366; 0.970728; ACCA; 17.26172; 18.34347;
Sequence; Zic1 [T04669]; 377; 380; 0.970728; ACCA; 17.26172; 18.34347;
Sequence; Zic1 [T04669]; 380; 383; 0.970728; ACCA; 17.26172; 18.34347;
Sequence; Zic1 [T04669]; 384; 387; 0.970728; AGGT; 17.26172; 18.34347;

Sequence; Zic1 [T04669]; 399; 402; 0.970728; AGGT; 17.26172; 18.34347;
Sequence; Zic1 [T04669]; 406; 409; 0.970728; ACCT; 17.26172; 18.34347;
Sequence; Zic1 [T04669]; 419; 422; 0.970728; ACCT; 17.26172; 18.34347;
Sequence; Zic1 [T04669]; 438; 441; 0.970728; ACCT; 17.26172; 18.34347;
Sequence; Zic1 [T04669]; 446; 449; 0.970728; ACCA; 17.26172; 18.34347;
Sequence; Zic1 [T04669]; 450; 453; 0.000000; ACCC; 5.75391; 6.94211;
Sequence; Zic1 [T04669]; 503; 506; 0.970728; ACCT; 17.26172; 18.34347;
Sequence; Zic1 [T04669]; 510; 513; 0.000000; ACCC; 5.75391; 6.94211;
Sequence; Zic1 [T04669]; 515; 518; 0.970728; ACCG; 17.26172; 18.34347;
Sequence; Zic1 [T04669]; 558; 561; 0.000000; ACCC; 5.75391; 6.94211;
Sequence; Zic1 [T04669]; 612; 615; 0.000000; ACCC; 5.75391; 6.94211;
Sequence; Zic1 [T04669]; 620; 623; 0.000000; ACCC; 5.75391; 6.94211;
Sequence; Zic1 [T04669]; 680; 683; 0.000000; ACCC; 5.75391; 6.94211;
Sequence; Zic1 [T04669]; 691; 694; 0.000000; ACCC; 5.75391; 6.94211;
Sequence; Zic1 [T04669]; 702; 705; 0.970728; ACCT; 17.26172; 18.34347;
Sequence; Zic1 [T04669]; 707; 710; 0.000000; ACCC; 5.75391; 6.94211;
Sequence; Zic1 [T04669]; 719; 722; 0.970728; ACCT; 17.26172; 18.34347;
Sequence; Zic1 [T04669]; 739; 742; 0.000000; ACCC; 5.75391; 6.94211;
Sequence; Zic1 [T04669]; 755; 758; 0.000000; ACCC; 5.75391; 6.94211;
Sequence; Zic1 [T04669]; 759; 762; 0.000000; ACCC; 5.75391; 6.94211;
Sequence; Zic1 [T04669]; 766; 769; 0.970728; ACCT; 17.26172; 18.34347;
Sequence; Zic1 [T04669]; 783; 786; 0.970728; ACCA; 17.26172; 18.34347;
Sequence; Zic1 [T04669]; 801; 804; 0.970728; AGGT; 17.26172; 18.34347;
Sequence; Zic1 [T04669]; 813; 816; 0.000000; ACCC; 5.75391; 6.94211;
Sequence; Zic1 [T04669]; 817; 820; 0.970728; ACCT; 17.26172; 18.34347;
Sequence; Zic1 [T04669]; 880; 883; 0.000000; GGGT; 5.75391; 6.94211;
Sequence; Zic1 [T04669]; 895; 898; 0.000000; GGGT; 5.75391; 6.94211;
Sequence; Zic1 [T04669]; 923; 926; 0.970728; ACCT; 17.26172; 18.34347;
Sequence; Zic1 [T04669]; 962; 965; 0.970728; ACCT; 17.26172; 18.34347;
Sequence; Zic1 [T04669]; 971; 974; 0.970728; ACCT; 17.26172; 18.34347;
Sequence; Zic1 [T04669]; 983; 986; 0.000000; ACCC; 5.75391; 6.94211;
Sequence; Zic1 [T04669]; 996; 999; 0.970728; ACCA; 17.26172; 18.34347;
Sequence; Zic1 [T04669]; 1032; 1035; 0.970728; ACCT; 17.26172; 18.34347;
Sequence; Zic1 [T04669]; 1058; 1061; 0.000000; GGGT; 5.75391; 6.94211;
Sequence; Zic1 [T04669]; 1096; 1099; 0.970728; ACCA; 17.26172; 18.34347;
Sequence; Zic1 [T04669]; 1170; 1173; 0.970728; ACCA; 17.26172; 18.34347;
Sequence; Zic1 [T04669]; 1426; 1429; 0.970728; CGGT; 17.26172; 18.34347;
Sequence; Zic1 [T04669]; 1429; 1432; 0.970728; TGGT; 17.26172; 18.34347;
Sequence; Zic2 [T04670]; 46; 48; 0.000000; GGT; 23.01562; 25.28558;
Sequence; Zic2 [T04670]; 69; 71; 0.000000; ACC; 23.01562; 25.28558;
Sequence; Zic2 [T04670]; 78; 80; 0.000000; GGT; 23.01562; 25.28558;
Sequence; Zic2 [T04670]; 87; 89; 0.000000; ACC; 23.01562; 25.28558;
Sequence; Zic2 [T04670]; 131; 133; 0.000000; ACC; 23.01562; 25.28558;
Sequence; Zic2 [T04670]; 195; 197; 0.000000; ACC; 23.01562; 25.28558;
Sequence; Zic2 [T04670]; 301; 303; 0.000000; ACC; 23.01562; 25.28558;
Sequence; Zic2 [T04670]; 311; 313; 0.000000; ACC; 23.01562; 25.28558;
Sequence; Zic2 [T04670]; 330; 332; 0.000000; GGT; 23.01562; 25.28558;
Sequence; Zic2 [T04670]; 334; 336; 0.000000; GGT; 23.01562; 25.28558;
Sequence; Zic2 [T04670]; 343; 345; 0.000000; ACC; 23.01562; 25.28558;

Sequence; Zic2 [T04670]; 347; 349; 0.000000; GGT; 23.01562; 25.28558;
Sequence; Zic2 [T04670]; 363; 365; 0.000000; ACC; 23.01562; 25.28558;
Sequence; Zic2 [T04670]; 377; 379; 0.000000; ACC; 23.01562; 25.28558;
Sequence; Zic2 [T04670]; 380; 382; 0.000000; ACC; 23.01562; 25.28558;
Sequence; Zic2 [T04670]; 385; 387; 0.000000; GGT; 23.01562; 25.28558;
Sequence; Zic2 [T04670]; 400; 402; 0.000000; GGT; 23.01562; 25.28558;
Sequence; Zic2 [T04670]; 406; 408; 0.000000; ACC; 23.01562; 25.28558;
Sequence; Zic2 [T04670]; 419; 421; 0.000000; ACC; 23.01562; 25.28558;
Sequence; Zic2 [T04670]; 438; 440; 0.000000; ACC; 23.01562; 25.28558;
Sequence; Zic2 [T04670]; 446; 448; 0.000000; ACC; 23.01562; 25.28558;
Sequence; Zic2 [T04670]; 450; 452; 0.000000; ACC; 23.01562; 25.28558;
Sequence; Zic2 [T04670]; 503; 505; 0.000000; ACC; 23.01562; 25.28558;
Sequence; Zic2 [T04670]; 510; 512; 0.000000; ACC; 23.01562; 25.28558;
Sequence; Zic2 [T04670]; 515; 517; 0.000000; ACC; 23.01562; 25.28558;
Sequence; Zic2 [T04670]; 558; 560; 0.000000; ACC; 23.01562; 25.28558;
Sequence; Zic2 [T04670]; 612; 614; 0.000000; ACC; 23.01562; 25.28558;
Sequence; Zic2 [T04670]; 620; 622; 0.000000; ACC; 23.01562; 25.28558;
Sequence; Zic2 [T04670]; 680; 682; 0.000000; ACC; 23.01562; 25.28558;
Sequence; Zic2 [T04670]; 691; 693; 0.000000; ACC; 23.01562; 25.28558;
Sequence; Zic2 [T04670]; 702; 704; 0.000000; ACC; 23.01562; 25.28558;
Sequence; Zic2 [T04670]; 707; 709; 0.000000; ACC; 23.01562; 25.28558;
Sequence; Zic2 [T04670]; 719; 721; 0.000000; ACC; 23.01562; 25.28558;
Sequence; Zic2 [T04670]; 739; 741; 0.000000; ACC; 23.01562; 25.28558;
Sequence; Zic2 [T04670]; 755; 757; 0.000000; ACC; 23.01562; 25.28558;
Sequence; Zic2 [T04670]; 759; 761; 0.000000; ACC; 23.01562; 25.28558;
Sequence; Zic2 [T04670]; 766; 768; 0.000000; ACC; 23.01562; 25.28558;
Sequence; Zic2 [T04670]; 783; 785; 0.000000; ACC; 23.01562; 25.28558;
Sequence; Zic2 [T04670]; 802; 804; 0.000000; GGT; 23.01562; 25.28558;
Sequence; Zic2 [T04670]; 813; 815; 0.000000; ACC; 23.01562; 25.28558;
Sequence; Zic2 [T04670]; 817; 819; 0.000000; ACC; 23.01562; 25.28558;
Sequence; Zic2 [T04670]; 881; 883; 0.000000; GGT; 23.01562; 25.28558;
Sequence; Zic2 [T04670]; 896; 898; 0.000000; GGT; 23.01562; 25.28558;
Sequence; Zic2 [T04670]; 923; 925; 0.000000; ACC; 23.01562; 25.28558;
Sequence; Zic2 [T04670]; 962; 964; 0.000000; ACC; 23.01562; 25.28558;
Sequence; Zic2 [T04670]; 971; 973; 0.000000; ACC; 23.01562; 25.28558;
Sequence; Zic2 [T04670]; 983; 985; 0.000000; ACC; 23.01562; 25.28558;
Sequence; Zic2 [T04670]; 996; 998; 0.000000; ACC; 23.01562; 25.28558;
Sequence; Zic2 [T04670]; 1032; 1034; 0.000000; ACC; 23.01562; 25.28558;
Sequence; Zic2 [T04670]; 1059; 1061; 0.000000; GGT; 23.01562; 25.28558;
Sequence; Zic2 [T04670]; 1096; 1098; 0.000000; ACC; 23.01562; 25.28558;
Sequence; Zic2 [T04670]; 1170; 1172; 0.000000; ACC; 23.01562; 25.28558;
Sequence; Zic2 [T04670]; 1427; 1429; 0.000000; GGT; 23.01562; 25.28558;
Sequence; Zic2 [T04670]; 1430; 1432; 0.000000; GGT; 23.01562; 25.28558;
Sequence; USF-1 [T00875]; 44; 50; 6.006507; AAGGTGG; 0.35962; 0.33755;
Sequence; USF-1 [T00875]; 76; 82; 5.152973; TAGGTGG; 0.71924; 0.77188;
Sequence; USF-1 [T00875]; 132; 138; 9.731297; CCACGGC; 0.35962; 0.47843;
Sequence; USF-1 [T00875]; 312; 318; 5.152973; CCACTTC; 0.71924; 0.77188;
Sequence; USF-1 [T00875]; 328; 334; 6.006507; AAGGTGG; 0.35962; 0.33755;
Sequence; USF-1 [T00875]; 375; 381; 11.607096; CCACCAC; 1.07886; 1.30093;

Sequence; USF-1 [T00875]; 378; 384; 11.607096; CCACCAA; 1.07886; 1.30093;
Sequence; USF-1 [T00875]; 545; 551; 0.000000; CCACATG; 0.35962; 0.40224;
Sequence; USF-1 [T00875]; 576; 582; 6.454123; CCACAAG; 0.35962; 0.44080;
Sequence; USF-1 [T00875]; 610; 616; 11.538613; CCACCCT; 1.07886; 1.30093;
Sequence; USF-1 [T00875]; 689; 695; 10.685079; CCACCCC; 1.07886; 1.40071;
Sequence; USF-1 [T00875]; 717; 723; 5.152973; CCACCTC; 0.71924; 0.77188;
Sequence; USF-1 [T00875]; 757; 763; 10.685079; CCACCCC; 1.07886; 1.40071;
Sequence; USF-1 [T00875]; 811; 817; 10.685079; CCACCCA; 1.07886; 1.40071;
Sequence; USF-1 [T00875]; 815; 821; 6.006507; CCACCTT; 0.35962; 0.33755;
Sequence; USF-1 [T00875]; 832; 838; 10.653315; CCACACA; 1.07886; 1.40071;
Sequence; USF-1 [T00875]; 960; 966; 0.031764; CCACCTG; 0.35962; 0.40224;
Sequence; USF-1 [T00875]; 969; 975; 6.006507; CCACCTT; 0.35962; 0.33755;
Sequence; USF-1 [T00875]; 985; 991; 9.731297; CCACGGC; 0.35962; 0.47843;
Sequence; USF-1 [T00875]; 991; 997; 11.575332; CCACAAC; 1.07886; 1.30093;
Sequence; USF-1 [T00875]; 1053; 1059; 11.575332; TTCGTGG; 1.07886; 1.30093;
Sequence; USF-1 [T00875]; 1073; 1079; 11.607096; TTAGTGG; 1.07886; 1.30093;
Sequence; USF-1 [T00875]; 1097; 1103; 10.653315; CCACGCC; 1.07886; 1.40071;
Sequence; USF-1 [T00875]; 1137; 1143; 10.653315; GGCGTGG; 1.07886; 1.40071;
Sequence; USF-1 [T00875]; 1425; 1431; 10.616595; ACGGTGG; 1.07886; 1.40071;
Sequence; WT1 I [T01840]; 47; 53; 7.130842; GTGGGCC; 0.71924; 0.86881;
Sequence; WT1 I [T01840]; 216; 222; 8.510618; TCCTCAC; 0.35962; 0.37340;
Sequence; WT1 I [T01840]; 306; 312; 6.133670; AGCACAC; 0.62933; 0.78463;
Sequence; WT1 I [T01840]; 331; 337; 0.997172; GTGGGTG; 0.17981; 0.26171;
Sequence; WT1 I [T01840]; 335; 341; 10.504962; GTGAGAA; 0.17981; 0.18485;
Sequence; WT1 I [T01840]; 372; 378; 3.988688; CTCCCAC; 0.44952; 0.57510;
Sequence; WT1 I [T01840]; 492; 498; 4.139327; GTGTGTC; 0.44952; 0.57510;
Sequence; WT1 I [T01840]; 505; 511; 4.985860; CTCTCAC; 0.53943; 0.70234;
Sequence; WT1 I [T01840]; 542; 548; 9.507790; TTCCCAC; 0.26971; 0.29129;
Sequence; WT1 I [T01840]; 702; 708; 7.130842; ACCTCAC; 0.71924; 0.86881;
Sequence; WT1 I [T01840]; 714; 720; 7.513446; TCCCCAC; 0.26971; 0.28595;
Sequence; WT1 I [T01840]; 754; 760; 5.136498; AACCCAC; 0.53943; 0.70234;
Sequence; WT1 I [T01840]; 808; 814; 7.513446; TCCCCAC; 0.26971; 0.28595;
Sequence; WT1 I [T01840]; 812; 818; 0.997172; CACCCAC; 0.17981; 0.26171;
Sequence; WT1 I [T01840]; 831; 837; 5.136498; GCCACAC; 0.53943; 0.70234;
Sequence; WT1 I [T01840]; 901; 907; 1.994344; CACTCAC; 0.26971; 0.36799;
Sequence; WT1 I [T01840]; 957; 963; 8.128015; GTCCCAC; 0.53943; 0.66488;
Sequence; WT1 I [T01840]; 982; 988; 5.136498; GACCCAC; 0.53943; 0.70234;
Sequence; WT1 I [T01840]; 1027; 1033; 9.507790; TGCTCAC; 0.26971; 0.29129;
Sequence; WT1 I [T01840]; 1056; 1062; 5.136498; GTGGGTC; 0.53943; 0.70234;
Sequence; WT1 I [T01840]; 1140; 1146; 9.507790; GTGGGAA; 0.26971; 0.29129;
Sequence; WT1 I [T01840]; 1202; 1208; 4.985860; GTGAGAG; 0.53943; 0.70234;
Sequence; p300 [T01427]; 9; 13; 3.482089; GGAGG; 2.87695; 3.25673;
Sequence; p300 [T01427]; 16; 20; 3.482089; GGAGG; 2.87695; 3.25673;
Sequence; p300 [T01427]; 56; 60; 0.000000; AGTCC; 1.43848; 1.48605;
Sequence; p300 [T01427]; 62; 66; 4.639858; GGATC; 4.31543; 4.25265;
Sequence; p300 [T01427]; 63; 67; 4.639858; GATCC; 4.31543; 4.25265;
Sequence; p300 [T01427]; 71; 75; 3.482089; CCTCC; 2.87695; 3.25673;
Sequence; p300 [T01427]; 92; 96; 3.482089; GGAGG; 2.87695; 3.25673;
Sequence; p300 [T01427]; 110; 114; 1.540766; GGATT; 1.43848; 1.30860;

Sequence; p300 [T01427]; 124; 128; 2.711705; GGACA; 4.31543; 4.54139;
Sequence; p300 [T01427]; 139; 143; 3.482089; GGAGA; 2.87695; 3.25673;
Sequence; p300 [T01427]; 159; 163; 5.408046; GGAAA; 2.87695; 2.82890;
Sequence; p300 [T01427]; 181; 185; 3.869475; GGAGC; 1.43848; 1.75385;
Sequence; p300 [T01427]; 193; 197; 3.099092; GGACC; 1.43848; 1.77606;
Sequence; p300 [T01427]; 199; 203; 4.252471; CATCC; 4.31543; 4.25265;
Sequence; p300 [T01427]; 214; 218; 4.252471; CATCC; 4.31543; 4.25265;
Sequence; p300 [T01427]; 248; 252; 2.711705; TGTCC; 4.31543; 4.54139;
Sequence; p300 [T01427]; 251; 255; 3.482089; CCTCC; 2.87695; 3.25673;
Sequence; p300 [T01427]; 258; 262; 3.869475; GCTCC; 1.43848; 1.75385;
Sequence; p300 [T01427]; 285; 289; 3.869475; GGAGC; 1.43848; 1.75385;
Sequence; p300 [T01427]; 368; 372; 3.869475; GGAGC; 1.43848; 1.75385;
Sequence; p300 [T01427]; 371; 375; 3.869475; GCTCC; 1.43848; 1.75385;
Sequence; p300 [T01427]; 385; 389; 3.099092; GGTCC; 1.43848; 1.77606;
Sequence; p300 [T01427]; 417; 421; 3.099092; GGACC; 1.43848; 1.77606;
Sequence; p300 [T01427]; 424; 428; 5.795432; GTTCC; 1.43848; 1.53260;
Sequence; p300 [T01427]; 432; 436; 5.408046; GGAAG; 2.87695; 2.82890;
Sequence; p300 [T01427]; 495; 499; 2.711705; TGTCC; 4.31543; 4.54139;
Sequence; p300 [T01427]; 519; 523; 4.252471; CATCC; 4.31543; 4.25265;
Sequence; p300 [T01427]; 541; 545; 2.696340; ATTCC; 4.31543; 4.54139;
Sequence; p300 [T01427]; 556; 560; 3.099092; GGACC; 1.43848; 1.77606;
Sequence; p300 [T01427]; 560; 564; 3.482089; CCTCC; 2.87695; 3.25673;
Sequence; p300 [T01427]; 573; 577; 5.408046; TTTCC; 2.87695; 2.82890;
Sequence; p300 [T01427]; 607; 611; 3.869475; GCTCC; 1.43848; 1.75385;
Sequence; p300 [T01427]; 658; 662; 5.408046; CTTCC; 2.87695; 2.82890;
Sequence; p300 [T01427]; 686; 690; 3.869475; GCTCC; 1.43848; 1.75385;
Sequence; p300 [T01427]; 712; 716; 5.408046; CTTCC; 2.87695; 2.82890;
Sequence; p300 [T01427]; 737; 741; 3.099092; GGACC; 1.43848; 1.77606;
Sequence; p300 [T01427]; 781; 785; 3.099092; GGACC; 1.43848; 1.77606;
Sequence; p300 [T01427]; 799; 803; 3.482089; GGAGG; 2.87695; 3.25673;
Sequence; p300 [T01427]; 806; 810; 4.252471; CATCC; 4.31543; 4.25265;
Sequence; p300 [T01427]; 869; 873; 0.000000; AGTCC; 1.43848; 1.48605;
Sequence; p300 [T01427]; 874; 878; 0.770383; GGAGT; 1.43848; 1.51166;
Sequence; p300 [T01427]; 913; 917; 3.482089; GGAGG; 2.87695; 3.25673;
Sequence; p300 [T01427]; 940; 944; 4.639858; GGATC; 4.31543; 4.25265;
Sequence; p300 [T01427]; 946; 950; 3.869475; GGAGC; 1.43848; 1.75385;
Sequence; p300 [T01427]; 956; 960; 0.000000; AGTCC; 1.43848; 1.48605;
Sequence; p300 [T01427]; 1023; 1027; 0.000000; GGACT; 1.43848; 1.48605;
Sequence; p300 [T01427]; 1063; 1067; 2.711705; GGACA; 4.31543; 4.54139;
Sequence; p300 [T01427]; 1078; 1082; 2.711705; GGACA; 4.31543; 4.54139;
Sequence; p300 [T01427]; 1103; 1107; 2.711705; CGTCC; 4.31543; 4.54139;
Sequence; p300 [T01427]; 1131; 1135; 5.408046; CTTCC; 2.87695; 2.82890;
Sequence; p300 [T01427]; 1143; 1147; 2.696340; GGAAT; 4.31543; 4.54139;
Sequence; p300 [T01427]; 1151; 1155; 4.252471; GGATG; 4.31543; 4.25265;
Sequence; p300 [T01427]; 1189; 1193; 5.795432; GTTCC; 1.43848; 1.53260;
Sequence; p300 [T01427]; 1209; 1213; 2.711705; GGACG; 4.31543; 4.54139;
Sequence; p300 [T01427]; 1231; 1235; 3.482089; GGAGA; 2.87695; 3.25673;
Sequence; p300 [T01427]; 1255; 1259; 5.795432; GTTCC; 1.43848; 1.53260;
Sequence; p300 [T01427]; 1261; 1265; 5.408046; GGAAA; 2.87695; 2.82890;

Sequence; p300 [T01427]; 1269; 1273; 5.408046; GGAAG; 2.87695; 2.82890;
Sequence; p300 [T01427]; 1279; 1283; 5.408046; GGAAA; 2.87695; 2.82890;
Sequence; p300 [T01427]; 1295; 1299; 0.000000; GGACT; 1.43848; 1.48605;
Sequence; p300 [T01427]; 1306; 1310; 4.252471; TATCC; 4.31543; 4.25265;
Sequence; p300 [T01427]; 1345; 1349; 5.408046; GGAAG; 2.87695; 2.82890;
Sequence; p300 [T01427]; 1359; 1363; 5.408046; GGAAA; 2.87695; 2.82890;
Sequence; p300 [T01427]; 1366; 1370; 1.540766; AATCC; 1.43848; 1.30860;
Sequence; p300 [T01427]; 1375; 1379; 2.711705; GGACA; 4.31543; 4.54139;
Sequence; p300 [T01427]; 1420; 1424; 3.482089; GGAGG; 2.87695; 3.25673;
Sequence; p300 [T01427]; 1423; 1427; 2.711705; GGACG; 4.31543; 4.54139;
Sequence; p300 [T01427]; 1462; 1466; 0.000000; GGACT; 1.43848; 1.48605;
Sequence; FACB [T02841]; 9; 11; 0.000000; GGA; 23.01562; 24.24849;
Sequence; FACB [T02841]; 16; 18; 0.000000; GGA; 23.01562; 24.24849;
Sequence; FACB [T02841]; 58; 60; 0.000000; TCC; 23.01562; 24.24849;
Sequence; FACB [T02841]; 62; 64; 0.000000; GGA; 23.01562; 24.24849;
Sequence; FACB [T02841]; 65; 67; 0.000000; TCC; 23.01562; 24.24849;
Sequence; FACB [T02841]; 73; 75; 0.000000; TCC; 23.01562; 24.24849;
Sequence; FACB [T02841]; 92; 94; 0.000000; GGA; 23.01562; 24.24849;
Sequence; FACB [T02841]; 110; 112; 0.000000; GGA; 23.01562; 24.24849;
Sequence; FACB [T02841]; 124; 126; 0.000000; GGA; 23.01562; 24.24849;
Sequence; FACB [T02841]; 139; 141; 0.000000; GGA; 23.01562; 24.24849;
Sequence; FACB [T02841]; 159; 161; 0.000000; GGA; 23.01562; 24.24849;
Sequence; FACB [T02841]; 181; 183; 0.000000; GGA; 23.01562; 24.24849;
Sequence; FACB [T02841]; 193; 195; 0.000000; GGA; 23.01562; 24.24849;
Sequence; FACB [T02841]; 201; 203; 0.000000; TCC; 23.01562; 24.24849;
Sequence; FACB [T02841]; 216; 218; 0.000000; TCC; 23.01562; 24.24849;
Sequence; FACB [T02841]; 250; 252; 0.000000; TCC; 23.01562; 24.24849;
Sequence; FACB [T02841]; 253; 255; 0.000000; TCC; 23.01562; 24.24849;
Sequence; FACB [T02841]; 260; 262; 0.000000; TCC; 23.01562; 24.24849;
Sequence; FACB [T02841]; 285; 287; 0.000000; GGA; 23.01562; 24.24849;
Sequence; FACB [T02841]; 368; 370; 0.000000; GGA; 23.01562; 24.24849;
Sequence; FACB [T02841]; 373; 375; 0.000000; TCC; 23.01562; 24.24849;
Sequence; FACB [T02841]; 387; 389; 0.000000; TCC; 23.01562; 24.24849;
Sequence; FACB [T02841]; 417; 419; 0.000000; GGA; 23.01562; 24.24849;
Sequence; FACB [T02841]; 426; 428; 0.000000; TCC; 23.01562; 24.24849;
Sequence; FACB [T02841]; 432; 434; 0.000000; GGA; 23.01562; 24.24849;
Sequence; FACB [T02841]; 497; 499; 0.000000; TCC; 23.01562; 24.24849;
Sequence; FACB [T02841]; 521; 523; 0.000000; TCC; 23.01562; 24.24849;
Sequence; FACB [T02841]; 543; 545; 0.000000; TCC; 23.01562; 24.24849;
Sequence; FACB [T02841]; 556; 558; 0.000000; GGA; 23.01562; 24.24849;
Sequence; FACB [T02841]; 562; 564; 0.000000; TCC; 23.01562; 24.24849;
Sequence; FACB [T02841]; 575; 577; 0.000000; TCC; 23.01562; 24.24849;
Sequence; FACB [T02841]; 609; 611; 0.000000; TCC; 23.01562; 24.24849;
Sequence; FACB [T02841]; 660; 662; 0.000000; TCC; 23.01562; 24.24849;
Sequence; FACB [T02841]; 688; 690; 0.000000; TCC; 23.01562; 24.24849;
Sequence; FACB [T02841]; 714; 716; 0.000000; TCC; 23.01562; 24.24849;
Sequence; FACB [T02841]; 737; 739; 0.000000; GGA; 23.01562; 24.24849;
Sequence; FACB [T02841]; 781; 783; 0.000000; GGA; 23.01562; 24.24849;
Sequence; FACB [T02841]; 799; 801; 0.000000; GGA; 23.01562; 24.24849;

Sequence; FACB [T02841]; 808; 810; 0.000000; TCC; 23.01562; 24.24849;
Sequence; FACB [T02841]; 871; 873; 0.000000; TCC; 23.01562; 24.24849;
Sequence; FACB [T02841]; 874; 876; 0.000000; GGA; 23.01562; 24.24849;
Sequence; FACB [T02841]; 913; 915; 0.000000; GGA; 23.01562; 24.24849;
Sequence; FACB [T02841]; 940; 942; 0.000000; GGA; 23.01562; 24.24849;
Sequence; FACB [T02841]; 946; 948; 0.000000; GGA; 23.01562; 24.24849;
Sequence; FACB [T02841]; 958; 960; 0.000000; TCC; 23.01562; 24.24849;
Sequence; FACB [T02841]; 1023; 1025; 0.000000; GGA; 23.01562; 24.24849;
Sequence; FACB [T02841]; 1063; 1065; 0.000000; GGA; 23.01562; 24.24849;
Sequence; FACB [T02841]; 1078; 1080; 0.000000; GGA; 23.01562; 24.24849;
Sequence; FACB [T02841]; 1105; 1107; 0.000000; TCC; 23.01562; 24.24849;
Sequence; FACB [T02841]; 1133; 1135; 0.000000; TCC; 23.01562; 24.24849;
Sequence; FACB [T02841]; 1143; 1145; 0.000000; GGA; 23.01562; 24.24849;
Sequence; FACB [T02841]; 1151; 1153; 0.000000; GGA; 23.01562; 24.24849;
Sequence; FACB [T02841]; 1191; 1193; 0.000000; TCC; 23.01562; 24.24849;
Sequence; FACB [T02841]; 1209; 1211; 0.000000; GGA; 23.01562; 24.24849;
Sequence; FACB [T02841]; 1231; 1233; 0.000000; GGA; 23.01562; 24.24849;
Sequence; FACB [T02841]; 1257; 1259; 0.000000; TCC; 23.01562; 24.24849;
Sequence; FACB [T02841]; 1261; 1263; 0.000000; GGA; 23.01562; 24.24849;
Sequence; FACB [T02841]; 1269; 1271; 0.000000; GGA; 23.01562; 24.24849;
Sequence; FACB [T02841]; 1279; 1281; 0.000000; GGA; 23.01562; 24.24849;
Sequence; FACB [T02841]; 1295; 1297; 0.000000; GGA; 23.01562; 24.24849;
Sequence; FACB [T02841]; 1308; 1310; 0.000000; TCC; 23.01562; 24.24849;
Sequence; FACB [T02841]; 1345; 1347; 0.000000; GGA; 23.01562; 24.24849;
Sequence; FACB [T02841]; 1359; 1361; 0.000000; GGA; 23.01562; 24.24849;
Sequence; FACB [T02841]; 1368; 1370; 0.000000; TCC; 23.01562; 24.24849;
Sequence; FACB [T02841]; 1375; 1377; 0.000000; GGA; 23.01562; 24.24849;
Sequence; FACB [T02841]; 1420; 1422; 0.000000; GGA; 23.01562; 24.24849;
Sequence; FACB [T02841]; 1423; 1425; 0.000000; GGA; 23.01562; 24.24849;
Sequence; FACB [T02841]; 1462; 1464; 0.000000; GGA; 23.01562; 24.24849;
Sequence; R2 [T00712]; 6; 11; 4.756447; GAGGGA; 2.15771; 2.25262;
Sequence; R2 [T00712]; 13; 18; 9.512894; GGCGGA; 5.39429; 5.48154;
Sequence; R2 [T00712]; 58; 63; 11.337888; TCCAGG; 1.79810; 2.01982;
Sequence; R2 [T00712]; 65; 70; 0.000000; TCCAAC; 1.07886; 1.10942;
Sequence; R2 [T00712]; 89; 94; 14.269340; CAGGGA; 4.31543; 4.37212;
Sequence; R2 [T00712]; 107; 112; 14.269340; TGAGGA; 4.31543; 4.37212;
Sequence; R2 [T00712]; 136; 141; 9.512894; GGCGGA; 5.39429; 5.48154;
Sequence; R2 [T00712]; 156; 161; 0.000000; GATGGA; 1.07886; 1.10942;
Sequence; R2 [T00712]; 178; 183; 9.512894; TGGGGA; 5.39429; 5.48154;
Sequence; R2 [T00712]; 190; 195; 14.269340; ATGGGA; 4.31543; 4.37212;
Sequence; R2 [T00712]; 201; 206; 14.269340; TCCCCT; 4.31543; 4.37212;
Sequence; R2 [T00712]; 216; 221; 14.269340; TCCTCA; 4.31543; 4.37212;
Sequence; R2 [T00712]; 250; 255; 9.512894; TCCTCC; 5.39429; 5.48154;
Sequence; R2 [T00712]; 373; 378; 4.756447; TCCCAC; 2.15771; 2.25262;
Sequence; R2 [T00712]; 387; 392; 14.269340; TCCTCA; 4.31543; 4.37212;
Sequence; R2 [T00712]; 429; 434; 14.269340; CGGGGA; 4.31543; 4.37212;
Sequence; R2 [T00712]; 497; 502; 11.337888; TCCTGC; 1.79810; 2.01982;
Sequence; R2 [T00712]; 543; 548; 4.756447; TCCCAC; 2.15771; 2.25262;
Sequence; R2 [T00712]; 553; 558; 14.269340; TAAGGA; 4.31543; 4.37212;

Sequence; R2 [T00712]; 575; 580; 4.756447; TCCACA; 2.15771; 2.25262;
Sequence; R2 [T00712]; 609; 614; 0.000000; TCCACC; 1.07886; 1.10942;
Sequence; R2 [T00712]; 660; 665; 14.269340; TCCCCT; 4.31543; 4.37212;
Sequence; R2 [T00712]; 688; 693; 0.000000; TCCACC; 1.07886; 1.10942;
Sequence; R2 [T00712]; 714; 719; 9.512894; TCCCCA; 5.39429; 5.48154;
Sequence; R2 [T00712]; 778; 783; 9.512894; GGCGGA; 5.39429; 5.48154;
Sequence; R2 [T00712]; 796; 801; 14.269340; CAGGGA; 4.31543; 4.37212;
Sequence; R2 [T00712]; 808; 813; 9.512894; TCCCCA; 5.39429; 5.48154;
Sequence; R2 [T00712]; 910; 915; 4.756447; GGGGGA; 2.15771; 2.25262;
Sequence; R2 [T00712]; 958; 963; 4.756447; TCCCAC; 2.15771; 2.25262;
Sequence; R2 [T00712]; 1020; 1025; 1.824994; GCTGGA; 0.35962; 0.40224;
Sequence; R2 [T00712]; 1060; 1065; 9.512894; GTCGGA; 5.39429; 5.48154;
Sequence; R2 [T00712]; 1075; 1080; 9.512894; AGTGGA; 5.39429; 5.48154;
Sequence; R2 [T00712]; 1105; 1110; 9.512894; TCCTTC; 5.39429; 5.48154;
Sequence; R2 [T00712]; 1140; 1145; 4.756447; GTGGGA; 2.15771; 2.25262;
Sequence; R2 [T00712]; 1148; 1153; 9.512894; GAAGGA; 5.39429; 5.48154;
Sequence; R2 [T00712]; 1191; 1196; 9.512894; TCCCAA; 5.39429; 5.48154;
Sequence; R2 [T00712]; 1206; 1211; 4.756447; GAGGGA; 2.15771; 2.25262;
Sequence; R2 [T00712]; 1258; 1263; 11.337888; CCTGGA; 1.79810; 2.01982;
Sequence; R2 [T00712]; 1266; 1271; 9.512894; GTAGGA; 5.39429; 5.48154;
Sequence; R2 [T00712]; 1276; 1281; 9.512894; TAGGGA; 5.39429; 5.48154;
Sequence; R2 [T00712]; 1308; 1313; 9.512894; TCCCCA; 5.39429; 5.48154;
Sequence; R2 [T00712]; 1356; 1361; 11.337888; GCAGGA; 1.79810; 2.01982;
Sequence; R2 [T00712]; 1372; 1377; 4.756447; GGGGGA; 2.15771; 2.25262;
Sequence; R2 [T00712]; 1417; 1422; 9.512894; TTGGGA; 5.39429; 5.48154;
Sequence; R2 [T00712]; 1420; 1425; 9.512894; GGAGGA; 5.39429; 5.48154;
Sequence; Nrf2:MafK [T05666]; 59; 65; 9.575183; CCAGGAT; 1.43848; 1.62233;
Sequence; Nrf2:MafK [T05666]; 112; 118; 8.033921; ATTCTGC; 1.07886; 1.06170;
Sequence; Nrf2:MafK [T05666]; 174; 180; 9.333580; GTGCTGG; 1.43848; 1.62233;
Sequence; Nrf2:MafK [T05666]; 210; 216; 0.000000; TCAGCAT; 0.53943; 0.42958;
Sequence; Nrf2:MafK [T05666]; 288; 294; 9.333580; GCAGTAG; 1.43848; 1.62233;
Sequence; Nrf2:MafK [T05666]; 295; 301; 4.016961; ACAGCAA; 0.53943; 0.47835;
Sequence; Nrf2:MafK [T05666]; 470; 476; 9.333580; GCAGCAG; 1.43848; 1.62233;
Sequence; Nrf2:MafK [T05666]; 478; 484; 1.299659; CTTCTGA; 0.71924; 0.65856;
Sequence; Nrf2:MafK [T05666]; 496; 502; 10.874842; GTCCTGC; 0.35962; 0.44531;
Sequence; Nrf2:MafK [T05666]; 748; 754; 0.000000; TTTCTGA; 0.53943; 0.42958;
Sequence; Nrf2:MafK [T05666]; 838; 844; 0.000000; ATGCTGA; 0.53943; 0.42958;
Sequence; Nrf2:MafK [T05666]; 943; 949; 2.840920; TCAGGAG; 0.17981; 0.18268;
Sequence; Nrf2:MafK [T05666]; 1018; 1024; 8.033921; TTGCTGG; 1.07886; 1.06170;
Sequence; Nrf2:MafK [T05666]; 1035; 1041; 1.299659; TCAGCAC; 0.71924; 0.65856;
Sequence; Nrf2:MafK [T05666]; 1171; 1177; 9.333580; CCAGTAG; 1.43848; 1.62233;
Sequence; Nrf2:MafK [T05666]; 1184; 1190; 5.316619; CTTCTGT; 0.71924; 0.72880;
Sequence; Nrf2:MafK [T05666]; 1222; 1228; 8.033921; CCAGAAA; 1.07886; 1.06170;
Sequence; Nrf2:MafK [T05666]; 1256; 1262; 9.575183; TTCCTGG; 1.43848; 1.62233;
Sequence; Nrf2:MafK [T05666]; 1302; 1308; 0.000000; TCAGTAT; 0.53943; 0.42958;
Sequence; Nrf2:MafK [T05666]; 1356; 1362; 9.575183; GCAGGAA; 1.43848; 1.62233;
Sequence; Nrf2:MafK [T05666]; 1367; 1373; 9.575183; ATCCTGG; 1.43848; 1.62233;
Sequence; Nrf2:MafK [T05666]; 1442; 1448; 1.299659; TCAGCAG; 0.71924; 0.65856;
Sequence; Nrf2:MafK [T05666]; 1445; 1451; 8.033921; GCAGTAA; 1.07886; 1.06170;

Sequence; LVb-binding factor [T00477]; 60; 66; 14.285714; CAGGATC; 1.88800; 1.72064;
Sequence; LVb-binding factor [T00477]; 495; 501; 14.285714; TGTCCTG; 1.88800; 1.72064;
Sequence; LVb-binding factor [T00477]; 1357; 1363; 14.285714; CAGGAAA; 1.88800; 1.72064;
Sequence; LVb-binding factor [T00477]; 1366; 1372; 14.285714; AATCCTG; 1.88800; 1.72064;
Sequence; HELIOS [T06012]; 61; 66; 5.714286; AGGATC; 0.71924; 0.67645;
Sequence; HELIOS [T06012]; 71; 76; 7.142857; CCTCCT; 1.43848; 1.52488;
Sequence; HELIOS [T06012]; 109; 114; 0.000000; AGGATT; 0.71924; 0.57848;
Sequence; HELIOS [T06012]; 123; 128; 2.857143; AGGACA; 0.71924; 0.70647;
Sequence; HELIOS [T06012]; 214; 219; 5.714286; CATCCT; 0.71924; 0.67645;
Sequence; HELIOS [T06012]; 248; 253; 2.857143; TGTCCT; 0.71924; 0.70647;
Sequence; HELIOS [T06012]; 284; 289; 7.142857; AGGAGC; 1.43848; 1.52488;
Sequence; HELIOS [T06012]; 367; 372; 7.142857; AGGAGC; 1.43848; 1.52488;
Sequence; HELIOS [T06012]; 385; 390; 8.571428; GGTCCT; 0.71924; 0.82613;
Sequence; HELIOS [T06012]; 495; 500; 2.857143; TGTCCT; 0.71924; 0.70647;
Sequence; HELIOS [T06012]; 519; 524; 5.714286; CATCCT; 0.71924; 0.67645;
Sequence; HELIOS [T06012]; 555; 560; 8.571428; AGGACC; 0.71924; 0.82613;
Sequence; HELIOS [T06012]; 736; 741; 8.571428; AGGACC; 0.71924; 0.82613;
Sequence; HELIOS [T06012]; 939; 944; 5.714286; AGGATC; 0.71924; 0.67645;
Sequence; HELIOS [T06012]; 945; 950; 7.142857; AGGAGC; 1.43848; 1.52488;
Sequence; HELIOS [T06012]; 1103; 1108; 8.571428; CGTCCT; 0.71924; 0.82613;
Sequence; HELIOS [T06012]; 1150; 1155; 5.714286; AGGATG; 0.71924; 0.67645;
Sequence; HELIOS [T06012]; 1230; 1235; 1.428571; AGGAGA; 1.43848; 1.30402;
Sequence; HELIOS [T06012]; 1255; 1260; 7.142857; GTTCCT; 1.43848; 1.52488;
Sequence; HELIOS [T06012]; 1268; 1273; 7.142857; AGGAAG; 1.43848; 1.52488;
Sequence; HELIOS [T06012]; 1294; 1299; 2.857143; AGGACT; 0.71924; 0.70647;
Sequence; HELIOS [T06012]; 1358; 1363; 1.428571; AGGAAA; 1.43848; 1.30402;
Sequence; HELIOS [T06012]; 1366; 1371; 0.000000; AATCCT; 0.71924; 0.57848;
Sequence; HELIOS [T06012]; 1422; 1427; 8.571428; AGGACG; 0.71924; 0.82613;
Sequence; RC2 [T00724]; 66; 71; 10.398498; CCAACC; 5.75391; 6.82079;
Sequence; RC2 [T00724]; 84; 89; 8.645460; ATCACC; 2.87695; 3.05117;
Sequence; RC2 [T00724]; 128; 133; 8.645460; ATCACC; 2.87695; 3.05117;
Sequence; RC2 [T00724]; 192; 197; 10.398498; GGGACC; 5.75391; 6.82079;
Sequence; RC2 [T00724]; 298; 303; 10.398498; GCAACC; 5.75391; 6.82079;
Sequence; RC2 [T00724]; 308; 313; 10.398498; CACACC; 5.75391; 6.82079;
Sequence; RC2 [T00724]; 334; 339; 10.398498; GGTGAG; 5.75391; 6.82079;
Sequence; RC2 [T00724]; 340; 345; 1.753037; AAGACC; 1.43848; 1.42783;
Sequence; RC2 [T00724]; 347; 352; 3.506075; GGTCAG; 2.87695; 3.17608;
Sequence; RC2 [T00724]; 360; 365; 6.892423; TATACC; 2.87695; 2.78172;
Sequence; RC2 [T00724]; 385; 390; 8.645460; GGTCCT; 2.87695; 3.05117;
Sequence; RC2 [T00724]; 400; 405; 8.645460; GGTTCT; 2.87695; 3.05117;
Sequence; RC2 [T00724]; 403; 408; 13.784846; TCTACC; 1.43848; 1.49129;
Sequence; RC2 [T00724]; 416; 421; 10.398498; CGGACC; 5.75391; 6.82079;
Sequence; RC2 [T00724]; 443; 448; 8.645460; AACACC; 2.87695; 3.05117;
Sequence; RC2 [T00724]; 447; 452; 10.398498; CCAACC; 5.75391; 6.82079;
Sequence; RC2 [T00724]; 500; 505; 13.784846; TGCACC; 1.43848; 1.49129;
Sequence; RC2 [T00724]; 507; 512; 10.398498; CTCACC; 5.75391; 6.82079;
Sequence; RC2 [T00724]; 555; 560; 8.645460; AGGACC; 2.87695; 3.05117;
Sequence; RC2 [T00724]; 609; 614; 13.784846; TCCACC; 1.43848; 1.49129;
Sequence; RC2 [T00724]; 617; 622; 3.506075; CAGACC; 2.87695; 3.17608;

Sequence; RC2 [T00724]; 677; 682; 10.398498; GTCACC; 5.75391; 6.82079;
Sequence; RC2 [T00724]; 688; 693; 13.784846; TCCACC; 1.43848; 1.49129;
Sequence; RC2 [T00724]; 699; 704; 3.506075; CTGACC; 2.87695; 3.17608;
Sequence; RC2 [T00724]; 704; 709; 10.398498; CTCACC; 5.75391; 6.82079;
Sequence; RC2 [T00724]; 736; 741; 8.645460; AGGACC; 2.87695; 3.05117;
Sequence; RC2 [T00724]; 752; 757; 6.892423; TGAACC; 2.87695; 2.78172;
Sequence; RC2 [T00724]; 763; 768; 3.506075; CAGACC; 2.87695; 3.17608;
Sequence; RC2 [T00724]; 780; 785; 10.398498; CGGACC; 5.75391; 6.82079;
Sequence; RC2 [T00724]; 802; 807; 6.892423; GGTTCA; 2.87695; 2.78172;
Sequence; RC2 [T00724]; 881; 886; 8.645460; GGTCGT; 2.87695; 3.05117;
Sequence; RC2 [T00724]; 896; 901; 10.398498; GGTTGC; 5.75391; 6.82079;
Sequence; RC2 [T00724]; 920; 925; 6.892423; TATACC; 2.87695; 2.78172;
Sequence; RC2 [T00724]; 980; 985; 1.753037; ATGACC; 1.43848; 1.42783;
Sequence; RC2 [T00724]; 993; 998; 8.645460; ACAACC; 2.87695; 3.05117;
Sequence; RC2 [T00724]; 1029; 1034; 10.398498; CTCACC; 5.75391; 6.82079;
Sequence; RC2 [T00724]; 1059; 1064; 10.398498; GGTCGG; 5.75391; 6.82079;
Sequence; RC2 [T00724]; 1093; 1098; 1.753037; AAGACC; 1.43848; 1.42783;
Sequence; RC2 [T00724]; 1167; 1172; 8.645460; AACACC; 2.87695; 3.05117;
Sequence; RC2 [T00724]; 1430; 1435; 3.506075; GGTCTC; 2.87695; 3.17608;
Sequence; MYB2 [T02536]; 68; 72; 3.846154; AACCC; 8.63086; 8.65976;
Sequence; MYB2 [T02536]; 265; 269; 1.923077; GAGTT; 5.75391; 5.58047;
Sequence; MYB2 [T02536]; 300; 304; 3.846154; AACCC; 8.63086; 8.65976;
Sequence; MYB2 [T02536]; 399; 403; 5.769231; AGGTT; 5.75391; 5.42276;
Sequence; MYB2 [T02536]; 422; 426; 5.769231; TTGTT; 5.75391; 5.42276;
Sequence; MYB2 [T02536]; 443; 447; 5.769231; AACAC; 5.75391; 5.42276;
Sequence; MYB2 [T02536]; 449; 453; 3.846154; AACCC; 8.63086; 8.65976;
Sequence; MYB2 [T02536]; 538; 542; 7.692307; AACAT; 1.43848; 1.14252;
Sequence; MYB2 [T02536]; 591; 595; 3.846154; AACTT; 8.63086; 8.65976;
Sequence; MYB2 [T02536]; 600; 604; 3.846154; AAGTT; 8.63086; 8.65976;
Sequence; MYB2 [T02536]; 656; 660; 3.846154; AACTT; 8.63086; 8.65976;
Sequence; MYB2 [T02536]; 666; 670; 5.769231; ACGTT; 5.75391; 5.42276;
Sequence; MYB2 [T02536]; 754; 758; 3.846154; AACCC; 8.63086; 8.65976;
Sequence; MYB2 [T02536]; 770; 774; 0.000000; CAGTT; 1.43848; 1.27082;
Sequence; MYB2 [T02536]; 786; 790; 3.846154; AACAG; 8.63086; 8.65976;
Sequence; MYB2 [T02536]; 801; 805; 5.769231; AGGTT; 5.75391; 5.42276;
Sequence; MYB2 [T02536]; 895; 899; 3.846154; GGGTT; 8.63086; 8.65976;
Sequence; MYB2 [T02536]; 932; 936; 7.692307; ATGTT; 1.43848; 1.14252;
Sequence; MYB2 [T02536]; 995; 999; 3.846154; AACCA; 8.63086; 8.65976;
Sequence; MYB2 [T02536]; 1043; 1047; 5.769231; TTGTT; 5.75391; 5.42276;
Sequence; MYB2 [T02536]; 1070; 1074; 5.769231; TTGTT; 5.75391; 5.42276;
Sequence; MYB2 [T02536]; 1088; 1092; 0.000000; AACTG; 1.43848; 1.27082;
Sequence; MYB2 [T02536]; 1129; 1133; 3.846154; AACTT; 8.63086; 8.65976;
Sequence; MYB2 [T02536]; 1167; 1171; 5.769231; AACAC; 5.75391; 5.42276;
Sequence; MYB2 [T02536]; 1187; 1191; 3.846154; CTGTT; 8.63086; 8.65976;
Sequence; MYB2 [T02536]; 1251; 1255; 3.846154; AACAG; 8.63086; 8.65976;
Sequence; MYB2 [T02536]; 1253; 1257; 0.000000; CAGTT; 1.43848; 1.27082;
Sequence; MYB2 [T02536]; 1287; 1291; 0.000000; AACTG; 1.43848; 1.27082;
Sequence; MYB2 [T02536]; 1315; 1319; 0.000000; AACTG; 1.43848; 1.27082;
Sequence; MYB2 [T02536]; 1325; 1329; 3.846154; AACGC; 8.63086; 8.65976;

Sequence; MYB2 [T02536]; 1386; 1390; 7.692307; ATGTT; 1.43848; 1.14252;
Sequence; Zic3 [T04671]; 45; 48; 2.676494; AGGT; 5.75391; 5.56125;
Sequence; Zic3 [T04671]; 69; 72; 0.000000; ACCC; 5.75391; 6.94211;
Sequence; Zic3 [T04671]; 77; 80; 2.676494; AGGT; 5.75391; 5.56125;
Sequence; Zic3 [T04671]; 87; 90; 2.944143; ACCA; 11.50781; 12.78221;
Sequence; Zic3 [T04671]; 131; 134; 2.944143; ACCA; 11.50781; 12.78221;
Sequence; Zic3 [T04671]; 195; 198; 0.000000; ACCC; 5.75391; 6.94211;
Sequence; Zic3 [T04671]; 301; 304; 0.000000; ACCC; 5.75391; 6.94211;
Sequence; Zic3 [T04671]; 311; 314; 2.944143; ACCA; 11.50781; 12.78221;
Sequence; Zic3 [T04671]; 329; 332; 2.676494; AGGT; 5.75391; 5.56125;
Sequence; Zic3 [T04671]; 333; 336; 0.000000; GGGT; 5.75391; 6.94211;
Sequence; Zic3 [T04671]; 343; 346; 3.211792; ACCG; 11.50781; 12.78221;
Sequence; Zic3 [T04671]; 346; 349; 0.000000; GGGT; 5.75391; 6.94211;
Sequence; Zic3 [T04671]; 363; 366; 2.944143; ACCA; 11.50781; 12.78221;
Sequence; Zic3 [T04671]; 377; 380; 2.944143; ACCA; 11.50781; 12.78221;
Sequence; Zic3 [T04671]; 380; 383; 2.944143; ACCA; 11.50781; 12.78221;
Sequence; Zic3 [T04671]; 384; 387; 2.676494; AGGT; 5.75391; 5.56125;
Sequence; Zic3 [T04671]; 399; 402; 2.676494; AGGT; 5.75391; 5.56125;
Sequence; Zic3 [T04671]; 406; 409; 2.676494; ACCT; 5.75391; 5.56125;
Sequence; Zic3 [T04671]; 419; 422; 2.676494; ACCT; 5.75391; 5.56125;
Sequence; Zic3 [T04671]; 438; 441; 2.676494; ACCT; 5.75391; 5.56125;
Sequence; Zic3 [T04671]; 446; 449; 2.944143; ACCA; 11.50781; 12.78221;
Sequence; Zic3 [T04671]; 450; 453; 0.000000; ACCC; 5.75391; 6.94211;
Sequence; Zic3 [T04671]; 503; 506; 2.676494; ACCT; 5.75391; 5.56125;
Sequence; Zic3 [T04671]; 510; 513; 0.000000; ACCC; 5.75391; 6.94211;
Sequence; Zic3 [T04671]; 515; 518; 3.211792; ACCG; 11.50781; 12.78221;
Sequence; Zic3 [T04671]; 558; 561; 0.000000; ACCC; 5.75391; 6.94211;
Sequence; Zic3 [T04671]; 612; 615; 0.000000; ACCC; 5.75391; 6.94211;
Sequence; Zic3 [T04671]; 620; 623; 0.000000; ACCC; 5.75391; 6.94211;
Sequence; Zic3 [T04671]; 680; 683; 0.000000; ACCC; 5.75391; 6.94211;
Sequence; Zic3 [T04671]; 691; 694; 0.000000; ACCC; 5.75391; 6.94211;
Sequence; Zic3 [T04671]; 702; 705; 2.676494; ACCT; 5.75391; 5.56125;
Sequence; Zic3 [T04671]; 707; 710; 0.000000; ACCC; 5.75391; 6.94211;
Sequence; Zic3 [T04671]; 719; 722; 2.676494; ACCT; 5.75391; 5.56125;
Sequence; Zic3 [T04671]; 739; 742; 0.000000; ACCC; 5.75391; 6.94211;
Sequence; Zic3 [T04671]; 755; 758; 0.000000; ACCC; 5.75391; 6.94211;
Sequence; Zic3 [T04671]; 759; 762; 0.000000; ACCC; 5.75391; 6.94211;
Sequence; Zic3 [T04671]; 766; 769; 2.676494; ACCT; 5.75391; 5.56125;
Sequence; Zic3 [T04671]; 783; 786; 2.944143; ACCA; 11.50781; 12.78221;
Sequence; Zic3 [T04671]; 801; 804; 2.676494; AGGT; 5.75391; 5.56125;
Sequence; Zic3 [T04671]; 813; 816; 0.000000; ACCC; 5.75391; 6.94211;
Sequence; Zic3 [T04671]; 817; 820; 2.676494; ACCT; 5.75391; 5.56125;
Sequence; Zic3 [T04671]; 880; 883; 0.000000; GGGT; 5.75391; 6.94211;
Sequence; Zic3 [T04671]; 895; 898; 0.000000; GGGT; 5.75391; 6.94211;
Sequence; Zic3 [T04671]; 923; 926; 2.676494; ACCT; 5.75391; 5.56125;
Sequence; Zic3 [T04671]; 962; 965; 2.676494; ACCT; 5.75391; 5.56125;
Sequence; Zic3 [T04671]; 971; 974; 2.676494; ACCT; 5.75391; 5.56125;
Sequence; Zic3 [T04671]; 983; 986; 0.000000; ACCC; 5.75391; 6.94211;
Sequence; Zic3 [T04671]; 996; 999; 2.944143; ACCA; 11.50781; 12.78221;

Sequence; Zic3 [T04671]; 1032; 1035; 2.676494; ACCT; 5.75391; 5.56125;
Sequence; Zic3 [T04671]; 1058; 1061; 0.000000; GGGT; 5.75391; 6.94211;
Sequence; Zic3 [T04671]; 1096; 1099; 2.944143; ACCA; 11.50781; 12.78221;
Sequence; Zic3 [T04671]; 1170; 1173; 2.944143; ACCA; 11.50781; 12.78221;
Sequence; Zic3 [T04671]; 1426; 1429; 3.211792; CGGT; 11.50781; 12.78221;
Sequence; Zic3 [T04671]; 1429; 1432; 2.944143; TGGT; 11.50781; 12.78221;
Sequence; NF-1 [T00537]; 23; 27; 0.000000; GCCAA; 1.43848; 1.59675;
Sequence; NF-1 [T00537]; 35; 39; 0.686673; GCCAG; 1.43848; 1.77606;
Sequence; NF-1 [T00537]; 79; 83; 1.716684; GTGGC; 2.87695; 3.31492;
Sequence; NF-1 [T00537]; 268; 272; 0.000000; TTGGC; 1.43848; 1.59675;
Sequence; NF-1 [T00537]; 486; 490; 0.000000; GCCAA; 1.43848; 1.59675;
Sequence; NF-1 [T00537]; 731; 735; 0.686673; GCCAG; 1.43848; 1.77606;
Sequence; NF-1 [T00537]; 831; 835; 1.716684; GCCAC; 2.87695; 3.31492;
Sequence; NF-1 [T00537]; 852; 856; 0.000000; GCCAA; 1.43848; 1.59675;
Sequence; NF-1 [T00537]; 968; 972; 1.716684; GCCAC; 2.87695; 3.31492;
Sequence; NF-1 [T00537]; 977; 981; 1.545015; GCCAT; 2.87695; 3.31492;
Sequence; NF-1 [T00537]; 990; 994; 1.716684; GCCAC; 2.87695; 3.31492;
Sequence; NF-1 [T00537]; 1010; 1014; 1.545015; GCCAT; 2.87695; 3.31492;
Sequence; NF-1 [T00537]; 1153; 1157; 1.545015; ATGGC; 2.87695; 3.31492;
Sequence; NF-1 [T00537]; 1177; 1181; 0.686673; GCCAG; 1.43848; 1.77606;
Sequence; NF-1 [T00537]; 1221; 1225; 0.686673; GCCAG; 1.43848; 1.77606;
Sequence; NF-1 [T00537]; 1246; 1250; 0.000000; GCCAA; 1.43848; 1.59675;
Sequence; ENKTF-1 [T00255]; 19; 26; 13.885529; GGCAGCCA; 0.80914; 0.94544;
Sequence; ENKTF-1 [T00255]; 31; 38; 5.687009; CTAGGCCA; 0.53943; 0.61780;
Sequence; ENKTF-1 [T00255]; 80; 87; 12.629773; TGGCATCA; 1.61829; 1.76700;
Sequence; ENKTF-1 [T00255]; 269; 276; 6.942764; TGGCAAAG; 1.07886; 1.32781;
Sequence; ENKTF-1 [T00255]; 482; 489; 12.629773; TGATGCCA; 1.61829; 1.76700;
Sequence; ENKTF-1 [T00255]; 727; 734; 6.942764; CACAGCCA; 1.07886; 1.32781;
Sequence; ENKTF-1 [T00255]; 827; 834; 12.629773; TGAGGCCA; 1.61829; 1.76700;
Sequence; ENKTF-1 [T00255]; 848; 855; 12.629773; TCATGCCA; 1.61829; 1.76700;
Sequence; ENKTF-1 [T00255]; 964; 971; 5.687009; CTGGGCCA; 0.53943; 0.61780;
Sequence; ENKTF-1 [T00255]; 973; 980; 6.942764; CTTAGCCA; 1.07886; 1.32781;
Sequence; ENKTF-1 [T00255]; 986; 993; 6.942764; CACGGCCA; 1.07886; 1.32781;
Sequence; ENKTF-1 [T00255]; 1006; 1013; 8.198520; CCTTGCCA; 0.53943; 0.70790;
Sequence; ENKTF-1 [T00255]; 1154; 1161; 11.374018; TGGCTTTT; 0.80914; 0.81967;
Sequence; ENKTF-1 [T00255]; 1173; 1180; 13.885529; AGTAGCCA; 0.80914; 0.94544;
Sequence; ENKTF-1 [T00255]; 1217; 1224; 6.942764; CTCAGCCA; 1.07886; 1.32781;
Sequence; ENKTF-1 [T00255]; 1242; 1249; 11.374018; AAAGGCCA; 0.80914; 0.81967;
Sequence; TF68 [T00809]; 79; 88; 8.431005; GTGGCATCAC; 0.05479; 0.05909;
Sequence; TF68 [T00809]; 123; 132; 12.548836; AGGACATCAC; 0.18964; 0.19142;
Sequence; Msx-1 [T02072]; 47; 49; 0.000000; GTG; 23.01562; 25.28558;
Sequence; Msx-1 [T02072]; 79; 81; 0.000000; GTG; 23.01562; 25.28558;
Sequence; Msx-1 [T02072]; 86; 88; 0.000000; CAC; 23.01562; 25.28558;
Sequence; Msx-1 [T02072]; 130; 132; 0.000000; CAC; 23.01562; 25.28558;
Sequence; Msx-1 [T02072]; 133; 135; 0.000000; CAC; 23.01562; 25.28558;
Sequence; Msx-1 [T02072]; 174; 176; 0.000000; GTG; 23.01562; 25.28558;
Sequence; Msx-1 [T02072]; 185; 187; 0.000000; CAC; 23.01562; 25.28558;
Sequence; Msx-1 [T02072]; 220; 222; 0.000000; CAC; 23.01562; 25.28558;
Sequence; Msx-1 [T02072]; 247; 249; 0.000000; GTG; 23.01562; 25.28558;

Sequence; Msx-1 [T02072]; 308; 310; 0.000000; CAC; 23.01562; 25.28558;
Sequence; Msx-1 [T02072]; 310; 312; 0.000000; CAC; 23.01562; 25.28558;
Sequence; Msx-1 [T02072]; 313; 315; 0.000000; CAC; 23.01562; 25.28558;
Sequence; Msx-1 [T02072]; 331; 333; 0.000000; GTG; 23.01562; 25.28558;
Sequence; Msx-1 [T02072]; 335; 337; 0.000000; GTG; 23.01562; 25.28558;
Sequence; Msx-1 [T02072]; 352; 354; 0.000000; GTG; 23.01562; 25.28558;
Sequence; Msx-1 [T02072]; 376; 378; 0.000000; CAC; 23.01562; 25.28558;
Sequence; Msx-1 [T02072]; 379; 381; 0.000000; CAC; 23.01562; 25.28558;
Sequence; Msx-1 [T02072]; 437; 439; 0.000000; CAC; 23.01562; 25.28558;
Sequence; Msx-1 [T02072]; 445; 447; 0.000000; CAC; 23.01562; 25.28558;
Sequence; Msx-1 [T02072]; 492; 494; 0.000000; GTG; 23.01562; 25.28558;
Sequence; Msx-1 [T02072]; 494; 496; 0.000000; GTG; 23.01562; 25.28558;
Sequence; Msx-1 [T02072]; 502; 504; 0.000000; CAC; 23.01562; 25.28558;
Sequence; Msx-1 [T02072]; 509; 511; 0.000000; CAC; 23.01562; 25.28558;
Sequence; Msx-1 [T02072]; 546; 548; 0.000000; CAC; 23.01562; 25.28558;
Sequence; Msx-1 [T02072]; 577; 579; 0.000000; CAC; 23.01562; 25.28558;
Sequence; Msx-1 [T02072]; 611; 613; 0.000000; CAC; 23.01562; 25.28558;
Sequence; Msx-1 [T02072]; 633; 635; 0.000000; GTG; 23.01562; 25.28558;
Sequence; Msx-1 [T02072]; 646; 648; 0.000000; GTG; 23.01562; 25.28558;
Sequence; Msx-1 [T02072]; 679; 681; 0.000000; CAC; 23.01562; 25.28558;
Sequence; Msx-1 [T02072]; 690; 692; 0.000000; CAC; 23.01562; 25.28558;
Sequence; Msx-1 [T02072]; 706; 708; 0.000000; CAC; 23.01562; 25.28558;
Sequence; Msx-1 [T02072]; 718; 720; 0.000000; CAC; 23.01562; 25.28558;
Sequence; Msx-1 [T02072]; 727; 729; 0.000000; CAC; 23.01562; 25.28558;
Sequence; Msx-1 [T02072]; 758; 760; 0.000000; CAC; 23.01562; 25.28558;
Sequence; Msx-1 [T02072]; 792; 794; 0.000000; CAC; 23.01562; 25.28558;
Sequence; Msx-1 [T02072]; 812; 814; 0.000000; CAC; 23.01562; 25.28558;
Sequence; Msx-1 [T02072]; 816; 818; 0.000000; CAC; 23.01562; 25.28558;
Sequence; Msx-1 [T02072]; 833; 835; 0.000000; CAC; 23.01562; 25.28558;
Sequence; Msx-1 [T02072]; 835; 837; 0.000000; CAC; 23.01562; 25.28558;
Sequence; Msx-1 [T02072]; 901; 903; 0.000000; CAC; 23.01562; 25.28558;
Sequence; Msx-1 [T02072]; 905; 907; 0.000000; CAC; 23.01562; 25.28558;
Sequence; Msx-1 [T02072]; 961; 963; 0.000000; CAC; 23.01562; 25.28558;
Sequence; Msx-1 [T02072]; 970; 972; 0.000000; CAC; 23.01562; 25.28558;
Sequence; Msx-1 [T02072]; 986; 988; 0.000000; CAC; 23.01562; 25.28558;
Sequence; Msx-1 [T02072]; 992; 994; 0.000000; CAC; 23.01562; 25.28558;
Sequence; Msx-1 [T02072]; 1031; 1033; 0.000000; CAC; 23.01562; 25.28558;
Sequence; Msx-1 [T02072]; 1039; 1041; 0.000000; CAC; 23.01562; 25.28558;
Sequence; Msx-1 [T02072]; 1056; 1058; 0.000000; GTG; 23.01562; 25.28558;
Sequence; Msx-1 [T02072]; 1076; 1078; 0.000000; GTG; 23.01562; 25.28558;
Sequence; Msx-1 [T02072]; 1098; 1100; 0.000000; CAC; 23.01562; 25.28558;
Sequence; Msx-1 [T02072]; 1114; 1116; 0.000000; CAC; 23.01562; 25.28558;
Sequence; Msx-1 [T02072]; 1140; 1142; 0.000000; GTG; 23.01562; 25.28558;
Sequence; Msx-1 [T02072]; 1169; 1171; 0.000000; CAC; 23.01562; 25.28558;
Sequence; Msx-1 [T02072]; 1202; 1204; 0.000000; GTG; 23.01562; 25.28558;
Sequence; Msx-1 [T02072]; 1342; 1344; 0.000000; CAC; 23.01562; 25.28558;
Sequence; Msx-1 [T02072]; 1349; 1351; 0.000000; GTG; 23.01562; 25.28558;
Sequence; Msx-1 [T02072]; 1392; 1394; 0.000000; CAC; 23.01562; 25.28558;
Sequence; Msx-1 [T02072]; 1428; 1430; 0.000000; GTG; 23.01562; 25.28558;

Sequence; RAR-gamma [T00720]; 82; 89; 5.085116; GCATCACC; 0.17981; 0.19066;
Sequence; RAR-gamma [T00720]; 126; 133; 6.005075; ACATCACC; 0.08990; 0.09120;
Sequence; RAR-gamma [T00720]; 334; 341; 3.232527; GGTGAGAA; 0.17981; 0.17074;
Sequence; RAR-gamma [T00720]; 505; 512; 2.312568; CTCTCACC; 0.08990; 0.09309;
Sequence; RAR-gamma [T00720]; 675; 682; 4.625136; GCGTCACC; 0.15733; 0.16936;
Sequence; RAR-gamma [T00720]; 702; 709; 4.625136; ACCTCACC; 0.15733; 0.16936;
Sequence; RAR-gamma [T00720]; 1027; 1034; 1.379938; TGCTCACC; 0.08990; 0.09936;
Sequence; ABF1 [T00056]; 84; 89; 0.310085; ATCACC; 1.07886; 1.02079;
Sequence; ABF1 [T00056]; 128; 133; 0.310085; ATCACC; 1.07886; 1.02079;
Sequence; ABF1 [T00056]; 235; 240; 2.171892; ATCATC; 1.07886; 0.81566;
Sequence; ABF1 [T00056]; 276; 281; 5.274904; GCTGAT; 1.07886; 0.98547;
Sequence; ABF1 [T00056]; 396; 401; 5.274904; ATCAGG; 1.07886; 0.98547;
Sequence; ABF1 [T00056]; 480; 485; 5.378265; TCTGAT; 1.07886; 0.98547;
Sequence; ABF1 [T00056]; 847; 852; 2.171892; ATCATG; 1.07886; 0.81566;
Sequence; ABF1 [T00056]; 942; 947; 5.274904; ATCAGG; 1.07886; 0.98547;
Sequence; ABF1 [T00056]; 952; 957; 4.654302; ATCAAG; 1.07886; 0.86351;
Sequence; ABF1 [T00056]; 1441; 1446; 5.274904; ATCAGC; 1.07886; 0.98547;
Sequence; ABF1 [T00056]; 1464; 1469; 4.964819; ACTGAT; 0.71924; 0.56436;
Sequence; ABI4 [T05743]; 46; 51; 3.812783; GGTGGG; 1.07886; 1.38257;
Sequence; ABI4 [T05743]; 78; 83; 3.435306; GGTGGC; 0.71924; 0.97142;
Sequence; ABI4 [T05743]; 84; 89; 4.933727; ATCACC; 1.43848; 1.51166;
Sequence; ABI4 [T05743]; 128; 133; 4.933727; ATCACC; 1.43848; 1.51166;
Sequence; ABI4 [T05743]; 308; 313; 4.621089; CACACC; 1.07886; 1.24760;
Sequence; ABI4 [T05743]; 330; 335; 3.812783; GGTGGG; 1.07886; 1.38257;
Sequence; ABI4 [T05743]; 334; 339; 5.227320; GGTGAG; 1.43848; 1.51166;
Sequence; ABI4 [T05743]; 374; 379; 3.812783; CCCACC; 1.07886; 1.38257;
Sequence; ABI4 [T05743]; 377; 382; 3.519189; ACCACC; 0.71924; 0.97142;
Sequence; ABI4 [T05743]; 435; 440; 0.083884; AGCACC; 0.71924; 0.92452;
Sequence; ABI4 [T05743]; 443; 448; 4.327497; AACACC; 1.07886; 1.24760;
Sequence; ABI4 [T05743]; 500; 505; 0.377477; TGCACC; 0.71924; 0.90435;
Sequence; ABI4 [T05743]; 507; 512; 5.227320; CTCACC; 1.43848; 1.51166;
Sequence; ABI4 [T05743]; 609; 614; 3.812783; TCCACC; 1.07886; 1.38257;
Sequence; ABI4 [T05743]; 677; 682; 4.849844; GTCACC; 1.43848; 1.51166;
Sequence; ABI4 [T05743]; 688; 693; 3.812783; TCCACC; 1.07886; 1.38257;
Sequence; ABI4 [T05743]; 704; 709; 5.227320; CTCACC; 1.43848; 1.51166;
Sequence; ABI4 [T05743]; 716; 721; 3.812783; CCCACC; 1.07886; 1.38257;
Sequence; ABI4 [T05743]; 756; 761; 3.812783; CCCACC; 1.07886; 1.38257;
Sequence; ABI4 [T05743]; 810; 815; 3.812783; CCCACC; 1.07886; 1.38257;
Sequence; ABI4 [T05743]; 814; 819; 3.812783; CCCACC; 1.07886; 1.38257;
Sequence; ABI4 [T05743]; 959; 964; 3.812783; CCCACC; 1.07886; 1.38257;
Sequence; ABI4 [T05743]; 968; 973; 3.435306; GCCACC; 0.71924; 0.97142;
Sequence; ABI4 [T05743]; 1029; 1034; 5.227320; CTCACC; 1.43848; 1.51166;
Sequence; ABI4 [T05743]; 1167; 1172; 4.327497; AACACC; 1.07886; 1.24760;
Sequence; ABI4 [T05743]; 1427; 1432; 3.519189; GGTGGT; 0.71924; 0.97142;
Sequence; Pax-9a [T03593]; 43; 49; 11.148154; GAAGGTG; 1.61829; 1.84562;
Sequence; Pax-9a [T03593]; 75; 81; 11.148154; CTAGGTG; 1.61829; 1.84562;
Sequence; Pax-9a [T03593]; 86; 92; 11.636384; CACCAGG; 0.53943; 0.65762;
Sequence; Pax-9a [T03593]; 130; 136; 11.148154; CACCACG; 1.61829; 1.84562;
Sequence; Pax-9a [T03593]; 310; 316; 2.809330; CACCACT; 0.53943; 0.51140;

Sequence; Pax-9a [T03593]; 327; 333; 2.809330; AAAGGTG; 0.53943; 0.51140;
Sequence; Pax-9a [T03593]; 331; 337; 9.743489; GTGGGTG; 0.80914; 1.10780;
Sequence; Pax-9a [T03593]; 376; 382; 11.148154; CACCACC; 1.61829; 1.84562;
Sequence; Pax-9a [T03593]; 379; 385; 11.148154; CACCAAG; 1.61829; 1.84562;
Sequence; Pax-9a [T03593]; 437; 443; 11.148154; CACCTCA; 1.61829; 1.84562;
Sequence; Pax-9a [T03593]; 445; 451; 11.148154; CACCAAC; 1.61829; 1.84562;
Sequence; Pax-9a [T03593]; 502; 508; 2.809330; CACCTCT; 0.53943; 0.51140;
Sequence; Pax-9a [T03593]; 509; 515; 9.743489; CACCCTA; 0.80914; 1.10780;
Sequence; Pax-9a [T03593]; 611; 617; 9.743489; CACCCTC; 0.80914; 1.10780;
Sequence; Pax-9a [T03593]; 679; 685; 9.743489; CACCCTA; 0.80914; 1.10780;
Sequence; Pax-9a [T03593]; 690; 696; 1.404665; CACCCCT; 0.26971; 0.30327;
Sequence; Pax-9a [T03593]; 706; 712; 9.743489; CACCCCC; 0.80914; 1.10780;
Sequence; Pax-9a [T03593]; 718; 724; 2.809330; CACCTCT; 0.53943; 0.51140;
Sequence; Pax-9a [T03593]; 758; 764; 9.743489; CACCCCA; 0.80914; 1.10780;
Sequence; Pax-9a [T03593]; 812; 818; 9.743489; CACCCAC; 0.80914; 1.10780;
Sequence; Pax-9a [T03593]; 816; 822; 11.148154; CACCTTA; 1.61829; 1.84562;
Sequence; Pax-9a [T03593]; 961; 967; 11.636384; CACCTGG; 0.53943; 0.65762;
Sequence; Pax-9a [T03593]; 970; 976; 11.148154; CACCTTA; 1.61829; 1.84562;
Sequence; Pax-9a [T03593]; 1031; 1037; 11.148154; CACCTCA; 1.61829; 1.84562;
Sequence; Pax-9a [T03593]; 1169; 1175; 3.297560; CACCAGT; 0.17981; 0.18542;
Sequence; Pax-9a [T03593]; 1424; 1430; 8.338824; GACGGTG; 0.80914; 1.05040;
Sequence; Pax-9b [T03594]; 43; 49; 11.148154; GAAGGTG; 1.61829; 1.84562;
Sequence; Pax-9b [T03594]; 75; 81; 11.148154; CTAGGTG; 1.61829; 1.84562;
Sequence; Pax-9b [T03594]; 86; 92; 11.636384; CACCAGG; 0.53943; 0.65762;
Sequence; Pax-9b [T03594]; 130; 136; 11.148154; CACCACG; 1.61829; 1.84562;
Sequence; Pax-9b [T03594]; 310; 316; 2.809330; CACCACT; 0.53943; 0.51140;
Sequence; Pax-9b [T03594]; 327; 333; 2.809330; AAAGGTG; 0.53943; 0.51140;
Sequence; Pax-9b [T03594]; 331; 337; 9.743489; GTGGGTG; 0.80914; 1.10780;
Sequence; Pax-9b [T03594]; 376; 382; 11.148154; CACCACC; 1.61829; 1.84562;
Sequence; Pax-9b [T03594]; 379; 385; 11.148154; CACCAAG; 1.61829; 1.84562;
Sequence; Pax-9b [T03594]; 437; 443; 11.148154; CACCTCA; 1.61829; 1.84562;
Sequence; Pax-9b [T03594]; 445; 451; 11.148154; CACCAAC; 1.61829; 1.84562;
Sequence; Pax-9b [T03594]; 502; 508; 2.809330; CACCTCT; 0.53943; 0.51140;
Sequence; Pax-9b [T03594]; 509; 515; 9.743489; CACCCTA; 0.80914; 1.10780;
Sequence; Pax-9b [T03594]; 611; 617; 9.743489; CACCCTC; 0.80914; 1.10780;
Sequence; Pax-9b [T03594]; 679; 685; 9.743489; CACCCTA; 0.80914; 1.10780;
Sequence; Pax-9b [T03594]; 690; 696; 1.404665; CACCCCT; 0.26971; 0.30327;
Sequence; Pax-9b [T03594]; 706; 712; 9.743489; CACCCCC; 0.80914; 1.10780;
Sequence; Pax-9b [T03594]; 718; 724; 2.809330; CACCTCT; 0.53943; 0.51140;
Sequence; Pax-9b [T03594]; 758; 764; 9.743489; CACCCCA; 0.80914; 1.10780;
Sequence; Pax-9b [T03594]; 812; 818; 9.743489; CACCCAC; 0.80914; 1.10780;
Sequence; Pax-9b [T03594]; 816; 822; 11.148154; CACCTTA; 1.61829; 1.84562;
Sequence; Pax-9b [T03594]; 961; 967; 11.636384; CACCTGG; 0.53943; 0.65762;
Sequence; Pax-9b [T03594]; 970; 976; 11.148154; CACCTTA; 1.61829; 1.84562;
Sequence; Pax-9b [T03594]; 1031; 1037; 11.148154; CACCTCA; 1.61829; 1.84562;
Sequence; Pax-9b [T03594]; 1169; 1175; 3.297560; CACCAGT; 0.17981; 0.18542;
Sequence; Pax-9b [T03594]; 1424; 1430; 8.338824; GACGGTG; 0.80914; 1.05040;
Sequence; XPF-1 [T00906]; 85; 93; 10.392427; TCACCAGGG; 0.40457; 0.46901;
Sequence; XPF-1 [T00906]; 792; 800; 10.392427; CACTCAGGG; 0.40457; 0.46901;

Sequence; EBF [T05427]; 85; 95; 13.496832; TCACCAGGGAG; 0.08007; 0.09637;
Sequence; EBF [T05427]; 792; 802; 10.717307; CACTCAGGGAG; 0.05198; 0.06326;
Sequence; Sp3 [T02419]; 89; 99; 12.271607; CAGGGAGGCGT; 0.02529; 0.03500;
Sequence; USF2 [T02115]; 96; 101; 0.537759; GCGTCA; 1.43848; 1.48262;
Sequence; USF2 [T02115]; 168; 173; 1.136475; CTGTCA; 1.43848; 1.43336;
Sequence; USF2 [T02115]; 346; 351; 0.000000; GGGTCA; 0.35962; 0.40224;
Sequence; USF2 [T02115]; 353; 358; 1.735191; TGACAA; 1.07886; 0.92471;
Sequence; USF2 [T02115]; 639; 644; 2.333908; AAGTCA; 1.07886; 0.93191;
Sequence; USF2 [T02115]; 675; 680; 0.537759; GCGTCA; 1.43848; 1.48262;
Sequence; USF2 [T02115]; 700; 705; 1.796148; TGACCT; 0.35962; 0.33891;
Sequence; USF2 [T02115]; 981; 986; 0.000000; TGACCC; 0.35962; 0.40224;
Sequence; USF2 [T02115]; 1014; 1019; 2.333908; TGACTT; 1.07886; 0.93191;
Sequence; USF2 [T02115]; 1317; 1322; 1.136475; CTGTCA; 1.43848; 1.43336;
Sequence; c-Fos [T00123]; 95; 102; 6.315788; GGCGTCAT; 0.22476; 0.21778;
Sequence; c-Fos [T00123]; 167; 174; 12.325220; GCTGTCAG; 0.24724; 0.25756;
Sequence; c-Fos [T00123]; 345; 352; 10.179875; CGGGTCAG; 0.42705; 0.40527;
Sequence; c-Fos [T00123]; 638; 645; 10.717768; CAAGTCAA; 0.35962; 0.32494;
Sequence; c-Fos [T00123]; 674; 681; 7.846178; GGCGTCAC; 0.24724; 0.23146;
Sequence; c-Fos [T00123]; 699; 706; 12.345273; CTGACCTC; 0.24724; 0.25756;
Sequence; c-Fos [T00123]; 980; 987; 4.962405; ATGACCCA; 0.22476; 0.20725;
Sequence; c-Fos [T00123]; 1013; 1020; 8.932314; ATGACTTG; 0.40457; 0.35126;
Sequence; c-Fos [T00123]; 1316; 1323; 12.972410; ACTGTCAT; 0.44952; 0.43374;
Sequence; MafG [T01437]; 98; 102; 0.000000; GTCAT; 1.43848; 1.27082;
Sequence; MafG [T01437]; 980; 984; 0.000000; ATGAC; 1.43848; 1.27082;
Sequence; MafG [T01437]; 1013; 1017; 0.000000; ATGAC; 1.43848; 1.27082;
Sequence; MafG [T01437]; 1319; 1323; 0.000000; GTCAT; 1.43848; 1.27082;
Sequence; YY1 [T00915]; 98; 104; 6.515559; GTCATGG; 0.26971; 0.28104;
Sequence; YY1 [T00915]; 154; 160; 2.781239; GAGATGG; 0.26971; 0.26018;
Sequence; YY1 [T00915]; 187; 193; 8.884219; CGCATGG; 0.53943; 0.61835;
Sequence; YY1 [T00915]; 198; 204; 7.691761; CCATCCC; 0.98895; 1.02677;
Sequence; YY1 [T00915]; 978; 984; 6.515559; CCATGAC; 0.26971; 0.28104;
Sequence; YY1 [T00915]; 1011; 1017; 6.515559; CCATGAC; 0.26971; 0.28104;
Sequence; YY1 [T00915]; 1150; 1156; 5.043792; AGGATGG; 0.71924; 0.67645;
Sequence; Pax-2 [T01823]; 98; 104; 0.591451; GTCATGG; 0.26971; 0.29405;
Sequence; Pax-2 [T01823]; 170; 176; 3.489330; GTCAGTG; 0.44952; 0.43230;
Sequence; Pax-2 [T01823]; 348; 354; 3.489330; GTCAGTG; 0.44952; 0.43230;
Sequence; Pax-2 [T01823]; 350; 356; 2.531143; CAGTGAC; 0.26971; 0.26200;
Sequence; Pax-2 [T01823]; 641; 647; 2.862577; GTCAAGT; 0.98895; 0.99534;
Sequence; Pax-2 [T01823]; 677; 683; 2.904541; GTCACCC; 0.98895; 0.99534;
Sequence; Pax-2 [T01823]; 697; 703; 5.767118; AGCTGAC; 0.71924; 0.63810;
Sequence; Pax-2 [T01823]; 978; 984; 0.591451; CCATGAC; 0.26971; 0.29405;
Sequence; Pax-2 [T01823]; 1011; 1017; 0.591451; CCATGAC; 0.26971; 0.29405;
Sequence; Pax-2 [T01823]; 1319; 1325; 4.482873; GTCATTA; 0.35962; 0.30858;
Sequence; Staf [T02247]; 94; 105; 14.801337; AGGCGTCATGGG; 0.01106; 0.01198;
Sequence; YY1 [T00865]; 101; 106; 3.493585; ATGGGC; 1.07886; 1.04932;
Sequence; YY1 [T00865]; 157; 162; 0.633427; ATGGAA; 0.71924; 0.69290;
Sequence; YY1 [T00865]; 190; 195; 3.071301; ATGGGA; 0.71924; 0.69682;
Sequence; YY1 [T00865]; 196; 201; 2.437874; CCCCAT; 0.35962; 0.41260;
Sequence; YY1 [T00865]; 976; 981; 1.332144; AGCCAT; 0.71924; 0.74491;

Sequence; YY1 [T00865]; 1009; 1014; 1.121002; TGCCAT; 1.07886; 0.96563;
Sequence; YY1 [T00865]; 1153; 1158; 1.332144; ATGGCT; 0.71924; 0.74491;
Sequence; Mitf [T01554]; 95; 107; 13.759864; GGCGTCATGGGCT; 0.01475; 0.01593;
Sequence; Mitf [T01554]; 218; 230; 9.962122; CTCACATGAAGCT; 0.00503; 0.00446;
Sequence; NF-1 (-like proteins) [T00601]; 21; 28; 0.000000; CAGCCAAA; 0.17981; 0.23067;
Sequence; NF-1 (-like proteins) [T00601]; 101; 108; 0.229559; ATGGGCTG; 0.17981; 0.23067;
Sequence; NF-1 (-like proteins) [T00601]; 729; 736; 1.026855; CAGCCAGA; 0.08990; 0.12544;
Sequence; NF-1 (-like proteins) [T00601]; 1219; 1226; 1.026855; CAGCCAGA; 0.08990; 0.12544;
Sequence; C/EBP [T01386]; 79; 84; 9.789909; GTGGCA; 1.43848; 1.38050;
Sequence; C/EBP [T01386]; 114; 119; 8.756040; TCTGCA; 2.15771; 2.11752;
Sequence; C/EBP [T01386]; 116; 121; 8.756040; TGCAGA; 2.15771; 2.11752;
Sequence; C/EBP [T01386]; 185; 190; 13.650994; CACGCA; 2.15771; 2.57378;
Sequence; C/EBP [T01386]; 268; 273; 11.373637; TTGGCA; 1.43848; 1.37637;
Sequence; C/EBP [T01386]; 407; 412; 8.756040; CCTGCA; 2.15771; 2.11752;
Sequence; C/EBP [T01386]; 409; 414; 7.172312; TGCAGC; 2.15771; 2.12541;
Sequence; C/EBP [T01386]; 485; 490; 11.373637; TGCCAA; 1.43848; 1.37637;
Sequence; C/EBP [T01386]; 498; 503; 8.756040; CCTGCA; 2.15771; 2.11752;
Sequence; C/EBP [T01386]; 500; 505; 7.172312; TGCACC; 2.15771; 2.12541;
Sequence; C/EBP [T01386]; 515; 520; 12.067266; ACCGCA; 2.15771; 2.62789;
Sequence; C/EBP [T01386]; 550; 555; 11.373637; TGCTAA; 1.43848; 1.37637;
Sequence; C/EBP [T01386]; 645; 650; 7.172312; AGTGCA; 2.15771; 2.12541;
Sequence; C/EBP [T01386]; 647; 652; 1.583727; TGCAAG; 0.71924; 0.63936;
Sequence; C/EBP [T01386]; 723; 728; 11.373637; CTAGCA; 1.43848; 1.37637;
Sequence; C/EBP [T01386]; 851; 856; 11.373637; TGCCAA; 1.43848; 1.37637;
Sequence; C/EBP [T01386]; 897; 902; 0.000000; GTTGCA; 0.71924; 0.63146;
Sequence; C/EBP [T01386]; 899; 904; 7.172312; TGCACT; 2.15771; 2.12541;
Sequence; C/EBP [T01386]; 926; 931; 4.894955; TGCGAC; 0.71924; 0.74491;
Sequence; C/EBP [T01386]; 1009; 1014; 9.789909; TGCCAT; 1.43848; 1.38050;
Sequence; C/EBP [T01386]; 1082; 1087; 9.789909; ATAGCA; 1.43848; 1.38050;
Sequence; C/EBP [T01386]; 1196; 1201; 9.789909; ATAGCA; 1.43848; 1.38050;
Sequence; C/EBP [T01386]; 1338; 1343; 1.583727; TTTGCA; 0.71924; 0.63936;
Sequence; C/EBP [T01386]; 1340; 1345; 8.756040; TGCACG; 2.15771; 2.11752;
Sequence; C/EBP [T01386]; 1348; 1353; 7.172312; AGTGCA; 2.15771; 2.12541;
Sequence; C/EBP [T01386]; 1350; 1355; 1.583727; TGCAAA; 0.71924; 0.63936;
Sequence; C/EBP [T01386]; 1467; 1472; 7.172312; GATGCA; 2.15771; 2.12541;
Sequence; GATA-1 [T05705]; 117; 123; 5.115149; GCAGATA; 1.07886; 0.89064;
Sequence; GATA-1 [T05705]; 139; 145; 3.069090; GGAGATA; 0.89905; 0.68225;
Sequence; GATA-1 [T05705]; 276; 282; 4.092120; GCTGATA; 1.07886; 0.86619;
Sequence; GATA-1 [T05705]; 390; 396; 4.092120; TCAGATA; 1.07886; 0.86619;
Sequence; GATA-1 [T05705]; 395; 401; 6.138180; TATCAGG; 0.89905; 0.77683;
Sequence; GATA-1 [T05705]; 1306; 1312; 5.115149; TATCCCC; 1.07886; 0.89064;
Sequence; NF-X3 [T01514]; 110; 117; 10.700472; GGATTCTG; 0.26971; 0.32824;
Sequence; NF-X3 [T01514]; 118; 125; 12.359921; CAGATAGG; 1.01143; 1.01838;
Sequence; NF-X3 [T01514]; 235; 242; 5.226274; ATCATCTG; 0.24724; 0.23327;
Sequence; NF-X3 [T01514]; 391; 398; 8.502197; CAGATATC; 0.11238; 0.10781;
Sequence; NF-X3 [T01514]; 476; 483; 3.566824; GGCTTCTG; 0.13486; 0.15374;
Sequence; NF-X3 [T01514]; 527; 534; 14.267296; CAGACTGC; 0.26971; 0.31075;
Sequence; NF-X3 [T01514]; 582; 589; 7.133648; GTCGTCTG; 0.33714; 0.38662;
Sequence; NF-X3 [T01514]; 617; 624; 10.700472; CAGACCCC; 0.26971; 0.32824;

Sequence; NF-X3 [T01514]; 733; 740; 7.133648; CAGAGGAC; 0.33714; 0.38662;
Sequence; NF-X3 [T01514]; 763; 770; 12.069021; CAGACCTC; 0.08990; 0.08911;
Sequence; NF-X3 [T01514]; 1182; 1189; 8.793097; CGCTTCTG; 0.40457; 0.40371;
Sequence; GATA-1 [T00306]; 120; 125; 0.060507; GATAGG; 0.71924; 0.62821;
Sequence; GATA-1 [T00306]; 142; 147; 0.552356; GATAGA; 2.15771; 1.72334;
Sequence; GATA-1 [T00306]; 279; 284; 0.491849; GATAAA; 2.15771; 1.72334;
Sequence; GATA-1 [T00306]; 393; 398; 2.089613; GATATC; 2.15771; 1.70742;
Sequence; GATA-1 [T00306]; 1304; 1309; 1.834316; AGTATC; 2.15771; 1.70742;
Sequence; GATA-3 [T00311]; 119; 126; 1.766593; AGATAGGA; 0.31467; 0.22895;
Sequence; GATA-3 [T00311]; 141; 148; 0.237607; AGATAGAG; 0.11238; 0.08651;
Sequence; GATA-3 [T00311]; 392; 399; 2.128655; AGATATCA; 0.31467; 0.22895;
Sequence; Pax-6 [T00682]; 57; 61; 3.743085; GTCCA; 5.75391; 6.57703;
Sequence; Pax-6 [T00682]; 98; 102; 0.000000; GTCAT; 5.75391; 5.69476;
Sequence; Pax-6 [T00682]; 123; 127; 1.871542; AGGAC; 11.50781; 12.28370;
Sequence; Pax-6 [T00682]; 170; 174; 1.871542; GTCAG; 11.50781; 12.28370;
Sequence; Pax-6 [T00682]; 192; 196; 1.871542; GGGAC; 11.50781; 12.28370;
Sequence; Pax-6 [T00682]; 249; 253; 1.871542; GTCCT; 11.50781; 12.28370;
Sequence; Pax-6 [T00682]; 292; 296; 1.871542; TAGAC; 11.50781; 12.28370;
Sequence; Pax-6 [T00682]; 340; 344; 0.000000; AAGAC; 5.75391; 5.69476;
Sequence; Pax-6 [T00682]; 348; 352; 1.871542; GTCAG; 11.50781; 12.28370;
Sequence; Pax-6 [T00682]; 352; 356; 0.000000; GTGAC; 5.75391; 5.69476;
Sequence; Pax-6 [T00682]; 386; 390; 1.871542; GTCCT; 11.50781; 12.28370;
Sequence; Pax-6 [T00682]; 416; 420; 3.743085; CGGAC; 5.75391; 6.57703;
Sequence; Pax-6 [T00682]; 496; 500; 1.871542; GTCCT; 11.50781; 12.28370;
Sequence; Pax-6 [T00682]; 527; 531; 1.871542; CAGAC; 11.50781; 12.28370;
Sequence; Pax-6 [T00682]; 555; 559; 1.871542; AGGAC; 11.50781; 12.28370;
Sequence; Pax-6 [T00682]; 582; 586; 1.871542; GTCGT; 11.50781; 12.28370;
Sequence; Pax-6 [T00682]; 585; 589; 1.871542; GTCTG; 11.50781; 12.28370;
Sequence; Pax-6 [T00682]; 617; 621; 1.871542; CAGAC; 11.50781; 12.28370;
Sequence; Pax-6 [T00682]; 641; 645; 1.871542; GTCAA; 11.50781; 12.28370;
Sequence; Pax-6 [T00682]; 677; 681; 0.000000; GTCAC; 5.75391; 5.69476;
Sequence; Pax-6 [T00682]; 699; 703; 1.871542; CTGAC; 11.50781; 12.28370;
Sequence; Pax-6 [T00682]; 736; 740; 1.871542; AGGAC; 11.50781; 12.28370;
Sequence; Pax-6 [T00682]; 763; 767; 1.871542; CAGAC; 11.50781; 12.28370;
Sequence; Pax-6 [T00682]; 780; 784; 3.743085; CGGAC; 5.75391; 6.57703;
Sequence; Pax-6 [T00682]; 788; 792; 1.871542; CAGAC; 11.50781; 12.28370;
Sequence; Pax-6 [T00682]; 870; 874; 3.743085; GTCCG; 5.75391; 6.57703;
Sequence; Pax-6 [T00682]; 877; 881; 3.743085; GTCGG; 5.75391; 6.57703;
Sequence; Pax-6 [T00682]; 882; 886; 1.871542; GTCGT; 11.50781; 12.28370;
Sequence; Pax-6 [T00682]; 885; 889; 1.871542; GTCTA; 11.50781; 12.28370;
Sequence; Pax-6 [T00682]; 927; 931; 1.871542; GCGAC; 11.50781; 12.28370;
Sequence; Pax-6 [T00682]; 957; 961; 1.871542; GTCCC; 11.50781; 12.28370;
Sequence; Pax-6 [T00682]; 980; 984; 0.000000; ATGAC; 5.75391; 5.69476;
Sequence; Pax-6 [T00682]; 1000; 1004; 0.000000; AAGAC; 5.75391; 5.69476;
Sequence; Pax-6 [T00682]; 1013; 1017; 0.000000; ATGAC; 5.75391; 5.69476;
Sequence; Pax-6 [T00682]; 1022; 1026; 3.743085; TGGAC; 5.75391; 6.57703;
Sequence; Pax-6 [T00682]; 1060; 1064; 3.743085; GTCGG; 5.75391; 6.57703;
Sequence; Pax-6 [T00682]; 1062; 1066; 3.743085; CGGAC; 5.75391; 6.57703;
Sequence; Pax-6 [T00682]; 1077; 1081; 3.743085; TGGAC; 5.75391; 6.57703;

Sequence; Pax-6 [T00682]; 1093; 1097; 0.000000; AAGAC; 5.75391; 5.69476;
Sequence; Pax-6 [T00682]; 1104; 1108; 1.871542; GTCCT; 11.50781; 12.28370;
Sequence; Pax-6 [T00682]; 1208; 1212; 1.871542; GGGAC; 11.50781; 12.28370;
Sequence; Pax-6 [T00682]; 1211; 1215; 1.871542; ACGAC; 11.50781; 12.28370;
Sequence; Pax-6 [T00682]; 1273; 1277; 1.871542; GTCTA; 11.50781; 12.28370;
Sequence; Pax-6 [T00682]; 1294; 1298; 1.871542; AGGAC; 11.50781; 12.28370;
Sequence; Pax-6 [T00682]; 1319; 1323; 0.000000; GTCAT; 5.75391; 5.69476;
Sequence; Pax-6 [T00682]; 1374; 1378; 1.871542; GGGAC; 11.50781; 12.28370;
Sequence; Pax-6 [T00682]; 1403; 1407; 0.000000; GTCTC; 5.75391; 5.69476;
Sequence; Pax-6 [T00682]; 1422; 1426; 1.871542; AGGAC; 11.50781; 12.28370;
Sequence; Pax-6 [T00682]; 1431; 1435; 0.000000; GTCTC; 5.75391; 5.69476;
Sequence; Pax-6 [T00682]; 1461; 1465; 3.743085; CGGAC; 5.75391; 6.57703;
Sequence; HOXA3 [T00378]; 125; 129; 4.198308; GACAT; 2.87695; 2.77156;
Sequence; HOXA3 [T00378]; 168; 172; 3.338396; CTGTC; 4.31543; 4.00119;
Sequence; HOXA3 [T00378]; 220; 224; 0.859912; CACAT; 1.43848; 1.30860;
Sequence; HOXA3 [T00378]; 247; 251; 5.918131; GTGTC; 1.43848; 1.59675;
Sequence; HOXA3 [T00378]; 294; 298; 3.338396; GACAG; 4.31543; 4.00119;
Sequence; HOXA3 [T00378]; 308; 312; 2.579736; CACAC; 5.75391; 5.24426;
Sequence; HOXA3 [T00378]; 354; 358; 3.338396; GACAA; 4.31543; 4.00119;
Sequence; HOXA3 [T00378]; 422; 426; 1.669198; TTGTT; 2.87695; 2.74789;
Sequence; HOXA3 [T00378]; 443; 447; 4.248934; AACAC; 2.87695; 2.77156;
Sequence; HOXA3 [T00378]; 490; 494; 0.859912; ATGTG; 1.43848; 1.30860;
Sequence; HOXA3 [T00378]; 492; 496; 2.579736; GTGTG; 5.75391; 5.24426;
Sequence; HOXA3 [T00378]; 494; 498; 5.918131; GTGTC; 1.43848; 1.59675;
Sequence; HOXA3 [T00378]; 538; 542; 2.529110; AACAT; 5.75391; 5.24426;
Sequence; HOXA3 [T00378]; 546; 550; 0.859912; CACAT; 1.43848; 1.30860;
Sequence; HOXA3 [T00378]; 577; 581; 0.000000; CACAA; 2.87695; 3.09748;
Sequence; HOXA3 [T00378]; 587; 591; 2.503797; CTGTA; 5.75391; 5.24426;
Sequence; HOXA3 [T00378]; 633; 637; 5.083532; GTGTA; 1.43848; 1.30860;
Sequence; HOXA3 [T00378]; 636; 640; 2.503797; TACAA; 5.75391; 5.24426;
Sequence; HOXA3 [T00378]; 727; 731; 0.000000; CACAG; 2.87695; 3.09748;
Sequence; HOXA3 [T00378]; 786; 790; 1.669198; AACAG; 2.87695; 2.74789;
Sequence; HOXA3 [T00378]; 790; 794; 5.918131; GACAC; 1.43848; 1.59675;
Sequence; HOXA3 [T00378]; 833; 837; 2.579736; CACAC; 5.75391; 5.24426;
Sequence; HOXA3 [T00378]; 835; 839; 0.859912; CACAT; 1.43848; 1.30860;
Sequence; HOXA3 [T00378]; 865; 869; 3.363709; ATGTA; 4.31543; 4.00119;
Sequence; HOXA3 [T00378]; 929; 933; 4.198308; GACAT; 2.87695; 2.77156;
Sequence; HOXA3 [T00378]; 932; 936; 2.529110; ATGTT; 5.75391; 5.24426;
Sequence; HOXA3 [T00378]; 992; 996; 0.000000; CACAA; 2.87695; 3.09748;
Sequence; HOXA3 [T00378]; 1043; 1047; 1.669198; TTGTT; 2.87695; 2.74789;
Sequence; HOXA3 [T00378]; 1064; 1068; 4.198308; GACAT; 2.87695; 2.77156;
Sequence; HOXA3 [T00378]; 1070; 1074; 1.669198; TTGTT; 2.87695; 2.74789;
Sequence; HOXA3 [T00378]; 1079; 1083; 4.198308; GACAT; 2.87695; 2.77156;
Sequence; HOXA3 [T00378]; 1114; 1118; 0.000000; CACAG; 2.87695; 3.09748;
Sequence; HOXA3 [T00378]; 1167; 1171; 4.248934; AACAC; 2.87695; 2.77156;
Sequence; HOXA3 [T00378]; 1187; 1191; 1.669198; CTGTT; 2.87695; 2.74789;
Sequence; HOXA3 [T00378]; 1251; 1255; 1.669198; AACAG; 2.87695; 2.74789;
Sequence; HOXA3 [T00378]; 1317; 1321; 3.338396; CTGTC; 4.31543; 4.00119;
Sequence; HOXA3 [T00378]; 1376; 1380; 3.338396; GACAA; 4.31543; 4.00119;

Sequence; HOXA3 [T00378]; 1386; 1390; 2.529110; ATGTT; 5.75391; 5.24426;
Sequence; HOXA3 [T00378]; 1390; 1394; 5.083532; TACAC; 1.43848; 1.30860;
Sequence; HOXA3 [T00378]; 1438; 1442; 3.363709; TACAT; 4.31543; 4.00119;
Sequence; NHP-1 [T00621]; 124; 130; 14.314808; GGACATC; 1.07886; 0.95892;
Sequence; NHP-1 [T00621]; 167; 173; 11.916538; GCTGTCA; 1.61829; 1.64612;
Sequence; NHP-1 [T00621]; 341; 347; 11.056154; AGACCGG; 0.53943; 0.58998;
Sequence; NHP-1 [T00621]; 345; 351; 6.571346; CGGGTCA; 0.26971; 0.30688;
Sequence; NHP-1 [T00621]; 353; 359; 11.056154; TGACAAG; 0.53943; 0.58998;
Sequence; NHP-1 [T00621]; 383; 389; 9.830000; AAGGTCC; 1.34857; 1.27648;
Sequence; NHP-1 [T00621]; 417; 423; 9.830000; GGACCTT; 1.34857; 1.27648;
Sequence; NHP-1 [T00621]; 618; 624; 11.916538; AGACCCC; 1.61829; 1.64612;
Sequence; NHP-1 [T00621]; 638; 644; 8.969616; CAAGTCA; 0.44952; 0.47157;
Sequence; NHP-1 [T00621]; 700; 706; 0.860385; TGACCTC; 0.26971; 0.24699;
Sequence; NHP-1 [T00621]; 764; 770; 5.345192; AGACCTC; 0.53943; 0.46264;
Sequence; NHP-1 [T00621]; 928; 934; 13.454424; CGACATG; 0.35962; 0.35036;
Sequence; NHP-1 [T00621]; 981; 987; 7.431731; TGACCCA; 0.80914; 0.84026;
Sequence; NHP-1 [T00621]; 1014; 1020; 8.969616; TGACTTG; 0.44952; 0.47157;
Sequence; NHP-1 [T00621]; 1063; 1069; 14.314808; GGACATA; 1.07886; 0.95892;
Sequence; NHP-1 [T00621]; 1078; 1084; 14.314808; GGACATA; 1.07886; 0.95892;
Sequence; NHP-1 [T00621]; 1094; 1100; 11.916538; AGACCAC; 1.61829; 1.64612;
Sequence; NHP-1 [T00621]; 1270; 1276; 14.314808; GAAGTCT; 1.07886; 0.95892;
Sequence; NHP-1 [T00621]; 1316; 1322; 11.916538; ACTGTCA; 1.61829; 1.64612;
Sequence; NHP-1 [T00621]; 1400; 1406; 13.454424; CAAGTCT; 0.35962; 0.35036;
Sequence; NHP-1 [T00621]; 1428; 1434; 11.916538; GTGGTCT; 1.61829; 1.64612;
Sequence; Alfin1 [T04733]; 47; 53; 8.600685; GTGGGCC; 0.80914; 0.91173;
Sequence; Alfin1 [T04733]; 79; 85; 10.351631; GTGGCAT; 0.26971; 0.32563;
Sequence; Alfin1 [T04733]; 129; 135; 0.975568; TCACCAC; 0.35962; 0.43791;
Sequence; Alfin1 [T04733]; 309; 315; 2.701132; ACACCAC; 0.26971; 0.34719;
Sequence; Alfin1 [T04733]; 331; 337; 8.692027; GTGGGTG; 0.80914; 0.91173;
Sequence; Alfin1 [T04733]; 372; 378; 7.450309; CTCCCAC; 0.17981; 0.25145;
Sequence; Alfin1 [T04733]; 375; 381; 0.400380; CCACCAC; 0.35962; 0.49537;
Sequence; Alfin1 [T04733]; 542; 548; 8.025497; TTCCCAC; 0.71924; 0.86378;
Sequence; Alfin1 [T04733]; 573; 579; 8.425878; TTTCCAC; 0.80914; 0.91173;
Sequence; Alfin1 [T04733]; 607; 613; 1.550756; GCTCCAC; 0.17981; 0.23146;
Sequence; Alfin1 [T04733]; 686; 692; 1.550756; GCTCCAC; 0.17981; 0.23146;
Sequence; Alfin1 [T04733]; 714; 720; 0.575188; TCCCCAC; 0.35962; 0.49537;
Sequence; Alfin1 [T04733]; 754; 760; 10.992780; AACCCAC; 0.08990; 0.11732;
Sequence; Alfin1 [T04733]; 808; 814; 0.575188; TCCCCAC; 0.35962; 0.49537;
Sequence; Alfin1 [T04733]; 812; 818; 8.692027; CACCCAC; 0.80914; 0.91173;
Sequence; Alfin1 [T04733]; 829; 835; 10.351631; AGGCCAC; 0.26971; 0.32563;
Sequence; Alfin1 [T04733]; 957; 963; 8.600685; GTCCCAC; 0.80914; 0.91173;
Sequence; Alfin1 [T04733]; 966; 972; 9.201255; GGGCCAC; 0.71924; 0.86067;
Sequence; Alfin1 [T04733]; 982; 988; 9.842403; GACCCAC; 0.89905; 1.00990;
Sequence; Alfin1 [T04733]; 988; 994; 8.050879; CGGCCAC; 0.71924; 0.86378;
Sequence; Alfin1 [T04733]; 1056; 1062; 9.842403; GTGGGTC; 0.89905; 1.00990;
Sequence; Alfin1 [T04733]; 1076; 1082; 8.425878; GTGGACA; 0.80914; 0.91173;
Sequence; Alfin1 [T04733]; 1094; 1100; 10.151442; AGACCAC; 0.89905; 1.00990;
Sequence; Alfin1 [T04733]; 1140; 1146; 8.025497; GTGGGAA; 0.71924; 0.86378;
Sequence; Alfin1 [T04733]; 1428; 1434; 10.151442; GTGGTCT; 0.89905; 1.00990;

Sequence; Vpr [T02399]; 76; 83; 13.930127; TAGGTGGC; 1.21371; 1.48242;
Sequence; Vpr [T02399]; 131; 138; 14.834297; ACCACGGC; 0.80914; 0.91722;
Sequence; Vpr [T02399]; 197; 204; 11.938466; CCCATCCC; 0.40457; 0.60070;
Sequence; Vpr [T02399]; 265; 272; 13.930127; GAGTTGGC; 1.21371; 1.48242;
Sequence; Vpr [T02399]; 311; 318; 14.834297; ACCACTTC; 0.80914; 0.91722;
Sequence; Vpr [T02399]; 374; 381; 11.938466; CCCACCAC; 0.40457; 0.60070;
Sequence; Vpr [T02399]; 688; 695; 9.946805; TCCACCCC; 0.08990; 0.12802;
Sequence; Vpr [T02399]; 716; 723; 11.938466; CCCACCTC; 0.40457; 0.60070;
Sequence; Vpr [T02399]; 756; 763; 5.665926; CCCACCCC; 0.04495; 0.07748;
Sequence; Vpr [T02399]; 761; 768; 11.938466; CCCAGACC; 0.40457; 0.60070;
Sequence; Vpr [T02399]; 810; 817; 11.938466; CCCACCCA; 0.40457; 0.60070;
Sequence; Vpr [T02399]; 831; 838; 7.657587; GCCACACA; 0.40457; 0.56484;
Sequence; Vpr [T02399]; 968; 975; 13.930127; GCCACCTT; 1.21371; 1.48242;
Sequence; Vpr [T02399]; 977; 984; 12.545079; GCCATGAC; 1.21371; 1.40664;
Sequence; Vpr [T02399]; 984; 991; 10.553418; CCCACGGC; 0.40457; 0.54621;
Sequence; Vpr [T02399]; 990; 997; 7.657587; GCCACAAC; 0.40457; 0.56484;
Sequence; Vpr [T02399]; 1010; 1017; 12.545079; GCCATGAC; 1.21371; 1.40664;
Sequence; Vpr [T02399]; 1096; 1103; 8.561758; ACCACGCC; 0.08990; 0.11591;
Sequence; Vpr [T02399]; 1137; 1144; 4.280879; GGCGTGGG; 0.04495; 0.06938;
Sequence; Vpr [T02399]; 1150; 1157; 13.930127; AGGATGGC; 1.21371; 1.48242;
Sequence; Vpr [T02399]; 1177; 1184; 13.930127; GCCAGCGC; 1.21371; 1.48242;
Sequence; Vpr [T02399]; 1246; 1253; 13.930127; GCCAAAAC; 1.21371; 1.48242;
Sequence; Pax-2a [T00678]; 132; 138; 8.014558; CCACGGC; 1.61829; 1.90839;
Sequence; Pax-2a [T00678]; 171; 177; 0.000000; TCAGTGC; 0.80914; 0.99336;
Sequence; Pax-2a [T00678]; 349; 355; 4.007279; TCAGTGA; 0.80914; 0.83306;
Sequence; Pax-2a [T00678]; 630; 636; 8.014558; GCAGTGT; 1.61829; 1.90839;
Sequence; Pax-2a [T00678]; 726; 732; 1.537547; GCACAGC; 0.53943; 0.67185;
Sequence; Pax-2a [T00678]; 985; 991; 8.014558; CCACGGC; 1.61829; 1.90839;
Sequence; Pax-2a [T00678]; 1113; 1119; 5.544826; TCACAGC; 0.53943; 0.56108;
Sequence; Pax-2a [T00678]; 1199; 1205; 4.007279; GCAGTGA; 0.80914; 0.83306;
Sequence; Pax-2a [T00678]; 1341; 1347; 0.000000; GCACGGA; 0.80914; 0.99336;
Sequence; Pax-2a [T00678]; 1425; 1431; 8.014558; ACGGTGG; 1.61829; 1.90839;
Sequence; HES-1 [T01649]; 133; 139; 13.788140; CACGGCG; 0.35962; 0.43871;
Sequence; HES-1 [T01649]; 185; 191; 8.085605; CACGCAT; 1.07886; 1.30875;
Sequence; HES-1 [T01649]; 986; 992; 13.159552; CACGGCC; 0.35962; 0.45175;
Sequence; HES-1 [T01649]; 1052; 1058; 8.399899; CTTCGTG; 0.35962; 0.46490;
Sequence; HES-1 [T01649]; 1098; 1104; 8.399899; CACGCCG; 0.35962; 0.46490;
Sequence; HES-1 [T01649]; 1136; 1142; 7.771311; GGGCGTG; 1.07886; 1.30875;
Sequence; HES-1 [T01649]; 1342; 1348; 13.473845; CACGGAA; 0.71924; 0.76149;
Sequence; TCF-1(P) [T01109]; 141; 150; 14.517477; AGATAGAGAG; 0.10114; 0.09845;
Sequence; TCF-1(P) [T01109]; 730; 739; 11.613981; AGCCAGAGGA; 0.15171; 0.14690;
Sequence; TCF-1(P) [T01109]; 743; 752; 6.746396; GCCTCTTTCT; 0.07586; 0.07332;
Sequence; TCF-1(P) [T01109]; 1201; 1210; 9.824524; AGTGAGAGGG; 0.03652; 0.03042;
Sequence; STAT5A [T04683]; 159; 162; 0.000000; GGAA; 5.75391; 5.61643;
Sequence; STAT5A [T04683]; 425; 428; 0.000000; TTCC; 5.75391; 5.61643;
Sequence; STAT5A [T04683]; 432; 435; 0.000000; GGAA; 5.75391; 5.61643;
Sequence; STAT5A [T04683]; 542; 545; 0.000000; TTCC; 5.75391; 5.61643;
Sequence; STAT5A [T04683]; 574; 577; 0.000000; TTCC; 5.75391; 5.61643;
Sequence; STAT5A [T04683]; 659; 662; 0.000000; TTCC; 5.75391; 5.61643;

Sequence; STAT5A [T04683]; 713; 716; 0.000000; TTCC; 5.75391; 5.61643;
Sequence; STAT5A [T04683]; 1132; 1135; 0.000000; TTCC; 5.75391; 5.61643;
Sequence; STAT5A [T04683]; 1143; 1146; 0.000000; GGAA; 5.75391; 5.61643;
Sequence; STAT5A [T04683]; 1190; 1193; 0.000000; TTCC; 5.75391; 5.61643;
Sequence; STAT5A [T04683]; 1256; 1259; 0.000000; TTCC; 5.75391; 5.61643;
Sequence; STAT5A [T04683]; 1261; 1264; 0.000000; GGAA; 5.75391; 5.61643;
Sequence; STAT5A [T04683]; 1269; 1272; 0.000000; GGAA; 5.75391; 5.61643;
Sequence; STAT5A [T04683]; 1279; 1282; 0.000000; GGAA; 5.75391; 5.61643;
Sequence; STAT5A [T04683]; 1345; 1348; 0.000000; GGAA; 5.75391; 5.61643;
Sequence; STAT5A [T04683]; 1359; 1362; 0.000000; GGAA; 5.75391; 5.61643;
Sequence; IRF-1 [T00423]; 155; 163; 3.228293; AGATGGAAA; 0.08990; 0.07579;
Sequence; IRF-1 [T00423]; 573; 581; 8.452712; TTTCCACAA; 0.08429; 0.08267;
Sequence; IRF-1 [T00423]; 1257; 1265; 7.776815; TCCTGGAAA; 0.13486; 0.12118;
Sequence; IRF-1 [T00423]; 1275; 1283; 4.389325; CTAGGGAAA; 0.08429; 0.07967;
Sequence; IRF-1 [T00423]; 1355; 1363; 5.444136; AGCAGGAAA; 0.24724; 0.21794;
Sequence; POU2F2C [T00665]; 157; 164; 13.840971; ATGGAAAA; 1.01143; 0.77249;
Sequence; POU2F2C [T00665]; 484; 491; 7.988450; ATGCCAAT; 0.11238; 0.07532;
Sequence; POU2F2C [T00665]; 549; 556; 13.840971; ATGCTAAG; 1.01143; 0.77249;
Sequence; POU2F2C [T00665]; 850; 857; 13.840971; ATGCCAAG; 1.01143; 0.77249;
Sequence; POU2F2C [T00665]; 1236; 1243; 13.840971; ATGAGAAA; 1.01143; 0.77249;
Sequence; POU2F2 [T01032]; 157; 164; 13.840971; ATGGAAAA; 1.01143; 0.77249;
Sequence; POU2F2 [T01032]; 484; 491; 7.988450; ATGCCAAT; 0.11238; 0.07532;
Sequence; POU2F2 [T01032]; 549; 556; 13.840971; ATGCTAAG; 1.01143; 0.77249;
Sequence; POU2F2 [T01032]; 850; 857; 13.840971; ATGCCAAG; 1.01143; 0.77249;
Sequence; POU2F2 [T01032]; 1236; 1243; 13.840971; ATGAGAAA; 1.01143; 0.77249;
Sequence; DEF:GLO [T03216]; 157; 164; 3.646766; ATGGAAAA; 0.08990; 0.06763;
Sequence; DEF:GLO [T03216]; 572; 579; 4.862355; TTTTCCAC; 0.02248; 0.01819;
Sequence; DEF:GLO [T03216]; 1158; 1165; 9.885571; TTTTAGAG; 0.35962; 0.27695;
Sequence; DEF:GLO [T03216]; 1222; 1229; 8.211165; CCAGAAAA; 0.26971; 0.22163;
Sequence; DEF:GLO [T03216]; 1237; 1244; 9.885571; TGAGAAAA; 0.35962; 0.27695;
Sequence; DEF:GLO [T03216]; 1277; 1284; 12.316748; AGGGAAAA; 0.35962; 0.29023;
Sequence; DEF:GLO [T03216]; 1299; 1306; 8.211165; TTTTCAGT; 0.26971; 0.22163;
Sequence; DEF:GLO [T03216]; 1337; 1344; 13.532337; TTTTGCAC; 0.08990; 0.07710;
Sequence; IRF-3 [T04673]; 159; 165; 1.209248; GGAAAAG; 0.35962; 0.30826;
Sequence; IRF-3 [T04673]; 571; 577; 1.209248; CTTTTCC; 0.35962; 0.30826;
Sequence; IRF-3 [T04673]; 1261; 1267; 0.238426; GGAAAGT; 0.17981; 0.17385;
Sequence; IRF-3 [T04673]; 1279; 1285; 1.924527; GGAAAAA; 0.17981; 0.17056;
Sequence; IRF-3 [T04673]; 1359; 1365; 2.418497; GGAAATA; 0.08990; 0.06904;
Sequence; MNB1a [T01059]; 160; 165; 1.457401; GAAAAG; 0.71924; 0.58728;
Sequence; MNB1a [T01059]; 571; 576; 1.457401; CTTTTC; 0.71924; 0.58728;
Sequence; MNB1a [T01059]; 1157; 1162; 1.457401; CTTTTA; 0.71924; 0.58728;
Sequence; MNB1a [T01059]; 1226; 1231; 0.000000; AAAAAG; 0.35962; 0.31134;
Sequence; MNB1a [T01059]; 1240; 1245; 1.457401; GAAAAG; 0.71924; 0.58728;
Sequence; MNB1a [T01059]; 1298; 1303; 1.457401; CTTTTC; 0.71924; 0.58728;
Sequence; NF-AT1 [T00550]; 159; 167; 6.201000; GGAAAAGAG; 0.06743; 0.05600;
Sequence; NF-AT1 [T00550]; 569; 577; 6.201000; CTCTTTTCC; 0.06743; 0.05600;
Sequence; NF-AT1 [T00550]; 1261; 1269; 7.819661; GGAAAGTAG; 0.12924; 0.12354;
Sequence; NF-AT1 [T00550]; 1279; 1287; 5.402171; GGAAAAAAA; 0.03371; 0.03050;
Sequence; NF-AT1 [T00550]; 1359; 1367; 7.675131; GGAAATAAA; 0.05619; 0.05203;

Sequence; Dl [T00196]; 160; 168; 10.259304; GAAAAGAGC; 0.14048; 0.11244;
Sequence; Dl [T00196]; 568; 576; 10.609284; CCTCTTTTC; 0.14048; 0.11244;
Sequence; Dl [T00196]; 1225; 1233; 10.043722; GAAAAAGGA; 0.19105; 0.15719;
Sequence; Dl [T00196]; 1240; 1248; 6.438680; GAAAAGGCC; 0.10114; 0.09957;
Sequence; Dl [T00196]; 1280; 1288; 9.739932; GAAAAAAAA; 0.16857; 0.14545;
Sequence; Dl [T00196]; 1295; 1303; 5.591632; GGACTTTTC; 0.07305; 0.05868;
Sequence; TGIF [T04076]; 166; 173; 0.000000; AGCTGTCA; 0.02248; 0.02064;
Sequence; TGIF [T04076]; 353; 360; 1.691794; TGACAAGT; 0.13486; 0.12585;
Sequence; TGIF [T04076]; 1315; 1322; 1.211671; AACTGTCA; 0.13486; 0.12464;
Sequence; PKNOX1 [T04122]; 167; 177; 3.606009; GCTGTCAGTGC; 0.02107; 0.02163;
Sequence; PKNOX1 [T04122]; 1316; 1326; 7.055274; ACTGTCATTAA; 0.02458; 0.02330;
Sequence; YY1 [T04970]; 120; 128; 11.746419; GATAGGACA; 0.40457; 0.44124;
Sequence; YY1 [T04970]; 169; 177; 5.873209; TGTCAGTGC; 0.26971; 0.29727;
Sequence; YY1 [T04970]; 248; 256; 10.283857; TGTCCTCCG; 0.49448; 0.52619;
Sequence; YY1 [T04970]; 289; 297; 14.982425; CAGTAGACA; 0.13486; 0.15713;
Sequence; YY1 [T04970]; 349; 357; 9.552577; TCAGTGACA; 0.31467; 0.32690;
Sequence; YY1 [T04970]; 495; 503; 14.251144; TGTCCTGCA; 0.53943; 0.52277;
Sequence; YY1 [T04970]; 924; 932; 12.633142; CCTGCGACA; 0.13486; 0.13044;
Sequence; YY1 [T04970]; 1059; 1067; 11.746419; GGTCGGACA; 0.40457; 0.44124;
Sequence; YY1 [T04970]; 1318; 1326; 14.251144; TGTCATTAA; 0.53943; 0.52277;
Sequence; YY1 [T04970]; 1371; 1379; 13.076502; TGGGGGACA; 0.26971; 0.26634;
Sequence; E47 [T00207]; 35; 41; 2.197452; GCCAGAG; 0.26971; 0.29060;
Sequence; E47 [T00207]; 58; 64; 5.965250; TCCAGGA; 0.44952; 0.52104;
Sequence; E47 [T00207]; 87; 93; 6.985046; ACCAGGG; 0.44952; 0.53445;
Sequence; E47 [T00207]; 175; 181; 6.034770; TGCTGGG; 0.44952; 0.52104;
Sequence; E47 [T00207]; 731; 737; 2.197452; GCCAGAG; 0.26971; 0.29060;
Sequence; E47 [T00207]; 761; 767; 1.216765; CCCAGAC; 0.26971; 0.32531;
Sequence; E47 [T00207]; 962; 968; 5.136837; ACCTGGG; 0.89905; 1.01788;
Sequence; E47 [T00207]; 1019; 1025; 5.510905; TGCTGGA; 0.62933; 0.73834;
Sequence; E47 [T00207]; 1170; 1176; 6.367045; ACCAGTA; 0.62933; 0.75009;
Sequence; E47 [T00207]; 1177; 1183; 5.832214; GCCAGCG; 0.44952; 0.52104;
Sequence; E47 [T00207]; 1221; 1227; 1.352278; GCCAGAA; 0.08990; 0.10164;
Sequence; E47 [T00207]; 1257; 1263; 5.965250; TCCTGGA; 0.44952; 0.52104;
Sequence; E47 [T00207]; 1368; 1374; 6.489116; TCCTGGG; 0.62933; 0.75009;
Sequence; MZF-1 [T00529]; 177; 183; 2.559408; CTGGGGA; 0.35962; 0.47174;
Sequence; MZF-1 [T00529]; 201; 207; 1.166538; TCCCCTT; 0.35962; 0.39956;
Sequence; MZF-1 [T00529]; 428; 434; 2.672072; CCGGGGA; 0.35962; 0.47174;
Sequence; MZF-1 [T00529]; 660; 666; 2.220412; TCCCCTA; 0.35962; 0.41260;
Sequence; MZF-1 [T00529]; 714; 720; 0.000000; TCCCCAC; 0.35962; 0.47797;
Sequence; MZF-1 [T00529]; 808; 814; 0.000000; TCCCCAC; 0.35962; 0.47797;
Sequence; MZF-1 [T00529]; 909; 915; 2.615740; CGGGGGA; 0.35962; 0.47174;
Sequence; MZF-1 [T00529]; 1308; 1314; 2.107748; TCCCCAA; 0.35962; 0.41260;
Sequence; MZF-1 [T00529]; 1371; 1377; 2.164080; TGGGGGA; 0.35962; 0.41260;
Sequence; CP2 [T00152]; 32; 39; 11.802361; TAGGCCAG; 0.31467; 0.35107;
Sequence; CP2 [T00152]; 55; 62; 11.569437; GAGTCCAG; 0.35962; 0.40020;
Sequence; CP2 [T00152]; 84; 91; 10.317604; ATCACCAG; 0.40457; 0.45792;
Sequence; CP2 [T00152]; 177; 184; 3.679807; CTGGGGAG; 0.04495; 0.05066;
Sequence; CP2 [T00152]; 728; 735; 11.439058; ACAGCCAG; 0.35962; 0.40020;
Sequence; CP2 [T00152]; 758; 765; 4.265877; CACCCCAG; 0.11238; 0.14149;

Sequence; CP2 [T00152]; 964; 971; 13.921238; CTGGGCCA; 0.22476; 0.28945;
Sequence; CP2 [T00152]; 1021; 1028; 4.970520; CTGGACTG; 0.15733; 0.19773;
Sequence; CP2 [T00152]; 1167; 1174; 10.903674; AACACCAG; 0.38210; 0.41886;
Sequence; CP2 [T00152]; 1174; 1181; 7.996460; GTAGCCAG; 0.26971; 0.31142;
Sequence; CP2 [T00152]; 1218; 1225; 12.098950; TCAGCCAG; 0.31467; 0.35107;
Sequence; CP2 [T00152]; 1259; 1266; 3.004522; CTGGAAAG; 0.08990; 0.09744;
Sequence; CP2 [T00152]; 1370; 1377; 14.381214; CTGGGGGA; 0.31467; 0.40735;
Sequence; Pax-5 [T00070]; 96; 102; 4.979591; GCGTCAT; 0.62933; 0.69990;
Sequence; Pax-5 [T00070]; 183; 189; 3.281312; AGCACGC; 0.08990; 0.11549;
Sequence; Pax-5 [T00070]; 675; 681; 2.739140; GCGTCAC; 0.35962; 0.40217;
Sequence; Pax-5 [T00070]; 1000; 1006; 4.979591; AAGACGC; 0.62933; 0.69990;
Sequence; Pax-5 [T00070]; 1096; 1102; 4.322173; ACCACGC; 0.08990; 0.12212;
Sequence; Pax-5 [T00070]; 1138; 1144; 8.803077; GCGTGGG; 0.17981; 0.24771;
Sequence; Pax-5 [T00070]; 1323; 1329; 10.830065; TTAACGC; 0.35962; 0.36274;
Sequence; DBP [T00183]; 172; 178; 5.613135; CAGTGCT; 0.44952; 0.40583;
Sequence; DBP [T00183]; 183; 189; 6.075302; AGCACGC; 0.89905; 0.95254;
Sequence; DBP [T00183]; 212; 218; 6.603631; AGCATCC; 0.80914; 0.82856;
Sequence; DBP [T00183]; 238; 244; 1.056658; ATCTGCT; 0.44952; 0.44545;
Sequence; DBP [T00183]; 287; 293; 6.141464; AGCAGTA; 0.89905; 0.95254;
Sequence; DBP [T00183]; 297; 303; 6.075302; AGCAACC; 0.89905; 0.95254;
Sequence; DBP [T00183]; 306; 312; 0.000000; AGCACAC; 0.35962; 0.36102;
Sequence; DBP [T00183]; 435; 441; 6.075302; AGCACCT; 0.89905; 0.95254;
Sequence; DBP [T00183]; 469; 475; 7.660289; AGCAGCA; 0.35962; 0.38081;
Sequence; DBP [T00183]; 472; 478; 7.131960; AGCAGGC; 0.89905; 0.99545;
Sequence; DBP [T00183]; 529; 535; 5.613135; GACTGCT; 0.44952; 0.40583;
Sequence; DBP [T00183]; 547; 553; 6.603631; ACATGCT; 0.80914; 0.82856;
Sequence; DBP [T00183]; 725; 731; 1.056658; AGCACAG; 0.44952; 0.44545;
Sequence; DBP [T00183]; 823; 829; 7.131960; AGCATGA; 0.89905; 0.99545;
Sequence; DBP [T00183]; 836; 842; 6.603631; ACATGCT; 0.80914; 0.82856;
Sequence; DBP [T00183]; 1016; 1022; 6.075302; ACTTGCT; 0.89905; 0.95254;
Sequence; DBP [T00183]; 1024; 1030; 5.613135; GACTGCT; 0.44952; 0.40583;
Sequence; DBP [T00183]; 1037; 1043; 4.556477; AGCACTT; 0.35962; 0.31996;
Sequence; DBP [T00183]; 1084; 1090; 0.000000; AGCAAAC; 0.35962; 0.36102;
Sequence; DBP [T00183]; 1126; 1132; 6.075302; AGCAACT; 0.89905; 0.95254;
Sequence; DBP [T00183]; 1198; 1204; 6.669793; AGCAGTG; 0.80914; 0.82856;
Sequence; DBP [T00183]; 1355; 1361; 7.660289; AGCAGGA; 0.35962; 0.38081;
Sequence; DBP [T00183]; 1398; 1404; 6.075302; AGCAAGT; 0.89905; 0.95254;
Sequence; DBP [T00183]; 1444; 1450; 6.141464; AGCAGTA; 0.89905; 0.95254;
Sequence; DBP [T00183]; 1452; 1458; 7.131960; AGCAGGC; 0.89905; 0.99545;
Sequence; ZF5 [T02349]; 14; 16; 0.000000; GCG; 23.01562; 28.71437;
Sequence; ZF5 [T02349]; 96; 98; 0.000000; GCG; 23.01562; 28.71437;
Sequence; ZF5 [T02349]; 137; 139; 0.000000; GCG; 23.01562; 28.71437;
Sequence; ZF5 [T02349]; 187; 189; 0.000000; CGC; 23.01562; 28.71437;
Sequence; ZF5 [T02349]; 262; 264; 0.000000; CGC; 23.01562; 28.71437;
Sequence; ZF5 [T02349]; 263; 265; 0.000000; GCG; 23.01562; 28.71437;
Sequence; ZF5 [T02349]; 517; 519; 0.000000; CGC; 23.01562; 28.71437;
Sequence; ZF5 [T02349]; 624; 626; 0.000000; CGC; 23.01562; 28.71437;
Sequence; ZF5 [T02349]; 675; 677; 0.000000; GCG; 23.01562; 28.71437;
Sequence; ZF5 [T02349]; 742; 744; 0.000000; CGC; 23.01562; 28.71437;

Sequence; ZF5 [T02349]; 779; 781; 0.000000; GCG; 23.01562; 28.71437;
Sequence; ZF5 [T02349]; 927; 929; 0.000000; GCG; 23.01562; 28.71437;
Sequence; ZF5 [T02349]; 949; 951; 0.000000; GCG; 23.01562; 28.71437;
Sequence; ZF5 [T02349]; 1004; 1006; 0.000000; CGC; 23.01562; 28.71437;
Sequence; ZF5 [T02349]; 1100; 1102; 0.000000; CGC; 23.01562; 28.71437;
Sequence; ZF5 [T02349]; 1138; 1140; 0.000000; GCG; 23.01562; 28.71437;
Sequence; ZF5 [T02349]; 1181; 1183; 0.000000; GCG; 23.01562; 28.71437;
Sequence; ZF5 [T02349]; 1182; 1184; 0.000000; CGC; 23.01562; 28.71437;
Sequence; ZF5 [T02349]; 1327; 1329; 0.000000; CGC; 23.01562; 28.71437;
Sequence; ZF5 [T02349]; 1328; 1330; 0.000000; GCG; 23.01562; 28.71437;
Sequence; ZF5 [T02349]; 1329; 1331; 0.000000; CGC; 23.01562; 28.71437;
Sequence; ZF5 [T02349]; 1460; 1462; 0.000000; GCG; 23.01562; 28.71437;
Sequence; AhR [T00018]; 184; 192; 7.262416; GCACGCATG; 0.17981; 0.23424;
Sequence; AhR [T00018]; 1097; 1105; 9.831297; CCACGCCGT; 0.08990; 0.12271;
Sequence; AhR [T00018]; 1135; 1143; 8.794940; CGGGCGTGG; 0.14048; 0.18264;
Sequence; AhR:Arnt [T05394]; 185; 193; 5.888384; CACGCATGG; 0.28095; 0.34855;
Sequence; AhR:Arnt [T05394]; 1098; 1106; 4.499480; CACGCCGTC; 0.11238; 0.16806;
Sequence; AhR:Arnt [T05394]; 1134; 1142; 4.623905; CCGGGCGTG; 0.11238; 0.16806;
Sequence; PTF1-beta [T00701]; 186; 195; 3.961287; ACGCATGGGA; 0.06743; 0.06108;
Sequence; PTF1 [T01227]; 186; 196; 6.551229; ACGCATGGGAC; 0.01686; 0.01521;
Sequence; MYBAS1 [T05553]; 179; 183; 1.530821; GGGGA; 2.87695; 3.94670;
Sequence; MYBAS1 [T05553]; 195; 199; 0.000000; ACCCC; 1.43848; 1.91318;
Sequence; MYBAS1 [T05553]; 201; 205; 1.530821; TCCCC; 2.87695; 3.94670;
Sequence; MYBAS1 [T05553]; 430; 434; 1.530821; GGGGA; 2.87695; 3.94670;
Sequence; MYBAS1 [T05553]; 620; 624; 0.000000; ACCCC; 1.43848; 1.91318;
Sequence; MYBAS1 [T05553]; 660; 664; 1.530821; TCCCC; 2.87695; 3.94670;
Sequence; MYBAS1 [T05553]; 672; 676; 1.224657; GGGGC; 1.43848; 2.11265;
Sequence; MYBAS1 [T05553]; 691; 695; 0.000000; ACCCC; 1.43848; 1.91318;
Sequence; MYBAS1 [T05553]; 707; 711; 0.000000; ACCCC; 1.43848; 1.91318;
Sequence; MYBAS1 [T05553]; 708; 712; 1.530821; CCCCC; 2.87695; 3.94670;
Sequence; MYBAS1 [T05553]; 714; 718; 1.530821; TCCCC; 2.87695; 3.94670;
Sequence; MYBAS1 [T05553]; 759; 763; 0.000000; ACCCC; 1.43848; 1.91318;
Sequence; MYBAS1 [T05553]; 808; 812; 1.530821; TCCCC; 2.87695; 3.94670;
Sequence; MYBAS1 [T05553]; 910; 914; 1.530821; GGGGG; 2.87695; 3.94670;
Sequence; MYBAS1 [T05553]; 911; 915; 1.530821; GGGGA; 2.87695; 3.94670;
Sequence; MYBAS1 [T05553]; 1308; 1312; 1.530821; TCCCC; 2.87695; 3.94670;
Sequence; MYBAS1 [T05553]; 1372; 1376; 1.530821; GGGGG; 2.87695; 3.94670;
Sequence; MYBAS1 [T05553]; 1373; 1377; 1.530821; GGGGA; 2.87695; 3.94670;
Sequence; AP-2alpha [T00033]; 178; 186; 1.053726; TGGGGAGCA; 0.13486; 0.15467;
Sequence; AP-2alpha [T00033]; 192; 200; 6.513673; GGGACCCCA; 0.13486; 0.18500;
Sequence; AP-2alpha [T00033]; 671; 679; 4.310562; TGGGGCGTC; 0.13486; 0.18805;
Sequence; AP-2alpha [T00033]; 711; 719; 3.161178; CCTTCCCCA; 0.04495; 0.05164;
Sequence; AP-2alpha [T00033]; 756; 764; 9.674850; CCCACCCCA; 0.08990; 0.12589;
Sequence; AP-2alpha [T00033]; 805; 813; 1.053726; TCATCCCCA; 0.13486; 0.15467;
Sequence; AP-2alpha [T00033]; 1305; 1313; 1.053726; GTATCCCCA; 0.13486; 0.15467;
Sequence; AP-2alpha [T00033]; 1371; 1379; 7.567399; TGGGGGACA; 0.26971; 0.37338;
Sequence; YY1 [T00278]; 98; 104; 8.393145; GTCATGG; 0.89905; 0.92712;
Sequence; YY1 [T00278]; 154; 160; 2.304535; GAGATGG; 0.26971; 0.23304;
Sequence; YY1 [T00278]; 187; 193; 7.540151; CGCATGG; 0.98895; 1.08593;

Sequence; YY1 [T00278]; 198; 204; 6.848277; CCATCCC; 0.35962; 0.37333;
Sequence; YY1 [T00278]; 978; 984; 8.393145; CCATGAC; 0.89905; 0.92712;
Sequence; YY1 [T00278]; 1011; 1017; 8.393145; CCATGAC; 0.89905; 0.92712;
Sequence; YY1 [T00278]; 1150; 1156; 4.543742; AGGATGG; 0.08990; 0.09118;
Sequence; MCB1 [T06035]; 198; 206; 5.955048; CCATCCCCT; 0.13486; 0.13165;
Sequence; MCB1 [T06035]; 213; 221; 6.593716; GCATCCTCA; 0.40457; 0.46243;
Sequence; MCB1 [T06035]; 518; 526; 5.955048; GCATCCTCT; 0.13486; 0.13165;
Sequence; MCB1 [T06035]; 805; 813; 5.516611; TCATCCCCA; 0.20229; 0.22575;
Sequence; MCB1 [T06035]; 1148; 1156; 2.792877; GAAGGATGG; 0.06743; 0.06427;
Sequence; MCB2 [T06036]; 198; 206; 5.955048; CCATCCCCT; 0.13486; 0.13165;
Sequence; MCB2 [T06036]; 213; 221; 6.593716; GCATCCTCA; 0.40457; 0.46243;
Sequence; MCB2 [T06036]; 518; 526; 5.955048; GCATCCTCT; 0.13486; 0.13165;
Sequence; MCB2 [T06036]; 805; 813; 5.516611; TCATCCCCA; 0.20229; 0.22575;
Sequence; MCB2 [T06036]; 1148; 1156; 2.792877; GAAGGATGG; 0.06743; 0.06427;
Sequence; EIIaE-A [T00246]; 6; 12; 4.354651; GAGGGAG; 0.53943; 0.68452;
Sequence; EIIaE-A [T00246]; 10; 16; 0.000000; GAGGGCG; 0.17981; 0.23555;
Sequence; EIIaE-A [T00246]; 89; 95; 5.763567; CAGGGAG; 0.53943; 0.68342;
Sequence; EIIaE-A [T00246]; 178; 184; 14.472869; TGGGGAG; 2.33752; 2.89592;
Sequence; EIIaE-A [T00246]; 201; 207; 13.063953; TCCCCTT; 2.33752; 2.87555;
Sequence; EIIaE-A [T00246]; 372; 378; 13.063953; CTCCCAC; 2.33752; 2.87555;
Sequence; EIIaE-A [T00246]; 425; 431; 14.472869; TTCCCGG; 2.33752; 2.89592;
Sequence; EIIaE-A [T00246]; 509; 515; 10.118218; CACCCTA; 1.61829; 2.07850;
Sequence; EIIaE-A [T00246]; 561; 567; 10.118218; CTCCCGG; 1.61829; 2.07850;
Sequence; EIIaE-A [T00246]; 611; 617; 8.709302; CACCCTC; 1.61829; 2.06363;
Sequence; EIIaE-A [T00246]; 660; 666; 14.472869; TCCCCTA; 2.33752; 2.89592;
Sequence; EIIaE-A [T00246]; 671; 677; 10.118218; TGGGGCG; 1.61829; 2.07850;
Sequence; EIIaE-A [T00246]; 679; 685; 10.118218; CACCCTA; 1.61829; 2.07850;
Sequence; EIIaE-A [T00246]; 708; 714; 8.709302; CCCCCTT; 1.61829; 2.06363;
Sequence; EIIaE-A [T00246]; 796; 802; 5.763567; CAGGGAG; 0.53943; 0.68342;
Sequence; EIIaE-A [T00246]; 908; 914; 14.472869; TCGGGGG; 2.33752; 2.89592;
Sequence; EIIaE-A [T00246]; 910; 916; 13.063953; GGGGGAG; 2.33752; 2.87555;
Sequence; EIIaE-A [T00246]; 1134; 1140; 5.763567; CCGGGCG; 0.53943; 0.68342;
Sequence; EIIaE-A [T00246]; 1206; 1212; 13.063953; GAGGGAC; 2.33752; 2.87555;
Sequence; EIIaE-A [T00246]; 1276; 1282; 10.118218; TAGGGAA; 1.61829; 2.07850;
Sequence; EIIaE-A [T00246]; 1417; 1423; 14.472869; TTGGGAG; 2.33752; 2.89592;
Sequence; FOXO3a [T02938]; 205; 212; 5.687009; CTTATTCA; 0.53943; 0.35590;
Sequence; FOXO3a [T02938]; 243; 250; 12.629773; CTTAGTGT; 1.61829; 1.37569;
Sequence; FOXO3a [T02938]; 549; 556; 13.885529; ATGCTAAG; 0.80914; 0.80434;
Sequence; FOXO3a [T02938]; 595; 602; 8.198520; TGCCTAAG; 0.53943; 0.48667;
Sequence; FOXO3a [T02938]; 817; 824; 12.629773; ACCTTAAG; 1.61829; 1.37569;
Sequence; FOXO3a [T02938]; 819; 826; 6.942764; CTTAAGCA; 1.07886; 0.83236;
Sequence; FOXO3a [T02938]; 918; 925; 11.374018; CTTATACC; 0.80914; 0.58822;
Sequence; FOXO3a [T02938]; 973; 980; 8.198520; CTTAGCCA; 0.53943; 0.48667;
Sequence; USF [T00873]; 217; 225; 13.317287; CCTCACATG; 0.30343; 0.31650;
Sequence; USF [T00873]; 676; 684; 14.866241; CGTCACCCT; 0.65743; 0.64129;
Sequence; muEBP-C2 [T00215]; 220; 225; 0.000000; CACATG; 0.35962; 0.34611;
Sequence; muEBP-C2 [T00215]; 546; 551; 0.000000; CACATG; 0.35962; 0.34611;
Sequence; muEBP-C2 [T00215]; 835; 840; 0.000000; CACATG; 0.35962; 0.34611;
Sequence; USF-1 [T00877]; 216; 226; 8.109390; TCCTCACATGA; 0.02248; 0.02240;

Sequence; USF-1 [T00877]; 542; 552; 1.386985; TTCCCACATGC; 0.01967; 0.01629;
Sequence; USF-1 [T00877]; 831; 841; 11.576853; GCCACACATGC; 0.00983; 0.01050;
Sequence; c-Myc [T00143]; 220; 226; 7.324248; CACATGA; 0.53943; 0.50119;
Sequence; c-Myc [T00143]; 546; 552; 7.324248; CACATGC; 0.53943; 0.50119;
Sequence; c-Myc [T00143]; 642; 648; 7.324248; TCAAGTG; 0.53943; 0.50119;
Sequence; c-Myc [T00143]; 835; 841; 7.324248; CACATGC; 0.53943; 0.50119;
Sequence; c-Myc [T00143]; 961; 967; 3.341592; CACCTGG; 0.08990; 0.10748;
Sequence; LCR-F1 [T01599]; 97; 102; 0.029165; CGTCAT; 1.43848; 1.27082;
Sequence; LCR-F1 [T01599]; 223; 228; 5.375891; ATGAAG; 1.79810; 1.43553;
Sequence; LCR-F1 [T01599]; 231; 236; 5.114259; ACTCAT; 1.07886; 0.91602;
Sequence; LCR-F1 [T01599]; 234; 239; 3.748626; CATCAT; 1.07886; 0.77796;
Sequence; LCR-F1 [T01599]; 803; 808; 5.550882; GTTCAT; 1.79810; 1.43553;
Sequence; LCR-F1 [T01599]; 826; 831; 5.143424; ATGAGG; 1.07886; 0.91602;
Sequence; LCR-F1 [T01599]; 846; 851; 3.923618; GATCAT; 0.35962; 0.28535;
Sequence; LCR-F1 [T01599]; 861; 866; 5.346725; ATTCAT; 1.79810; 1.43553;
Sequence; LCR-F1 [T01599]; 980; 985; 0.204157; ATGACC; 1.43848; 1.27082;
Sequence; LCR-F1 [T01599]; 1013; 1018; 0.000000; ATGACT; 1.43848; 1.27082;
Sequence; LCR-F1 [T01599]; 1146; 1151; 5.375891; ATGAAG; 1.79810; 1.43553;
Sequence; LCR-F1 [T01599]; 1236; 1241; 5.114259; ATGAGA; 1.07886; 0.91602;
Sequence; LCR-F1 [T01599]; 1318; 1323; 0.000000; TGTCAT; 1.43848; 1.27082;
Sequence; Stra13 [T02327]; 216; 226; 14.377309; TCCTCACATGA; 0.11800; 0.11394;
Sequence; Stra13 [T02327]; 219; 229; 14.939254; TCACATGAAGC; 0.09588; 0.08560;
Sequence; STE12 [T00772]; 223; 230; 14.912281; ATGAAGCT; 0.94400; 0.77963;
Sequence; STE12 [T00772]; 771; 778; 14.912281; AGTTTCAG; 0.94400; 0.77963;
Sequence; STE12 [T00772]; 801; 808; 14.912281; AGGTTCAT; 0.94400; 0.77963;
Sequence; STE12 [T00772]; 859; 866; 14.912281; AGATTCAT; 0.94400; 0.77963;
Sequence; Pax-8 [T01828]; 55; 60; 4.910009; GAGTCC; 1.07886; 1.15751;
Sequence; Pax-8 [T01828]; 229; 234; 5.658723; CTACTC; 0.35962; 0.33755;
Sequence; Pax-8 [T01828]; 265; 270; 5.938178; GAGTTG; 0.35962; 0.35928;
Sequence; Pax-8 [T01828]; 461; 466; 5.658723; CTACTC; 0.35962; 0.33755;
Sequence; Pax-8 [T01828]; 791; 796; 7.145650; ACACTC; 0.71924; 0.76151;
Sequence; Pax-8 [T01828]; 875; 880; 2.864172; GAGTCG; 1.07886; 0.98072;
Sequence; Pax-8 [T01828]; 900; 905; 7.145650; GCACTC; 0.71924; 0.76151;
Sequence; Pax-8 [T01828]; 904; 909; 2.235640; TCACTC; 0.35962; 0.33755;
Sequence; Pax-8 [T01828]; 1163; 1168; 2.794550; GAGTAA; 1.07886; 0.98072;
Sequence; Pax-8 [T01828]; 1212; 1217; 2.864172; CGACTC; 1.07886; 0.98072;
Sequence; Pax-8 [T01828]; 1391; 1396; 7.145650; ACACTC; 0.71924; 0.76151;
Sequence; FOXN2 [T04206]; 246; 256; 9.256105; AGTGTCCTCCG; 0.09342; 0.09581;
Sequence; FOXN2 [T04206]; 517; 527; 12.435761; CGCATCCTCTC; 0.08218; 0.08100;
Sequence; FOXN2 [T04206]; 733; 743; 13.336832; CAGAGGACCCG; 0.10957; 0.11488;
Sequence; FOXN2 [T04206]; 1419; 1429; 12.208624; GGGAGGACGGT; 0.05057; 0.05612;
Sequence; E2F-1 [T01542]; 13; 19; 5.331496; GGCGGAG; 0.35962; 0.55731;
Sequence; E2F-1 [T01542]; 136; 142; 5.331496; GGCGGAG; 0.35962; 0.55731;
Sequence; E2F-1 [T01542]; 259; 265; 5.331496; CTCCGCG; 0.35962; 0.55731;
Sequence; E2F-1 [T01542]; 514; 520; 4.630685; TACCGCA; 0.17981; 0.18542;
Sequence; E2F-1 [T01542]; 621; 627; 5.126038; CCCCGCT; 0.17981; 0.26581;
Sequence; E2F-1 [T01542]; 739; 745; 5.944817; ACCCGCC; 0.35962; 0.57368;
Sequence; E2F-1 [T01542]; 778; 784; 7.879274; GGCGGAC; 0.89905; 1.26476;
Sequence; E2F-1 [T01542]; 1459; 1465; 9.608274; AGCGGAC; 0.71924; 0.88364;

Sequence; f(alpha)-f(epsilon) [T00287]; 13; 18; 10.521398; GGCGGA; 5.39429; 7.03435;
Sequence; f(alpha)-f(epsilon) [T00287]; 133; 138; 10.521398; CACGGC; 5.39429; 7.03435;
Sequence; f(alpha)-f(epsilon) [T00287]; 136; 141; 10.521398; GGCGGA; 5.39429; 7.03435;
Sequence; f(alpha)-f(epsilon) [T00287]; 260; 265; 10.521398; TCCGCG; 5.39429; 7.03435;
Sequence; f(alpha)-f(epsilon) [T00287]; 343; 348; 10.521398; ACCGGG; 5.39429; 7.03435;
Sequence; f(alpha)-f(epsilon) [T00287]; 427; 432; 10.521398; CCCGGG; 5.39429; 7.03435;
Sequence; f(alpha)-f(epsilon) [T00287]; 515; 520; 14.400746; ACCGCA; 1.43848; 1.62793;
Sequence; f(alpha)-f(epsilon) [T00287]; 563; 568; 10.521398; CCCGGC; 5.39429; 7.03435;
Sequence; f(alpha)-f(epsilon) [T00287]; 778; 783; 10.521398; GGCGGA; 5.39429; 7.03435;
Sequence; f(alpha)-f(epsilon) [T00287]; 871; 876; 4.800249; TCCGGA; 0.71924; 0.88251;
Sequence; f(alpha)-f(epsilon) [T00287]; 892; 897; 0.920900; GCCGGG; 1.07886; 1.60322;
Sequence; f(alpha)-f(epsilon) [T00287]; 986; 991; 10.521398; CACGGC; 5.39429; 7.03435;
Sequence; f(alpha)-f(epsilon) [T00287]; 1101; 1106; 10.521398; GCCGTC; 5.39429; 7.03435;
Sequence; f(alpha)-f(epsilon) [T00287]; 1133; 1138; 5.721149; TCCGGG; 2.15771; 2.97041;
Sequence; f(alpha)-f(epsilon) [T00287]; 1459; 1464; 10.521398; AGCGGA; 5.39429; 7.03435;
Sequence; NF-1 [T00539]; 23; 27; 0.000000; GCCAA; 1.43848; 1.59675;
Sequence; NF-1 [T00539]; 268; 272; 0.000000; TTGGC; 1.43848; 1.59675;
Sequence; NF-1 [T00539]; 486; 490; 0.000000; GCCAA; 1.43848; 1.59675;
Sequence; NF-1 [T00539]; 852; 856; 0.000000; GCCAA; 1.43848; 1.59675;
Sequence; NF-1 [T00539]; 1246; 1250; 0.000000; GCCAA; 1.43848; 1.59675;
Sequence; C/EBPbeta [T00017]; 267; 274; 9.292018; GTTGGCAA; 0.08990; 0.09038;
Sequence; C/EBPbeta [T00017]; 294; 301; 12.949852; GACAGCAA; 0.58438; 0.69664;
Sequence; C/EBPbeta [T00017]; 594; 601; 4.188507; TTGCCTAA; 0.11238; 0.08563;
Sequence; C/EBPbeta [T00017]; 644; 651; 8.386864; AAGTGCAA; 0.26971; 0.26379;
Sequence; C/EBPbeta [T00017]; 898; 905; 10.378629; TTGCACTC; 0.17981; 0.18936;
Sequence; C/EBPbeta [T00017]; 1008; 1015; 13.404756; TTGCCATG; 0.17981; 0.20197;
Sequence; C/EBPbeta [T00017]; 1018; 1025; 7.797103; TTGCTGGA; 0.26971; 0.24254;
Sequence; C/EBPbeta [T00017]; 1081; 1088; 12.078067; CATAGCAA; 0.38210; 0.40268;
Sequence; C/EBPbeta [T00017]; 1123; 1130; 10.376897; AAGAGCAA; 0.17981; 0.18936;
Sequence; C/EBPbeta [T00017]; 1339; 1346; 11.201177; TTGCACGG; 0.53943; 0.56303;
Sequence; C/EBPbeta [T00017]; 1347; 1354; 8.386864; AAGTGCAA; 0.26971; 0.26379;
Sequence; C/EBPbeta [T00017]; 1395; 1402; 7.215913; TCGAGCAA; 0.44952; 0.41222;
Sequence; HNF-4alpha2 [T02422]; 270; 280; 7.332396; GGCAAAGCTGA; 0.01475; 0.01458;
Sequence; HNF-4alpha1 [T02429]; 270; 280; 7.332396; GGCAAAGCTGA; 0.01475; 0.01458;
Sequence; GATA-1 [T00305]; 276; 283; 1.377780; GCTGATAA; 0.22476; 0.15438;
Sequence; Ncx [T04368]; 206; 208; 0.000000; TTA; 23.01562; 17.57015;
Sequence; Ncx [T04368]; 244; 246; 0.000000; TTA; 23.01562; 17.57015;
Sequence; Ncx [T04368]; 281; 283; 0.000000; TAA; 23.01562; 17.57015;
Sequence; Ncx [T04368]; 537; 539; 0.000000; TAA; 23.01562; 17.57015;
Sequence; Ncx [T04368]; 553; 555; 0.000000; TAA; 23.01562; 17.57015;
Sequence; Ncx [T04368]; 590; 592; 0.000000; TAA; 23.01562; 17.57015;
Sequence; Ncx [T04368]; 599; 601; 0.000000; TAA; 23.01562; 17.57015;
Sequence; Ncx [T04368]; 603; 605; 0.000000; TTA; 23.01562; 17.57015;
Sequence; Ncx [T04368]; 820; 822; 0.000000; TTA; 23.01562; 17.57015;
Sequence; Ncx [T04368]; 821; 823; 0.000000; TAA; 23.01562; 17.57015;
Sequence; Ncx [T04368]; 888; 890; 0.000000; TAA; 23.01562; 17.57015;
Sequence; Ncx [T04368]; 919; 921; 0.000000; TTA; 23.01562; 17.57015;
Sequence; Ncx [T04368]; 974; 976; 0.000000; TTA; 23.01562; 17.57015;
Sequence; Ncx [T04368]; 1073; 1075; 0.000000; TTA; 23.01562; 17.57015;

Sequence; Ncx [T04368]; 1160; 1162; 0.000000; TTA; 23.01562; 17.57015;
Sequence; Ncx [T04368]; 1166; 1168; 0.000000; TAA; 23.01562; 17.57015;
Sequence; Ncx [T04368]; 1323; 1325; 0.000000; TTA; 23.01562; 17.57015;
Sequence; Ncx [T04368]; 1324; 1326; 0.000000; TAA; 23.01562; 17.57015;
Sequence; Ncx [T04368]; 1364; 1366; 0.000000; TAA; 23.01562; 17.57015;
Sequence; Ncx [T04368]; 1381; 1383; 0.000000; TAA; 23.01562; 17.57015;
Sequence; Ncx [T04368]; 1384; 1386; 0.000000; TAA; 23.01562; 17.57015;
Sequence; Ncx [T04368]; 1389; 1391; 0.000000; TTA; 23.01562; 17.57015;
Sequence; Ncx [T04368]; 1449; 1451; 0.000000; TAA; 23.01562; 17.57015;
Sequence; GATA-1 [T00267]; 276; 284; 0.371905; GCTGATAAA; 0.02248; 0.01909;
Sequence; Cdx-1 [T01484]; 205; 208; 2.545394; CTTA; 5.75391; 4.68568;
Sequence; Cdx-1 [T01484]; 243; 246; 2.545394; CTTA; 5.75391; 4.68568;
Sequence; Cdx-1 [T01484]; 281; 284; 0.848465; TAAA; 5.75391; 4.15396;
Sequence; Cdx-1 [T01484]; 537; 540; 1.272697; TAAC; 5.75391; 4.78526;
Sequence; Cdx-1 [T01484]; 553; 556; 2.545394; TAAG; 5.75391; 4.68568;
Sequence; Cdx-1 [T01484]; 590; 593; 1.272697; TAAC; 5.75391; 4.78526;
Sequence; Cdx-1 [T01484]; 599; 602; 2.545394; TAAG; 5.75391; 4.68568;
Sequence; Cdx-1 [T01484]; 602; 605; 1.272697; GTTA; 5.75391; 4.78526;
Sequence; Cdx-1 [T01484]; 819; 822; 2.545394; CTTA; 5.75391; 4.68568;
Sequence; Cdx-1 [T01484]; 821; 824; 2.545394; TAAG; 5.75391; 4.68568;
Sequence; Cdx-1 [T01484]; 888; 891; 0.848465; TAAA; 5.75391; 4.15396;
Sequence; Cdx-1 [T01484]; 918; 921; 2.545394; CTTA; 5.75391; 4.68568;
Sequence; Cdx-1 [T01484]; 973; 976; 2.545394; CTTA; 5.75391; 4.68568;
Sequence; Cdx-1 [T01484]; 1072; 1075; 1.272697; GTTA; 5.75391; 4.78526;
Sequence; Cdx-1 [T01484]; 1159; 1162; 0.848465; TTTA; 5.75391; 4.15396;
Sequence; Cdx-1 [T01484]; 1166; 1169; 1.272697; TAAC; 5.75391; 4.78526;
Sequence; Cdx-1 [T01484]; 1322; 1325; 0.000000; ATTA; 5.75391; 3.94524;
Sequence; Cdx-1 [T01484]; 1324; 1327; 1.272697; TAAC; 5.75391; 4.78526;
Sequence; Cdx-1 [T01484]; 1364; 1367; 0.848465; TAAA; 5.75391; 4.15396;
Sequence; Cdx-1 [T01484]; 1381; 1384; 0.000000; TAAT; 5.75391; 3.94524;
Sequence; Cdx-1 [T01484]; 1384; 1387; 0.000000; TAAT; 5.75391; 3.94524;
Sequence; Cdx-1 [T01484]; 1388; 1391; 1.272697; GTTA; 5.75391; 4.78526;
Sequence; Cdx-1 [T01484]; 1449; 1452; 0.848465; TAAA; 5.75391; 4.15396;
Sequence; HOXD8 [T01426]; 280; 286; 12.536583; ATAAAGG; 0.71924; 0.55466;
Sequence; HOXD8 [T01426]; 916; 922; 11.316162; GGCTTAT; 0.71924; 0.53918;
Sequence; HOXD8 [T01426]; 1363; 1369; 11.316162; ATAAATC; 0.71924; 0.53918;
Sequence; HOXD8 [T01426]; 1380; 1386; 1.220421; ATAATAA; 0.17981; 0.10879;
Sequence; HOXD8 [T01426]; 1383; 1389; 7.544108; ATAATGT; 0.89905; 0.62596;
Sequence; HOXD8 [T01754]; 280; 286; 12.536583; ATAAAGG; 0.71924; 0.55466;
Sequence; HOXD8 [T01754]; 916; 922; 11.316162; GGCTTAT; 0.71924; 0.53918;
Sequence; HOXD8 [T01754]; 1363; 1369; 11.316162; ATAAATC; 0.71924; 0.53918;
Sequence; HOXD8 [T01754]; 1380; 1386; 1.220421; ATAATAA; 0.17981; 0.10879;
Sequence; HOXD8 [T01754]; 1383; 1389; 7.544108; ATAATGT; 0.89905; 0.62596;
Sequence; POU1F1b [T01516]; 280; 287; 10.500194; ATAAAGGA; 0.87657; 0.56586;
Sequence; POU1F1b [T01516]; 1363; 1370; 11.843128; ATAAATCC; 0.29219; 0.21415;
Sequence; POU1F1b [T01516]; 1380; 1387; 7.000129; ATAATAAT; 0.60686; 0.35518;
Sequence; POU1F1b [T01516]; 1383; 1390; 3.500065; ATAATGTT; 0.20229; 0.10875;
Sequence; POU1F1c [T01902]; 280; 287; 10.500194; ATAAAGGA; 0.87657; 0.56586;
Sequence; POU1F1c [T01902]; 1363; 1370; 11.843128; ATAAATCC; 0.29219; 0.21415;

Sequence; POU1F1c [T01902]; 1380; 1387; 7.000129; ATAATAAT; 0.60686; 0.35518;
Sequence; POU1F1c [T01902]; 1383; 1390; 3.500065; ATAATGTT; 0.20229; 0.10875;
Sequence; HNF-3beta [T02513]; 281; 287; 3.504129; TAAAGGA; 0.89905; 0.66321;
Sequence; HNF-3beta [T02513]; 888; 894; 6.557271; TAAAGCC; 0.35962; 0.28520;
Sequence; HNF-3beta [T02513]; 1156; 1162; 4.114758; GCTTTTA; 0.35962; 0.29426;
Sequence; HNF-3beta [T02513]; 1364; 1370; 3.663770; TAAATCC; 0.89905; 0.66321;
Sequence; HNF-3beta [T02513]; 1449; 1455; 5.946643; TAAAGCA; 0.71924; 0.52475;
Sequence; RFX1 [T01673]; 294; 302; 11.490731; GACAGCAAC; 0.12924; 0.13446;
Sequence; RFX1 [T01673]; 897; 905; 13.273591; GTTGCACTC; 0.04495; 0.04570;
Sequence; RFX1 [T01673]; 1123; 1131; 12.452942; AAGAGCAAC; 0.08429; 0.09083;
Sequence; Nkx2-1 [T00856]; 302; 307; 1.818579; CCCAAG; 0.71924; 0.83074;
Sequence; Nkx2-1 [T00856]; 354; 359; 2.762992; GACAAG; 1.07886; 1.10361;
Sequence; Nkx2-1 [T00856]; 363; 368; 3.216595; ACCAAG; 1.07886; 1.10361;
Sequence; Nkx2-1 [T00856]; 380; 385; 3.216595; ACCAAG; 1.07886; 1.10361;
Sequence; Nkx2-1 [T00856]; 421; 426; 3.398453; CTTGTT; 1.07886; 1.05401;
Sequence; Nkx2-1 [T00856]; 577; 582; 2.000437; CACAAG; 0.71924; 0.83074;
Sequence; Nkx2-1 [T00856]; 593; 598; 3.308565; CTTGCC; 1.07886; 1.05401;
Sequence; Nkx2-1 [T00856]; 636; 641; 3.271361; TACAAG; 1.07886; 1.05401;
Sequence; Nkx2-1 [T00856]; 641; 646; 0.762554; GTCAAG; 0.35962; 0.33891;
Sequence; Nkx2-1 [T00856]; 647; 652; 3.816935; TGCAAG; 0.71924; 0.70647;
Sequence; Nkx2-1 [T00856]; 852; 857; 2.581134; GCCAAG; 0.71924; 0.83980;
Sequence; Nkx2-1 [T00856]; 935; 940; 1.270924; TTCAAG; 0.71924; 0.58580;
Sequence; Nkx2-1 [T00856]; 952; 957; 1.398016; ATCAAG; 0.71924; 0.58580;
Sequence; Nkx2-1 [T00856]; 1007; 1012; 3.308565; CTTGCC; 1.07886; 1.05401;
Sequence; Nkx2-1 [T00856]; 1017; 1022; 3.944027; CTTGCT; 0.71924; 0.70647;
Sequence; Nkx2-1 [T00856]; 1398; 1403; 3.944027; AGCAAG; 0.71924; 0.70647;
Sequence; Nkx2-1 [T00856]; 1416; 1421; 1.818579; CTTGGG; 0.71924; 0.83074;
Sequence; DEC2 [T05845]; 220; 229; 6.140450; CACATGAAGC; 0.08429; 0.07676;
Sequence; DEC2 [T05845]; 314; 323; 9.210675; ACTTCAGCTG; 0.21071; 0.18636;
Sequence; DEC2 [T05845]; 318; 327; 9.210675; CAGCTGAAGA; 0.21071; 0.18636;
Sequence; DEC2 [T05845]; 1087; 1096; 12.280901; AAACTGAAGA; 0.44250; 0.38802;
Sequence; DEC2 [T05845]; 1143; 1152; 12.280901; GGAATGAAGG; 0.44250; 0.38802;
Sequence; AP-4 [T00036]; 0; 10; 3.254586; GCAGCTGAGGG; 0.01299; 0.01500;
Sequence; AP-4 [T00036]; 314; 324; 3.601469; ACTTCAGCTGA; 0.01299; 0.01500;
Sequence; AP-4 [T00036]; 317; 327; 3.601469; TCAGCTGAAGA; 0.01299; 0.01500;
Sequence; Spz1 [T04668]; 69; 75; 0.047083; ACCCTCC; 0.35962; 0.52916;
Sequence; Spz1 [T04668]; 195; 201; 14.631618; ACCCCAT; 0.98895; 1.06063;
Sequence; Spz1 [T04668]; 301; 307; 13.451489; ACCCAAG; 0.71924; 0.92452;
Sequence; Spz1 [T04668]; 330; 336; 0.047083; GGTGGGT; 0.35962; 0.52916;
Sequence; Spz1 [T04668]; 343; 349; 14.772866; ACCGGGT; 0.44952; 0.46240;
Sequence; Spz1 [T04668]; 450; 456; 2.910575; ACCCTCA; 0.35962; 0.46931;
Sequence; Spz1 [T04668]; 510; 516; 10.997067; ACCCTAC; 0.71924; 0.97142;
Sequence; Spz1 [T04668]; 558; 564; 0.047083; ACCCTCC; 0.35962; 0.52916;
Sequence; Spz1 [T04668]; 612; 618; 2.910575; ACCCTCA; 0.35962; 0.46931;
Sequence; Spz1 [T04668]; 620; 626; 10.949985; ACCCCGC; 0.71924; 0.97142;
Sequence; Spz1 [T04668]; 680; 686; 13.451489; ACCCTAG; 0.71924; 0.92452;
Sequence; Spz1 [T04668]; 691; 697; 14.725975; ACCCCTA; 0.98895; 1.06063;
Sequence; Spz1 [T04668]; 707; 713; 3.681633; ACCCCCT; 0.26971; 0.30327;
Sequence; Spz1 [T04668]; 739; 745; 0.141249; ACCCGCC; 0.35962; 0.52916;

Sequence; Spz1 [T04668]; 755; 761; 0.047083; ACCCACC; 0.35962; 0.52916;
Sequence; Spz1 [T04668]; 759; 765; 13.404407; ACCCCAG; 0.71924; 0.92452;
Sequence; Spz1 [T04668]; 813; 819; 0.047083; ACCCACC; 0.35962; 0.52916;
Sequence; Spz1 [T04668]; 877; 883; 11.091234; GTCGGGT; 0.71924; 0.97142;
Sequence; Spz1 [T04668]; 892; 898; 11.091234; GCCGGGT; 0.71924; 0.97142;
Sequence; Spz1 [T04668]; 983; 989; 2.501505; ACCCACG; 0.35962; 0.50211;
Sequence; Spz1 [T04668]; 1055; 1061; 2.501505; CGTGGGT; 0.35962; 0.50211;
Sequence; VDR [T00885]; 69; 72; 0.000000; ACCC; 5.75391; 6.94211;
Sequence; VDR [T00885]; 195; 198; 0.000000; ACCC; 5.75391; 6.94211;
Sequence; VDR [T00885]; 301; 304; 0.000000; ACCC; 5.75391; 6.94211;
Sequence; VDR [T00885]; 333; 336; 0.000000; GGGT; 5.75391; 6.94211;
Sequence; VDR [T00885]; 346; 349; 0.000000; GGGT; 5.75391; 6.94211;
Sequence; VDR [T00885]; 450; 453; 0.000000; ACCC; 5.75391; 6.94211;
Sequence; VDR [T00885]; 510; 513; 0.000000; ACCC; 5.75391; 6.94211;
Sequence; VDR [T00885]; 558; 561; 0.000000; ACCC; 5.75391; 6.94211;
Sequence; VDR [T00885]; 612; 615; 0.000000; ACCC; 5.75391; 6.94211;
Sequence; VDR [T00885]; 620; 623; 0.000000; ACCC; 5.75391; 6.94211;
Sequence; VDR [T00885]; 680; 683; 0.000000; ACCC; 5.75391; 6.94211;
Sequence; VDR [T00885]; 691; 694; 0.000000; ACCC; 5.75391; 6.94211;
Sequence; VDR [T00885]; 707; 710; 0.000000; ACCC; 5.75391; 6.94211;
Sequence; VDR [T00885]; 739; 742; 0.000000; ACCC; 5.75391; 6.94211;
Sequence; VDR [T00885]; 755; 758; 0.000000; ACCC; 5.75391; 6.94211;
Sequence; VDR [T00885]; 759; 762; 0.000000; ACCC; 5.75391; 6.94211;
Sequence; VDR [T00885]; 813; 816; 0.000000; ACCC; 5.75391; 6.94211;
Sequence; VDR [T00885]; 880; 883; 0.000000; GGGT; 5.75391; 6.94211;
Sequence; VDR [T00885]; 895; 898; 0.000000; GGGT; 5.75391; 6.94211;
Sequence; VDR [T00885]; 983; 986; 0.000000; ACCC; 5.75391; 6.94211;
Sequence; VDR [T00885]; 1058; 1061; 0.000000; GGGT; 5.75391; 6.94211;
Sequence; E2 [T00205]; 341; 346; 2.893664; AGACCG; 1.79810; 2.13658;
Sequence; E2 [T00205]; 513; 518; 2.881236; CTACCG; 1.79810; 2.13658;
Sequence; E2 [T00205]; 1426; 1431; 2.639357; CGGTGG; 1.43848; 1.68783;
Sequence; GAMYB [T02679]; 342; 348; 10.100104; GACCGGG; 0.44952; 0.49725;
Sequence; GAMYB [T02679]; 514; 520; 7.758495; TACCGCA; 0.35962; 0.43174;
Sequence; GAMYB [T02679]; 1424; 1430; 9.209902; GACGGTG; 0.44952; 0.54538;
Sequence; T3R-alpha [T00841]; 343; 351; 10.328797; ACCGGGTCA; 0.15171; 0.15700;
Sequence; T3R-alpha [T00841]; 700; 708; 0.183218; TGACCTCAC; 0.02248; 0.02082;
Sequence; T3R-alpha [T00841]; 981; 989; 10.863737; TGACCCACG; 0.10676; 0.11802;
Sequence; ER-beta [T04651]; 343; 351; 4.689452; ACCGGGTCA; 0.05619; 0.05877;
Sequence; ER-beta [T04651]; 700; 708; 5.279465; TGACCTCAC; 0.10114; 0.09507;
Sequence; ER-beta [T04651]; 981; 989; 11.077188; TGACCCACG; 0.04495; 0.04680;
Sequence; RAR-beta [T00721]; 344; 351; 3.187872; CCGGGTCA; 0.15733; 0.18773;
Sequence; RAR-beta [T00721]; 700; 707; 0.169238; TGACCTCA; 0.08990; 0.08770;
Sequence; RAR-beta [T00721]; 981; 988; 3.839955; TGACCCAC; 0.04495; 0.05102;
Sequence; PPAR-alpha [T00694]; 345; 351; 0.294918; CGGGTCA; 0.17981; 0.20213;
Sequence; PPAR-alpha [T00694]; 700; 706; 0.778616; TGACCTC; 0.35962; 0.33891;
Sequence; PPAR-alpha [T00694]; 981; 987; 0.344071; TGACCCA; 0.17981; 0.20213;
Sequence; ER-alpha [T00261]; 345; 352; 2.461283; CGGGTCAG; 0.13486; 0.15610;
Sequence; ER-alpha [T00261]; 699; 706; 1.269456; CTGACCTC; 0.29219; 0.29178;
Sequence; ER-alpha [T00261]; 980; 987; 2.876596; ATGACCCA; 0.31467; 0.33914;

Sequence; USF2b [T02377]; 98; 105; 6.942764; GTCATGGG; 1.07886; 1.02604;
Sequence; USF2b [T02377]; 170; 177; 12.629773; GTCAGTGC; 1.61829; 1.54204;
Sequence; USF2b [T02377]; 348; 355; 12.629773; GTCAGTGA; 1.61829; 1.54204;
Sequence; USF2b [T02377]; 349; 356; 12.629773; TCAGTGAC; 1.61829; 1.54204;
Sequence; USF2b [T02377]; 641; 648; 1.255756; GTCAAGTG; 0.17981; 0.17200;
Sequence; USF2b [T02377]; 677; 684; 11.374018; GTCACCCT; 0.80914; 0.90160;
Sequence; USF2b [T02377]; 696; 703; 5.687009; TAGCTGAC; 0.53943; 0.59991;
Sequence; USF2b [T02377]; 977; 984; 12.629773; GCCATGAC; 1.61829; 1.54204;
Sequence; USF2b [T02377]; 1010; 1017; 12.629773; GCCATGAC; 1.61829; 1.54204;
Sequence; USF2b [T02377]; 1319; 1326; 13.885529; GTCATTAA; 0.80914; 0.65935;
Sequence; c-Jun [T00131]; 96; 101; 2.036051; GCGTCA; 0.71924; 0.74491;
Sequence; c-Jun [T00131]; 168; 173; 4.581115; CTGTCA; 0.71924; 0.73771;
Sequence; c-Jun [T00131]; 346; 351; 2.545064; GGGTCA; 1.43848; 1.43336;
Sequence; c-Jun [T00131]; 353; 358; 3.054077; TGACAA; 1.43848; 1.27082;
Sequence; c-Jun [T00131]; 639; 644; 0.509013; AAGTCA; 0.71924; 0.58580;
Sequence; c-Jun [T00131]; 675; 680; 2.036051; GCGTCA; 0.71924; 0.74491;
Sequence; c-Jun [T00131]; 700; 705; 3.054077; TGACCT; 1.43848; 1.27082;
Sequence; c-Jun [T00131]; 981; 986; 2.545064; TGACCC; 1.43848; 1.43336;
Sequence; c-Jun [T00131]; 1014; 1019; 0.509013; TGACTT; 0.71924; 0.58580;
Sequence; c-Jun [T00131]; 1317; 1322; 4.581115; CTGTCA; 0.71924; 0.73771;
Sequence; JunB [T00436]; 96; 101; 2.941176; GCGTCA; 2.15771; 2.06929;
Sequence; JunB [T00436]; 168; 173; 4.411765; CTGTCA; 1.43848; 1.48949;
Sequence; JunB [T00436]; 346; 351; 4.411765; GGGTCA; 1.43848; 1.48949;
Sequence; JunB [T00436]; 353; 358; 2.941176; TGACAA; 2.15771; 2.06929;
Sequence; JunB [T00436]; 639; 644; 0.000000; AAGTCA; 0.35962; 0.30045;
Sequence; JunB [T00436]; 675; 680; 2.941176; GCGTCA; 2.15771; 2.06929;
Sequence; JunB [T00436]; 700; 705; 2.941176; TGACCT; 2.15771; 2.06929;
Sequence; JunB [T00436]; 981; 986; 4.411765; TGACCC; 1.43848; 1.48949;
Sequence; JunB [T00436]; 1014; 1019; 0.000000; TGACTT; 0.35962; 0.30045;
Sequence; JunB [T00436]; 1317; 1322; 4.411765; CTGTCA; 1.43848; 1.48949;
Sequence; c-Fos [T00124]; 95; 101; 3.691530; GGCGTCA; 0.44952; 0.40638;
Sequence; c-Fos [T00124]; 167; 173; 8.252728; GCTGTCA; 0.26971; 0.29405;
Sequence; c-Fos [T00124]; 345; 351; 7.383060; CGGGTCA; 0.44952; 0.45359;
Sequence; c-Fos [T00124]; 353; 359; 9.860665; TGACAAG; 0.44952; 0.44939;
Sequence; c-Fos [T00124]; 638; 644; 6.684522; CAAGTCA; 0.53943; 0.52547;
Sequence; c-Fos [T00124]; 674; 680; 3.691530; GGCGTCA; 0.44952; 0.40638;
Sequence; c-Fos [T00124]; 700; 706; 6.270644; TGACCTC; 0.53943; 0.52547;
Sequence; c-Fos [T00124]; 981; 987; 2.185101; TGACCCA; 0.17981; 0.17729;
Sequence; c-Fos [T00124]; 1014; 1020; 6.684522; TGACTTG; 0.53943; 0.52547;
Sequence; c-Fos [T00124]; 1316; 1322; 10.201962; ACTGTCA; 0.44952; 0.44939;
Sequence; c-Jun [T00132]; 95; 101; 2.439359; GGCGTCA; 0.17981; 0.18439;
Sequence; c-Jun [T00132]; 167; 173; 7.786713; GCTGTCA; 0.53943; 0.48490;
Sequence; c-Jun [T00132]; 345; 351; 4.522753; CGGGTCA; 0.62933; 0.60949;
Sequence; c-Jun [T00132]; 353; 359; 8.787043; TGACAAG; 0.26971; 0.26556;
Sequence; c-Jun [T00132]; 638; 644; 5.944049; CAAGTCA; 0.35962; 0.32237;
Sequence; c-Jun [T00132]; 674; 680; 2.439359; GGCGTCA; 0.17981; 0.18439;
Sequence; c-Jun [T00132]; 700; 706; 4.876153; TGACCTC; 0.35962; 0.33117;
Sequence; c-Jun [T00132]; 981; 987; 0.609299; TGACCCA; 0.08990; 0.09465;
Sequence; c-Jun [T00132]; 1014; 1020; 5.944049; TGACTTG; 0.35962; 0.32237;

Sequence; c-Jun [T00132]; 1316; 1322; 8.904842; ACTGTCA; 0.26971; 0.26556;
Sequence; c-Jun [T00133]; 95; 101; 4.818265; GGCGTCA; 0.35962; 0.35418;
Sequence; c-Jun [T00133]; 167; 173; 8.792045; GCTGTCA; 0.35962; 0.32651;
Sequence; c-Jun [T00133]; 345; 351; 7.295288; CGGGTCA; 0.44952; 0.44043;
Sequence; c-Jun [T00133]; 353; 359; 9.869451; TGACAAG; 0.17981; 0.15622;
Sequence; c-Jun [T00133]; 638; 644; 6.451291; CAAGTCA; 0.53943; 0.55664;
Sequence; c-Jun [T00133]; 674; 680; 4.818265; GGCGTCA; 0.35962; 0.35418;
Sequence; c-Jun [T00133]; 700; 706; 7.315067; TGACCTC; 0.44952; 0.44043;
Sequence; c-Jun [T00133]; 981; 987; 2.672639; TGACCCA; 0.08990; 0.09465;
Sequence; c-Jun [T00133]; 1014; 1020; 6.451291; TGACTTG; 0.53943; 0.55664;
Sequence; c-Jun [T00133]; 1316; 1322; 9.796968; ACTGTCA; 0.17981; 0.15622;
Sequence; JunD [T00437]; 95; 101; 1.885328; GGCGTCA; 0.71924; 0.71615;
Sequence; JunD [T00437]; 167; 173; 4.797873; GCTGTCA; 0.17981; 0.19940;
Sequence; JunD [T00437]; 345; 351; 7.034347; CGGGTCA; 1.07886; 1.05767;
Sequence; JunD [T00437]; 353; 359; 8.807055; TGACAAG; 0.53943; 0.49120;
Sequence; JunD [T00437]; 638; 644; 6.527383; CAAGTCA; 0.35962; 0.32660;
Sequence; JunD [T00437]; 674; 680; 1.885328; GGCGTCA; 0.71924; 0.71615;
Sequence; JunD [T00437]; 700; 706; 2.138809; TGACCTC; 0.71924; 0.71615;
Sequence; JunD [T00437]; 981; 987; 0.506964; TGACCCA; 0.53943; 0.48879;
Sequence; JunD [T00437]; 1014; 1020; 6.527383; TGACTTG; 0.35962; 0.32660;
Sequence; JunD [T00437]; 1316; 1322; 9.693411; ACTGTCA; 0.53943; 0.57106;
Sequence; SRY [T00997]; 353; 359; 6.224664; TGACAAG; 0.89905; 0.89209;
Sequence; SRY [T00997]; 421; 427; 2.154058; CTTGTTC; 0.53943; 0.52083;
Sequence; SRY [T00997]; 576; 582; 6.723683; CCACAAG; 0.71924; 0.84437;
Sequence; SRY [T00997]; 635; 641; 5.698344; GTACAAG; 0.53943; 0.44584;
Sequence; SRY [T00997]; 991; 997; 6.723683; CCACAAC; 0.71924; 0.84437;
Sequence; SRY [T00997]; 1042; 1048; 0.656330; TTTGTTG; 0.35962; 0.33361;
Sequence; SRY [T00997]; 1069; 1075; 0.045365; ATTGTTA; 0.35962; 0.28529;
Sequence; SRY [T00997]; 1375; 1381; 4.751086; GGACAAT; 0.71924; 0.72208;
Sequence; c-Fos [T00122]; 94; 101; 4.470965; AGGCGTCA; 0.13486; 0.12270;
Sequence; c-Fos [T00122]; 166; 173; 13.129597; AGCTGTCA; 0.35962; 0.31933;
Sequence; c-Fos [T00122]; 344; 351; 7.945733; CCGGGTCA; 0.58438; 0.58180;
Sequence; c-Fos [T00122]; 353; 360; 9.439499; TGACAAGT; 0.26971; 0.27282;
Sequence; c-Fos [T00122]; 637; 644; 4.258138; ACAAGTCA; 0.13486; 0.12270;
Sequence; c-Fos [T00122]; 673; 680; 5.143462; GGGCGTCA; 0.22476; 0.20999;
Sequence; c-Fos [T00122]; 700; 707; 14.474589; TGACCTCA; 0.35962; 0.33998;
Sequence; c-Fos [T00122]; 981; 988; 7.273237; TGACCCAC; 0.13486; 0.14148;
Sequence; c-Fos [T00122]; 1014; 1021; 4.930634; TGACTTGC; 0.22476; 0.20999;
Sequence; c-Fos [T00122]; 1315; 1322; 13.129597; AACTGTCA; 0.35962; 0.31933;
Sequence; TMF [T00835]; 357; 363; 9.356185; AAGTATA; 1.43848; 1.06331;
Sequence; TMF [T00835]; 360; 366; 8.053378; TATACCA; 0.71924; 0.49015;
Sequence; TMF [T00835]; 917; 923; 2.605613; GCTTATA; 0.35962; 0.24077;
Sequence; TMF [T00835]; 920; 926; 8.053378; TATACCT; 0.71924; 0.49015;
Sequence; MEDEA (MED) [T04379]; 367; 377; 11.406384; AGGAGCTCCCA; 0.03793; 0.05565;
Sequence; MEDEA (MED) [T04379]; 605; 615; 14.432816; AGGCTCCACCC; 0.17068; 0.27750;
Sequence; CAC-binding protein [T00076]; 46; 53; 2.152461; GGTGGGCC; 0.13486; 0.20021;
Sequence; CAC-binding protein [T00076]; 78; 85; 4.590575; GGTGGCAT; 0.80914; 1.00172;
Sequence; CAC-binding protein [T00076]; 330; 337; 2.438115; GGTGGGTG; 0.40457; 0.56440;
Sequence; CAC-binding protein [T00076]; 372; 379; 2.438115; CTCCCACC; 0.40457; 0.56440;

Sequence; CAC-binding protein [T00076]; 375; 382; 2.438115; CCACCACC; 0.40457; 0.56440;
Sequence; CAC-binding protein [T00076]; 607; 614; 2.152461; GCTCCACC; 0.13486; 0.20021;
Sequence; CAC-binding protein [T00076]; 686; 693; 2.152461; GCTCCACC; 0.13486; 0.20021;
Sequence; CAC-binding protein [T00076]; 714; 721; 0.285654; TCCCCACC; 0.06743; 0.10859;
Sequence; CAC-binding protein [T00076]; 754; 761; 2.438115; AACCCACC; 0.40457; 0.56440;
Sequence; CAC-binding protein [T00076]; 808; 815; 0.285654; TCCCCACC; 0.06743; 0.10859;
Sequence; CAC-binding protein [T00076]; 812; 819; 2.438115; CACCCACC; 0.40457; 0.56440;
Sequence; CAC-binding protein [T00076]; 957; 964; 2.152461; GTCCCACC; 0.13486; 0.20021;
Sequence; CAC-binding protein [T00076]; 966; 973; 4.304922; GGGCCACC; 0.80914; 1.00172;
Sequence; CAC-binding protein [T00076]; 1427; 1434; 4.590575; GGTGGTCT; 0.80914; 1.00172;
Sequence; Yi [T00913]; 42; 51; 13.915856; AGAAGGTGGG; 0.35400; 0.52601;
Sequence; Yi [T00913]; 374; 383; 14.174757; CCCACCACCA; 0.35400; 0.52601;
Sequence; Yi [T00913]; 740; 749; 12.218299; CCCGCCTCTT; 0.07586; 0.09781;
Sequence; Yi [T00913]; 810; 819; 11.000294; CCCACCCACC; 0.22757; 0.34587;
Sequence; Yi [T00913]; 984; 993; 11.000294; CCCACGGCCA; 0.22757; 0.34587;
Sequence; Yi [T00913]; 1051; 1060; 12.477200; CCTTCGTGGG; 0.18964; 0.28644;
Sequence; Yi [T00913]; 1135; 1144; 11.000294; CGGGCGTGGG; 0.22757; 0.34587;
Sequence; Yi [T00913]; 1201; 1210; 12.697851; AGTGAGAGGG; 0.18964; 0.28644;
Sequence; Ik-1 [T02702]; 373; 385; 11.871492; TCCCACCACCAAG; 0.04748; 0.05439;
Sequence; HNF-4alpha [T05287]; 377; 388; 14.826332; ACCACCAAGGTC; 0.04425; 0.04264;
Sequence; FXR:RXR-alpha [T05318]; 379; 390; 13.283396; CACCAAGGTCCT; 0.05347; 0.05066;
Sequence; TFIIB [T00818]; 3; 10; 11.657154; GCTGAGGG; 0.58438; 0.69877;
Sequence; TFIIB [T00818]; 52; 59; 10.522345; CCTGAGTC; 0.58438; 0.67115;
Sequence; TFIIB [T00818]; 105; 112; 10.522345; GCTGAGGA; 0.58438; 0.67115;
Sequence; TFIIB [T00818]; 151; 158; 4.642258; CCTGAGAT; 0.13486; 0.14072;
Sequence; TFIIB [T00818]; 387; 394; 3.507448; TCCTCAGA; 0.13486; 0.13777;
Sequence; TFIIB [T00818]; 451; 458; 4.642258; CCCTCAGA; 0.13486; 0.14072;
Sequence; TFIIB [T00818]; 523; 530; 1.134810; CTCTCAGA; 0.04495; 0.03873;
Sequence; TFIIB [T00818]; 613; 620; 4.642258; CCCTCAGA; 0.13486; 0.14072;
Sequence; TFIIB [T00818]; 759; 766; 11.657154; ACCCCAGA; 0.58438; 0.69877;
Sequence; TFIIB [T00818]; 766; 773; 11.657154; ACCTCAGT; 0.58438; 0.69877;
Sequence; TFIIB [T00818]; 792; 799; 11.657154; CACTCAGG; 0.58438; 0.69877;
Sequence; TFIIB [T00818]; 924; 931; 7.014897; CCTGCGAC; 0.40457; 0.41947;
Sequence; TFIIB [T00818]; 1032; 1039; 11.657154; ACCTCAGC; 0.58438; 0.69877;
Sequence; TFIIB [T00818]; 1112; 1119; 14.029794; TTCACAGC; 0.80914; 0.92129;
Sequence; TFIIB [T00818]; 1215; 1222; 8.149706; CTCTCAGC; 0.40457; 0.43291;
Sequence; GATA-2 [T01302]; 120; 127; 10.162762; GATAGGAC; 0.26971; 0.23543;
Sequence; GATA-2 [T01302]; 142; 149; 14.323298; GATAGAGA; 0.40457; 0.35230;
Sequence; GATA-2 [T01302]; 279; 286; 5.339574; GATAAAGG; 0.26971; 0.21396;
Sequence; GATA-2 [T01302]; 391; 398; 12.627873; CAGATATC; 0.53943; 0.44430;
Sequence; GATA-2 [T01302]; 393; 400; 7.288299; GATATCAG; 0.08990; 0.07334;
Sequence; Tal-1 [T01799]; 398; 406; 8.925647; CAGGTTCTA; 0.25286; 0.26359;
Sequence; Tal-1 [T01799]; 402; 410; 10.050277; TTCTACCTG; 0.20229; 0.20821;
Sequence; Tal-1 [T01799]; 919; 927; 10.050277; TTATACCTG; 0.20229; 0.20821;
Sequence; Tal-1 [T01799]; 958; 966; 4.498518; TCCCACCTG; 0.06743; 0.07739;
Sequence; AREB6 [T00625]; 398; 405; 7.585013; CAGGTTCT; 0.11238; 0.10498;
Sequence; AREB6 [T00625]; 403; 410; 4.838811; TCTACCTG; 0.04495; 0.04544;
Sequence; AREB6 [T00625]; 920; 927; 4.505247; TATACCTG; 0.13486; 0.12465;
Sequence; AREB6 [T00625]; 959; 966; 0.664330; CCCACCTG; 0.06743; 0.08024;

Sequence; DSXF [T00955]; 72; 78; 14.472869; CTCCTAG; 2.33752; 2.18074;
Sequence; DSXF [T00955]; 404; 410; 14.472869; CTACCTG; 2.33752; 2.18074;
Sequence; DSXF [T00955]; 536; 542; 13.063953; CTAACAT; 2.33752; 1.86489;
Sequence; DSXF [T00955]; 598; 604; 13.063953; CTAAGTT; 2.33752; 1.86489;
Sequence; DSXF [T00955]; 664; 670; 8.709302; CTACGTT; 1.61829; 1.28497;
Sequence; DSXF [T00955]; 864; 870; 5.763567; CATGTAG; 0.53943; 0.49210;
Sequence; DSXF [T00955]; 887; 893; 14.472869; CTAAAGC; 2.33752; 2.18074;
Sequence; DSXF [T00955]; 1070; 1076; 13.063953; TTGTTAG; 2.33752; 1.86489;
Sequence; DSXF [T00955]; 1119; 1125; 10.118218; CTAGAAG; 1.61829; 1.50261;
Sequence; DSXF [T00955]; 1157; 1163; 5.763567; CTTTTAG; 0.53943; 0.49210;
Sequence; DSXF [T00955]; 1263; 1269; 13.063953; AAAGTAG; 2.33752; 1.86489;
Sequence; DSXM [T00956]; 72; 78; 14.472869; CTCCTAG; 2.33752; 2.18074;
Sequence; DSXM [T00956]; 404; 410; 14.472869; CTACCTG; 2.33752; 2.18074;
Sequence; DSXM [T00956]; 536; 542; 13.063953; CTAACAT; 2.33752; 1.86489;
Sequence; DSXM [T00956]; 598; 604; 13.063953; CTAAGTT; 2.33752; 1.86489;
Sequence; DSXM [T00956]; 664; 670; 8.709302; CTACGTT; 1.61829; 1.28497;
Sequence; DSXM [T00956]; 864; 870; 5.763567; CATGTAG; 0.53943; 0.49210;
Sequence; DSXM [T00956]; 887; 893; 14.472869; CTAAAGC; 2.33752; 2.18074;
Sequence; DSXM [T00956]; 1070; 1076; 13.063953; TTGTTAG; 2.33752; 1.86489;
Sequence; DSXM [T00956]; 1119; 1125; 10.118218; CTAGAAG; 1.61829; 1.50261;
Sequence; DSXM [T00956]; 1157; 1163; 5.763567; CTTTTAG; 0.53943; 0.49210;
Sequence; DSXM [T00956]; 1263; 1269; 13.063953; AAAGTAG; 2.33752; 1.86489;
Sequence; LVc [T00478]; 407; 411; 0.000000; CCTGC; 1.43848; 1.75385;
Sequence; LVc [T00478]; 473; 477; 0.000000; GCAGG; 1.43848; 1.75385;
Sequence; LVc [T00478]; 498; 502; 0.000000; CCTGC; 1.43848; 1.75385;
Sequence; LVc [T00478]; 924; 928; 0.000000; CCTGC; 1.43848; 1.75385;
Sequence; LVc [T00478]; 1356; 1360; 0.000000; GCAGG; 1.43848; 1.75385;
Sequence; LVc [T00478]; 1453; 1457; 0.000000; GCAGG; 1.43848; 1.75385;
Sequence; NF-1/L [T00599]; 408; 420; 10.924665; CTGCAGCTCGGAC; 0.01896; 0.02130;
Sequence; En-1 [T02016]; 355; 360; 2.144000; ACAAGT; 1.07886; 1.03772;
Sequence; En-1 [T02016]; 420; 425; 3.789256; CCTTGT; 0.35962; 0.36756;
Sequence; En-1 [T02016]; 578; 583; 2.144000; ACAAGT; 1.07886; 1.03772;
Sequence; En-1 [T02016]; 637; 642; 2.144000; ACAAGT; 1.07886; 1.03772;
Sequence; En-1 [T02016]; 993; 998; 0.000000; ACAACC; 1.07886; 1.11080;
Sequence; En-1 [T02016]; 1041; 1046; 2.717256; CTTTGT; 0.35962; 0.34733;
Sequence; En-1 [T02016]; 1068; 1073; 3.215999; TATTGT; 1.07886; 0.84207;
Sequence; En-1 [T02016]; 1377; 1382; 3.215999; ACAATA; 1.07886; 0.84207;
Sequence; PR B [T00696]; 421; 427; 5.756916; CTTGTTC; 0.53943; 0.50929;
Sequence; PR B [T00696]; 442; 448; 7.879889; CAACACC; 0.53943; 0.63035;
Sequence; PR B [T00696]; 537; 543; 5.661264; TAACATT; 0.17981; 0.11416;
Sequence; PR B [T00696]; 785; 791; 7.172232; CAACAGA; 0.53943; 0.58190;
Sequence; PR B [T00696]; 931; 937; 5.756916; CATGTTC; 0.53943; 0.50929;
Sequence; PR B [T00696]; 1042; 1048; 8.587547; TTTGTTG; 1.34857; 1.27135;
Sequence; PR B [T00696]; 1069; 1075; 5.661264; ATTGTTA; 0.17981; 0.11416;
Sequence; PR B [T00696]; 1166; 1172; 9.295206; TAACACC; 0.26971; 0.24768;
Sequence; PR B [T00696]; 1186; 1192; 4.341599; TCTGTTC; 0.26971; 0.28825;
Sequence; PR B [T00696]; 1250; 1256; 2.830632; AAACAGT; 0.17981; 0.15536;
Sequence; PR B [T00696]; 1385; 1391; 5.661264; AATGTTA; 0.17981; 0.11416;
Sequence; PR A [T01661]; 421; 427; 5.756916; CTTGTTC; 0.53943; 0.50929;

Sequence; PR A [T01661]; 442; 448; 7.879889; CAACACC; 0.53943; 0.63035;
Sequence; PR A [T01661]; 537; 543; 5.661264; TAACATT; 0.17981; 0.11416;
Sequence; PR A [T01661]; 785; 791; 7.172232; CAACAGA; 0.53943; 0.58190;
Sequence; PR A [T01661]; 931; 937; 5.756916; CATGTTC; 0.53943; 0.50929;
Sequence; PR A [T01661]; 1042; 1048; 8.587547; TTTGTTG; 1.34857; 1.27135;
Sequence; PR A [T01661]; 1069; 1075; 5.661264; ATTGTTA; 0.17981; 0.11416;
Sequence; PR A [T01661]; 1166; 1172; 9.295206; TAACACC; 0.26971; 0.24768;
Sequence; PR A [T01661]; 1186; 1192; 4.341599; TCTGTTC; 0.26971; 0.28825;
Sequence; PR A [T01661]; 1250; 1256; 2.830632; AAACAGT; 0.17981; 0.15536;
Sequence; PR A [T01661]; 1385; 1391; 5.661264; AATGTTA; 0.17981; 0.11416;
Sequence; Sox2 [T01836]; 353; 359; 10.140486; TGACAAG; 0.53943; 0.57027;
Sequence; Sox2 [T01836]; 421; 427; 5.115009; CTTGTTC; 0.62933; 0.62788;
Sequence; Sox2 [T01836]; 576; 582; 10.698872; CCACAAG; 1.07886; 1.07153;
Sequence; Sox2 [T01836]; 635; 641; 10.698872; GTACAAG; 1.07886; 1.07153;
Sequence; Sox2 [T01836]; 991; 997; 10.698872; CCACAAC; 1.07886; 1.07153;
Sequence; Sox2 [T01836]; 1042; 1048; 2.022887; TTTGTTG; 0.35962; 0.32218;
Sequence; Sox2 [T01836]; 1069; 1075; 3.955464; ATTGTTA; 0.26971; 0.20952;
Sequence; Sox2 [T01836]; 1375; 1381; 8.980940; GGACAAT; 0.26971; 0.22772;
Sequence; HMG I(Y) [T02368]; 159; 165; 2.675522; GGAAAAG; 0.35962; 0.35675;
Sequence; HMG I(Y) [T02368]; 422; 428; 6.766802; TTGTTCC; 0.44952; 0.52446;
Sequence; HMG I(Y) [T02368]; 432; 438; 7.874227; GGAAGCA; 0.35962; 0.37217;
Sequence; HMG I(Y) [T02368]; 539; 545; 8.577088; ACATTCC; 0.53943; 0.60045;
Sequence; HMG I(Y) [T02368]; 571; 577; 2.675522; CTTTTCC; 0.35962; 0.35675;
Sequence; HMG I(Y) [T02368]; 656; 662; 1.615531; AACTTCC; 0.17981; 0.14727;
Sequence; HMG I(Y) [T02368]; 710; 716; 7.014009; CCCTTCC; 0.44952; 0.52446;
Sequence; HMG I(Y) [T02368]; 1129; 1135; 1.615531; AACTTCC; 0.17981; 0.14727;
Sequence; HMG I(Y) [T02368]; 1143; 1149; 10.297523; GGAATGA; 0.17981; 0.15668;
Sequence; HMG I(Y) [T02368]; 1187; 1193; 5.906584; CTGTTCC; 0.53943; 0.50997;
Sequence; HMG I(Y) [T02368]; 1253; 1259; 4.091280; CAGTTCC; 0.26971; 0.25041;
Sequence; HMG I(Y) [T02368]; 1261; 1267; 4.538260; GGAAAGT; 0.35962; 0.34617;
Sequence; HMG I(Y) [T02368]; 1269; 1275; 2.475749; GGAAGTC; 0.35962; 0.35675;
Sequence; HMG I(Y) [T02368]; 1279; 1285; 3.535739; GGAAAAA; 0.26971; 0.23517;
Sequence; HMG I(Y) [T02368]; 1345; 1351; 2.475749; GGAAGTG; 0.35962; 0.35675;
Sequence; HMG I(Y) [T02368]; 1359; 1365; 1.720435; GGAAATA; 0.17981; 0.14727;
Sequence; STAT4 [T01577]; 159; 164; 1.470588; GGAAAA; 1.43848; 1.31856;
Sequence; STAT4 [T01577]; 423; 428; 2.941176; TGTTCC; 2.15771; 2.10612;
Sequence; STAT4 [T01577]; 432; 437; 4.411765; GGAAGC; 1.43848; 1.50153;
Sequence; STAT4 [T01577]; 540; 545; 4.411765; CATTCC; 1.43848; 1.50153;
Sequence; STAT4 [T01577]; 572; 577; 1.470588; TTTTCC; 1.43848; 1.31856;
Sequence; STAT4 [T01577]; 657; 662; 2.941176; ACTTCC; 2.15771; 2.10612;
Sequence; STAT4 [T01577]; 711; 716; 5.882353; CCTTCC; 0.35962; 0.39956;
Sequence; STAT4 [T01577]; 1130; 1135; 2.941176; ACTTCC; 2.15771; 2.10612;
Sequence; STAT4 [T01577]; 1143; 1148; 4.411765; GGAATG; 1.43848; 1.50153;
Sequence; STAT4 [T01577]; 1188; 1193; 2.941176; TGTTCC; 2.15771; 2.10612;
Sequence; STAT4 [T01577]; 1254; 1259; 1.470588; AGTTCC; 1.43848; 1.31856;
Sequence; STAT4 [T01577]; 1261; 1266; 2.941176; GGAAAG; 2.15771; 2.10612;
Sequence; STAT4 [T01577]; 1269; 1274; 2.941176; GGAAGT; 2.15771; 2.10612;
Sequence; STAT4 [T01577]; 1279; 1284; 1.470588; GGAAAA; 1.43848; 1.31856;
Sequence; STAT4 [T01577]; 1345; 1350; 2.941176; GGAAGT; 2.15771; 2.10612;

Sequence; STAT4 [T01577]; 1359; 1364; 0.000000; GGAAAT; 0.35962; 0.29067;
Sequence; GR-alpha [T00337]; 422; 429; 8.110311; TTGTTCCC; 0.17981; 0.19803;
Sequence; GR-alpha [T00337]; 440; 447; 12.539283; CTCAACAC; 0.13486; 0.12529;
Sequence; GR-alpha [T00337]; 783; 790; 13.275928; ACCAACAG; 0.42705; 0.42211;
Sequence; GR-alpha [T00337]; 932; 939; 5.675100; ATGTTCAA; 0.17981; 0.16311;
Sequence; GR-alpha [T00337]; 1043; 1050; 14.443525; TTGTTGGG; 0.58438; 0.57227;
Sequence; GR-alpha [T00337]; 1187; 1194; 6.737035; CTGTTCCC; 0.22476; 0.22467;
Sequence; GR-alpha [T00337]; 1248; 1255; 7.824533; CAAAACAG; 0.17981; 0.19803;
Sequence; PR B [T00697]; 423; 429; 1.746570; TGTTCCC; 0.08990; 0.09649;
Sequence; PR B [T00697]; 933; 939; 2.633301; TGTTCAA; 0.44952; 0.40209;
Sequence; PR B [T00697]; 1188; 1194; 1.746570; TGTTCCC; 0.08990; 0.09649;
Sequence; COE2 [T05006]; 90; 98; 14.251144; AGGGAGGCG; 0.53943; 0.61269;
Sequence; COE2 [T05006]; 179; 187; 14.982425; GGGGAGCAC; 0.13486; 0.15255;
Sequence; COE2 [T05006]; 422; 430; 14.982425; TTGTTCCCG; 0.13486; 0.15255;
Sequence; COE2 [T05006]; 430; 438; 14.251144; GGGGAAGCA; 0.53943; 0.61269;
Sequence; COE2 [T05006]; 539; 547; 7.047851; ACATTCCCA; 0.22476; 0.26296;
Sequence; COE2 [T05006]; 656; 664; 9.552577; AACTTCCCC; 0.31467; 0.32796;
Sequence; COE2 [T05006]; 710; 718; 10.727219; CCCTTCCCC; 0.49448; 0.54904;
Sequence; COE2 [T05006]; 797; 805; 14.251144; AGGGAGGTT; 0.53943; 0.61269;
Sequence; COE2 [T05006]; 911; 919; 14.251144; GGGGAGGCT; 0.53943; 0.61269;
Sequence; COE2 [T05006]; 954; 962; 5.873209; CAAGTCCCA; 0.26971; 0.28582;
Sequence; COE2 [T05006]; 1141; 1149; 11.746419; TGGGAATGA; 0.40457; 0.48560;
Sequence; COE2 [T05006]; 1187; 1195; 10.283857; CTGTTCCCA; 0.49448; 0.54904;
Sequence; COE2 [T05006]; 1207; 1215; 13.076502; AGGGACGAC; 0.26971; 0.28340;
Sequence; COE2 [T05006]; 1277; 1285; 13.076502; AGGGAAAAA; 0.26971; 0.28340;
Sequence; COE2 [T05006]; 1304; 1312; 14.251144; AGTATCCCC; 0.53943; 0.61269;
Sequence; COE2 [T05006]; 1373; 1381; 8.377934; GGGGACAAT; 0.08990; 0.09335;
Sequence; GR-beta [T01920]; 423; 430; 13.101211; TGTTCCCG; 0.89905; 0.83262;
Sequence; GR-beta [T01920]; 933; 940; 11.395403; TGTTCAAG; 0.26971; 0.25105;
Sequence; GR-beta [T01920]; 1188; 1195; 12.819070; TGTTCCCA; 0.89905; 0.83262;
Sequence; GR-beta [T01920]; 1247; 1254; 12.222795; CCAAAACA; 0.35962; 0.38368;
Sequence; c-Ets-1 [T00112]; 157; 163; 7.714180; ATGGAAA; 0.26971; 0.26189;
Sequence; c-Ets-1 [T00112]; 424; 430; 9.968715; GTTCCCG; 0.35962; 0.34290;
Sequence; c-Ets-1 [T00112]; 430; 436; 6.310307; GGGGAAG; 0.35962; 0.37075;
Sequence; c-Ets-1 [T00112]; 541; 547; 11.278440; ATTCCCA; 0.08990; 0.07823;
Sequence; c-Ets-1 [T00112]; 573; 579; 7.552643; TTTCCAC; 0.26971; 0.26189;
Sequence; c-Ets-1 [T00112]; 658; 664; 6.310307; CTTCCCC; 0.35962; 0.37075;
Sequence; c-Ets-1 [T00112]; 712; 718; 6.310307; CTTCCCC; 0.35962; 0.37075;
Sequence; c-Ets-1 [T00112]; 1131; 1137; 3.742021; CTTCCGG; 0.08990; 0.11085;
Sequence; c-Ets-1 [T00112]; 1141; 1147; 11.278440; TGGGAAT; 0.08990; 0.07823;
Sequence; c-Ets-1 [T00112]; 1189; 1195; 10.614861; GTTCCCA; 0.53943; 0.51881;
Sequence; c-Ets-1 [T00112]; 1255; 1261; 3.981481; GTTCCTG; 0.26971; 0.30625;
Sequence; c-Ets-1 [T00112]; 1259; 1265; 7.229570; CTGGAAA; 0.08990; 0.07932;
Sequence; c-Ets-1 [T00112]; 1267; 1273; 0.646146; TAGGAAG; 0.26971; 0.25504;
Sequence; c-Ets-1 [T00112]; 1277; 1283; 8.462584; AGGGAAA; 0.53943; 0.52964;
Sequence; c-Ets-1 [T00112]; 1343; 1349; 4.226631; ACGGAAG; 0.26971; 0.30625;
Sequence; c-Ets-1 [T00112]; 1357; 1363; 1.990740; CAGGAAA; 0.08990; 0.09012;
Sequence; E2F [T01547]; 424; 431; 14.145926; GTTCCCGG; 1.41600; 1.61880;
Sequence; E2F [T01547]; 541; 548; 14.145926; ATTCCCAC; 1.41600; 1.61880;

Sequence; E2F [T01547]; 1140; 1147; 14.145926; GTGGGAAT; 1.41600; 1.61880;
Sequence; E2F [T01547]; 1356; 1363; 14.145926; GCAGGAAA; 1.41600; 1.61880;
Sequence; STAT6 [T01581]; 425; 433; 13.630951; TTCCCGGGG; 0.03371; 0.04457;
Sequence; STAT6 [T01581]; 427; 435; 14.960856; CCCGGGGAA; 0.11800; 0.14357;
Sequence; STAT6 [T01581]; 542; 550; 11.220546; TTCCCACAT; 0.01686; 0.01623;
Sequence; STAT6 [T01581]; 659; 667; 10.683418; TTCCCCTAC; 0.06743; 0.05949;
Sequence; STAT6 [T01581]; 1190; 1198; 8.955346; TTCCCAATA; 0.07305; 0.06650;
Sequence; STAT6 [T01581]; 1274; 1282; 9.221328; TCTAGGGAA; 0.07305; 0.06650;
Sequence; C1 (long form) [T01592]; 426; 433; 12.500000; TCCCGGGG; 0.53943; 0.77055;
Sequence; C1 (long form) [T01592]; 906; 913; 12.500000; ACTCGGGG; 0.53943; 0.77055;
Sequence; C1 (short form) [T01593]; 426; 433; 12.500000; TCCCGGGG; 0.53943; 0.77055;
Sequence; C1 (short form) [T01593]; 906; 913; 12.500000; ACTCGGGG; 0.53943; 0.77055;
Sequence; C1-I [T02946]; 426; 433; 12.500000; TCCCGGGG; 0.53943; 0.77055;
Sequence; C1-I [T02946]; 906; 913; 12.500000; ACTCGGGG; 0.53943; 0.77055;
Sequence; c-Ets-1 [T00111]; 429; 438; 11.524840; CGGGGAAGCA; 0.14048; 0.14605;
Sequence; c-Ets-1 [T00111]; 656; 665; 10.459604; AACTTCCCCT; 0.06181; 0.06519;
Sequence; c-Ets-1 [T00111]; 710; 719; 14.771421; CCCTTCCCCA; 0.02248; 0.02294;
Sequence; c-Ets-1 [T00111]; 1129; 1138; 1.931657; AACTTCCGGG; 0.03090; 0.03572;
Sequence; c-Ets-1 [T00111]; 1266; 1275; 7.702312; GTAGGAAGTC; 0.07305; 0.07471;
Sequence; c-Ets-1 [T00111]; 1342; 1351; 6.833056; CACGGAAGTG; 0.04636; 0.04970;
Sequence; c-Ets-1 68 [T00115]; 429; 438; 9.959024; CGGGGAAGCA; 0.04636; 0.05288;
Sequence; c-Ets-1 68 [T00115]; 656; 665; 13.519868; AACTTCCCCT; 0.02669; 0.02814;
Sequence; c-Ets-1 68 [T00115]; 1129; 1138; 7.121687; AACTTCCGGG; 0.03231; 0.03595;
Sequence; c-Ets-1 68 [T00115]; 1266; 1275; 13.160949; GTAGGAAGTC; 0.06883; 0.06771;
Sequence; NF-kappaB1 [T00593]; 430; 441; 8.647610; GGGGAAGCACCT; 0.01010; 0.01341;
Sequence; NF-kappaB1 [T00593]; 801; 812; 13.714488; AGGTTCATCCCC; 0.02019; 0.02583;
Sequence; HNF-3beta [T02344]; 423; 429; 11.542216; TGTTCCC; 0.35962; 0.32089;
Sequence; HNF-3beta [T02344]; 440; 446; 3.281312; CTCAACA; 0.08990; 0.08738;
Sequence; HNF-3beta [T02344]; 535; 541; 9.460495; TCTAACA; 0.71924; 0.62934;
Sequence; HNF-3beta [T02344]; 783; 789; 6.349161; ACCAACA; 0.53943; 0.57230;
Sequence; HNF-3beta [T02344]; 933; 939; 10.501356; TGTTCAA; 0.35962; 0.26588;
Sequence; HNF-3beta [T02344]; 1044; 1050; 2.240452; TGTTGGG; 0.26971; 0.32020;
Sequence; HNF-3beta [T02344]; 1071; 1077; 10.830065; TGTTAGT; 0.35962; 0.33164;
Sequence; HNF-3beta [T02344]; 1164; 1170; 12.911786; AGTAACA; 0.17981; 0.16285;
Sequence; HNF-3beta [T02344]; 1188; 1194; 11.542216; TGTTCCC; 0.35962; 0.32089;
Sequence; HNF-3beta [T02344]; 1248; 1254; 0.000000; CAAAACA; 0.17981; 0.20160;
Sequence; HNF-3beta [T02344]; 1387; 1393; 11.542216; TGTTACA; 0.35962; 0.32089;
Sequence; MEF1 [T00506]; 263; 272; 13.163561; GCGAGTTGGC; 0.20650; 0.23628;
Sequence; MEF1 [T00506]; 440; 449; 10.881840; CTCAACACCA; 0.05900; 0.06863;
Sequence; MEF1 [T00506]; 653; 662; 12.561117; CCCAACTTCC; 0.06883; 0.09112;
Sequence; MEF1 [T00506]; 783; 792; 10.023282; ACCAACAGAC; 0.11800; 0.13676;
Sequence; LBM1 [T05245]; 326; 337; 9.426007; GAAAGGTGGGTG; 0.01422; 0.02194;
Sequence; LBM1 [T05245]; 445; 456; 6.645768; CACCAACCCTCA; 0.00711; 0.01042;
Sequence; LBM1 [T05245]; 812; 823; 8.978489; CACCCACCTTAA; 0.01422; 0.02238;
Sequence; RelA [T00595]; 461; 471; 12.180280; CTACTCGAAGC; 0.01580; 0.01652;
Sequence; c-Rel [T01154]; 462; 471; 1.028734; TACTCGAAGC; 0.01686; 0.01798;
Sequence; XBP1 [T03707]; 463; 472; 1.243456; ACTCGAAGCA; 0.01545; 0.01521;
Sequence; XBP1 [T03707]; 1393; 1402; 7.348919; ACTCGAGCAA; 0.03652; 0.03963;
Sequence; Myf-5 [T00521]; 466; 478; 11.871492; CGAAGCAGCAGGC; 0.04748; 0.05693;

Sequence; NF-E4 [T00560]; 474; 483; 12.280901; CAGGCTTCTG; 0.44250; 0.53277;
Sequence; NF-E4 [T00560]; 1363; 1372; 13.458911; ATAAATCCTG; 0.14750; 0.15837;
Sequence; NF-E4 [T00560]; 1454; 1463; 12.280901; CAGGCAGCGG; 0.44250; 0.53277;
Sequence; NF-Y [T00150]; 484; 491; 2.516854; ATGCCAAT; 0.31467; 0.29317;
Sequence; NF-Y [T00150]; 1190; 1197; 3.846095; TTCCCAAT; 0.08990; 0.07133;
Sequence; POU2F1a [T00644]; 484; 491; 2.798811; ATGCCAAT; 0.04495; 0.03545;
Sequence; POU2F1a [T00644]; 549; 556; 10.378645; ATGCTAAG; 0.04495; 0.03508;
Sequence; POU2F1a [T00644]; 850; 857; 12.555498; ATGCCAAG; 0.13486; 0.12203;
Sequence; POU5F1 [T00651]; 484; 491; 4.459547; ATGCCAAT; 0.04495; 0.03545;
Sequence; LF-A1 [T00467]; 16; 22; 5.631412; GGAGGCA; 0.80914; 0.84678;
Sequence; LF-A1 [T00467]; 78; 84; 9.701266; GGTGGCA; 1.61829; 1.82820;
Sequence; LF-A1 [T00467]; 267; 273; 9.701266; GTTGGCA; 1.61829; 1.82820;
Sequence; LF-A1 [T00467]; 485; 491; 9.701266; TGCCAAT; 1.61829; 1.82820;
Sequence; LF-A1 [T00467]; 595; 601; 5.631412; TGCCTAA; 0.80914; 0.84678;
Sequence; LF-A1 [T00467]; 626; 632; 4.850633; CTAGGCA; 0.53943; 0.58418;
Sequence; LF-A1 [T00467]; 851; 857; 8.920488; TGCCAAG; 1.07886; 1.22651;
Sequence; LF-A1 [T00467]; 1009; 1015; 8.139709; TGCCATG; 0.17981; 0.20741;
Sequence; LF-A1 [T00467]; 1453; 1459; 5.631412; GCAGGCA; 0.80914; 0.84678;
Sequence; Cutl1 [T02042]; 486; 491; 0.000000; GCCAAT; 0.71924; 0.66152;
Sequence; Cutl1 [T02042]; 1069; 1074; 4.770093; ATTGTT; 1.43848; 1.15891;
Sequence; Cutl1 [T02042]; 1192; 1197; 1.543329; CCCAAT; 0.71924; 0.64709;
Sequence; Cutl1 [T02042]; 1232; 1237; 14.310279; GAGAAT; 2.87695; 2.02230;
Sequence; Cutl1 [T02042]; 1336; 1341; 11.083515; ATTTTG; 3.59619; 2.81753;
Sequence; Cutl1 [T02042]; 1359; 1364; 14.310279; GGAAAT; 2.87695; 2.02230;
Sequence; Cutl1 [T02042]; 1363; 1368; 14.310279; ATAAAT; 2.87695; 2.02230;
Sequence; Cutl1 [T02042]; 1376; 1381; 4.770093; GACAAT; 1.43848; 1.15891;
Sequence; Cutl1 [T02042]; 1379; 1384; 14.310279; AATAAT; 2.87695; 2.02230;
Sequence; Cutl1 [T02042]; 1382; 1387; 14.310279; AATAAT; 2.87695; 2.02230;
Sequence; Cutl1 [T02042]; 1433; 1438; 11.083515; CTCAAT; 3.59619; 2.81753;
Sequence; POU1F1a [T00691]; 112; 116; 4.810880; ATTCT; 2.87695; 2.10214;
Sequence; POU1F1a [T00691]; 208; 212; 0.000000; ATTCA; 2.87695; 1.97262;
Sequence; POU1F1a [T00691]; 487; 491; 7.514293; CCAAT; 2.87695; 2.37192;
Sequence; POU1F1a [T00691]; 541; 545; 5.921083; ATTCC; 4.31543; 3.26086;
Sequence; POU1F1a [T00691]; 861; 865; 0.000000; ATTCA; 2.87695; 1.97262;
Sequence; POU1F1a [T00691]; 1069; 1073; 5.663955; ATTGT; 1.43848; 1.14252;
Sequence; POU1F1a [T00691]; 1143; 1147; 5.921083; GGAAT; 4.31543; 3.26086;
Sequence; POU1F1a [T00691]; 1193; 1197; 7.514293; CCAAT; 2.87695; 2.37192;
Sequence; POU1F1a [T00691]; 1233; 1237; 4.810880; AGAAT; 2.87695; 2.10214;
Sequence; POU1F1a [T00691]; 1322; 1326; 0.974943; ATTAA; 2.87695; 1.97262;
Sequence; POU1F1a [T00691]; 1333; 1337; 5.921083; GAAAT; 4.31543; 3.26086;
Sequence; POU1F1a [T00691]; 1336; 1340; 4.810880; ATTTT; 2.87695; 2.10214;
Sequence; POU1F1a [T00691]; 1360; 1364; 5.921083; GAAAT; 4.31543; 3.26086;
Sequence; POU1F1a [T00691]; 1364; 1368; 0.000000; TAAAT; 2.87695; 1.97262;
Sequence; POU1F1a [T00691]; 1377; 1381; 5.663955; ACAAT; 1.43848; 1.14252;
Sequence; POU1F1a [T00691]; 1380; 1384; 5.785823; ATAAT; 4.31543; 3.26086;
Sequence; POU1F1a [T00691]; 1383; 1387; 5.785823; ATAAT; 4.31543; 3.26086;
Sequence; POU1F1a [T00691]; 1434; 1438; 0.853075; TCAAT; 2.87695; 1.97262;
Sequence; C/EBPdelta [T00109]; 112; 114; 0.000000; ATT; 23.01562; 17.57015;
Sequence; C/EBPdelta [T00109]; 208; 210; 0.000000; ATT; 23.01562; 17.57015;

Sequence; C/EBPdelta [T00109]; 489; 491; 0.000000; AAT; 23.01562; 17.57015;
Sequence; C/EBPdelta [T00109]; 541; 543; 0.000000; ATT; 23.01562; 17.57015;
Sequence; C/EBPdelta [T00109]; 861; 863; 0.000000; ATT; 23.01562; 17.57015;
Sequence; C/EBPdelta [T00109]; 1069; 1071; 0.000000; ATT; 23.01562; 17.57015;
Sequence; C/EBPdelta [T00109]; 1145; 1147; 0.000000; AAT; 23.01562; 17.57015;
Sequence; C/EBPdelta [T00109]; 1195; 1197; 0.000000; AAT; 23.01562; 17.57015;
Sequence; C/EBPdelta [T00109]; 1235; 1237; 0.000000; AAT; 23.01562; 17.57015;
Sequence; C/EBPdelta [T00109]; 1322; 1324; 0.000000; ATT; 23.01562; 17.57015;
Sequence; C/EBPdelta [T00109]; 1335; 1337; 0.000000; AAT; 23.01562; 17.57015;
Sequence; C/EBPdelta [T00109]; 1336; 1338; 0.000000; ATT; 23.01562; 17.57015;
Sequence; C/EBPdelta [T00109]; 1362; 1364; 0.000000; AAT; 23.01562; 17.57015;
Sequence; C/EBPdelta [T00109]; 1366; 1368; 0.000000; AAT; 23.01562; 17.57015;
Sequence; C/EBPdelta [T00109]; 1379; 1381; 0.000000; AAT; 23.01562; 17.57015;
Sequence; C/EBPdelta [T00109]; 1382; 1384; 0.000000; AAT; 23.01562; 17.57015;
Sequence; C/EBPdelta [T00109]; 1385; 1387; 0.000000; AAT; 23.01562; 17.57015;
Sequence; C/EBPdelta [T00109]; 1436; 1438; 0.000000; AAT; 23.01562; 17.57015;
Sequence; unc-86 [T01882]; 111; 115; 5.769231; GATTC; 5.75391; 4.66827;
Sequence; unc-86 [T01882]; 207; 211; 1.923077; TATTC; 5.75391; 4.01185;
Sequence; unc-86 [T01882]; 488; 492; 5.769231; CAATG; 5.75391; 4.66827;
Sequence; unc-86 [T01882]; 540; 544; 3.846154; CATTC; 8.63086; 6.57590;
Sequence; unc-86 [T01882]; 860; 864; 5.769231; GATTC; 5.75391; 4.66827;
Sequence; unc-86 [T01882]; 1068; 1072; 3.846154; TATTG; 8.63086; 6.57590;
Sequence; unc-86 [T01882]; 1144; 1148; 3.846154; GAATG; 8.63086; 6.57590;
Sequence; unc-86 [T01882]; 1194; 1198; 3.846154; CAATA; 8.63086; 6.57590;
Sequence; unc-86 [T01882]; 1234; 1238; 3.846154; GAATG; 8.63086; 6.57590;
Sequence; unc-86 [T01882]; 1321; 1325; 3.846154; CATTA; 8.63086; 6.57590;
Sequence; unc-86 [T01882]; 1334; 1338; 1.923077; AAATT; 5.75391; 4.01185;
Sequence; unc-86 [T01882]; 1335; 1339; 1.923077; AATTT; 5.75391; 4.01185;
Sequence; unc-86 [T01882]; 1361; 1365; 0.000000; AAATA; 1.43848; 1.00552;
Sequence; unc-86 [T01882]; 1365; 1369; 3.846154; AAATC; 8.63086; 6.57590;
Sequence; unc-86 [T01882]; 1378; 1382; 3.846154; CAATA; 8.63086; 6.57590;
Sequence; unc-86 [T01882]; 1381; 1385; 1.923077; TAATA; 5.75391; 4.01185;
Sequence; unc-86 [T01882]; 1384; 1388; 3.846154; TAATG; 8.63086; 6.57590;
Sequence; unc-86 [T01882]; 1435; 1439; 3.846154; CAATA; 8.63086; 6.57590;
Sequence; POU2F1 [T00641]; 484; 493; 13.626621; ATGCCAATGT; 0.47200; 0.34284;
Sequence; BR-C Z2 [T01478]; 110; 114; 9.216108; GGATT; 2.87695; 2.37192;
Sequence; BR-C Z2 [T01478]; 206; 210; 2.971418; TTATT; 1.43848; 1.00552;
Sequence; BR-C Z2 [T01478]; 489; 493; 8.363359; AATGT; 2.87695; 2.33413;
Sequence; BR-C Z2 [T01478]; 539; 543; 8.363359; ACATT; 2.87695; 2.33413;
Sequence; BR-C Z2 [T01478]; 859; 863; 9.216108; AGATT; 2.87695; 2.37192;
Sequence; BR-C Z2 [T01478]; 1067; 1071; 4.952363; ATATT; 2.87695; 2.05182;
Sequence; BR-C Z2 [T01478]; 1145; 1149; 6.382414; AATGA; 1.43848; 1.09662;
Sequence; BR-C Z2 [T01478]; 1195; 1199; 0.000000; AATAG; 1.43848; 1.09662;
Sequence; BR-C Z2 [T01478]; 1235; 1239; 6.382414; AATGA; 1.43848; 1.09662;
Sequence; BR-C Z2 [T01478]; 1320; 1324; 6.382414; TCATT; 1.43848; 1.09662;
Sequence; BR-C Z2 [T01478]; 1334; 1338; 7.510610; AAATT; 2.87695; 1.97262;
Sequence; BR-C Z2 [T01478]; 1335; 1339; 7.510610; AATTT; 2.87695; 1.97262;
Sequence; BR-C Z2 [T01478]; 1362; 1366; 2.971418; AATAA; 1.43848; 1.00552;
Sequence; BR-C Z2 [T01478]; 1366; 1370; 9.216108; AATCC; 2.87695; 2.37192;

Sequence; BR-C Z2 [T01478]; 1379; 1383; 2.971418; AATAA; 1.43848; 1.00552;
Sequence; BR-C Z2 [T01478]; 1382; 1386; 2.971418; AATAA; 1.43848; 1.00552;
Sequence; BR-C Z2 [T01478]; 1385; 1389; 8.363359; AATGT; 2.87695; 2.33413;
Sequence; BR-C Z2 [T01478]; 1436; 1440; 4.952363; AATAC; 2.87695; 2.05182;
Sequence; POU2F2B [T00964]; 483; 494; 14.988089; GATGCCAATGTG; 0.09956; 0.07751;
Sequence; POU2F2 [T00647]; 484; 494; 13.051865; ATGCCAATGTG; 0.29500; 0.22677;
Sequence; POU2F2 [T00648]; 484; 494; 13.388269; ATGCCAATGTG; 0.35400; 0.26096;
Sequence; POU2F2 (Oct-2.1) [T01864]; 484; 494; 13.388269; ATGCCAATGTG; 0.35400; 0.26096;
Sequence; POU2F2 (Oct-2.3) [T01865]; 484; 494; 13.388269; ATGCCAATGTG; 0.35400; 0.26096;
Sequence; POU2F2 (Oct-2.4) [T01866]; 484; 494; 13.388269; ATGCCAATGTG; 0.35400; 0.26096;
Sequence; POU2F2 (Oct-2.6) [T01867]; 484; 494; 13.388269; ATGCCAATGTG; 0.35400; 0.26096;
Sequence; POU2F2 (Oct-2.1) [T01870]; 484; 494; 13.388269; ATGCCAATGTG; 0.35400; 0.26096;
Sequence; NF-Y [T00613]; 486; 494; 2.758625; GCCAATGTG; 0.05619; 0.05639;
Sequence; NF-Y [T00613]; 1192; 1200; 7.344961; CCCAATAGC; 0.11238; 0.10369;
Sequence; MATalpha2 [T00487]; 124; 129; 1.844646; GGACAT; 2.15771; 1.88033;
Sequence; MATalpha2 [T00487]; 219; 224; 2.576772; TCACAT; 0.35962; 0.28781;
Sequence; MATalpha2 [T00487]; 490; 495; 3.073046; ATGTGT; 1.07886; 1.02079;
Sequence; MATalpha2 [T00487]; 537; 542; 1.472441; TAACAT; 0.71924; 0.53300;
Sequence; MATalpha2 [T00487]; 545; 550; 3.073046; CCACAT; 1.07886; 1.02079;
Sequence; MATalpha2 [T00487]; 834; 839; 3.073046; ACACAT; 1.07886; 1.02079;
Sequence; MATalpha2 [T00487]; 865; 870; 0.496274; ATGTAG; 1.07886; 0.82082;
Sequence; MATalpha2 [T00487]; 928; 933; 1.968715; CGACAT; 2.15771; 1.88033;
Sequence; MATalpha2 [T00487]; 932; 937; 1.844646; ATGTTC; 2.15771; 1.88033;
Sequence; MATalpha2 [T00487]; 1063; 1068; 1.844646; GGACAT; 2.15771; 1.88033;
Sequence; MATalpha2 [T00487]; 1078; 1083; 1.844646; GGACAT; 2.15771; 1.88033;
Sequence; MATalpha2 [T00487]; 1386; 1391; 1.472441; ATGTTA; 0.71924; 0.53300;
Sequence; MATalpha2 [T00487]; 1437; 1442; 0.496274; ATACAT; 1.07886; 0.82082;
Sequence; OSH15 [T05493]; 352; 359; 2.682344; GTGACAAG; 0.04495; 0.04554;
Sequence; OSH15 [T05493]; 504; 511; 14.878437; CCTCTCAC; 0.40457; 0.45302;
Sequence; OSH15 [T05493]; 1202; 1209; 14.878437; GTGAGAGG; 0.40457; 0.45302;
Sequence; PEBP2alphaA1 [T01062]; 512; 521; 7.283639; CCTACCGCAT; 0.03371; 0.04080;
Sequence; abaA [T01085]; 536; 545; 2.934548; CTAACATTCC; 0.06321; 0.05996;
Sequence; abaA [T01085]; 1143; 1152; 1.563999; GGAATGAAGG; 0.02107; 0.01849;
Sequence; RelA [T00594]; 430; 439; 9.226406; GGGGAAGCAC; 0.04636; 0.05075;
Sequence; RelA [T00594]; 538; 547; 8.027640; AACATTCCCA; 0.02248; 0.02262;
Sequence; RelA [T00594]; 655; 664; 12.664417; CAACTTCCCC; 0.07024; 0.07197;
Sequence; RelA [T00594]; 1141; 1150; 14.458365; TGGGAATGAA; 0.08990; 0.09150;
Sequence; HSF1 (long) [T01042]; 541; 548; 14.291973; ATTCCCAC; 0.60686; 0.51527;
Sequence; HSF1 (long) [T01042]; 861; 868; 12.600256; ATTCATGT; 0.80914; 0.71381;
Sequence; HSF1 (long) [T01042]; 1140; 1147; 14.291973; GTGGGAAT; 0.60686; 0.51527;
Sequence; HSF1 (short) [T02104]; 541; 548; 14.291973; ATTCCCAC; 0.60686; 0.51527;
Sequence; HSF1 (short) [T02104]; 861; 868; 12.600256; ATTCATGT; 0.80914; 0.71381;
Sequence; HSF1 (short) [T02104]; 1140; 1147; 14.291973; GTGGGAAT; 0.60686; 0.51527;
Sequence; Zta [T00923]; 493; 500; 10.953458; TGTGTCCT; 0.17981; 0.21019;
Sequence; Zta [T00923]; 542; 549; 10.667006; TTCCCACA; 0.35962; 0.36368;
Sequence; Zta [T00923]; 573; 580; 7.184667; TTTCCACA; 0.35962; 0.32019;
Sequence; Zta [T00923]; 723; 730; 10.667006; CTAGCACA; 0.35962; 0.36368;
Sequence; Zta [T00923]; 829; 836; 14.567312; AGGCCACA; 0.53943; 0.63397;
Sequence; Zta [T00923]; 988; 995; 14.567312; CGGCCACA; 0.53943; 0.63397;

Sequence; Zta [T00923]; 1110; 1117; 8.389285; CTTTCACA; 0.11238; 0.09007;
Sequence; COE3 [T05008]; 539; 551; 12.222268; ACATTCCCACATG; 0.02120; 0.02476;
Sequence; AML1 [T01067]; 540; 552; 14.492509; CATTCCCACATGC; 0.02805; 0.02853;
Sequence; Nrf2 [T01443]; 208; 218; 9.796651; ATTCAGCATCC; 0.06321; 0.05118;
Sequence; Nrf2 [T01443]; 547; 557; 12.623767; ACATGCTAAGG; 0.05338; 0.04401;
Sequence; Nrf2 [T01443]; 819; 829; 13.939071; CTTAAGCATGA; 0.09482; 0.07497;
Sequence; Nrf2 [T01443]; 836; 846; 11.111955; ACATGCTGAAG; 0.05900; 0.04810;
Sequence; MAZ [T00490]; 4; 14; 2.265536; CTGAGGGAGGG; 0.02037; 0.02926;
Sequence; MAZ [T00490]; 559; 569; 1.826224; CCCTCCCGGCC; 0.02213; 0.03151;
Sequence; MAZ [T00490]; 710; 720; 14.713202; CCCTTCCCCAC; 0.10325; 0.16516;
Sequence; MAZ [T00490]; 711; 721; 14.290795; CCTTCCCCACC; 0.10325; 0.16516;
Sequence; T-Ag [T00788]; 17; 25; 7.460153; GAGGCAGCC; 0.30343; 0.36002;
Sequence; T-Ag [T00788]; 93; 101; 7.460153; GAGGCGTCA; 0.30343; 0.36002;
Sequence; T-Ag [T00788]; 563; 571; 3.069503; CCCGGCCTC; 0.06743; 0.08776;
Sequence; T-Ag [T00788]; 739; 747; 7.460153; ACCCGCCTC; 0.30343; 0.36002;
Sequence; T-Ag [T00788]; 828; 836; 5.048016; GAGGCCACA; 0.20229; 0.24428;
Sequence; T-Ag [T00788]; 914; 922; 4.390650; GAGGCTTAT; 0.20229; 0.23768;
Sequence; NF-AT3 [T02462]; 159; 168; 10.185185; GGAAAAGAGC; 0.02248; 0.02558;
Sequence; NF-AT3 [T02462]; 568; 577; 9.259259; CCTCTTTTCC; 0.03371; 0.03486;
Sequence; NF-AT3 [T02462]; 1279; 1288; 0.925926; GGAAAAAAAA; 0.02248; 0.01616;
Sequence; NF-AT2 [T01945]; 159; 167; 8.685310; GGAAAAGAG; 0.14048; 0.13699;
Sequence; NF-AT2 [T01945]; 569; 577; 8.685310; CTCTTTTCC; 0.14048; 0.13699;
Sequence; NF-AT2 [T01945]; 1261; 1269; 8.321211; GGAAAGTAG; 0.14048; 0.13699;
Sequence; NF-AT2 [T01945]; 1279; 1287; 3.772227; GGAAAAAAA; 0.09552; 0.07530;
Sequence; NF-AT2 [T01945]; 1359; 1367; 5.292966; GGAAATAAA; 0.07305; 0.06354;
Sequence; NF-AT1 [T01948]; 159; 166; 3.634465; GGAAAAGA; 0.20229; 0.20178;
Sequence; NF-AT1 [T01948]; 570; 577; 3.634465; TCTTTTCC; 0.20229; 0.20178;
Sequence; NF-AT1 [T01948]; 1261; 1268; 2.565505; GGAAAGTA; 0.22476; 0.20058;
Sequence; NF-AT1 [T01948]; 1279; 1286; 1.068960; GGAAAAAA; 0.11238; 0.09192;
Sequence; NF-AT1 [T01948]; 1359; 1366; 1.924128; GGAAATAA; 0.13486; 0.11942;
Sequence; TRM1 [T05311]; 159; 166; 10.700472; GGAAAAGA; 0.26971; 0.26267;
Sequence; TRM1 [T05311]; 570; 577; 10.700472; TCTTTTCC; 0.26971; 0.26267;
Sequence; TRM1 [T05311]; 745; 752; 8.793097; CTCTTTCT; 0.40457; 0.34100;
Sequence; TRM1 [T05311]; 770; 777; 5.226274; CAGTTTCA; 0.24724; 0.19906;
Sequence; TRM1 [T05311]; 1108; 1115; 12.359921; TTCTTTCA; 1.01143; 0.90815;
Sequence; TRM1 [T05311]; 1224; 1231; 8.793097; AGAAAAAG; 0.40457; 0.34100;
Sequence; TRM1 [T05311]; 1239; 1246; 12.359921; AGAAAAGG; 1.01143; 0.90815;
Sequence; TRM1 [T05311]; 1261; 1268; 3.566824; GGAAAGTA; 0.13486; 0.12048;
Sequence; TRM1 [T05311]; 1279; 1286; 7.133648; GGAAAAAA; 0.33714; 0.32327;
Sequence; TRM1 [T05311]; 1332; 1339; 13.728472; TGAAATTT; 0.33714; 0.25104;
Sequence; TRM1 [T05311]; 1359; 1366; 8.502197; GGAAATAA; 0.11238; 0.09092;
Sequence; NF-AT1 [T01944]; 159; 165; 1.067633; GGAAAAG; 0.44952; 0.39642;
Sequence; NF-AT1 [T01944]; 571; 577; 1.067633; CTTTTCC; 0.44952; 0.39642;
Sequence; NF-AT1 [T01944]; 1261; 1267; 1.011016; GGAAAGT; 0.44952; 0.39642;
Sequence; NF-AT1 [T01944]; 1279; 1285; 0.355878; GGAAAAA; 0.17981; 0.17056;
Sequence; NF-AT1 [T01944]; 1359; 1365; 1.366893; GGAAATA; 0.44952; 0.43117;
Sequence; STAT1beta [T01573]; 155; 164; 12.705831; AGATGGAAAA; 0.15171; 0.14489;
Sequence; STAT1beta [T01573]; 572; 581; 11.593735; TTTTCCACAA; 0.22757; 0.20697;
Sequence; STAT1beta [T01573]; 1257; 1266; 2.898434; TCCTGGAAAG; 0.03793; 0.03192;

Sequence; STAT1beta [T01573]; 1355; 1364; 5.796867; AGCAGGAAAT; 0.11379; 0.09715;
Sequence; DPBF-1 [T04363]; 576; 585; 6.027747; CCACAAGTCG; 0.05619; 0.06485;
Sequence; DPBF-1 [T04363]; 991; 1000; 9.410325; CCACAACCAA; 0.07586; 0.08630;
Sequence; DPBF-2 [T04364]; 576; 585; 6.027747; CCACAAGTCG; 0.05619; 0.06485;
Sequence; DPBF-2 [T04364]; 991; 1000; 9.410325; CCACAACCAA; 0.07586; 0.08630;
Sequence; TAF [T00778]; 583; 589; 4.046435; TCGTCTG; 1.07886; 1.05795;
Sequence; TAF [T00778]; 883; 889; 4.046435; TCGTCTA; 1.07886; 1.05795;
Sequence; TAF [T00778]; 1054; 1060; 11.992048; TCGTGGG; 1.43848; 1.41838;
Sequence; TAF [T00778]; 1208; 1214; 9.294425; GGGACGA; 0.35962; 0.40623;
Sequence; c-Myb [T00137]; 263; 270; 7.587189; GCGAGTTG; 0.31467; 0.26564;
Sequence; c-Myb [T00137]; 590; 597; 4.840682; TAACTTGC; 0.22476; 0.20486;
Sequence; c-Myb [T00137]; 598; 605; 10.427413; CTAAGTTA; 0.13486; 0.11253;
Sequence; c-Myb [T00137]; 655; 662; 2.687937; CAACTTCC; 0.04495; 0.04417;
Sequence; c-Myb [T00137]; 768; 775; 9.024874; CTCAGTTT; 0.29219; 0.24044;
Sequence; c-Myb [T00137]; 1087; 1094; 11.014669; AAACTGAA; 0.31467; 0.24081;
Sequence; c-Myb [T00137]; 1128; 1135; 2.687937; CAACTTCC; 0.04495; 0.04417;
Sequence; c-Myb [T00137]; 1251; 1258; 9.729271; AACAGTTC; 0.26971; 0.22742;
Sequence; c-Myb [T00137]; 1286; 1293; 11.014669; AAACTGAA; 0.31467; 0.24081;
Sequence; c-Myb [T00137]; 1314; 1321; 6.040180; AAACTGTC; 0.15733; 0.13444;
Sequence; C/EBPalpha [T00104]; 271; 276; 2.526630; GCAAAG; 1.07886; 1.05795;
Sequence; C/EBPalpha [T00104]; 298; 303; 0.359839; GCAACC; 1.07886; 1.20681;
Sequence; C/EBPalpha [T00104]; 592; 597; 3.610025; ACTTGC; 1.43848; 1.53260;
Sequence; C/EBPalpha [T00104]; 648; 653; 3.610025; GCAAGC; 1.43848; 1.53260;
Sequence; C/EBPalpha [T00104]; 896; 901; 0.359839; GGTTGC; 1.07886; 1.20681;
Sequence; C/EBPalpha [T00104]; 1006; 1011; 3.610025; CCTTGC; 1.43848; 1.53260;
Sequence; C/EBPalpha [T00104]; 1016; 1021; 3.610025; ACTTGC; 1.43848; 1.53260;
Sequence; C/EBPalpha [T00104]; 1085; 1090; 2.526630; GCAAAC; 1.07886; 1.05795;
Sequence; C/EBPalpha [T00104]; 1127; 1132; 0.359839; GCAACT; 1.07886; 1.20681;
Sequence; C/EBPalpha [T00104]; 1337; 1342; 2.166790; TTTTGC; 0.35962; 0.34733;
Sequence; C/EBPalpha [T00104]; 1351; 1356; 2.526630; GCAAAG; 1.07886; 1.05795;
Sequence; C/EBPalpha [T00104]; 1399; 1404; 3.610025; GCAAGT; 1.43848; 1.53260;
Sequence; C/EBPalpha [T00105]; 269; 274; 1.717627; TGGCAA; 2.15771; 2.02832;
Sequence; C/EBPalpha [T00105]; 296; 301; 1.073517; CAGCAA; 1.43848; 1.51651;
Sequence; C/EBPalpha [T00105]; 594; 599; 1.502923; TTGCCT; 2.15771; 2.02832;
Sequence; C/EBPalpha [T00105]; 646; 651; 0.644110; GTGCAA; 0.71924; 0.68624;
Sequence; C/EBPalpha [T00105]; 898; 903; 0.644110; TTGCAC; 0.71924; 0.68624;
Sequence; C/EBPalpha [T00105]; 1008; 1013; 1.717627; TTGCCA; 2.15771; 2.02832;
Sequence; C/EBPalpha [T00105]; 1018; 1023; 1.073517; TTGCTG; 1.43848; 1.51651;
Sequence; C/EBPalpha [T00105]; 1083; 1088; 0.858813; TAGCAA; 1.43848; 1.51651;
Sequence; C/EBPalpha [T00105]; 1125; 1130; 0.000000; GAGCAA; 0.35962; 0.36756;
Sequence; C/EBPalpha [T00105]; 1339; 1344; 0.644110; TTGCAC; 0.71924; 0.68624;
Sequence; C/EBPalpha [T00105]; 1349; 1354; 0.644110; GTGCAA; 0.71924; 0.68624;
Sequence; C/EBPalpha [T00105]; 1397; 1402; 0.000000; GAGCAA; 0.35962; 0.36756;
Sequence; C/EBPalpha [T00108]; 269; 274; 3.200469; TGGCAA; 2.15771; 2.28955;
Sequence; C/EBPalpha [T00108]; 296; 301; 2.608671; CAGCAA; 1.07886; 1.11028;
Sequence; C/EBPalpha [T00108]; 594; 599; 2.956994; TTGCCT; 2.15771; 2.28955;
Sequence; C/EBPalpha [T00108]; 646; 651; 0.000000; GTGCAA; 0.71924; 0.63936;
Sequence; C/EBPalpha [T00108]; 898; 903; 0.000000; TTGCAC; 0.71924; 0.63936;
Sequence; C/EBPalpha [T00108]; 1008; 1013; 3.200469; TTGCCA; 2.15771; 2.28955;

Sequence; C/EBPalpha [T00108]; 1018; 1023; 2.608671; TTGCTG; 1.07886; 1.11028;
Sequence; C/EBPalpha [T00108]; 1083; 1088; 2.486934; TAGCAA; 1.07886; 1.11028;
Sequence; C/EBPalpha [T00108]; 1125; 1130; 2.081143; GAGCAA; 0.71924; 0.71490;
Sequence; C/EBPalpha [T00108]; 1339; 1344; 0.000000; TTGCAC; 0.71924; 0.63936;
Sequence; C/EBPalpha [T00108]; 1349; 1354; 0.000000; GTGCAA; 0.71924; 0.63936;
Sequence; C/EBPalpha [T00108]; 1397; 1402; 2.081143; GAGCAA; 0.71924; 0.71490;
Sequence; C/EBPbeta [T00459]; 269; 274; 2.241783; TGGCAA; 1.43848; 1.37540;
Sequence; C/EBPbeta [T00459]; 296; 301; 2.114582; CAGCAA; 1.43848; 1.37540;
Sequence; C/EBPbeta [T00459]; 594; 599; 1.593835; TTGCCT; 1.43848; 1.38896;
Sequence; C/EBPbeta [T00459]; 646; 651; 0.000000; GTGCAA; 0.35962; 0.33891;
Sequence; C/EBPbeta [T00459]; 898; 903; 0.000000; TTGCAC; 0.35962; 0.33891;
Sequence; C/EBPbeta [T00459]; 1008; 1013; 2.241783; TTGCCA; 1.43848; 1.37540;
Sequence; C/EBPbeta [T00459]; 1018; 1023; 2.114582; TTGCTG; 1.43848; 1.37540;
Sequence; C/EBPbeta [T00459]; 1083; 1088; 1.790608; TAGCAA; 1.43848; 1.37540;
Sequence; C/EBPbeta [T00459]; 1125; 1130; 0.386721; GAGCAA; 0.71924; 0.78746;
Sequence; C/EBPbeta [T00459]; 1339; 1344; 0.000000; TTGCAC; 0.35962; 0.33891;
Sequence; C/EBPbeta [T00459]; 1349; 1354; 0.000000; GTGCAA; 0.35962; 0.33891;
Sequence; C/EBPbeta [T00459]; 1397; 1402; 0.386721; GAGCAA; 0.71924; 0.78746;
Sequence; C/EBPbeta [T00581]; 268; 274; 4.001351; TTGGCAA; 0.44952; 0.45706;
Sequence; C/EBPbeta [T00581]; 295; 301; 5.205703; ACAGCAA; 0.71924; 0.70534;
Sequence; C/EBPbeta [T00581]; 594; 600; 4.784433; TTGCCTA; 0.71924; 0.70534;
Sequence; C/EBPbeta [T00581]; 645; 651; 0.805985; AGTGCAA; 0.26971; 0.24940;
Sequence; C/EBPbeta [T00581]; 898; 904; 0.805985; TTGCACT; 0.26971; 0.24940;
Sequence; C/EBPbeta [T00581]; 1008; 1014; 4.807336; TTGCCAT; 0.71924; 0.70534;
Sequence; C/EBPbeta [T00581]; 1018; 1024; 6.280350; TTGCTGG; 0.62933; 0.75526;
Sequence; C/EBPbeta [T00581]; 1082; 1088; 3.639539; ATAGCAA; 0.53943; 0.45372;
Sequence; C/EBPbeta [T00581]; 1124; 1130; 1.681833; AGAGCAA; 0.17981; 0.18661;
Sequence; C/EBPbeta [T00581]; 1339; 1345; 1.880632; TTGCACG; 0.35962; 0.34037;
Sequence; C/EBPbeta [T00581]; 1348; 1354; 0.805985; AGTGCAA; 0.26971; 0.24940;
Sequence; C/EBPbeta [T00581]; 1396; 1402; 2.756480; CGAGCAA; 0.71924; 0.72719;
Sequence; AP-3 (2) [T00039]; 241; 247; 12.734565; TGCTTAG; 0.35962; 0.32963;
Sequence; AP-3 (2) [T00039]; 598; 604; 8.971049; CTAAGTT; 0.44952; 0.39801;
Sequence; AP-3 (2) [T00039]; 600; 606; 7.527031; AAGTTAG; 1.34857; 1.09977;
Sequence; AP-3 (2) [T00039]; 887; 893; 7.527031; CTAAAGC; 1.34857; 1.09977;
Sequence; AP-3 (2) [T00039]; 1157; 1163; 11.290546; CTTTTAG; 1.07886; 0.91295;
Sequence; BTEB3 [T05051]; 5; 13; 14.953278; TGAGGGAGG; 0.17981; 0.20169;
Sequence; BTEB3 [T05051]; 12; 20; 9.006155; GGGCGGAGG; 0.11238; 0.12637;
Sequence; BTEB3 [T05051]; 135; 143; 12.939919; CGGCGGAGA; 0.13486; 0.16242;
Sequence; BTEB3 [T05051]; 607; 615; 9.026283; GCTCCACCC; 0.11238; 0.12637;
Sequence; BTEB3 [T05051]; 686; 694; 9.026283; GCTCCACCC; 0.11238; 0.12637;
Sequence; BTEB3 [T05051]; 909; 917; 9.443521; CGGGGGAGG; 0.15733; 0.17275;
Sequence; BTEB3 [T05051]; 1227; 1235; 9.026283; AAAAGGAGA; 0.11238; 0.12637;
Sequence; R1 [T00711]; 3; 14; 9.864560; GCTGAGGGAGGG; 0.02177; 0.02864;
Sequence; R1 [T00711]; 7; 18; 7.453148; AGGGAGGGCGGA; 0.01545; 0.02012;
Sequence; R1 [T00711]; 609; 620; 9.060757; TCCACCCTCAGA; 0.02739; 0.03777;
Sequence; E2F-1 [T01543]; 622; 630; 4.784594; CCCGCTAGG; 0.13486; 0.16940;
Sequence; E2F-1 [T01543]; 740; 748; 6.570994; CCCGCCTCT; 0.15171; 0.25486;
Sequence; AP-1 [T00029]; 637; 644; 13.167522; ACAAGTCA; 0.17981; 0.16527;
Sequence; AP-1 [T00029]; 1014; 1021; 13.173523; TGACTTGC; 0.17981; 0.16527;

Sequence; WRKY2 [T03974]; 636; 647; 10.425390; TACAAGTCAAGT; 0.02581; 0.01941;
Sequence; Nkx2-5 [T01675]; 313; 321; 8.861753; CACTTCAGC; 0.13486; 0.12805;
Sequence; Nkx2-5 [T01675]; 640; 648; 0.000000; AGTCAAGTG; 0.01124; 0.00945;
Sequence; Nkx2-5 [T01675]; 1039; 1047; 11.835619; CACTTTGTT; 0.02810; 0.02444;
Sequence; Nkx2-5 [T01675]; 1343; 1351; 8.861753; ACGGAAGTG; 0.13486; 0.12805;
Sequence; WIZZ [T03807]; 639; 649; 7.806018; AAGTCAAGTGC; 0.03968; 0.03265;
Sequence; C/EBPbeta(p20) [T01420]; 644; 651; 14.000258; AAGTGCAA; 1.21371; 0.99050;
Sequence; C/EBPbeta(p20) [T01420]; 898; 905; 14.000258; TTGCACTC; 1.21371; 0.99050;
Sequence; C/EBPbeta(p20) [T01420]; 1339; 1346; 11.843128; TTGCACGG; 0.29219; 0.25767;
Sequence; C/EBPbeta(p20) [T01420]; 1347; 1354; 14.000258; AAGTGCAA; 1.21371; 0.99050;
Sequence; TCF-2 [T01110]; 646; 655; 6.140450; GTGCAAGCCC; 0.08429; 0.09082;
Sequence; p53 [T00671]; 12; 18; 7.700559; GGGCGGA; 0.35962; 0.45174;
Sequence; p53 [T00671]; 49; 55; 3.624632; GGGCCTG; 0.26971; 0.39278;
Sequence; p53 [T00671]; 103; 109; 7.700559; GGGCTGA; 0.35962; 0.45174;
Sequence; p53 [T00671]; 649; 655; 3.365730; CAAGCCC; 0.26971; 0.39278;
Sequence; p53 [T00671]; 673; 679; 8.213500; GGGCGTC; 0.53943; 0.68436;
Sequence; p53 [T00671]; 966; 972; 9.173557; GGGCCAC; 0.89905; 1.22197;
Sequence; p53 [T00671]; 1048; 1054; 9.164941; GGGCCTT; 0.89905; 1.22197;
Sequence; p53 [T00671]; 1136; 1142; 3.365730; GGGCGTG; 0.26971; 0.39278;
Sequence; GCR1 [T00322]; 655; 665; 10.598919; CAACTTCCCCT; 0.02880; 0.02935;
Sequence; TGA1a [T00829]; 664; 669; 2.596276; CTACGT; 1.07886; 1.04159;
Sequence; TGA1a [T00829]; 666; 671; 2.596276; ACGTTT; 1.07886; 1.04159;
Sequence; E2F-5 [T01607]; 669; 676; 14.505689; TTTGGGGC; 0.47200; 0.56942;
Sequence; ETF [T00270]; 672; 680; 6.076766; GGGGCGTCA; 0.15171; 0.22098;
Sequence; GBF [T00315]; 689; 699; 9.130134; CCACCCCTAGC; 0.05443; 0.07090;
Sequence; USF2 [T00878]; 640; 648; 6.671276; AGTCAAGTG; 0.04495; 0.04227;
Sequence; USF2 [T00878]; 696; 704; 11.049635; TAGCTGACC; 0.06743; 0.08087;
Sequence; CREMalpha [T01803]; 696; 707; 11.891117; TAGCTGACCTCA; 0.04425; 0.03442;
Sequence; CREMalpha [T01803]; 700; 711; 13.191537; TGACCTCACCCC; 0.04372; 0.03938;
Sequence; T3R-beta1 [T00851]; 697; 707; 13.908978; AGCTGACCTCA; 0.11800; 0.10956;
Sequence; CRE-BP2 [T01017]; 699; 707; 13.990059; CTGACCTCA; 0.35400; 0.33149;
Sequence; CRE-BP2 [T01017]; 700; 708; 13.522367; TGACCTCAC; 0.11800; 0.12243;
Sequence; T3R-beta2 [T01350]; 344; 352; 10.964855; CCGGGTCAG; 0.14048; 0.14803;
Sequence; T3R-beta2 [T01350]; 699; 707; 3.162308; CTGACCTCA; 0.07305; 0.06529;
Sequence; T3R-beta2 [T01350]; 980; 988; 11.739623; ATGACCCAC; 0.12924; 0.14345;
Sequence; ATF3 [T01095]; 94; 101; 3.726574; AGGCGTCA; 0.17981; 0.15880;
Sequence; ATF3 [T01095]; 166; 173; 14.906297; AGCTGTCA; 0.35962; 0.32419;
Sequence; ATF3 [T01095]; 344; 351; 13.591135; CCGGGTCA; 0.35962; 0.37034;
Sequence; ATF3 [T01095]; 637; 644; 4.932280; ACAAGTCA; 0.35962; 0.34922;
Sequence; ATF3 [T01095]; 673; 680; 4.932280; GGGCGTCA; 0.35962; 0.34922;
Sequence; ATF3 [T01095]; 700; 707; 7.453148; TGACCTCA; 0.44952; 0.39067;
Sequence; ATF3 [T01095]; 981; 988; 12.385428; TGACCCAC; 0.71924; 0.69013;
Sequence; ATF3 [T01095]; 1014; 1021; 6.137987; TGACTTGC; 0.17981; 0.19123;
Sequence; RAR-alpha1 [T00719]; 699; 708; 2.946352; CTGACCTCAC; 0.03371; 0.03005;
Sequence; ARP-1 [T00045]; 343; 351; 10.069066; ACCGGGTCA; 0.10676; 0.12362;
Sequence; ARP-1 [T00045]; 700; 708; 4.486272; TGACCTCAC; 0.07867; 0.06996;
Sequence; ARP-1 [T00045]; 981; 989; 6.408113; TGACCCACG; 0.05619; 0.06206;
Sequence; RXR-alpha [T01345]; 700; 708; 1.954458; TGACCTCAC; 0.14048; 0.13242;
Sequence; ATF-1 [T00968]; 699; 709; 14.586651; CTGACCTCACC; 0.02529; 0.02810;

Sequence; CREBbeta [T02361]; 699; 709; 14.732021; CTGACCTCACC; 0.00632; 0.00766;
Sequence; ER-alpha [T00264]; 700; 709; 6.108876; TGACCTCACC; 0.07305; 0.06733;
Sequence; CREB [T00989]; 700; 709; 14.841915; TGACCTCACC; 0.07586; 0.07923;
Sequence; ANT [T02639]; 707; 717; 12.761984; ACCCCCTTCCC; 0.04636; 0.05876;
Sequence; AP-2 [T00034]; 174; 183; 0.771098; GTGCTGGGGA; 0.00562; 0.00712;
Sequence; AP-2 [T00034]; 425; 434; 7.779688; TTCCCGGGGA; 0.06602; 0.08677;
Sequence; AP-2 [T00034]; 714; 723; 6.184331; TCCCCACCTC; 0.03090; 0.03883;
Sequence; AP-2 [T00034]; 808; 817; 12.705889; TCCCCACCCA; 0.05619; 0.06946;
Sequence; AP-2 [T00034]; 906; 915; 8.550786; ACTCGGGGGA; 0.03652; 0.04729;
Sequence; AP-2 [T00034]; 1308; 1317; 10.559069; TCCCCAAAAC; 0.06181; 0.08026;
Sequence; MIG1 [T00509]; 172; 182; 13.097944; CAGTGCTGGGG; 0.13486; 0.20182;
Sequence; MIG1 [T00509]; 665; 675; 8.747858; TACGTTTGGGG; 0.06743; 0.09306;
Sequence; MIG1 [T00509]; 715; 725; 8.238023; CCCCACCTCTA; 0.10114; 0.10937;
Sequence; MIG1 [T00509]; 760; 770; 8.747858; CCCCAGACCTC; 0.06743; 0.09306;
Sequence; MIG1 [T00509]; 809; 819; 13.097944; CCCCACCCACC; 0.13486; 0.20182;
Sequence; MIG1 [T00509]; 1309; 1319; 12.635795; CCCCAAAACTG; 0.11238; 0.14645;
Sequence; MIG1 [T00509]; 1365; 1375; 5.322071; AAATCCTGGGG; 0.02248; 0.02842;
Sequence; VSF-1 [T02976]; 727; 738; 12.631701; CACAGCCAGAGG; 0.05558; 0.06597;
Sequence; DRF1.1 [T05835]; 13; 22; 12.948233; GGCGGAGGCA; 0.05338; 0.08125;
Sequence; DRF1.1 [T05835]; 136; 145; 12.250669; GGCGGAGATA; 0.07586; 0.10973;
Sequence; DRF1.1 [T05835]; 736; 745; 13.167236; AGGACCCGCC; 0.05338; 0.08125;
Sequence; DRF1.3 [T05837]; 13; 22; 12.948233; GGCGGAGGCA; 0.05338; 0.08125;
Sequence; DRF1.3 [T05837]; 136; 145; 12.250669; GGCGGAGATA; 0.07586; 0.10973;
Sequence; DRF1.3 [T05837]; 736; 745; 13.167236; AGGACCCGCC; 0.05338; 0.08125;
Sequence; E2F-1:DP-1 [T05204]; 94; 102; 13.924129; AGGCGTCAT; 0.20790; 0.28042;
Sequence; E2F-1:DP-1 [T05204]; 738; 746; 10.009249; GACCCGCCT; 0.12924; 0.18303;
Sequence; c-Ets-1 54 [T00114]; 93; 98; 0.000000; GAGGCG; 0.35962; 0.47174;
Sequence; c-Ets-1 54 [T00114]; 742; 747; 0.000000; CGCCTC; 0.35962; 0.47174;
Sequence; Sp1 [T01228]; 738; 748; 9.402032; GACCCGCCTCT; 0.01896; 0.03268;
Sequence; Sp1 [T01228]; 1096; 1106; 12.635787; ACCACGCCGTC; 0.08218; 0.13032;
Sequence; Sp1 [T01228]; 1134; 1144; 13.898109; CCGGGCGTGGG; 0.09798; 0.14165;
Sequence; Pu box binding factor [T00704]; 743; 752; 14.492168; GCCTCTTTCT; 0.15171; 0.15227;
Sequence; Pu box binding factor [T00704]; 1106; 1115; 8.695301; CCTTCTTTCA; 0.16436; 0.15112;
Sequence; Pu box binding factor [T00704]; 1224; 1233; 8.695301; AGAAAAAGGA; 0.16436; 0.15112;
Sequence; Pu box binding factor [T00704]; 1279; 1288; 2.898434; GGAAAAAAAA; 0.03793; 0.03164;
Sequence; Pu box binding factor [T00704]; 1359; 1368; 6.908963; GGAAATAAAT; 0.07586; 0.05335;
Sequence; Smad3 [T04096]; 784; 793; 3.579109; CCAACAGACA; 0.03933; 0.04336;
Sequence; Smad4 [T04292]; 490; 500; 14.812591; ATGTGTGTCCT; 0.02529; 0.02269;
Sequence; Smad4 [T04292]; 784; 794; 10.489908; CCAACAGACAC; 0.02107; 0.02474;
Sequence; Zeste [T00918]; 792; 798; 0.016809; CACTCAG; 0.35962; 0.35928;
Sequence; Zeste [T00918]; 901; 907; 0.000000; CACTCAC; 0.35962; 0.35928;
Sequence; Zeste [T00918]; 905; 911; 3.278975; CACTCGG; 0.35962; 0.40224;
Sequence; Zeste [T00918]; 1392; 1398; 3.262166; CACTCGA; 0.35962; 0.40224;
Sequence; Zeste [T02100]; 792; 798; 0.032173; CACTCAG; 0.35962; 0.35928;
Sequence; Zeste [T02100]; 901; 907; 0.000000; CACTCAC; 0.35962; 0.35928;
Sequence; Zeste [T02100]; 905; 911; 2.936991; CACTCGG; 0.35962; 0.40224;
Sequence; Zeste [T02100]; 1392; 1398; 2.904818; CACTCGA; 0.35962; 0.40224;
Sequence; TFII-I [T00824]; 803; 813; 10.736480; GTTCATCCCCA; 0.07024; 0.07507;
Sequence; Egr-3 [T00243]; 327; 339; 14.375592; AAAGGTGGGTGAG; 0.03688; 0.05217;

Sequence; Egr-3 [T00243]; 810; 822; 11.366723; CCCACCCACCTTA; 0.02120; 0.03026;
Sequence; Egr-3 [T05889]; 327; 339; 14.375592; AAAGGTGGGTGAG; 0.03688; 0.05217;
Sequence; Egr-3 [T05889]; 810; 822; 11.366723; CCCACCCACCTTA; 0.02120; 0.03026;
Sequence; Pbx1 [T06000]; 823; 834; 3.974159; AGCATGAGGCCA; 0.01027; 0.00901;
Sequence; RF2a [T02811]; 487; 496; 14.625352; CCAATGTGTG; 0.51414; 0.57861;
Sequence; RF2a [T02811]; 831; 840; 11.700281; GCCACACATG; 0.27393; 0.31666;
Sequence; RF2a [T02811]; 990; 999; 14.625352; GCCACAACCA; 0.51414; 0.57861;
Sequence; ALF1B [T01496]; 834; 841; 14.878437; ACACATGC; 0.40457; 0.39968;
Sequence; ALF1B [T01496]; 1183; 1190; 14.878437; GCTTCTGT; 0.40457; 0.39968;
Sequence; ALF1B [T01496]; 1464; 1471; 14.878437; ACTGATGC; 0.40457; 0.39968;
Sequence; GCM [T02302]; 184; 192; 10.735841; GCACGCATG; 0.11238; 0.13246;
Sequence; GCM [T02302]; 209; 217; 12.887507; TTCAGCATC; 0.19105; 0.19042;
Sequence; GCM [T02302]; 514; 522; 14.099892; TACCGCATC; 0.20229; 0.20523;
Sequence; GCM [T02302]; 837; 845; 12.887507; CATGCTGAA; 0.19105; 0.19042;
Sequence; GCMa [T02306]; 184; 192; 10.735841; GCACGCATG; 0.11238; 0.13246;
Sequence; GCMa [T02306]; 209; 217; 12.887507; TTCAGCATC; 0.19105; 0.19042;
Sequence; GCMa [T02306]; 514; 522; 14.099892; TACCGCATC; 0.20229; 0.20523;
Sequence; GCMa [T02306]; 837; 845; 12.887507; CATGCTGAA; 0.19105; 0.19042;
Sequence; GCMa [T02307]; 184; 192; 10.735841; GCACGCATG; 0.11238; 0.13246;
Sequence; GCMa [T02307]; 209; 217; 12.887507; TTCAGCATC; 0.19105; 0.19042;
Sequence; GCMa [T02307]; 514; 522; 14.099892; TACCGCATC; 0.20229; 0.20523;
Sequence; GCMa [T02307]; 837; 845; 12.887507; CATGCTGAA; 0.19105; 0.19042;
Sequence; GCMb [T02308]; 184; 192; 10.735841; GCACGCATG; 0.11238; 0.13246;
Sequence; GCMb [T02308]; 209; 217; 12.887507; TTCAGCATC; 0.19105; 0.19042;
Sequence; GCMb [T02308]; 514; 522; 14.099892; TACCGCATC; 0.20229; 0.20523;
Sequence; GCMb [T02308]; 837; 845; 12.887507; CATGCTGAA; 0.19105; 0.19042;
Sequence; octamer-binding factor [T01225]; 850; 860; 14.648906; ATGCCAAGAAG; 0.08850; 0.07393;
Sequence; octamer-binding factor [T01225]; 1349; 1359; 14.648906; GTGCAAAGCAG; 0.08850; 0.07393;
Sequence; SXR:RXR-alpha [T05670]; 55; 63; 10.263835; GAGTCCAGG; 0.17981; 0.16752;
Sequence; SXR:RXR-alpha [T05670]; 644; 652; 8.174786; AAGTGCAAG; 0.11238; 0.09484;
Sequence; SXR:RXR-alpha [T05670]; 897; 905; 10.263835; GTTGCACTC; 0.17981; 0.16752;
Sequence; SXR:RXR-alpha [T05670]; 1020; 1028; 12.352884; GCTGGACTG; 0.08990; 0.08408;
Sequence; SXR:RXR-alpha [T05670]; 1347; 1355; 7.175614; AAGTGCAAA; 0.22476; 0.19427;
Sequence; TBP [T00794]; 916; 923; 5.472732; GGCTTATA; 0.40457; 0.26887;
Sequence; FXR [T04494]; 932; 942; 8.275998; ATGTTCAAGGA; 0.01967; 0.01562;
Sequence; Pax-5 [T01201]; 624; 630; 3.826711; CGCTAGG; 0.53943; 0.58422;
Sequence; Pax-5 [T01201]; 945; 951; 5.376595; AGGAGCG; 0.17981; 0.23555;
Sequence; Pax-5 [T01201]; 1177; 1183; 6.688892; GCCAGCG; 0.71924; 0.81313;
Sequence; Pax-5 [T01201]; 1182; 1188; 4.298989; CGCTTCT; 0.44952; 0.53423;
Sequence; Pax-5 [T01201]; 1329; 1335; 3.512541; CGCTGAA; 0.35962; 0.40217;
Sequence; Pax-5 [T01201]; 1456; 1462; 4.658192; GGCAGCG; 0.44952; 0.53423;
Sequence; E47 [T05421]; 958; 967; 6.741841; TCCCACCTGG; 0.01124; 0.01224;
Sequence; Clock:Bmal1 [T05866]; 961; 972; 14.945058; CACCTGGGCCAC; 0.10325; 0.12830;
Sequence; LIP15 [T02803]; 965; 974; 11.934248; TGGGCCACCT; 0.17700; 0.21014;
Sequence; ROM1 [T02809]; 968; 976; 11.111111; GCCACCTTA; 0.15171; 0.18649;
Sequence; ROM2 [T02810]; 968; 976; 11.111111; GCCACCTTA; 0.15171; 0.18649;
Sequence; T3R-alpha [T00838]; 975; 986; 8.047167; TAGCCATGACCC; 0.01475; 0.01493;
Sequence; AP-1 [T01140]; 54; 60; 14.285714; TGAGTCC; 1.88800; 1.69020;

Sequence; AP-1 [T01140]; 981; 987; 14.285714; TGACCCA; 1.88800; 1.69020;
Sequence; ERRalpha1 [T05682]; 1028; 1040; 14.492509; GCTCACCTCAGCA; 0.02805; 0.02721;
Sequence; Xvent-1 [T04665]; 1038; 1045; 3.128458; GCACTTTG; 0.11238; 0.10597;
Sequence; Xvent-1 [T04665]; 1086; 1093; 2.699376; CAAACTGA; 0.22476; 0.20012;
Sequence; Xvent-1 [T04665]; 1404; 1411; 3.950759; TCTCTTTG; 0.17981; 0.18896;
Sequence; mat1-Mc [T01275]; 996; 1008; 12.909866; ACCAAAGACGCCT; 0.00869; 0.00743;
Sequence; mat1-Mc [T01275]; 1035; 1047; 11.185672; TCAGCACTTTGTT; 0.00764; 0.00721;
Sequence; LEF-1 [T00930]; 1036; 1047; 14.599597; CAGCACTTTGTT; 0.07164; 0.06799;
Sequence; LEF-1 [T00930]; 1402; 1413; 8.696886; AGTCTCTTTGAA; 0.01519; 0.01343;
Sequence; CUTL1 [T00100]; 486; 491; 0.836904; GCCAAT; 1.79810; 1.57265;
Sequence; CUTL1 [T00100]; 1069; 1074; 0.925283; ATTGTT; 1.79810; 1.57265;
Sequence; CUTL1 [T00100]; 1192; 1197; 0.880951; CCCAAT; 1.79810; 1.57265;
Sequence; CUTL1 [T00100]; 1376; 1381; 1.762187; GACAAT; 2.15771; 1.83388;
Sequence; CUTL1 [T00100]; 1433; 1438; 1.511826; CTCAAT; 1.07886; 0.81566;
Sequence; ROX1 [T01286]; 1066; 1075; 4.555042; CATATTGTTA; 0.03793; 0.02919;
Sequence; POU6F1 [T04470]; 1066; 1076; 14.123853; CATATTGTTAG; 0.05654; 0.03656;
Sequence; c-Myb [T00138]; 770; 778; 7.662220; CAGTTTCAG; 0.08990; 0.07831;
Sequence; c-Myb [T00138]; 1084; 1092; 10.820798; AGCAAACTG; 0.02248; 0.01864;
Sequence; c-Myb [T00138]; 1253; 1261; 3.091886; CAGTTCCTG; 0.08429; 0.07505;
Sequence; c-Myb [T00138]; 1283; 1291; 6.750722; AAAAAACTG; 0.11800; 0.10378;
Sequence; c-Myb [T00138]; 1311; 1319; 2.302241; CCAAAACTG; 0.04495; 0.04373;
Sequence; BR-C Z3 [T01479]; 1085; 1094; 14.914334; GCAAACTGAA; 0.06181; 0.05603;
Sequence; BR-C Z3 [T01479]; 1284; 1293; 11.870947; AAAAACTGAA; 0.08007; 0.07126;
Sequence; Pbx1b [T02087]; 2; 11; 14.774452; AGCTGAGGGA; 0.15171; 0.12301;
Sequence; Pbx1b [T02087]; 1109; 1118; 11.353285; TCTTTCACAG; 0.13486; 0.12324;
Sequence; Pbx1b [T02087]; 1144; 1153; 10.383774; GAATGAAGGA; 0.10114; 0.08990;
Sequence; Fli-1 [T01408]; 1127; 1139; 9.952027; GCAACTTCCGGGC; 0.01383; 0.01217;
Sequence; NF-AT2 [T01947]; 1127; 1139; 11.890307; GCAACTTCCGGGC; 0.02120; 0.02024;
Sequence; Arnt [T01346]; 185; 193; 8.605524; CACGCATGG; 0.11238; 0.14065;
Sequence; Arnt [T01346]; 1098; 1106; 9.368188; CACGCCGTC; 0.13486; 0.16677;
Sequence; Arnt [T01346]; 1134; 1142; 9.895773; CCGGGCGTG; 0.09552; 0.12850;
Sequence; AhR [T01795]; 182; 189; 2.358057; GAGCACGC; 0.33714; 0.41240;
Sequence; AhR [T01795]; 1095; 1102; 2.789346; GACCACGC; 0.35962; 0.47197;
Sequence; AhR [T01795]; 1138; 1145; 1.078222; GCGTGGGA; 0.11238; 0.15901;
Sequence; LyF-1 [T00479]; 372; 380; 8.303507; CTCCCACCA; 0.08990; 0.09835;
Sequence; LyF-1 [T00479]; 542; 550; 12.328339; TTCCCACAT; 0.08990; 0.08869;
Sequence; LyF-1 [T00479]; 957; 965; 14.598454; GTCCCACCT; 0.08990; 0.09969;
Sequence; LyF-1 [T00479]; 1138; 1146; 14.562381; GCGTGGGAA; 0.08990; 0.09969;
Sequence; LyF-1 [T00479]; 1190; 1198; 8.443995; TTCCCAATA; 0.08990; 0.09835;
Sequence; LyF-1 [T00479]; 1415; 1423; 2.282962; GCTTGGGAG; 0.01686; 0.01967;
Sequence; GT-1 [T00339]; 541; 548; 6.318530; ATTCCCAC; 0.17981; 0.15436;
Sequence; GT-1 [T00339]; 1140; 1147; 6.318530; GTGGGAAT; 0.17981; 0.15436;
Sequence; GT-1 [T00339]; 1322; 1329; 2.527412; ATTAACGC; 0.26971; 0.21608;
Sequence; GT-1 [T00339]; 1357; 1364; 11.540582; CAGGAAAT; 0.53943; 0.40906;
Sequence; GT-1 [T00339]; 1374; 1381; 7.582236; GGGACAAT; 0.44952; 0.36585;
Sequence; Su(H) [T01615]; 540; 552; 10.059751; CATTCCCACATGC; 0.00790; 0.00811;
Sequence; Su(H) [T01615]; 1136; 1148; 10.170990; GGGCGTGGGAATG; 0.00790; 0.00811;
Sequence; PEA3 [T00685]; 196; 203; 9.450812; CCCCATCC; 0.53943; 0.62845;
Sequence; PEA3 [T00685]; 211; 218; 9.236341; CAGCATCC; 0.17981; 0.18639;

Sequence; PEA3 [T00685]; 516; 523; 9.450812; CCGCATCC; 0.53943; 0.62845;
Sequence; PEA3 [T00685]; 803; 810; 7.141727; GTTCATCC; 0.26971; 0.24645;
Sequence; PEA3 [T00685]; 1151; 1158; 9.665283; GGATGGCT; 0.53943; 0.62845;
Sequence; MIF-1 [T01047]; 1160; 1172; 14.500958; TTAGAGTAACACC; 0.00878; 0.00730;
Sequence; E12 [T01786]; 173; 181; 3.221223; AGTGCTGGG; 0.11238; 0.15714;
Sequence; E12 [T01786]; 1017; 1025; 13.026981; CTTGCTGGA; 0.06743; 0.07792;
Sequence; E12 [T01786]; 1177; 1185; 3.221223; GCCAGCGCT; 0.11238; 0.15714;
Sequence; AP-3 [T01150]; 267; 275; 2.183970; GTTGGCAAA; 0.06743; 0.05117;
Sequence; AP-3 [T01150]; 1189; 1197; 4.312007; GTTCCCAAT; 0.09552; 0.07877;
Sequence; AP-3 [T01150]; 1307; 1315; 9.802102; ATCCCCAAA; 0.13486; 0.12486;
Sequence; SPF1 [T03975]; 1195; 1201; 14.285714; AATAGCA; 1.88800; 1.31371;
Sequence; SPF1 [T03975]; 1379; 1385; 14.285714; AATAATA; 1.88800; 1.31371;
Sequence; HOXD9 [T01424]; 1195; 1204; 10.933692; AATAGCAGTG; 0.13767; 0.10519;
Sequence; HOXD9 [T01424]; 1379; 1388; 6.852796; AATAATAATG; 0.05619; 0.03402;
Sequence; HOXD10 [T01425]; 1195; 1204; 10.933692; AATAGCAGTG; 0.13767; 0.10519;
Sequence; HOXD10 [T01425]; 1379; 1388; 6.852796; AATAATAATG; 0.05619; 0.03402;
Sequence; HOXD9 [T01755]; 1195; 1204; 10.933692; AATAGCAGTG; 0.13767; 0.10519;
Sequence; HOXD9 [T01755]; 1379; 1388; 6.852796; AATAATAATG; 0.05619; 0.03402;
Sequence; HOXD9 [T01756]; 1195; 1204; 10.933692; AATAGCAGTG; 0.13767; 0.10519;
Sequence; HOXD9 [T01756]; 1379; 1388; 6.852796; AATAATAATG; 0.05619; 0.03402;
Sequence; HOXD10 [T01757]; 1195; 1204; 10.933692; AATAGCAGTG; 0.13767; 0.10519;
Sequence; HOXD10 [T01757]; 1379; 1388; 6.852796; AATAATAATG; 0.05619; 0.03402;
Sequence; HOXD10 [T01758]; 1195; 1204; 10.933692; AATAGCAGTG; 0.13767; 0.10519;
Sequence; HOXD10 [T01758]; 1379; 1388; 6.852796; AATAATAATG; 0.05619; 0.03402;
Sequence; ANF [T00025]; 1218; 1229; 14.171063; TCAGCCAGAAAA; 0.09482; 0.08614;
Sequence; ANF [T01183]; 1218; 1229; 14.171063; TCAGCCAGAAAA; 0.09482; 0.08614;
Sequence; ANF [T01184]; 1218; 1229; 14.171063; TCAGCCAGAAAA; 0.09482; 0.08614;
Sequence; CIZ6-1 [T05137]; 1224; 1233; 6.164918; AGAAAAAGGA; 0.03371; 0.02933;
Sequence; CIZ6-1 [T05137]; 1279; 1288; 2.632607; GGAAAAAAAA; 0.02810; 0.02339;
Sequence; FOXM1 [T04208]; 1233; 1243; 14.636473; AGAATGAGAAA; 0.17068; 0.13134;
Sequence; NBF [T00951]; 1236; 1244; 14.103745; ATGAGAAAA; 0.11800; 0.08090;
Sequence; NBF [T01190]; 1236; 1244; 14.103745; ATGAGAAAA; 0.11800; 0.08090;
Sequence; PPAR-gamma:RXR-alpha [T05236]; 1238; 1249; 6.209569; GAGAAAAGGCCA; 0.00439; 0.00390;
Sequence; ERR1 [T04849]; 413; 423; 14.570644; GCTCGGACCTT; 0.22863; 0.19615;
Sequence; ERR1 [T04849]; 1243; 1253; 14.533310; AAGGCCAAAAC; 0.22863; 0.19615;
Sequence; GR [T00333]; 423; 429; 4.037122; TGTTCCC; 0.35962; 0.36756;
Sequence; GR [T00333]; 933; 939; 3.511009; TGTTCAA; 0.35962; 0.30045;
Sequence; GR [T00333]; 1188; 1194; 4.037122; TGTTCCC; 0.35962; 0.36756;
Sequence; GR [T00333]; 1248; 1254; 11.154851; CAAAACA; 0.35962; 0.33988;
Sequence; HNF-3alpha [T00371]; 1248; 1255; 3.184334; CAAAACAG; 0.31467; 0.30412;
Sequence; c-Ets-2 [T01397]; 1252; 1260; 4.495539; ACAGTTCCT; 0.02248; 0.01825;
Sequence; c-Ets-2 [T01397]; 1268; 1276; 12.868981; AGGAAGTCT; 0.33714; 0.27358;
Sequence; c-Ets-2 [T01397]; 1344; 1352; 11.770038; CGGAAGTGC; 0.33714; 0.28147;
Sequence; c-Ets-2 [T01397]; 1358; 1366; 4.976847; AGGAAATAA; 0.06743; 0.05627;
Sequence; ELF-1 [T01113]; 624; 636; 14.280835; CGCTAGGCAGTGT; 0.03617; 0.03403;
Sequence; ELF-1 [T01113]; 1252; 1264; 13.529900; ACAGTTCCTGGAA; 0.02608; 0.02424;
Sequence; ELF-1 [T01113]; 1264; 1276; 10.625526; AAGTAGGAAGTCT; 0.00852; 0.00714;
Sequence; STAT3 [T01493]; 1254; 1265; 13.818022; AGTTCCTGGAAA; 0.04565; 0.04721;

Sequence; STAT3 [T01493]; 1255; 1266; 13.050354; GTTCCTGGAAAG; 0.02950; 0.02869;
Sequence; IRF-2 [T00425]; 1259; 1271; 12.906878; CTGGAAAGTAGGA; 0.01536; 0.01272;
Sequence; PEA3 [T00684]; 1255; 1262; 7.245909; GTTCCTGG; 0.26971; 0.26110;
Sequence; PEA3 [T00684]; 1266; 1273; 4.375434; GTAGGAAG; 0.04495; 0.04287;
Sequence; PEA3 [T00684]; 1356; 1363; 0.000000; GCAGGAAA; 0.04495; 0.04565;
Sequence; c-Ets-2 [T00113]; 1253; 1260; 0.683141; CAGTTCCT; 0.06743; 0.05917;
Sequence; c-Ets-2 [T00113]; 1268; 1275; 2.485451; AGGAAGTC; 0.13486; 0.12901;
Sequence; c-Ets-2 [T00113]; 1358; 1365; 2.143880; AGGAAATA; 0.13486; 0.11733;
Sequence; Elk-1 [T05013]; 1268; 1276; 1.510524; AGGAAGTCT; 0.13486; 0.12872;
Sequence; Hb [T00395]; 1281; 1287; 0.122746; AAAAAAA; 0.26971; 0.22934;
Sequence; Hb [T00395]; 1282; 1288; 0.122746; AAAAAAA; 0.26971; 0.22934;
Sequence; Hb [T00395]; 1283; 1289; 0.245491; AAAAAAC; 0.26971; 0.22934;
Sequence; GATA-2 [T00308]; 141; 149; 2.222222; AGATAGAGA; 0.16857; 0.14250;
Sequence; GATA-2 [T00308]; 278; 286; 4.444445; TGATAAAGG; 0.17981; 0.15927;
Sequence; GATA-2 [T00308]; 1302; 1310; 8.888889; TCAGTATCC; 0.16857; 0.14889;
Sequence; AP-2beta [T02469]; 427; 438; 10.927068; CCCGGGGAAGCA; 0.01712; 0.02349;
Sequence; AP-2beta [T02469]; 908; 919; 14.955991; TCGGGGGAGGCT; 0.15435; 0.24239;
Sequence; AP-2beta [T02469]; 1304; 1315; 12.463326; AGTATCCCCAAA; 0.09640; 0.15418;
Sequence; HNF-1B [T01950]; 1318; 1325; 10.606163; TGTCATTA; 0.38210; 0.27012;
Sequence; HNF-1B [T01950]; 1381; 1388; 7.539987; TAATAATG; 0.24724; 0.15598;
Sequence; HNF-1B [T01950]; 1384; 1391; 9.759576; TAATGTTA; 0.35962; 0.22606;
Sequence; Ubx [T00863]; 1320; 1325; 0.890790; TCATTA; 1.43848; 0.97968;
Sequence; Ubx [T00863]; 1381; 1386; 1.525523; TAATAA; 1.43848; 0.92011;
Sequence; Ubx [T00863]; 1384; 1389; 1.237209; TAATGT; 1.43848; 0.97968;
Sequence; Crx [T03461]; 1320; 1325; 1.744366; TCATTA; 1.43848; 0.99048;
Sequence; Crx [T03461]; 1381; 1386; 2.035094; TAATAA; 1.43848; 0.91446;
Sequence; Crx [T03461]; 1384; 1389; 1.885291; TAATGT; 1.43848; 0.91446;
Sequence; HNF-1A [T00368]; 1320; 1327; 0.148779; TCATTAAC; 0.08990; 0.05503;
Sequence; HOX11 [T02054]; 1322; 1328; 0.052342; ATTAACG; 0.35962; 0.28535;
Sequence; SUT1 [T04582]; 1325; 1334; 6.908963; AACGCGCTGA; 0.07586; 0.08753;
Sequence; POU3F1 [T00969]; 1331; 1337; 12.861823; CTGAAAT; 0.53943; 0.33578;
Sequence; POU3F1 [T00969]; 1336; 1342; 14.474929; ATTTTGC; 0.71924; 0.56345;
Sequence; POU3F1 [T00969]; 1358; 1364; 10.733734; AGGAAAT; 0.71924; 0.56345;
Sequence; AGL3 [T03025]; 1332; 1337; 3.627583; TGAAAT; 1.43848; 1.08127;
Sequence; AGL3 [T03025]; 1336; 1341; 1.350799; ATTTTG; 0.71924; 0.52666;
Sequence; AGL3 [T03025]; 1359; 1364; 3.552538; GGAAAT; 1.43848; 1.08127;
Sequence; AGL3 [T03025]; 1363; 1368; 0.106623; ATAAAT; 0.71924; 0.46351;
Sequence; Pax-4a [T02983]; 1334; 1339; 3.139060; AAATTT; 0.35962; 0.20418;
Sequence; Pax-4a [T02983]; 1361; 1366; 1.167421; AAATAA; 0.71924; 0.51153;
Sequence; Pax-4a [T02983]; 1365; 1370; 4.067809; AAATCC; 1.43848; 1.16953;
Sequence; POU3F2 [T00630]; 1333; 1339; 9.094656; GAAATTT; 0.26971; 0.18188;
Sequence; POU3F2 [T00630]; 1334; 1340; 7.730221; AAATTTT; 0.89905; 0.60070;
Sequence; POU3F2 [T00630]; 1361; 1367; 0.505365; AAATAAA; 0.53943; 0.39033;
Sequence; POU3F2 [T00630]; 1365; 1371; 5.710420; AAATCCT; 0.71924; 0.55310;
Sequence; RAR-beta2 [T01326]; 1331; 1342; 4.992815; CTGAAATTTTGC; 0.00474; 0.00375;
Sequence; RAR-beta2 [T01326]; 1434; 1445; 14.978447; TCAATACATCAG; 0.10202; 0.08761;
Sequence; HNF-3gamma [T01050]; 270; 279; 12.942758; GGCAAAGCTG; 0.03231; 0.03068;
Sequence; HNF-3gamma [T01050]; 1334; 1343; 14.739089; AAATTTTGCA; 0.08288; 0.07663;
Sequence; HNF-3gamma [T01050]; 1350; 1359; 10.783722; TGCAAAGCAG; 0.06462; 0.06122;

Sequence; EFII [T00239]; 1334; 1344; 10.324682; AAATTTTGCAC; 0.08499; 0.09019;
Sequence; MAC1 [T01265]; 1083; 1089; 14.285714; TAGCAAA; 1.88800; 1.85838;
Sequence; MAC1 [T01265]; 1125; 1131; 14.285714; GAGCAAC; 1.88800; 1.85838;
Sequence; MAC1 [T01265]; 1238; 1244; 14.285714; GAGAAAA; 1.88800; 1.85838;
Sequence; MAC1 [T01265]; 1338; 1344; 14.285714; TTTGCAC; 1.88800; 1.85838;
Sequence; MAC1 [T01265]; 1349; 1355; 14.285714; GTGCAAA; 1.88800; 1.85838;
Sequence; MAC1 [T01265]; 1397; 1403; 14.285714; GAGCAAG; 1.88800; 1.85838;
Sequence; POU2F1 [T01031]; 1337; 1346; 11.638779; TTTTGCACGG; 0.05057; 0.04776;
Sequence; POU2F1 [T01031]; 1347; 1356; 7.965058; AAGTGCAAAG; 0.00562; 0.00449;
Sequence; E74A [T00208]; 1131; 1140; 8.932414; CTTCCGGGCG; 0.04214; 0.04330;
Sequence; E74A [T00208]; 1340; 1349; 12.259928; TGCACGGAAG; 0.07726; 0.08167;
Sequence; GABP [T00268]; 311; 322; 14.847057; ACCACTTCAGCT; 0.03688; 0.03772;
Sequence; GABP [T00268]; 1342; 1353; 3.041476; CACGGAAGTGCA; 0.00351; 0.00373;
Sequence; Nkx6-2 [T02050]; 1358; 1365; 4.651163; AGGAAATA; 0.33714; 0.25550;
Sequence; HNF-3alpha [T02512]; 1357; 1366; 8.372425; CAGGAAATAA; 0.03371; 0.02403;
Sequence; NERF-1a [T05021]; 1250; 1261; 14.379476; AAACAGTTCCTG; 0.04794; 0.04659;
Sequence; NERF-1a [T05021]; 1357; 1368; 14.955702; CAGGAAATAAAT; 0.04425; 0.04006;
Sequence; Croc [T02291]; 1360; 1369; 14.947243; GAAATAAATC; 0.23600; 0.14834;
Sequence; Olf-1 [T01040]; 85; 97; 12.661559; TCACCAGGGAGGC; 0.01826; 0.02298;
Sequence; Olf-1 [T01040]; 1365; 1377; 6.056941; AAATCCTGGGGGA; 0.00439; 0.00553;
Sequence; Sox13 [T02420]; 1069; 1076; 1.346210; ATTGTTAG; 0.62933; 0.51303;
Sequence; Sox13 [T02420]; 1374; 1381; 0.673105; GGGACAAT; 0.40457; 0.34020;
Sequence; Tll [T00789]; 1322; 1327; 0.107555; ATTAAC; 1.43848; 0.90931;
Sequence; Tll [T00789]; 1379; 1384; 4.500828; AATAAT; 2.87695; 1.97262;
Sequence; Tll [T00789]; 1382; 1387; 4.500828; AATAAT; 2.87695; 1.97262;
Sequence; MBF1 [T00492]; 1376; 1386; 14.636473; GACAATAATAA; 0.17068; 0.14079;
Sequence; Antp [T00026]; 1320; 1325; 2.226231; TCATTA; 2.15771; 1.32847;
Sequence; Antp [T00026]; 1381; 1386; 2.226231; TAATAA; 2.15771; 1.32847;
Sequence; Antp [T00026]; 1384; 1389; 2.226231; TAATGT; 2.15771; 1.32847;
Sequence; Gt [T00328]; 1377; 1387; 14.541822; ACAATAATAAT; 0.17454; 0.11594;
Sequence; Gt [T00328]; 1380; 1390; 3.898698; ATAATAATGTT; 0.00316; 0.00160;
Sequence; Prd [T00699]; 1319; 1325; 11.870926; GTCATTA; 0.17981; 0.12490;
Sequence; Prd [T00699]; 1381; 1387; 10.830065; TAATAAT; 0.35962; 0.21088;
Sequence; Prd [T00699]; 1384; 1390; 9.460495; TAATGTT; 0.71924; 0.45397;
Sequence; IPF1 [T02057]; 1318; 1325; 0.469022; TGTCATTA; 0.08990; 0.06545;
Sequence; IPF1 [T02057]; 1384; 1391; 2.011113; TAATGTTA; 0.26971; 0.19578;
Sequence; C/EBPgamma [T00216]; 594; 601; 0.215655; TTGCCTAA; 0.08990; 0.06577;
Sequence; C/EBPgamma [T00216]; 1018; 1025; 8.367175; TTGCTGGA; 0.17981; 0.17729;
Sequence; C/EBPgamma [T00216]; 1395; 1402; 7.224460; TCGAGCAA; 0.26971; 0.23019;
Sequence; MRF-2 [T04675]; 1435; 1444; 10.183125; CAATACATCA; 0.10817; 0.08143;
Sequence; TSAP [T00855]; 1453; 1462; 12.717730; GCAGGCAGCG; 0.20229; 0.22852;
Sequence; Adf-1 [T00008]; 0; 7; 3.695360; GCAGCTGA; 0.35962; 0.40372;
Sequence; Adf-1 [T00008]; 20; 27; 2.219675; GCAGCCAA; 0.17981; 0.24395;
Sequence; Adf-1 [T00008]; 410; 417; 3.596210; GCAGCTCG; 0.35962; 0.40372;
Sequence; Adf-1 [T00008]; 470; 477; 3.448101; GCAGCAGG; 0.35962; 0.40372;
Sequence; Adf-1 [T00008]; 1457; 1464; 0.989036; GCAGCGGA; 0.24724; 0.33586;
Sequence; MATa1 [T00488]; 122; 131; 10.503211; TAGGACATCA; 0.04776; 0.04487;
Sequence; MATa1 [T00488]; 1435; 1444; 6.964562; CAATACATCA; 0.01405; 0.01178;
Sequence; PUR alpha [T05167]; 912; 921; 14.905954; GGGAGGCTTA; 0.07867; 0.10591;

Sequence; PUR alpha [T05167]; 1419; 1428; 14.905954; GGGAGGACGG; 0.07867; 0.10591;
Sequence; PUR beta [T05172]; 912; 921; 14.905954; GGGAGGCTTA; 0.07867; 0.10591;
Sequence; PUR beta [T05172]; 1419; 1428; 14.905954; GGGAGGACGG; 0.07867; 0.10591;
Sequence; ATHB-5 [T04066]; 1379; 1389; 11.565805; AATAATAATGT; 0.03617; 0.02118;
Sequence; ATHB-5 [T04066]; 1382; 1392; 9.324313; AATAATGTTAC; 0.01510; 0.01023;
Sequence; HNF-1C [T01951]; 1380; 1388; 14.429214; ATAATAATG; 0.13486; 0.08691;
Sequence; POU4F1(l) [T01877]; 1379; 1388; 6.140450; AATAATAATG; 0.08429; 0.04849;
Sequence; ARF1 [T04514]; 1370; 1382; 14.033846; CTGGGGGACAATA; 0.01567; 0.01502;
Sequence; POU2F2 (Oct-2.1) [T00646]; 1361; 1368; 7.923404; AAATAAAT; 0.17981; 0.11110;
Sequence; TBP [T00798]; 277; 284; 6.455021; CTGATAAA; 0.20229; 0.15245;
Sequence; TBP [T00798]; 1360; 1367; 4.015529; GAAATAAA; 0.08990; 0.06410;
Sequence; MED8 [T03491]; 1329; 1337; 12.047317; CGCTGAAAT; 0.13486; 0.11045;
Sequence; MED8 [T03491]; 1356; 1364; 1.964100; GCAGGAAAT; 0.08990; 0.06904;
Sequence; FOXP3 [T04280]; 1332; 1340; 7.334112; TGAAATTTT; 0.16857; 0.12193;
Sequence; SBF-1 [T00739]; 1322; 1330; 14.499644; ATTAACGCG; 0.08990; 0.06182;
Sequence; HNF-1 [T00369]; 1318; 1329; 14.705068; TGTCATTAACGC; 0.01791; 0.01309;
Sequence; Meis-1a [T03388]; 165; 173; 0.359064; GAGCTGTCA; 0.04495; 0.03818;
Sequence; Meis-1a [T03388]; 1314; 1322; 0.000000; AAACTGTCA; 0.04495; 0.03873;
Sequence; Meis-1b [T03389]; 165; 173; 0.296000; GAGCTGTCA; 0.05057; 0.04288;
Sequence; Meis-1b [T03389]; 1314; 1322; 0.081934; AAACTGTCA; 0.03933; 0.03403;
Sequence; Elf-1 [T01019]; 1249; 1258; 8.909527; AAAACAGTTC; 0.06462; 0.05813;
Sequence; Elf-1 [T01019]; 1285; 1294; 13.486299; AAAACTGAAA; 0.06743; 0.06031;
Sequence; Elf-1 [T01019]; 1313; 1322; 12.973844; AAAACTGTCA; 0.05760; 0.04904;
Sequence; HOXA9 [T01710]; 1309; 1322; 8.828312; CCCCAAAACTGTCA; 0.00082; 0.00064;
Sequence; NF-AT4 [T01946]; 1279; 1290; 5.541758; GGAAAAAAAACT; 0.00983; 0.00779;
Sequence; FOXD3 [T02290]; 1250; 1257; 3.444986; AAACAGTT; 0.08990; 0.07574;
Sequence; HNF-3beta [T01049]; 1247; 1254; 1.902729; CCAAAACA; 0.22476; 0.19212;
Sequence; AR [T00040]; 423; 430; 3.768253; TGTTCCCG; 0.04495; 0.05171;
Sequence; AR [T00040]; 933; 940; 5.759750; TGTTCAAG; 0.33714; 0.36712;
Sequence; AR [T00040]; 1188; 1195; 2.872832; TGTTCCCA; 0.08990; 0.07932;
Sequence; R [T00710]; 86; 94; 10.243542; CACCAGGGA; 0.07867; 0.10010;
Sequence; R [T00710]; 130; 138; 0.548655; CACCACGGC; 0.05619; 0.08196;
Sequence; R [T00710]; 310; 318; 3.632624; CACCACTTC; 0.07867; 0.10823;
Sequence; R [T00710]; 376; 384; 3.851239; CACCACCAA; 0.05619; 0.07290;
Sequence; R [T00710]; 379; 387; 9.002867; CACCAAGGT; 0.08990; 0.09665;
Sequence; R [T00710]; 445; 453; 12.509834; CACCAACCC; 0.16857; 0.18943;
Sequence; R [T00710]; 1169; 1177; 13.097996; CACCAGTAG; 0.13486; 0.16832;
Sequence; Hlf [T01071]; 585; 593; 14.759054; GTCTGTAAC; 0.04495; 0.03510;
Sequence; Hlf [T01071]; 1161; 1169; 8.501296; TAGAGTAAC; 0.12924; 0.09747;
Sequence; Elf-1 [T05012]; 1129; 1137; 0.529089; AACTTCCGG; 0.01686; 0.02023;
Sequence; NRF-2 [T01199]; 1128; 1138; 10.955864; CAACTTCCGGG; 0.00843; 0.00991;
Sequence; Pax-6 [T01122]; 1112; 1122; 14.220607; TTCACAGCTAG; 0.08042; 0.06939;
Sequence; POU2F1 [T01466]; 1062; 1069; 13.593447; CGGACATA; 0.47200; 0.45567;
Sequence; POU2F1 [T01466]; 1077; 1084; 10.506966; TGGACATA; 0.31467; 0.25075;
Sequence; HNF-3 [T00370]; 1038; 1046; 10.291635; GCACTTTGT; 0.16857; 0.15027;
Sequence; AIRE [T05990]; 991; 1000; 9.130294; CCACAACCAA; 0.09693; 0.10396;
Sequence; REB [T02808]; 968; 977; 14.535101; GCCACCTTAG; 0.07586; 0.08713;
Sequence; PPAR-alpha:RXR-alpha [T05221]; 962; 972; 4.886580; ACCTGGGCCAC; 0.01264; 0.01545;
Sequence; Lmo2 [T02251]; 960; 967; 0.000000; CCACCTGG; 0.02248; 0.02909;

Sequence; Tal-1 [T00790]; 232; 242; 10.875765; CTCATCATCTG; 0.05057; 0.05418;
Sequence; Tal-1 [T00790]; 313; 323; 14.153038; CACTTCAGCTG; 0.11379; 0.12009;
Sequence; Tal-1 [T00790]; 956; 966; 13.546753; AGTCCCACCTG; 0.09482; 0.09924;
Sequence; Tal-1 [T01800]; 232; 242; 10.875765; CTCATCATCTG; 0.05057; 0.05418;
Sequence; Tal-1 [T01800]; 313; 323; 14.153038; CACTTCAGCTG; 0.11379; 0.12009;
Sequence; Tal-1 [T01800]; 956; 966; 13.546753; AGTCCCACCTG; 0.09482; 0.09924;
Sequence; DTF-1 [T00201]; 927; 937; 13.860411; GCGACATGTTC; 0.06638; 0.05980;
Sequence; MEF-2C/delta8 [T01769]; 920; 930; 12.435761; TATACCTGCGA; 0.08218; 0.05845;
Sequence; B factor [T00061]; 916; 923; 13.449835; GGCTTATA; 0.26971; 0.18075;
Sequence; TFIID [T00820]; 916; 923; 7.238121; GGCTTATA; 0.08990; 0.06604;
Sequence; DEAF-1 [T05885]; 878; 886; 3.417227; TCGGGTCGT; 0.07867; 0.09598;
Sequence; DEAF-1 [T05885]; 908; 916; 7.212653; TCGGGGGAG; 0.15733; 0.20818;
Sequence; UME6 [T01247]; 904; 914; 12.927645; TCACTCGGGGG; 0.06954; 0.08292;
Sequence; dri [T04679]; 857; 867; 14.875705; GAAGATTCATG; 0.18438; 0.14804;
Sequence; ADR1 [T00011]; 195; 202; 3.038707; ACCCCATC; 0.06743; 0.08196;
Sequence; ADR1 [T00011]; 620; 627; 4.422598; ACCCCGCT; 0.08990; 0.10969;
Sequence; ADR1 [T00011]; 691; 698; 6.381348; ACCCCTAG; 0.20229; 0.25206;
Sequence; ADR1 [T00011]; 707; 714; 7.461305; ACCCCCTT; 0.04495; 0.04915;
Sequence; ADR1 [T00011]; 759; 766; 8.260309; ACCCCAGA; 0.17981; 0.24509;
Sequence; Sry-delta [T00767]; 714; 726; 13.356042; TCCCCACCTCTAG; 0.00606; 0.00492;
Sequence; IA-1 [T05887]; 705; 717; 14.700907; TCACCCCCTTCCC; 0.02184; 0.03000;
Sequence; T3R-alpha1 [T01152]; 700; 710; 13.969556; TGACCTCACCC; 0.24443; 0.22991;
Sequence; T3R-beta1 [T00853]; 700; 709; 4.183328; TGACCTCACC; 0.01686; 0.01837;
Sequence; RORalpha1 [T01527]; 700; 709; 6.166756; TGACCTCACC; 0.05479; 0.05980;
Sequence; SF-1 [T04014]; 700; 709; 9.056375; TGACCTCACC; 0.11379; 0.10641;
Sequence; COUP-TF1 [T00149]; 700; 708; 1.292649; TGACCTCAC; 0.11238; 0.10486;
Sequence; T3R-alpha [T01351]; 700; 708; 2.085552; TGACCTCAC; 0.02810; 0.02557;
Sequence; SF-1 [T02769]; 699; 709; 9.064415; CTGACCTCACC; 0.04214; 0.03890;
Sequence; RXR-beta [T01332]; 696; 705; 6.413388; TAGCTGACCT; 0.02529; 0.02380;
Sequence; E4F1 [T00223]; 673; 681; 5.128278; GGGCGTCAC; 0.17981; 0.21682;
Sequence; Sp3 [T02338]; 672; 680; 11.609185; GGGGCGTCA; 0.09552; 0.12921;
Sequence; BTEB4 [T05053]; 672; 680; 4.079815; GGGGCGTCA; 0.06743; 0.10474;
Sequence; Pbx1b [T02088]; 559; 569; 9.987966; CCCTCCCGGCC; 0.05689; 0.07913;
Sequence; PHO4 [T00690]; 543; 554; 13.124936; TCCCACATGCTA; 0.01291; 0.01629;
Sequence; Ttk 69K [T00843]; 495; 502; 13.585908; TGTCCTGC; 0.47200; 0.60786;
Sequence; CTF [T00174]; 485; 496; 10.779118; TGCCAATGTGTG; 0.02845; 0.03271;
Sequence; Oct-B1 [T00545]; 484; 491; 12.500000; ATGCCAAT; 0.53943; 0.40002;
Sequence; POU2F2B [T00662]; 484; 491; 12.500000; ATGCCAAT; 0.53943; 0.40002;
Sequence; POU2F1 [T00959]; 484; 491; 12.500000; ATGCCAAT; 0.53943; 0.40002;
Sequence; POU2F2B [T01871]; 484; 491; 12.500000; ATGCCAAT; 0.53943; 0.40002;
Sequence; POU5F1 (Oct-5) [T00653]; 483; 491; 12.876661; GATGCCAAT; 0.40457; 0.30393;
Sequence; POU3F1 [T00656]; 481; 491; 13.350458; CTGATGCCAAT; 0.25286; 0.20204;
Sequence; POU2F1b [T01862]; 481; 491; 12.607587; CTGATGCCAAT; 0.53943; 0.40002;
Sequence; POU2F1c [T01863]; 481; 491; 12.607587; CTGATGCCAAT; 0.53943; 0.40002;
Sequence; NF-kappaB [T00588]; 431; 440; 4.125075; GGGAAGCACC; 0.01124; 0.01463;
Sequence; AR [T00042]; 399; 405; 3.321435; AGGTTCT; 0.35962; 0.38993;
Sequence; NIT2 [T00627]; 116; 123; 5.523088; TGCAGATA; 0.44952; 0.35353;
Sequence; NIT2 [T00627]; 138; 145; 0.000000; CGGAGATA; 0.11238; 0.09325;
Sequence; NIT2 [T00627]; 389; 396; 5.460984; CTCAGATA; 0.44952; 0.35353;

Sequence; NFe [T00565]; 344; 355; 8.121273; CCGGGTCAGTGA; 0.02213; 0.02026;
Sequence; mtTFA [T04783]; 278; 287; 3.812478; TGATAAAGGA; 0.08850; 0.06079;
Sequence; RPN4 [T04539]; 267; 275; 11.111111; GTTGGCAAA; 0.15171; 0.15308;
Sequence; StuAp [T02248]; 259; 266; 2.687747; CTCCGCGA; 0.35962; 0.44131;
Sequence; POU2F1 [T00643]; 230; 240; 10.591017; TACTCATCATC; 0.05057; 0.04330;
Sequence; Sp1 [T00753]; 12; 17; 0.000000; GGGCGG; 0.35962; 0.57664;
Sequence; Sp1 [T00752]; 11; 20; 2.162070; AGGGCGGAGG; 0.01264; 0.01968;
Sequence; Sp1 [T00754]; 11; 20; 3.289853; AGGGCGGAGG; 0.04636; 0.07111;
Sequence; Egr-1 [T01200]; 9; 21; 8.442026; GGAGGGCGGAGGC; 0.00632; 0.01169;
Sequence; GKLF [T02450]; 7; 19; 13.888051; AGGGAGGGCGGAG; 0.01475; 0.01621;

-- END --

Distribution of the nucleotides over the given chain:

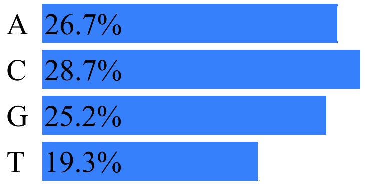

Supplement: Supplementary file 1 [file genes-13-00427-s001.zip › genes-1568895-supplementary/Table S2. Putative Transcription factor binding site on the murine MORG1 promoter sequence..pdf]
