# Supplementary material for: Cloning of the Human MORG1 Promoter: Differential Regulation by Hypoxia and Prolyl-Hydroxylase Inhibitors
Source: Genes (Basel). 2022 Feb 25;13(3):427. doi: 10.3390/genes13030427 (PMC8954370; doi:10.3390/genes13030427)
Supplement: Supplementary file 1 [file genes-13-00427-s001.zip › genes-1568895-supplementary/Table S3. Putative Transcription factor binding site on the human MORG1 promoter sequence..pdf]

Table S3. Putative transcription factor binding sites on the human *MORG1* promoter sequence.

Putative transcription factor binding sites on the human *MORG1* promoter sequence identified by the aid of the ALGGEN-PROMO link (http://alggen.lsi.upc.es/cgi-bin/promo_v3/promo/promoinit.cgi?dirDB=TF_8.3), which use the TFBS defined in the TRANSFAC database to construct specific binding site weight matrices for TFBS prediction.

Factors predicted within a dissimilarity margin less or equal than 15 % :

Data (txt)

-- Input sequence ---
>Sequence
CAGCTGGGGGCACCGAGACGGCCTCATTCAGGGAAGTCCAGGATGGCAGCTGAAGGCAGCAGGTTTAGCAG
CCAAAGCCCAGGCCTAGTGGATAGACAGGGTCCAAAATGTGACCCTTCTAGGCTGGTATCACCATGGGGGCG
TCATGGGCTGAGGATTCTGCAGATAGGACATCACCACGGCAGAGATGGACAGCCTGAGACAGGAGAGAGGT
GTCAGTCTAGGATGGCCAGGCTGGGGTCCCCCCACCCCTTACTCAAGAGTCACTTGTTCTGTAGGGCAAGTCTC
ACATGAAGCTACTCATCATTTGCTTCGTGTCCTCCGAGCTCCGAGAGTTGGCAAAGCTGATGAAGGAGCAGTA
GACAGCGACCCAAGCACACCACTTCAGCTAGGGAAAGGTAAGTGGGTGGGCAGGTTAGTGAAAGGCACAAC
AATGAGTCCCCGAAGCCACCTTTCCAGACACCAGATCATGCCCACTGATTCCATCTGATGCAGCTGGGACCTTG
CCCCACCCGGAATTCCTCAGTCCCACCTGCTCATGGCATACTCTAGATACGTGGTTTCTAGTCCTACTGTCTGGC
CTGCGTCTCTCAGGACCAAGAGAGACTCTGACATCCCACATGCTTAAGACCCTGCCCTTTTTCCACAACTCCTCT
CCAAGTTGCTTCAAAGCCTGGCCCCACTCGCAGACCAATCTGCCACATCCCCAACCTGACTCGAATCCTTGACTT
CACCAAAGACTGGGGCTTTCTCGGCCCAGCGTCATCGCAGCCCCACTCCCGGCCGATCTCAGGTCCCGCCCCAT
CAGCAATGACAAGACACCCCGCTTCATTCCAAACCCGCCCCCGGGCCTCGTTCTCAGGTCCAACTACACCCATA
GAAGCTCAACCCCGCCCACCTTAAGCATGAGGCCGCACATGCTGAAGATCATGCCCAGCAGGTTCATGTAGTC
CGGCGTCGGGTCGTCCAAGGCCGGGTTACATTCGCTCGGCGGGGGCTTGTACCTGCGACAGGCTCGAGGGT
CAGGGGCGCTCAGGTCCTGCCCCCGGGATAGACAGGCGCTTCCCAGGGCCCCGATCCACACCCACAACCCGA
ACCCGTGCCCACGACGCTGGGGTCCTCACCTCAGCACTTTGTTCGGCCTCCGTGGGTCCGACATATTGTTAGTG
GACATAGCGAGTCGAAGGCCAGATCACGCCTCTTCCGCTGCAGGAATCGCAGCTTCCGGCGTGCAAGCTGAG
GGCGGATTTTAGAGTAACACCCGAGGCCCTCGCATTTCCGTTCCTCATGACAGAGGCTTGGACTCCCCTTACCC
AGAGAACGGAGATTTAGGAGAAGCCAGAAGTCTTCCTTTCAAAGGGAAAATGGGGAAAAGAACCTGAAAGC
GGGCTTCAATAGTTCCAACCCGATCACCGAAATGCCAAAATGATGAACCCGGAAGTGATAAACAGGAAGTAG
GTCAGGAAGAACAATAATAAGGTTACACCCAAGCGTGGGTTTCTAAGGCGCGGAATTTTCCGTACAGACCGAT
TTAAGGCTGCAAGGAAGGAGTCCTGGGAGCATGGCTTTCCCTGAGC

-- Factors predicted by PROMO in this sequence ---------------------
NAME; MATRIX_WIDTH;
E47 [T00207]; 7
Tal-1 [T00790]; 11
ETF [T00270]; 9
HSF1 (long) [T01042]; 8
HSF1 (short) [T02104]; 8
STAT5A [T04683]; 4
Elk-1 [T00250]; 5
STAT3 [T01493]; 12
p300 [T01427]; 5
R2 [T00712]; 6

YY1 [T00915]; 7
PEA3 [T00685]; 8
ENKTF-1 [T00255]; 8
Myf-3 [T00519]; 8
MyoD [T00525]; 8
AP-2alphaA [T00035]; 6
TCF-1A [T00999]; 9
LEF-1 [T02905]; 8
TCF-4E [T02878]; 7
NFI/CTF [T00094]; 8
p53 [T00671]; 7
GATA-1 [T00306]; 6
VDR [T00885]; 4
MBF1 [T00492]; 11
POU3F2 [T00630]; 7
FOXP3 [T04280]; 9
USF2b [T02377]; 8
USF2 [T00878]; 9
c-Jun [T00133]; 7
NHP-1 [T00621]; 7
ARP-1 [T00045]; 9
RAR-gamma [T00720]; 8
c-Fos [T00123]; 8
Pax-2 [T01823]; 7
Nkx2-1 [T00857]; 7
NF-X3 [T01514]; 8
GATA-3 [T00311]; 8
TGIF [T04076]; 8
PKNOX1 [T04122]; 11
FOXO3a [T02938]; 8
Hlf [T01071]; 9
AP-1 [T00029]; 8
PR B [T00696]; 7
PR A [T01661]; 7
GR-alpha [T00337]; 8
GR-beta [T01920]; 8
GR [T05076]; 13
LCR-F1 [T01599]; 6
Oct-B1 [T00545]; 8
POU2F2 (Oct-2.1) [T00646]; 8
POU2F2B [T00662]; 8
C/EBPalpha [T00105]; 6
C/EBPbeta [T00581]; 7
FOXN2 [T04206]; 11
NF-1 [T00539]; 5
IRF-1 [T00423]; 9
IRF-3 [T04673]; 7
NF-AT1 [T00550]; 9
WT1 I [T01840]; 7
Pax-6 [T01122]; 11
SRY [T00997]; 7
NF-AT2 [T01945]; 9
NF-AT1 [T01948]; 8

HMG I(Y) [T02368]; 7
STAT4 [T01577]; 6
c-Ets-1 [T00112]; 7
STAT1beta [T01573]; 10
Smad3 [T04096]; 10
ANF [T00025]; 12
Smad4 [T04292]; 11
EIIaE-A [T00246]; 7
LF-A1 [T00467]; 7
POU2F2C [T00665]; 8
AP-4 [T00036]; 11
Myf-5 [T00521]; 13
Sp3 [T02338]; 9
TFIIB [T00818]; 8
AREB6 [T00625]; 8
Ik-1 [T02702]; 13
E12 [T00204]; 13
POU6F1 [T04470]; 11
Max [T05056]; 11
AIRE [T05990]; 10
Nrf2 [T01443]; 11
NF-AT4 [T01946]; 12
Pu box binding factor [T00704]; 10
NF-AT3 [T02462]; 10
AML1 [T01067]; 13
c-Myb [T00137]; 8
BTEB3 [T05051]; 9
NF-Y [T00150]; 8
PPAR-alpha:RXR-alpha [T05221]; 11
T3R-alpha1 [T01152]; 11
DP-1 [T01548]; 10
E2F-1 [T01542]; 7
E2F-1:DP-1 [T05204]; 9
GCF [T00320]; 11
MAZ [T00490]; 11
WT1 [T00899]; 11
Egr-3 [T00243]; 13
Pbx1 [T06000]; 12
GCMa [T02306]; 9
PXR-1:RXR-alpha [T05671]; 11
Sp1 [T00759]; 9
WT1 I -KTS [T00900]; 10
WT1 -KTS [T01839]; 10
ER-beta [T04651]; 9
RAR-beta [T00721]; 8
ER-alpha [T00261]; 8
RelA [T00594]; 10
EBF [T05427]; 11
RAR-beta:RXR-alpha [T05420]; 12
USF1 [T00874]; 9
Pax-5 [T00070]; 7
ERRalpha1 [T05682]; 13
HNF-4alpha [T03828]; 13

CUTL1 [T00100]; 6
AhR:Arnt [T05394]; 9
c-Ets-2 [T00113]; 8
Arnt [T01346]; 9
AhR [T01795]; 8
MIF-1 [T01047]; 13
SXR:RXR-alpha [T05670]; 9
MZF-1 [T00529]; 7
GABP-alpha [T01390]; 12
GABP [T00268]; 12
CDX2 [T03246]; 12
IRF-2 [T01491]; 11
FOXJ2 (long isoform) [T04169]; 10
HNF-3beta [T02513]; 7
NERF-1a [T05021]; 12
RORalpha2 [T01528]; 12
RORalpha1 [T01527]; 10
COUP-TF1 [T00149]; 9
SF-1 [T02769]; 11
AR [T00040]; 8
HOXD9 [T01424]; 10
HOXD10 [T01425]; 10
AP-3 (2) [T00039]; 7
NF-kappaB [T00590]; 11
E2F-5 [T01607]; 8
HNF-1B [T01950]; 8
RAR-alpha1 [T00719]; 10
RXR-alpha [T01345]; 9
ELF-1 [T01113]; 13
Pbx1b [T02087]; 10
PU.1 [T02068]; 10
R1 [T00711]; 12
TFII-I [T00824]; 11
T3R-beta1 [T00851]; 11
CRF [T00170]; 12
RFX1 [T01673]; 9
POU2F1 [T00641]; 10
POU2F2 [T00647]; 11
octamer-binding factor [T01225]; 11

-- PROMO predictions detail --

Sequence name; Factor name; Start position; End position; Dissimilarity; String; RE equally; RE query
Sequence; E47 [T00207]; 1; 7; 4.682492; AGCTGGG; 0.77002; 1.02300;
Sequence; E47 [T00207]; 36; 42; 5.965250; TCCAGGA; 0.48126; 0.59997;
Sequence; E47 [T00207]; 77; 83; 5.305872; CCCAGGC; 0.67377; 0.86109;
Sequence; E47 [T00207]; 121; 127; 4.502284; GGCTGGT; 0.77002; 1.02300;
Sequence; E47 [T00207]; 228; 234; 4.258142; GCCAGGC; 0.77002; 1.02300;
Sequence; E47 [T00207]; 232; 238; 4.851527; GGCTGGG; 0.96252; 1.18255;
Sequence; E47 [T00207]; 454; 460; 0.692900; TCCAGAC; 0.28876; 0.26117;
Sequence; E47 [T00207]; 461; 467; 0.698487; ACCAGAT; 0.28876; 0.26117;
Sequence; E47 [T00207]; 493; 499; 4.682492; AGCTGGG; 0.77002; 1.02300;
Sequence; E47 [T00207]; 574; 580; 0.169035; GTCTGGC; 0.19250; 0.23772;

Sequence; E47 [T00207]; 671; 677; 4.258142; GCCTGGC; 0.77002; 1.02300;
Sequence; E47 [T00207]; 738; 744; 5.533045; GACTGGG; 0.67377; 0.86109;
Sequence; E47 [T00207]; 755; 761; 6.879944; CCCAGCG; 0.48126; 0.64340;
Sequence; E47 [T00207]; 932; 938; 6.034770; CCCAGCA; 0.48126; 0.59997;
Sequence; E47 [T00207]; 1064; 1070; 7.334289; CCCAGGG; 0.19250; 0.30408;
Sequence; E47 [T00207]; 1109; 1115; 6.879944; CGCTGGG; 0.48126; 0.64340;
Sequence; E47 [T00207]; 1186; 1192; 0.000000; GCCAGAT; 0.19250; 0.23772;
Sequence; E47 [T00207]; 1312; 1318; 3.245181; CCCAGAG; 0.19250; 0.24211;
Sequence; E47 [T00207]; 1337; 1343; 1.352278; GCCAGAA; 0.09625; 0.10552;
Sequence; E47 [T00207]; 1551; 1557; 6.489116; TCCTGGG; 0.67377; 0.89212;
Sequence; Tal-1 [T00790]; 0; 10; 12.618019; CAGCTGGGGGC; 0.08121; 0.09181;
Sequence; Tal-1 [T00790]; 478; 488; 12.618019; GATTCCATCTG; 0.08121; 0.09181;
Sequence; Tal-1 [T00790]; 492; 502; 13.546753; CAGCTGGGACC; 0.10152; 0.11094;
Sequence; Tal-1 [T00790]; 524; 534; 13.546753; AGTCCCACCTG; 0.10152; 0.11094;
Sequence; ETF [T00270]; 6; 14; 8.217998; GGGGCACCG; 0.21657; 0.41104;
Sequence; ETF [T00270]; 137; 145; 6.076766; GGGGCGTCA; 0.16243; 0.29885;
Sequence; ETF [T00270]; 501; 509; 7.587476; CCTTGCCCC; 0.03609; 0.08254;
Sequence; ETF [T00270]; 672; 680; 7.587476; CCTGGCCCC; 0.03609; 0.08254;
Sequence; ETF [T00270]; 742; 750; 7.000683; GGGGCTTTC; 0.16243; 0.23040;
Sequence; ETF [T00270]; 766; 774; 1.554439; CGCAGCCCC; 0.09024; 0.19704;
Sequence; ETF [T00270]; 794; 802; 0.630521; TCCCGCCCC; 0.03609; 0.09846;
Sequence; ETF [T00270]; 837; 845; 0.630521; ACCCGCCCC; 0.03609; 0.09846;
Sequence; ETF [T00270]; 993; 1001; 6.370162; GGGGCTTGT; 0.10828; 0.18666;
Sequence; ETF [T00270]; 1025; 1033; 6.076766; GGGGCGCTC; 0.16243; 0.29885;
Sequence; ETF [T00270]; 1037; 1045; 3.402275; TCCTGCCCC; 0.07219; 0.16509;
Sequence; ETF [T00270]; 1066; 1074; 8.217998; CAGGGCCCC; 0.21657; 0.41104;
Sequence; HSF1 (long) [T01042]; 25; 32; 7.272359; ATTCAGGG; 0.14438; 0.14291;
Sequence; HSF1 (long) [T01042]; 479; 486; 10.655793; ATTCCATC; 0.79408; 0.50558;
Sequence; HSF1 (long) [T01042]; 511; 518; 7.272359; CCCGGAAT; 0.14438; 0.14291;
Sequence; HSF1 (long) [T01042]; 714; 721; 5.327897; ACTCGAAT; 0.43314; 0.30695;
Sequence; HSF1 (long) [T01042]; 830; 837; 10.655793; ATTCCAAA; 0.79408; 0.50558;
Sequence; HSF1 (long) [T01042]; 981; 988; 14.291973; ATTCGCTC; 0.64970; 0.51288;
Sequence; HSF1 (long) [T01042]; 1208; 1215; 12.600256; GCAGGAAT; 0.86627; 0.71289;
Sequence; HSF1 (long) [T01042]; 1506; 1513; 12.600256; CGCGGAAT; 0.86627; 0.71289;
Sequence; HSF1 (short) [T02104]; 25; 32; 7.272359; ATTCAGGG; 0.14438; 0.14291;
Sequence; HSF1 (short) [T02104]; 479; 486; 10.655793; ATTCCATC; 0.79408; 0.50558;
Sequence; HSF1 (short) [T02104]; 511; 518; 7.272359; CCCGGAAT; 0.14438; 0.14291;
Sequence; HSF1 (short) [T02104]; 714; 721; 5.327897; ACTCGAAT; 0.43314; 0.30695;
Sequence; HSF1 (short) [T02104]; 830; 837; 10.655793; ATTCCAAA; 0.79408; 0.50558;
Sequence; HSF1 (short) [T02104]; 981; 988; 14.291973; ATTCGCTC; 0.64970; 0.51288;
Sequence; HSF1 (short) [T02104]; 1208; 1215; 12.600256; GCAGGAAT; 0.86627; 0.71289;
Sequence; HSF1 (short) [T02104]; 1506; 1513; 12.600256; CGCGGAAT; 0.86627; 0.71289;
Sequence; STAT5A [T04683]; 31; 34; 0.000000; GGAA; 6.16016; 5.86404;
Sequence; STAT5A [T04683]; 392; 395; 0.000000; GGAA; 6.16016; 5.86404;
Sequence; STAT5A [T04683]; 453; 456; 0.000000; TTCC; 6.16016; 5.86404;
Sequence; STAT5A [T04683]; 480; 483; 0.000000; TTCC; 6.16016; 5.86404;
Sequence; STAT5A [T04683]; 514; 517; 0.000000; GGAA; 6.16016; 5.86404;
Sequence; STAT5A [T04683]; 518; 521; 0.000000; TTCC; 6.16016; 5.86404;
Sequence; STAT5A [T04683]; 641; 644; 0.000000; TTCC; 6.16016; 5.86404;
Sequence; STAT5A [T04683]; 831; 834; 0.000000; TTCC; 6.16016; 5.86404;
Sequence; STAT5A [T04683]; 1062; 1065; 0.000000; TTCC; 6.16016; 5.86404;
Sequence; STAT5A [T04683]; 1201; 1204; 0.000000; TTCC; 6.16016; 5.86404;
Sequence; STAT5A [T04683]; 1211; 1214; 0.000000; GGAA; 6.16016; 5.86404;

Sequence; STAT5A [T04683]; 1222; 1225; 0.000000; TTCC; 6.16016; 5.86404;
Sequence; STAT5A [T04683]; 1276; 1279; 0.000000; TTCC; 6.16016; 5.86404;
Sequence; STAT5A [T04683]; 1281; 1284; 0.000000; TTCC; 6.16016; 5.86404;
Sequence; STAT5A [T04683]; 1347; 1350; 0.000000; TTCC; 6.16016; 5.86404;
Sequence; STAT5A [T04683]; 1359; 1362; 0.000000; GGAA; 6.16016; 5.86404;
Sequence; STAT5A [T04683]; 1368; 1371; 0.000000; GGAA; 6.16016; 5.86404;
Sequence; STAT5A [T04683]; 1398; 1401; 0.000000; TTCC; 6.16016; 5.86404;
Sequence; STAT5A [T04683]; 1436; 1439; 0.000000; GGAA; 6.16016; 5.86404;
Sequence; STAT5A [T04683]; 1450; 1453; 0.000000; GGAA; 6.16016; 5.86404;
Sequence; STAT5A [T04683]; 1462; 1465; 0.000000; GGAA; 6.16016; 5.86404;
Sequence; STAT5A [T04683]; 1509; 1512; 0.000000; GGAA; 6.16016; 5.86404;
Sequence; STAT5A [T04683]; 1515; 1518; 0.000000; TTCC; 6.16016; 5.86404;
Sequence; STAT5A [T04683]; 1543; 1546; 0.000000; GGAA; 6.16016; 5.86404;
Sequence; STAT5A [T04683]; 1567; 1570; 0.000000; TTCC; 6.16016; 5.86404;
Sequence; Elk-1 [T00250]; 31; 35; 0.000000; GGAAG; 1.54004; 1.65111;
Sequence; Elk-1 [T00250]; 34; 38; 6.853012; AGTCC; 4.62012; 4.61150;
Sequence; Elk-1 [T00250]; 40; 44; 3.960472; GGATG; 3.08008; 3.84960;
Sequence; Elk-1 [T00250]; 89; 93; 6.081668; GGATA; 3.08008; 2.93146;
Sequence; Elk-1 [T00250]; 99; 103; 6.274504; GGTCC; 3.08008; 3.82271;
Sequence; Elk-1 [T00250]; 154; 158; 6.853012; GGATT; 4.62012; 4.61150;
Sequence; Elk-1 [T00250]; 168; 172; 6.081668; GGACA; 3.08008; 2.93146;
Sequence; Elk-1 [T00250]; 189; 193; 6.081668; GGACA; 3.08008; 2.93146;
Sequence; Elk-1 [T00250]; 204; 208; 7.213232; GGAGA; 4.62012; 4.61150;
Sequence; Elk-1 [T00250]; 223; 227; 3.960472; GGATG; 3.08008; 3.84960;
Sequence; Elk-1 [T00250]; 238; 242; 6.274504; GGTCC; 3.08008; 3.82271;
Sequence; Elk-1 [T00250]; 315; 319; 6.081668; TGTCC; 3.08008; 2.93146;
Sequence; Elk-1 [T00250]; 318; 322; 5.092035; CCTCC; 1.54004; 2.19561;
Sequence; Elk-1 [T00250]; 325; 329; 7.406067; GCTCC; 1.54004; 2.15767;
Sequence; Elk-1 [T00250]; 352; 356; 7.406067; GGAGC; 1.54004; 2.15767;
Sequence; Elk-1 [T00250]; 392; 396; 2.121196; GGAAA; 1.54004; 1.28146;
Sequence; Elk-1 [T00250]; 436; 440; 6.853012; AGTCC; 4.62012; 4.61150;
Sequence; Elk-1 [T00250]; 452; 456; 2.121196; TTTCC; 1.54004; 1.28146;
Sequence; Elk-1 [T00250]; 479; 483; 2.892540; ATTCC; 1.54004; 1.26569;
Sequence; Elk-1 [T00250]; 498; 502; 6.274504; GGACC; 3.08008; 3.82271;
Sequence; Elk-1 [T00250]; 514; 518; 2.892540; GGAAT; 1.54004; 1.26569;
Sequence; Elk-1 [T00250]; 517; 521; 2.892540; ATTCC; 1.54004; 1.26569;
Sequence; Elk-1 [T00250]; 524; 528; 6.853012; AGTCC; 4.62012; 4.61150;
Sequence; Elk-1 [T00250]; 565; 569; 6.853012; AGTCC; 4.62012; 4.61150;
Sequence; Elk-1 [T00250]; 593; 597; 6.274504; GGACC; 3.08008; 3.82271;
Sequence; Elk-1 [T00250]; 612; 616; 3.960472; CATCC; 3.08008; 3.84960;
Sequence; Elk-1 [T00250]; 640; 644; 2.121196; TTTCC; 1.54004; 1.28146;
Sequence; Elk-1 [T00250]; 648; 652; 7.984576; ACTCC; 1.54004; 1.69193;
Sequence; Elk-1 [T00250]; 653; 657; 7.213232; TCTCC; 4.62012; 4.61150;
Sequence; Elk-1 [T00250]; 701; 705; 3.960472; CATCC; 3.08008; 3.84960;
Sequence; Elk-1 [T00250]; 719; 723; 6.853012; AATCC; 4.62012; 4.61150;
Sequence; Elk-1 [T00250]; 775; 779; 7.984576; ACTCC; 1.54004; 1.69193;
Sequence; Elk-1 [T00250]; 792; 796; 6.274504; GGTCC; 3.08008; 3.82271;
Sequence; Elk-1 [T00250]; 830; 834; 2.892540; ATTCC; 1.54004; 1.26569;
Sequence; Elk-1 [T00250]; 861; 865; 6.274504; GGTCC; 3.08008; 3.82271;
Sequence; Elk-1 [T00250]; 948; 952; 6.853012; AGTCC; 4.62012; 4.61150;
Sequence; Elk-1 [T00250]; 963; 967; 3.960472; CGTCC; 3.08008; 3.84960;
Sequence; Elk-1 [T00250]; 1035; 1039; 6.274504; GGTCC; 3.08008; 3.82271;
Sequence; Elk-1 [T00250]; 1048; 1052; 6.081668; GGATA; 3.08008; 2.93146;

Sequence; Elk-1 [T00250]; 1061; 1065; 0.000000; CTTCC; 1.54004; 1.65111;
Sequence; Elk-1 [T00250]; 1075; 1079; 6.274504; GATCC; 3.08008; 3.82271;
Sequence; Elk-1 [T00250]; 1115; 1119; 6.274504; GGTCC; 3.08008; 3.82271;
Sequence; Elk-1 [T00250]; 1141; 1145; 5.092035; CCTCC; 1.54004; 2.19561;
Sequence; Elk-1 [T00250]; 1149; 1153; 6.274504; GGTCC; 3.08008; 3.82271;
Sequence; Elk-1 [T00250]; 1168; 1172; 6.081668; GGACA; 3.08008; 2.93146;
Sequence; Elk-1 [T00250]; 1200; 1204; 0.000000; CTTCC; 1.54004; 1.65111;
Sequence; Elk-1 [T00250]; 1211; 1215; 2.892540; GGAAT; 1.54004; 1.26569;
Sequence; Elk-1 [T00250]; 1221; 1225; 0.000000; CTTCC; 1.54004; 1.65111;
Sequence; Elk-1 [T00250]; 1244; 1248; 6.853012; GGATT; 4.62012; 4.61150;
Sequence; Elk-1 [T00250]; 1275; 1279; 2.121196; TTTCC; 1.54004; 1.28146;
Sequence; Elk-1 [T00250]; 1280; 1284; 2.314032; GTTCC; 1.54004; 1.66577;
Sequence; Elk-1 [T00250]; 1300; 1304; 6.853012; GGACT; 4.62012; 4.61150;
Sequence; Elk-1 [T00250]; 1302; 1306; 7.984576; ACTCC; 1.54004; 1.69193;
Sequence; Elk-1 [T00250]; 1322; 1326; 7.213232; GGAGA; 4.62012; 4.61150;
Sequence; Elk-1 [T00250]; 1331; 1335; 7.213232; GGAGA; 4.62012; 4.61150;
Sequence; Elk-1 [T00250]; 1346; 1350; 0.000000; CTTCC; 1.54004; 1.65111;
Sequence; Elk-1 [T00250]; 1359; 1363; 2.121196; GGAAA; 1.54004; 1.28146;
Sequence; Elk-1 [T00250]; 1368; 1372; 2.121196; GGAAA; 1.54004; 1.28146;
Sequence; Elk-1 [T00250]; 1397; 1401; 2.314032; GTTCC; 1.54004; 1.66577;
Sequence; Elk-1 [T00250]; 1436; 1440; 0.000000; GGAAG; 1.54004; 1.65111;
Sequence; Elk-1 [T00250]; 1450; 1454; 0.000000; GGAAG; 1.54004; 1.65111;
Sequence; Elk-1 [T00250]; 1462; 1466; 0.000000; GGAAG; 1.54004; 1.65111;
Sequence; Elk-1 [T00250]; 1509; 1513; 2.892540; GGAAT; 1.54004; 1.26569;
Sequence; Elk-1 [T00250]; 1514; 1518; 2.121196; TTTCC; 1.54004; 1.28146;
Sequence; Elk-1 [T00250]; 1543; 1547; 0.000000; GGAAG; 1.54004; 1.65111;
Sequence; Elk-1 [T00250]; 1547; 1551; 7.984576; GGAGT; 1.54004; 1.69193;
Sequence; Elk-1 [T00250]; 1549; 1553; 6.853012; AGTCC; 4.62012; 4.61150;
Sequence; Elk-1 [T00250]; 1556; 1560; 7.406067; GGAGC; 1.54004; 2.15767;
Sequence; Elk-1 [T00250]; 1566; 1570; 2.121196; TTTCC; 1.54004; 1.28146;
Sequence; STAT3 [T01493]; 25; 36; 13.050354; ATTCAGGGAAGT; 0.03158; 0.03032;
Sequence; p300 [T01427]; 31; 35; 5.408046; GGAAG; 3.08008; 2.93257;
Sequence; p300 [T01427]; 34; 38; 0.000000; AGTCC; 1.54004; 1.65548;
Sequence; p300 [T01427]; 40; 44; 4.252471; GGATG; 4.62012; 4.61311;
Sequence; p300 [T01427]; 89; 93; 4.252471; GGATA; 4.62012; 4.61311;
Sequence; p300 [T01427]; 99; 103; 3.099092; GGTCC; 1.54004; 2.16723;
Sequence; p300 [T01427]; 154; 158; 1.540766; GGATT; 1.54004; 1.30491;
Sequence; p300 [T01427]; 168; 172; 2.711705; GGACA; 4.62012; 5.08913;
Sequence; p300 [T01427]; 189; 193; 2.711705; GGACA; 4.62012; 5.08913;
Sequence; p300 [T01427]; 204; 208; 3.482089; GGAGA; 3.08008; 3.84672;
Sequence; p300 [T01427]; 223; 227; 4.252471; GGATG; 4.62012; 4.61311;
Sequence; p300 [T01427]; 238; 242; 3.099092; GGTCC; 1.54004; 2.16723;
Sequence; p300 [T01427]; 315; 319; 2.711705; TGTCC; 4.62012; 5.08913;
Sequence; p300 [T01427]; 318; 322; 3.482089; CCTCC; 3.08008; 3.84672;
Sequence; p300 [T01427]; 325; 329; 3.869475; GCTCC; 1.54004; 2.15767;
Sequence; p300 [T01427]; 352; 356; 3.869475; GGAGC; 1.54004; 2.15767;
Sequence; p300 [T01427]; 392; 396; 5.408046; GGAAA; 3.08008; 2.93257;
Sequence; p300 [T01427]; 436; 440; 0.000000; AGTCC; 1.54004; 1.65548;
Sequence; p300 [T01427]; 452; 456; 5.408046; TTTCC; 3.08008; 2.93257;
Sequence; p300 [T01427]; 479; 483; 2.696340; ATTCC; 4.62012; 5.08913;
Sequence; p300 [T01427]; 498; 502; 3.099092; GGACC; 1.54004; 2.16723;
Sequence; p300 [T01427]; 514; 518; 2.696340; GGAAT; 4.62012; 5.08913;
Sequence; p300 [T01427]; 517; 521; 2.696340; ATTCC; 4.62012; 5.08913;

Sequence; p300 [T01427]; 524; 528; 0.000000; AGTCC; 1.54004; 1.65548;
Sequence; p300 [T01427]; 565; 569; 0.000000; AGTCC; 1.54004; 1.65548;
Sequence; p300 [T01427]; 593; 597; 3.099092; GGACC; 1.54004; 2.16723;
Sequence; p300 [T01427]; 612; 616; 4.252471; CATCC; 4.62012; 4.61311;
Sequence; p300 [T01427]; 640; 644; 5.408046; TTTCC; 3.08008; 2.93257;
Sequence; p300 [T01427]; 648; 652; 0.770383; ACTCC; 1.54004; 1.69193;
Sequence; p300 [T01427]; 653; 657; 3.482089; TCTCC; 3.08008; 3.84672;
Sequence; p300 [T01427]; 701; 705; 4.252471; CATCC; 4.62012; 4.61311;
Sequence; p300 [T01427]; 719; 723; 1.540766; AATCC; 1.54004; 1.30491;
Sequence; p300 [T01427]; 775; 779; 0.770383; ACTCC; 1.54004; 1.69193;
Sequence; p300 [T01427]; 792; 796; 3.099092; GGTCC; 1.54004; 2.16723;
Sequence; p300 [T01427]; 830; 834; 2.696340; ATTCC; 4.62012; 5.08913;
Sequence; p300 [T01427]; 861; 865; 3.099092; GGTCC; 1.54004; 2.16723;
Sequence; p300 [T01427]; 948; 952; 0.000000; AGTCC; 1.54004; 1.65548;
Sequence; p300 [T01427]; 963; 967; 2.711705; CGTCC; 4.62012; 5.08913;
Sequence; p300 [T01427]; 1035; 1039; 3.099092; GGTCC; 1.54004; 2.16723;
Sequence; p300 [T01427]; 1048; 1052; 4.252471; GGATA; 4.62012; 4.61311;
Sequence; p300 [T01427]; 1061; 1065; 5.408046; CTTCC; 3.08008; 2.93257;
Sequence; p300 [T01427]; 1075; 1079; 4.639858; GATCC; 4.62012; 4.61311;
Sequence; p300 [T01427]; 1115; 1119; 3.099092; GGTCC; 1.54004; 2.16723;
Sequence; p300 [T01427]; 1141; 1145; 3.482089; CCTCC; 3.08008; 3.84672;
Sequence; p300 [T01427]; 1149; 1153; 3.099092; GGTCC; 1.54004; 2.16723;
Sequence; p300 [T01427]; 1168; 1172; 2.711705; GGACA; 4.62012; 5.08913;
Sequence; p300 [T01427]; 1200; 1204; 5.408046; CTTCC; 3.08008; 2.93257;
Sequence; p300 [T01427]; 1211; 1215; 2.696340; GGAAT; 4.62012; 5.08913;
Sequence; p300 [T01427]; 1221; 1225; 5.408046; CTTCC; 3.08008; 2.93257;
Sequence; p300 [T01427]; 1244; 1248; 1.540766; GGATT; 1.54004; 1.30491;
Sequence; p300 [T01427]; 1275; 1279; 5.408046; TTTCC; 3.08008; 2.93257;
Sequence; p300 [T01427]; 1280; 1284; 5.795432; GTTCC; 1.54004; 1.66577;
Sequence; p300 [T01427]; 1300; 1304; 0.000000; GGACT; 1.54004; 1.65548;
Sequence; p300 [T01427]; 1302; 1306; 0.770383; ACTCC; 1.54004; 1.69193;
Sequence; p300 [T01427]; 1322; 1326; 3.482089; GGAGA; 3.08008; 3.84672;
Sequence; p300 [T01427]; 1331; 1335; 3.482089; GGAGA; 3.08008; 3.84672;
Sequence; p300 [T01427]; 1346; 1350; 5.408046; CTTCC; 3.08008; 2.93257;
Sequence; p300 [T01427]; 1359; 1363; 5.408046; GGAAA; 3.08008; 2.93257;
Sequence; p300 [T01427]; 1368; 1372; 5.408046; GGAAA; 3.08008; 2.93257;
Sequence; p300 [T01427]; 1397; 1401; 5.795432; GTTCC; 1.54004; 1.66577;
Sequence; p300 [T01427]; 1436; 1440; 5.408046; GGAAG; 3.08008; 2.93257;
Sequence; p300 [T01427]; 1450; 1454; 5.408046; GGAAG; 3.08008; 2.93257;
Sequence; p300 [T01427]; 1462; 1466; 5.408046; GGAAG; 3.08008; 2.93257;
Sequence; p300 [T01427]; 1509; 1513; 2.696340; GGAAT; 4.62012; 5.08913;
Sequence; p300 [T01427]; 1514; 1518; 5.408046; TTTCC; 3.08008; 2.93257;
Sequence; p300 [T01427]; 1543; 1547; 5.408046; GGAAG; 3.08008; 2.93257;
Sequence; p300 [T01427]; 1547; 1551; 0.770383; GGAGT; 1.54004; 1.69193;
Sequence; p300 [T01427]; 1549; 1553; 0.000000; AGTCC; 1.54004; 1.65548;
Sequence; p300 [T01427]; 1556; 1560; 3.869475; GGAGC; 1.54004; 2.15767;
Sequence; p300 [T01427]; 1566; 1570; 5.408046; TTTCC; 3.08008; 2.93257;
Sequence; R2 [T00712]; 28; 33; 14.269340; CAGGGA; 4.62012; 4.83284;
Sequence; R2 [T00712]; 36; 41; 11.337888; TCCAGG; 1.92505; 2.36787;
Sequence; R2 [T00712]; 86; 91; 9.512894; AGTGGA; 5.77515; 6.05401;
Sequence; R2 [T00712]; 101; 106; 4.756447; TCCAAA; 2.31006; 2.52600;
Sequence; R2 [T00712]; 151; 156; 14.269340; TGAGGA; 4.62012; 4.83284;
Sequence; R2 [T00712]; 186; 191; 0.000000; GATGGA; 1.15503; 1.22117;

Sequence; R2 [T00712]; 240; 245; 4.756447; TCCCCC; 2.31006; 2.52600;
Sequence; R2 [T00712]; 317; 322; 9.512894; TCCTCC; 5.77515; 6.05401;
Sequence; R2 [T00712]; 349; 354; 9.512894; GAAGGA; 5.77515; 6.05401;
Sequence; R2 [T00712]; 389; 394; 9.512894; TAGGGA; 5.77515; 6.05401;
Sequence; R2 [T00712]; 438; 443; 14.269340; TCCCCG; 4.62012; 4.83284;
Sequence; R2 [T00712]; 454; 459; 6.581441; TCCAGA; 0.77002; 0.97452;
Sequence; R2 [T00712]; 481; 486; 0.000000; TCCATC; 1.15503; 1.22117;
Sequence; R2 [T00712]; 495; 500; 14.269340; CTGGGA; 4.62012; 4.83284;
Sequence; R2 [T00712]; 519; 524; 14.269340; TCCTCA; 4.62012; 4.83284;
Sequence; R2 [T00712]; 526; 531; 4.756447; TCCCAC; 2.31006; 2.52600;
Sequence; R2 [T00712]; 567; 572; 9.512894; TCCTAC; 5.77515; 6.05401;
Sequence; R2 [T00712]; 614; 619; 4.756447; TCCCAC; 2.31006; 2.52600;
Sequence; R2 [T00712]; 642; 647; 4.756447; TCCACA; 2.31006; 2.52600;
Sequence; R2 [T00712]; 655; 660; 9.512894; TCCAAG; 5.77515; 6.05401;
Sequence; R2 [T00712]; 703; 708; 9.512894; TCCCCA; 5.77515; 6.05401;
Sequence; R2 [T00712]; 794; 799; 6.581441; TCCCGC; 0.77002; 0.97452;
Sequence; R2 [T00712]; 832; 837; 4.756447; TCCAAA; 2.31006; 2.52600;
Sequence; R2 [T00712]; 863; 868; 0.000000; TCCAAC; 1.15503; 1.22117;
Sequence; R2 [T00712]; 950; 955; 11.337888; TCCGGC; 1.92505; 2.36787;
Sequence; R2 [T00712]; 965; 970; 9.512894; TCCAAG; 5.77515; 6.05401;
Sequence; R2 [T00712]; 1037; 1042; 11.337888; TCCTGC; 1.92505; 2.36787;
Sequence; R2 [T00712]; 1063; 1068; 14.269340; TCCCAG; 4.62012; 4.83284;
Sequence; R2 [T00712]; 1077; 1082; 4.756447; TCCACA; 2.31006; 2.52600;
Sequence; R2 [T00712]; 1117; 1122; 14.269340; TCCTCA; 4.62012; 4.83284;
Sequence; R2 [T00712]; 1151; 1156; 9.512894; TCCGAC; 5.77515; 6.05401;
Sequence; R2 [T00712]; 1165; 1170; 9.512894; AGTGGA; 5.77515; 6.05401;
Sequence; R2 [T00712]; 1208; 1213; 11.337888; GCAGGA; 1.92505; 2.36787;
Sequence; R2 [T00712]; 1223; 1228; 11.337888; TCCGGC; 1.92505; 2.36787;
Sequence; R2 [T00712]; 1241; 1246; 9.512894; GGCGGA; 5.77515; 6.05401;
Sequence; R2 [T00712]; 1282; 1287; 14.269340; TCCTCA; 4.62012; 4.83284;
Sequence; R2 [T00712]; 1297; 1302; 9.512894; CTTGGA; 5.77515; 6.05401;
Sequence; R2 [T00712]; 1304; 1309; 14.269340; TCCCCT; 4.62012; 4.83284;
Sequence; R2 [T00712]; 1328; 1333; 14.269340; TTAGGA; 4.62012; 4.83284;
Sequence; R2 [T00712]; 1356; 1361; 14.269340; AAGGGA; 4.62012; 4.83284;
Sequence; R2 [T00712]; 1365; 1370; 9.512894; TGGGGA; 5.77515; 6.05401;
Sequence; R2 [T00712]; 1399; 1404; 0.000000; TCCAAC; 1.15503; 1.22117;
Sequence; R2 [T00712]; 1516; 1521; 14.269340; TCCGTA; 4.62012; 4.83284;
Sequence; R2 [T00712]; 1544; 1549; 9.512894; GAAGGA; 5.77515; 6.05401;
Sequence; R2 [T00712]; 1553; 1558; 14.269340; CTGGGA; 4.62012; 4.83284;
Sequence; R2 [T00712]; 1568; 1573; 14.269340; TCCCTG; 4.62012; 4.83284;
Sequence; YY1 [T00915]; 39; 45; 5.043792; AGGATGG; 0.77002; 0.71592;
Sequence; YY1 [T00915]; 131; 137; 5.177063; ACCATGG; 0.77002; 0.71592;
Sequence; YY1 [T00915]; 132; 138; 8.884219; CCATGGG; 0.57751; 0.73458;
Sequence; YY1 [T00915]; 142; 148; 6.515559; GTCATGG; 0.28876; 0.29684;
Sequence; YY1 [T00915]; 184; 190; 2.781239; GAGATGG; 0.28876; 0.28208;
Sequence; YY1 [T00915]; 222; 228; 5.043792; AGGATGG; 0.77002; 0.71592;
Sequence; YY1 [T00915]; 482; 488; 3.840427; CCATCTG; 0.38501; 0.35631;
Sequence; YY1 [T00915]; 535; 541; 7.574747; CTCATGG; 1.05878; 1.13316;
Sequence; YY1 [T00915]; 801; 807; 7.441476; CCATCAG; 1.05878; 1.13316;
Sequence; YY1 [T00915]; 874; 880; 8.486152; CCATAGA; 0.96252; 0.93991;
Sequence; YY1 [T00915]; 1361; 1367; 0.000000; AAAATGG; 0.28876; 0.19590;
Sequence; YY1 [T00915]; 1558; 1564; 5.177063; AGCATGG; 0.77002; 0.71592;
Sequence; PEA3 [T00685]; 40; 47; 9.450812; GGATGGCA; 0.57751; 0.74931;

Sequence; PEA3 [T00685]; 223; 230; 9.665283; GGATGGCC; 0.57751; 0.74931;
Sequence; PEA3 [T00685]; 609; 616; 0.214471; TGACATCC; 0.28876; 0.26941;
Sequence; PEA3 [T00685]; 698; 705; 0.214471; CCACATCC; 0.28876; 0.26941;
Sequence; ENKTF-1 [T00255]; 43; 50; 13.885529; TGGCAGCT; 0.86627; 1.11837;
Sequence; ENKTF-1 [T00255]; 66; 73; 13.885529; AGCAGCCA; 0.86627; 1.11837;
Sequence; ENKTF-1 [T00255]; 224; 231; 12.629773; GATGGCCA; 1.73254; 1.97806;
Sequence; ENKTF-1 [T00255]; 226; 233; 8.198520; TGGCCAGG; 0.57751; 0.89527;
Sequence; ENKTF-1 [T00255]; 336; 343; 6.942764; TGGCAAAG; 1.15503; 1.58403;
Sequence; ENKTF-1 [T00255]; 442; 449; 6.942764; CGAAGCCA; 1.15503; 1.58403;
Sequence; ENKTF-1 [T00255]; 539; 546; 11.374018; TGGCATAC; 0.86627; 0.85949;
Sequence; ENKTF-1 [T00255]; 577; 584; 12.629773; TGGCCTGC; 1.73254; 1.97806;
Sequence; ENKTF-1 [T00255]; 674; 681; 12.629773; TGGCCCCA; 1.73254; 1.97806;
Sequence; ENKTF-1 [T00255]; 693; 700; 12.629773; ATCTGCCA; 1.73254; 1.97806;
Sequence; ENKTF-1 [T00255]; 1182; 1189; 11.374018; GAAGGCCA; 0.86627; 0.85949;
Sequence; ENKTF-1 [T00255]; 1333; 1340; 12.629773; AGAAGCCA; 1.73254; 1.97806;
Sequence; ENKTF-1 [T00255]; 1415; 1422; 11.374018; AAATGCCA; 0.86627; 0.85949;
Sequence; ENKTF-1 [T00255]; 1562; 1569; 11.374018; TGGCTTTC; 0.86627; 0.85949;
Sequence; Myf-3 [T00519]; 45; 52; 1.122441; GCAGCTGA; 0.43314; 0.44290;
Sequence; Myf-3 [T00519]; 482; 489; 1.331099; CCATCTGA; 0.28876; 0.31569;
Sequence; Myf-3 [T00519]; 491; 498; 0.000000; GCAGCTGG; 0.43314; 0.57674;
Sequence; Myf-3 [T00519]; 528; 535; 0.000000; CCACCTGC; 0.43314; 0.57674;
Sequence; MyoD [T00525]; 45; 52; 1.122441; GCAGCTGA; 0.43314; 0.44290;
Sequence; MyoD [T00525]; 482; 489; 1.331099; CCATCTGA; 0.28876; 0.31569;
Sequence; MyoD [T00525]; 491; 498; 0.000000; GCAGCTGG; 0.43314; 0.57674;
Sequence; MyoD [T00525]; 528; 535; 0.000000; CCACCTGC; 0.43314; 0.57674;
Sequence; AP-2alphaA [T00035]; 20; 25; 2.801655; GCCTCA; 1.15503; 1.41297;
Sequence; AP-2alphaA [T00035]; 51; 56; 3.004803; GAAGGC; 1.15503; 1.41297;
Sequence; AP-2alphaA [T00035]; 78; 83; 0.159691; CCAGGC; 0.77002; 1.22585;
Sequence; AP-2alphaA [T00035]; 82; 87; 3.414894; GCCTAG; 1.15503; 1.40286;
Sequence; AP-2alphaA [T00035]; 118; 123; 3.414894; CTAGGC; 1.15503; 1.40286;
Sequence; AP-2alphaA [T00035]; 194; 199; 0.798453; GCCTGA; 0.38501; 0.46563;
Sequence; AP-2alphaA [T00035]; 229; 234; 0.159691; CCAGGC; 0.77002; 1.22585;
Sequence; AP-2alphaA [T00035]; 421; 426; 4.442019; AAAGGC; 0.77002; 0.72356;
Sequence; AP-2alphaA [T00035]; 579; 584; 0.000000; GCCTGC; 0.77002; 1.22585;
Sequence; AP-2alphaA [T00035]; 671; 676; 0.159691; GCCTGG; 0.77002; 1.22585;
Sequence; AP-2alphaA [T00035]; 849; 854; 2.162893; GCCTCG; 0.77002; 1.22044;
Sequence; AP-2alphaA [T00035]; 906; 911; 2.801655; TGAGGC; 1.15503; 1.41297;
Sequence; AP-2alphaA [T00035]; 967; 972; 3.164494; CAAGGC; 1.15503; 1.41297;
Sequence; AP-2alphaA [T00035]; 1009; 1014; 1.437215; ACAGGC; 0.38501; 0.47575;
Sequence; AP-2alphaA [T00035]; 1054; 1059; 1.437215; ACAGGC; 0.38501; 0.47575;
Sequence; AP-2alphaA [T00035]; 1140; 1145; 2.003202; GCCTCC; 0.77002; 1.22044;
Sequence; AP-2alphaA [T00035]; 1182; 1187; 3.004803; GAAGGC; 1.15503; 1.41297;
Sequence; AP-2alphaA [T00035]; 1196; 1201; 3.440418; GCCTCT; 1.15503; 1.40286;
Sequence; AP-2alphaA [T00035]; 1262; 1267; 2.162893; CGAGGC; 0.77002; 1.22044;
Sequence; AP-2alphaA [T00035]; 1292; 1297; 3.440418; AGAGGC; 1.15503; 1.40286;
Sequence; AP-2alphaA [T00035]; 1501; 1506; 3.803256; TAAGGC; 0.77002; 0.71909;
Sequence; AP-2alphaA [T00035]; 1532; 1537; 3.803256; TAAGGC; 0.77002; 0.71909;
Sequence; TCF-1A [T00999]; 68; 76; 13.648823; CAGCCAAAG; 0.21657; 0.23810;
Sequence; TCF-1A [T00999]; 335; 343; 13.648823; TTGGCAAAG; 0.21657; 0.23810;
Sequence; TCF-1A [T00999]; 663; 671; 7.421728; GCTTCAAAG; 0.27071; 0.22186;
Sequence; TCF-1A [T00999]; 730; 738; 7.421728; TCACCAAAG; 0.27071; 0.22186;
Sequence; TCF-1A [T00999]; 1131; 1139; 0.597316; CTTTGTTCG; 0.03609; 0.02533;
Sequence; TCF-1A [T00999]; 1350; 1358; 7.421728; CTTTCAAAG; 0.27071; 0.22186;

Sequence; LEF-1 [T02905]; 69; 76; 9.741481; AGCCAAAG; 0.14438; 0.14980;
Sequence; LEF-1 [T02905]; 336; 343; 9.555277; TGGCAAAG; 0.14438; 0.14980;
Sequence; LEF-1 [T02905]; 664; 671; 6.707850; CTTCAAAG; 0.09625; 0.05979;
Sequence; LEF-1 [T02905]; 731; 738; 2.021471; CACCAAAG; 0.14438; 0.12192;
Sequence; LEF-1 [T02905]; 1131; 1138; 0.372407; CTTTGTTC; 0.09625; 0.06311;
Sequence; LEF-1 [T02905]; 1351; 1358; 6.707850; TTTCAAAG; 0.09625; 0.05979;
Sequence; TCF-4E [T02878]; 70; 76; 12.604771; GCCAAAG; 0.38501; 0.38502;
Sequence; TCF-4E [T02878]; 337; 343; 6.302385; GGCAAAG; 0.48126; 0.42042;
Sequence; TCF-4E [T02878]; 665; 671; 6.302385; TTCAAAG; 0.48126; 0.42042;
Sequence; TCF-4E [T02878]; 732; 738; 6.302385; ACCAAAG; 0.48126; 0.42042;
Sequence; TCF-4E [T02878]; 1131; 1137; 6.302385; CTTTGTT; 0.48126; 0.42042;
Sequence; TCF-4E [T02878]; 1352; 1358; 6.302385; TTCAAAG; 0.48126; 0.42042;
Sequence; NFI/CTF [T00094]; 71; 78; 9.352332; CCAAAGCC; 0.43314; 0.44084;
Sequence; NFI/CTF [T00094]; 102; 109; 14.373419; CCAAAATG; 1.15503; 0.98029;
Sequence; NFI/CTF [T00094]; 331; 338; 14.601592; AGAGTTGG; 1.15503; 0.98029;
Sequence; NFI/CTF [T00094]; 370; 377; 5.558661; CCAAGCAC; 0.43314; 0.46353;
Sequence; NFI/CTF [T00094]; 596; 603; 8.814757; CCAAGAGA; 0.38501; 0.37302;
Sequence; NFI/CTF [T00094]; 656; 663; 14.373419; CCAAGTTG; 1.15503; 0.98029;
Sequence; NFI/CTF [T00094]; 690; 697; 6.786076; CCAATCTG; 0.57751; 0.65567;
Sequence; NFI/CTF [T00094]; 706; 713; 8.241664; CCAACCTG; 0.14438; 0.19154;
Sequence; NFI/CTF [T00094]; 733; 740; 9.352332; CCAAAGAC; 0.43314; 0.44084;
Sequence; NFI/CTF [T00094]; 833; 840; 5.558661; CCAAACCC; 0.43314; 0.46353;
Sequence; NFI/CTF [T00094]; 864; 871; 14.601592; CCAACTAC; 1.15503; 0.98029;
Sequence; NFI/CTF [T00094]; 966; 973; 9.352332; CCAAGGCC; 0.43314; 0.44084;
Sequence; NFI/CTF [T00094]; 1294; 1301; 5.558661; AGGCTTGG; 0.43314; 0.46353;
Sequence; NFI/CTF [T00094]; 1400; 1407; 8.241664; CCAACCCG; 0.14438; 0.19154;
Sequence; NFI/CTF [T00094]; 1420; 1427; 14.373419; CCAAAATG; 1.15503; 0.98029;
Sequence; NFI/CTF [T00094]; 1486; 1493; 0.000000; CCAAGCGT; 0.14438; 0.17573;
Sequence; p53 [T00671]; 7; 13; 6.600658; GGGCACC; 0.09625; 0.17240;
Sequence; p53 [T00671]; 73; 79; 8.906038; AAAGCCC; 0.96252; 1.56763;
Sequence; p53 [T00671]; 138; 144; 8.213500; GGGCGTC; 0.57751; 0.84971;
Sequence; p53 [T00671]; 147; 153; 7.700559; GGGCTGA; 0.38501; 0.57262;
Sequence; p53 [T00671]; 277; 283; 0.701155; GGGCAAG; 0.19250; 0.30599;
Sequence; p53 [T00671]; 408; 414; 0.525867; GGGCAGG; 0.19250; 0.30599;
Sequence; p53 [T00671]; 468; 474; 0.000000; CATGCCC; 0.09625; 0.13459;
Sequence; p53 [T00671]; 502; 508; 0.701155; CTTGCCC; 0.19250; 0.30599;
Sequence; p53 [T00671]; 631; 637; 0.525867; CCTGCCC; 0.19250; 0.30599;
Sequence; p53 [T00671]; 673; 679; 4.325788; CTGGCCC; 0.28876; 0.40491;
Sequence; p53 [T00671]; 743; 749; 8.906038; GGGCTTT; 0.96252; 1.56763;
Sequence; p53 [T00671]; 751; 757; 7.959461; TCGGCCC; 0.57751; 0.84971;
Sequence; p53 [T00671]; 767; 773; 8.739367; GCAGCCC; 0.28876; 0.57878;
Sequence; p53 [T00671]; 795; 801; 3.891597; CCCGCCC; 0.57751; 1.06420;
Sequence; p53 [T00671]; 838; 844; 3.891597; CCCGCCC; 0.57751; 1.06420;
Sequence; p53 [T00671]; 847; 853; 8.472402; GGGCCTC; 0.28876; 0.57878;
Sequence; p53 [T00671]; 889; 895; 3.891597; CCCGCCC; 0.57751; 1.06420;
Sequence; p53 [T00671]; 928; 934; 0.000000; CATGCCC; 0.09625; 0.13459;
Sequence; p53 [T00671]; 994; 1000; 3.365730; GGGCTTG; 0.28876; 0.50604;
Sequence; p53 [T00671]; 1026; 1032; 10.658927; GGGCGCT; 0.19250; 0.31354;
Sequence; p53 [T00671]; 1038; 1044; 0.525867; CCTGCCC; 0.19250; 0.30599;
Sequence; p53 [T00671]; 1067; 1073; 10.917830; AGGGCCC; 0.09625; 0.17240;
Sequence; p53 [T00671]; 1068; 1074; 10.225290; GGGCCCC; 0.38501; 0.75307;
Sequence; p53 [T00671]; 1098; 1104; 1.752889; CGTGCCC; 0.09625; 0.17164;
Sequence; p53 [T00671]; 1240; 1246; 7.700559; GGGCGGA; 0.38501; 0.57262;

Sequence; p53 [T00671]; 1263; 1269; 8.472402; GAGGCCC; 0.28876; 0.57878;
Sequence; p53 [T00671]; 1386; 1392; 8.213500; GGGCTTC; 0.57751; 0.84971;
Sequence; GATA-1 [T00306]; 90; 95; 0.552356; GATAGA; 2.31006; 1.61008;
Sequence; GATA-1 [T00306]; 125; 130; 1.908093; GGTATC; 2.31006; 1.60948;
Sequence; GATA-1 [T00306]; 164; 169; 0.060507; GATAGG; 0.77002; 0.63276;
Sequence; GATA-1 [T00306]; 551; 556; 1.391652; GATACG; 0.77002; 0.63294;
Sequence; GATA-1 [T00306]; 1049; 1054; 0.552356; GATAGA; 2.31006; 1.61008;
Sequence; GATA-1 [T00306]; 1442; 1447; 0.491849; GATAAA; 2.31006; 1.61008;
Sequence; VDR [T00885]; 98; 101; 0.000000; GGGT; 6.16016; 8.05348;
Sequence; VDR [T00885]; 112; 115; 0.000000; ACCC; 6.16016; 8.05348;
Sequence; VDR [T00885]; 237; 240; 0.000000; GGGT; 6.16016; 8.05348;
Sequence; VDR [T00885]; 247; 250; 0.000000; ACCC; 6.16016; 8.05348;
Sequence; VDR [T00885]; 368; 371; 0.000000; ACCC; 6.16016; 8.05348;
Sequence; VDR [T00885]; 404; 407; 0.000000; GGGT; 6.16016; 8.05348;
Sequence; VDR [T00885]; 510; 513; 0.000000; ACCC; 6.16016; 8.05348;
Sequence; VDR [T00885]; 629; 632; 0.000000; ACCC; 6.16016; 8.05348;
Sequence; VDR [T00885]; 820; 823; 0.000000; ACCC; 6.16016; 8.05348;
Sequence; VDR [T00885]; 837; 840; 0.000000; ACCC; 6.16016; 8.05348;
Sequence; VDR [T00885]; 872; 875; 0.000000; ACCC; 6.16016; 8.05348;
Sequence; VDR [T00885]; 887; 890; 0.000000; ACCC; 6.16016; 8.05348;
Sequence; VDR [T00885]; 959; 962; 0.000000; GGGT; 6.16016; 8.05348;
Sequence; VDR [T00885]; 974; 977; 0.000000; GGGT; 6.16016; 8.05348;
Sequence; VDR [T00885]; 1019; 1022; 0.000000; GGGT; 6.16016; 8.05348;
Sequence; VDR [T00885]; 1082; 1085; 0.000000; ACCC; 6.16016; 8.05348;
Sequence; VDR [T00885]; 1089; 1092; 0.000000; ACCC; 6.16016; 8.05348;
Sequence; VDR [T00885]; 1095; 1098; 0.000000; ACCC; 6.16016; 8.05348;
Sequence; VDR [T00885]; 1114; 1117; 0.000000; GGGT; 6.16016; 8.05348;
Sequence; VDR [T00885]; 1148; 1151; 0.000000; GGGT; 6.16016; 8.05348;
Sequence; VDR [T00885]; 1259; 1262; 0.000000; ACCC; 6.16016; 8.05348;
Sequence; VDR [T00885]; 1311; 1314; 0.000000; ACCC; 6.16016; 8.05348;
Sequence; VDR [T00885]; 1403; 1406; 0.000000; ACCC; 6.16016; 8.05348;
Sequence; VDR [T00885]; 1432; 1435; 0.000000; ACCC; 6.16016; 8.05348;
Sequence; VDR [T00885]; 1484; 1487; 0.000000; ACCC; 6.16016; 8.05348;
Sequence; VDR [T00885]; 1494; 1497; 0.000000; GGGT; 6.16016; 8.05348;
Sequence; MBF1 [T00492]; 100; 110; 9.143821; GTCCAAAATGT; 0.06091; 0.03491;
Sequence; MBF1 [T00492]; 1244; 1254; 6.505099; GGATTTTAGAG; 0.05076; 0.03144;
Sequence; MBF1 [T00492]; 1418; 1428; 7.302340; TGCCAAAATGA; 0.03722; 0.02489;
Sequence; MBF1 [T00492]; 1467; 1477; 14.636473; AACAATAATAA; 0.18273; 0.09655;
Sequence; POU3F2 [T00630]; 105; 111; 7.224857; AAATGTG; 0.57751; 0.39605;
Sequence; POU3F2 [T00630]; 302; 308; 5.457738; ATCATTT; 0.77002; 0.49236;
Sequence; POU3F2 [T00630]; 1243; 1249; 5.710420; CGGATTT; 0.77002; 0.49236;
Sequence; POU3F2 [T00630]; 1271; 1277; 5.457738; CGCATTT; 0.77002; 0.49236;
Sequence; POU3F2 [T00630]; 1323; 1329; 8.841973; GAGATTT; 0.28876; 0.15321;
Sequence; POU3F2 [T00630]; 1362; 1368; 8.108417; AAATGGG; 0.19250; 0.13611;
Sequence; POU3F2 [T00630]; 1415; 1421; 6.822173; AAATGCC; 0.57751; 0.39605;
Sequence; POU3F2 [T00630]; 1423; 1429; 5.457738; AAATGAT; 0.77002; 0.49236;
Sequence; POU3F2 [T00630]; 1509; 1515; 7.327537; GGAATTT; 0.96252; 0.48913;
Sequence; POU3F2 [T00630]; 1526; 1532; 8.361098; CCGATTT; 0.67377; 0.40614;
Sequence; FOXP3 [T04280]; 104; 112; 6.153583; AAAATGTGA; 0.27071; 0.12233;
Sequence; FOXP3 [T04280]; 1242; 1250; 13.487695; GCGGATTTT; 0.14438; 0.09298;
Sequence; FOXP3 [T04280]; 1361; 1369; 6.153583; AAAATGGGG; 0.27071; 0.12233;
Sequence; FOXP3 [T04280]; 1422; 1430; 3.076791; AAAATGATG; 0.10828; 0.04197;
Sequence; FOXP3 [T04280]; 1508; 1516; 7.334112; CGGAATTTT; 0.18047; 0.09883;

Sequence; USF2b [T02377]; 106; 113; 6.942764; AATGTGAC; 1.15503; 1.08212;
Sequence; USF2b [T02377]; 142; 149; 6.942764; GTCATGGG; 1.15503; 1.08212;
Sequence; USF2b [T02377]; 214; 221; 12.629773; GTCAGTCT; 1.73254; 1.61621;
Sequence; USF2b [T02377]; 262; 269; 1.255756; GTCACTTG; 0.19250; 0.17803;
Sequence; USF2b [T02377]; 605; 612; 12.629773; ACTCTGAC; 1.73254; 1.61621;
Sequence; USF2b [T02377]; 708; 715; 5.687009; AACCTGAC; 0.57751; 0.70456;
Sequence; USF2b [T02377]; 721; 728; 12.629773; TCCTTGAC; 1.73254; 1.61621;
Sequence; USF2b [T02377]; 761; 768; 12.629773; GTCATCGC; 1.73254; 1.61621;
Sequence; USF2b [T02377]; 807; 814; 13.885529; GCAATGAC; 0.86627; 0.62057;
Sequence; USF2b [T02377]; 1021; 1028; 5.687009; GTCAGGGG; 0.57751; 0.70456;
Sequence; USF2b [T02377]; 1284; 1291; 6.942764; CTCATGAC; 1.15503; 1.08212;
Sequence; USF2b [T02377]; 1458; 1465; 11.374018; GTCAGGAA; 0.86627; 1.05230;
Sequence; USF2 [T00878]; 106; 114; 12.285069; AATGTGACC; 0.14438; 0.15218;
Sequence; USF2 [T00878]; 261; 269; 4.200409; AGTCACTTG; 0.04813; 0.04353;
Sequence; USF2 [T00878]; 708; 716; 14.014611; AACCTGACT; 0.07219; 0.08773;
Sequence; USF2 [T00878]; 1020; 1028; 13.520501; GGTCAGGGG; 0.18047; 0.16808;
Sequence; c-Jun [T00133]; 110; 116; 6.491349; TGACCCT; 0.57751; 0.61588;
Sequence; c-Jun [T00133]; 139; 145; 4.818265; GGCGTCA; 0.38501; 0.37512;
Sequence; c-Jun [T00133]; 211; 217; 4.951897; GGTGTCA; 0.38501; 0.37512;
Sequence; c-Jun [T00133]; 259; 265; 3.818710; AGAGTCA; 0.38501; 0.29155;
Sequence; c-Jun [T00133]; 609; 615; 6.780539; TGACATC; 0.28876; 0.23770;
Sequence; c-Jun [T00133]; 712; 718; 4.622649; TGACTCG; 0.28876; 0.25611;
Sequence; c-Jun [T00133]; 725; 731; 4.642428; TGACTTC; 0.28876; 0.25611;
Sequence; c-Jun [T00133]; 758; 764; 5.823189; AGCGTCA; 0.57751; 0.51416;
Sequence; c-Jun [T00133]; 811; 817; 9.869451; TGACAAG; 0.19250; 0.15498;
Sequence; c-Jun [T00133]; 1018; 1024; 6.491349; AGGGTCA; 0.57751; 0.61588;
Sequence; c-Jun [T00133]; 1288; 1294; 5.978259; TGACAGA; 0.57751; 0.51416;
Sequence; c-Jun [T00133]; 1455; 1461; 4.501281; TAGGTCA; 0.28876; 0.25611;
Sequence; NHP-1 [T00621]; 110; 116; 7.431731; TGACCCT; 0.86627; 0.93419;
Sequence; NHP-1 [T00621]; 168; 174; 14.314808; GGACATC; 1.15503; 0.96928;
Sequence; NHP-1 [T00621]; 211; 217; 11.916538; GGTGTCA; 1.73254; 1.69881;
Sequence; NHP-1 [T00621]; 280; 286; 13.454424; CAAGTCT; 0.38501; 0.37974;
Sequence; NHP-1 [T00621]; 498; 504; 9.830000; GGACCTT; 1.44379; 1.33181;
Sequence; NHP-1 [T00621]; 609; 615; 5.345192; TGACATC; 0.57751; 0.45707;
Sequence; NHP-1 [T00621]; 627; 633; 11.916538; AGACCCT; 1.73254; 1.69881;
Sequence; NHP-1 [T00621]; 687; 693; 11.916538; AGACCAA; 1.73254; 1.69881;
Sequence; NHP-1 [T00621]; 725; 731; 9.830000; TGACTTC; 1.44379; 1.33181;
Sequence; NHP-1 [T00621]; 790; 796; 8.969616; CAGGTCC; 0.48126; 0.52503;
Sequence; NHP-1 [T00621]; 811; 817; 11.056154; TGACAAG; 0.57751; 0.65416;
Sequence; NHP-1 [T00621]; 859; 865; 8.969616; CAGGTCC; 0.48126; 0.52503;
Sequence; NHP-1 [T00621]; 1018; 1024; 7.431731; AGGGTCA; 0.86627; 0.93419;
Sequence; NHP-1 [T00621]; 1033; 1039; 8.969616; CAGGTCC; 0.48126; 0.52503;
Sequence; NHP-1 [T00621]; 1153; 1159; 14.314808; CGACATA; 1.15503; 0.96928;
Sequence; NHP-1 [T00621]; 1168; 1174; 14.314808; GGACATA; 1.15503; 0.96928;
Sequence; NHP-1 [T00621]; 1288; 1294; 11.916538; TGACAGA; 1.73254; 1.69881;
Sequence; NHP-1 [T00621]; 1341; 1347; 14.314808; GAAGTCT; 1.15503; 0.96928;
Sequence; NHP-1 [T00621]; 1455; 1461; 0.860385; TAGGTCA; 0.28876; 0.25566;
Sequence; NHP-1 [T00621]; 1523; 1529; 11.916538; AGACCGA; 1.73254; 1.69881;
Sequence; ARP-1 [T00045]; 110; 118; 6.071920; TGACCCTTC; 0.04813; 0.06240;
Sequence; ARP-1 [T00045]; 1016; 1024; 6.149212; CGAGGGTCA; 0.04813; 0.06240;
Sequence; ARP-1 [T00045]; 1453; 1461; 7.852274; AGTAGGTCA; 0.15039; 0.15134;
Sequence; RAR-gamma [T00720]; 126; 133; 3.232527; GTATCACC; 0.19250; 0.18395;
Sequence; RAR-gamma [T00720]; 170; 177; 6.005075; ACATCACC; 0.09625; 0.09416;

Sequence; RAR-gamma [T00720]; 727; 734; 5.085116; ACTTCACC; 0.19250; 0.21388;
Sequence; RAR-gamma [T00720]; 1117; 1124; 5.085116; TCCTCACC; 0.19250; 0.21388;
Sequence; RAR-gamma [T00720]; 1406; 1413; 1.839917; CGATCACC; 0.09625; 0.10905;
Sequence; c-Fos [T00123]; 109; 116; 10.552944; GTGACCCT; 0.38501; 0.32576;
Sequence; c-Fos [T00123]; 139; 146; 6.315788; GGCGTCAT; 0.24063; 0.23634;
Sequence; c-Fos [T00123]; 211; 218; 7.473109; GGTGTCAG; 0.09625; 0.10177;
Sequence; c-Fos [T00123]; 259; 266; 5.590539; AGAGTCAC; 0.07219; 0.06130;
Sequence; c-Fos [T00123]; 608; 615; 11.443018; CTGACATC; 0.33688; 0.35655;
Sequence; c-Fos [T00123]; 711; 718; 5.217470; CTGACTCG; 0.24063; 0.21492;
Sequence; c-Fos [T00123]; 724; 731; 8.913258; TTGACTTC; 0.43314; 0.33991;
Sequence; c-Fos [T00123]; 758; 765; 7.218044; AGCGTCAT; 0.09625; 0.07815;
Sequence; c-Fos [T00123]; 810; 817; 13.874665; ATGACAAG; 0.40907; 0.36512;
Sequence; c-Fos [T00123]; 1018; 1025; 9.277619; AGGGTCAG; 0.26469; 0.28944;
Sequence; c-Fos [T00123]; 1287; 1294; 8.912261; ATGACAGA; 0.43314; 0.33991;
Sequence; c-Fos [T00123]; 1455; 1462; 9.187379; TAGGTCAG; 0.43314; 0.33991;
Sequence; Pax-2 [T01823]; 107; 113; 5.131658; ATGTGAC; 0.96252; 0.79285;
Sequence; Pax-2 [T01823]; 142; 148; 0.591451; GTCATGG; 0.28876; 0.33662;
Sequence; Pax-2 [T01823]; 214; 220; 3.217275; GTCAGTC; 1.05878; 1.08872;
Sequence; Pax-2 [T01823]; 262; 268; 4.163478; GTCACTT; 0.86627; 0.88178;
Sequence; Pax-2 [T01823]; 606; 612; 4.457510; CTCTGAC; 0.38501; 0.29963;
Sequence; Pax-2 [T01823]; 709; 715; 2.862577; ACCTGAC; 1.05878; 1.08872;
Sequence; Pax-2 [T01823]; 722; 728; 1.230243; CCTTGAC; 0.38501; 0.46809;
Sequence; Pax-2 [T01823]; 761; 767; 3.495993; GTCATCG; 0.48126; 0.46055;
Sequence; Pax-2 [T01823]; 808; 814; 2.850539; CAATGAC; 1.05878; 1.08872;
Sequence; Pax-2 [T01823]; 1021; 1027; 1.230243; GTCAGGG; 0.38501; 0.46809;
Sequence; Pax-2 [T01823]; 1285; 1291; 2.223786; TCATGAC; 0.38501; 0.35963;
Sequence; Pax-2 [T01823]; 1458; 1464; 2.862577; GTCAGGA; 1.05878; 1.08872;
Sequence; Nkx2-1 [T00857]; 21; 27; 5.183213; CCTCATT; 1.34753; 1.33737;
Sequence; Nkx2-1 [T00857]; 149; 155; 5.851350; GCTGAGG; 0.57751; 0.74249;
Sequence; Nkx2-1 [T00857]; 206; 212; 5.183213; AGAGAGG; 1.34753; 1.33737;
Sequence; Nkx2-1 [T00857]; 318; 324; 3.365659; CCTCCGA; 0.57751; 0.74249;
Sequence; Nkx2-1 [T00857]; 520; 526; 5.183213; CCTCAGT; 1.34753; 1.33737;
Sequence; Nkx2-1 [T00857]; 651; 657; 5.851350; CCTCTCC; 0.57751; 0.74249;
Sequence; Nkx2-1 [T00857]; 850; 856; 6.273746; CCTCGTT; 0.57751; 0.73274;
Sequence; Nkx2-1 [T00857]; 904; 910; 3.178803; CATGAGG; 0.67377; 0.86443;
Sequence; Nkx2-1 [T00857]; 1014; 1020; 2.908086; CTCGAGG; 0.67377; 0.86443;
Sequence; Nkx2-1 [T00857]; 1118; 1124; 5.851350; CCTCACC; 0.57751; 0.74249;
Sequence; Nkx2-1 [T00857]; 1123; 1129; 5.851350; CCTCAGC; 0.57751; 0.74249;
Sequence; Nkx2-1 [T00857]; 1141; 1147; 3.365659; CCTCCGT; 0.57751; 0.74249;
Sequence; Nkx2-1 [T00857]; 1197; 1203; 5.851350; CCTCTTC; 0.57751; 0.74249;
Sequence; Nkx2-1 [T00857]; 1235; 1241; 5.851350; GCTGAGG; 0.57751; 0.74249;
Sequence; Nkx2-1 [T00857]; 1260; 1266; 4.269335; CCCGAGG; 0.48126; 0.68775;
Sequence; Nkx2-1 [T00857]; 1268; 1274; 6.273746; CCTCGCA; 0.57751; 0.73274;
Sequence; Nkx2-1 [T00857]; 1283; 1289; 3.178803; CCTCATG; 0.67377; 0.86443;
Sequence; Nkx2-1 [T00857]; 1290; 1296; 5.183213; ACAGAGG; 1.34753; 1.33737;
Sequence; NF-X3 [T01514]; 154; 161; 10.700472; GGATTCTG; 0.28876; 0.35336;
Sequence; NF-X3 [T01514]; 162; 169; 12.359921; CAGATAGG; 1.08284; 1.05338;
Sequence; NF-X3 [T01514]; 267; 274; 12.359921; TTGTTCTG; 1.08284; 1.05338;
Sequence; NF-X3 [T01514]; 456; 463; 14.267296; CAGACACC; 0.28876; 0.33286;
Sequence; NF-X3 [T01514]; 463; 470; 8.793097; CAGATCAT; 0.43314; 0.40894;
Sequence; NF-X3 [T01514]; 481; 488; 5.226274; TCCATCTG; 0.26469; 0.24430;
Sequence; NF-X3 [T01514]; 690; 697; 12.359921; CCAATCTG; 1.08284; 1.05338;
Sequence; NF-X3 [T01514]; 1188; 1195; 3.566824; CAGATCAC; 0.14438; 0.16516;

Sequence; NF-X3 [T01514]; 1291; 1298; 12.359921; CAGAGGCT; 1.08284; 1.05338;
Sequence; NF-X3 [T01514]; 1314; 1321; 14.267296; CAGAGAAC; 0.28876; 0.33286;
Sequence; NF-X3 [T01514]; 1339; 1346; 4.935373; CAGAAGTC; 0.26469; 0.24430;
Sequence; GATA-3 [T00311]; 163; 170; 1.766593; AGATAGGA; 0.33688; 0.20324;
Sequence; GATA-3 [T00311]; 550; 557; 3.975111; AGATACGT; 0.02406; 0.01665;
Sequence; TGIF [T04076]; 210; 217; 1.303150; AGGTGTCA; 0.14438; 0.13398;
Sequence; TGIF [T04076]; 609; 616; 2.318943; TGACATCC; 0.36095; 0.28661;
Sequence; TGIF [T04076]; 811; 818; 2.490395; TGACAAGA; 0.36095; 0.28661;
Sequence; TGIF [T04076]; 1288; 1295; 2.169992; TGACAGAG; 0.31282; 0.27629;
Sequence; PKNOX1 [T04122]; 185; 195; 14.455439; AGATGGACAGC; 0.12182; 0.11413;
Sequence; PKNOX1 [T04122]; 211; 221; 14.455439; GGTGTCAGTCT; 0.12182; 0.11413;
Sequence; PKNOX1 [T04122]; 1284; 1294; 8.818179; CTCATGACAGA; 0.01805; 0.01785;
Sequence; FOXO3a [T02938]; 251; 258; 6.942764; CTTACTCA; 1.15503; 0.75166;
Sequence; FOXO3a [T02938]; 395; 402; 8.198520; AAGGTAAG; 0.57751; 0.48940;
Sequence; FOXO3a [T02938]; 621; 628; 6.942764; TGCTTAAG; 1.15503; 0.75166;
Sequence; FOXO3a [T02938]; 623; 630; 12.629773; CTTAAGAC; 1.73254; 1.34949;
Sequence; FOXO3a [T02938]; 896; 903; 12.629773; ACCTTAAG; 1.73254; 1.34949;
Sequence; FOXO3a [T02938]; 898; 905; 6.942764; CTTAAGCA; 1.15503; 0.75166;
Sequence; FOXO3a [T02938]; 1308; 1315; 8.198520; CTTACCCA; 0.57751; 0.48940;
Sequence; FOXO3a [T02938]; 1471; 1478; 11.374018; ATAATAAG; 0.86627; 0.51816;
Sequence; FOXO3a [T02938]; 1497; 1504; 6.942764; TTTCTAAG; 1.15503; 0.75166;
Sequence; FOXO3a [T02938]; 1528; 1535; 5.687009; GATTTAAG; 0.57751; 0.28861;
Sequence; Hlf [T01071]; 251; 259; 6.800232; CTTACTCAA; 0.10828; 0.07112;
Sequence; Hlf [T01071]; 1250; 1258; 8.501296; TAGAGTAAC; 0.13836; 0.09026;
Sequence; AP-1 [T00029]; 258; 265; 7.039020; AAGAGTCA; 0.09625; 0.07773;
Sequence; AP-1 [T00029]; 712; 719; 7.617752; TGACTCGA; 0.09625; 0.10113;
Sequence; AP-1 [T00029]; 725; 732; 11.843817; TGACTTCA; 0.28876; 0.23818;
Sequence; PR B [T00696]; 266; 272; 5.756916; CTTGTTC; 0.57751; 0.45498;
Sequence; PR B [T00696]; 428; 434; 4.245948; CAACAAT; 0.48126; 0.28331;
Sequence; PR B [T00696]; 1132; 1138; 5.756916; TTTGTTC; 0.57751; 0.45498;
Sequence; PR B [T00696]; 1159; 1165; 5.661264; ATTGTTA; 0.19250; 0.09232;
Sequence; PR B [T00696]; 1255; 1261; 9.295206; TAACACC; 0.28876; 0.22977;
Sequence; PR B [T00696]; 1445; 1451; 7.172232; AAACAGG; 0.57751; 0.53866;
Sequence; PR B [T00696]; 1466; 1472; 1.415316; GAACAAT; 0.19250; 0.12122;
Sequence; PR A [T01661]; 266; 272; 5.756916; CTTGTTC; 0.57751; 0.45498;
Sequence; PR A [T01661]; 428; 434; 4.245948; CAACAAT; 0.48126; 0.28331;
Sequence; PR A [T01661]; 1132; 1138; 5.756916; TTTGTTC; 0.57751; 0.45498;
Sequence; PR A [T01661]; 1159; 1165; 5.661264; ATTGTTA; 0.19250; 0.09232;
Sequence; PR A [T01661]; 1255; 1261; 9.295206; TAACACC; 0.28876; 0.22977;
Sequence; PR A [T01661]; 1445; 1451; 7.172232; AAACAGG; 0.57751; 0.53866;
Sequence; PR A [T01661]; 1466; 1472; 1.415316; GAACAAT; 0.19250; 0.12122;
Sequence; GR-alpha [T00337]; 267; 274; 1.373275; TTGTTCTG; 0.09625; 0.06511;
Sequence; GR-alpha [T00337]; 426; 433; 9.197807; CACAACAA; 0.28876; 0.20462;
Sequence; GR-alpha [T00337]; 1133; 1140; 6.618993; TTGTTCGG; 0.24063; 0.22143;
Sequence; GR-alpha [T00337]; 1443; 1450; 12.401641; ATAAACAG; 0.14438; 0.10929;
Sequence; GR-alpha [T00337]; 1464; 1471; 1.578952; AAGAACAA; 0.09625; 0.06511;
Sequence; GR-beta [T01920]; 268; 275; 0.282141; TGTTCTGT; 0.09625; 0.07714;
Sequence; GR-beta [T01920]; 425; 432; 10.375916; GCACAACA; 0.14438; 0.13742;
Sequence; GR-beta [T01920]; 1134; 1141; 12.081725; TGTTCGGC; 0.38501; 0.35499;
Sequence; GR-beta [T01920]; 1463; 1470; 0.726681; GAAGAACA; 0.09625; 0.07714;
Sequence; GR [T05076]; 264; 276; 9.294378; CACTTGTTCTGTA; 0.00837; 0.00621;
Sequence; LCR-F1 [T01599]; 21; 26; 5.143424; CCTCAT; 1.15503; 0.90891;
Sequence; LCR-F1 [T01599]; 141; 146; 0.029165; CGTCAT; 1.54004; 1.27130;

Sequence; LCR-F1 [T01599]; 290; 295; 5.375891; ATGAAG; 1.92505; 1.33393;
Sequence; LCR-F1 [T01599]; 298; 303; 5.114259; ACTCAT; 1.15503; 0.90891;
Sequence; LCR-F1 [T01599]; 301; 306; 3.748626; CATCAT; 1.15503; 0.69797;
Sequence; LCR-F1 [T01599]; 347; 352; 5.375891; ATGAAG; 1.92505; 1.33393;
Sequence; LCR-F1 [T01599]; 433; 438; 5.114259; ATGAGT; 1.15503; 0.90891;
Sequence; LCR-F1 [T01599]; 465; 470; 3.923618; GATCAT; 0.38501; 0.27314;
Sequence; LCR-F1 [T01599]; 534; 539; 5.318416; GCTCAT; 1.92505; 1.33393;
Sequence; LCR-F1 [T01599]; 760; 765; 0.029165; CGTCAT; 1.54004; 1.27130;
Sequence; LCR-F1 [T01599]; 810; 815; 0.000000; ATGACA; 1.54004; 1.27130;
Sequence; LCR-F1 [T01599]; 826; 831; 5.375891; CTTCAT; 1.92505; 1.33393;
Sequence; LCR-F1 [T01599]; 905; 910; 5.143424; ATGAGG; 1.15503; 0.90891;
Sequence; LCR-F1 [T01599]; 925; 930; 3.923618; GATCAT; 0.38501; 0.27314;
Sequence; LCR-F1 [T01599]; 940; 945; 5.550882; GTTCAT; 1.92505; 1.33393;
Sequence; LCR-F1 [T01599]; 1283; 1288; 5.143424; CCTCAT; 1.15503; 0.90891;
Sequence; LCR-F1 [T01599]; 1287; 1292; 0.000000; ATGACA; 1.54004; 1.27130;
Sequence; LCR-F1 [T01599]; 1425; 1430; 3.748626; ATGATG; 1.15503; 0.69797;
Sequence; LCR-F1 [T01599]; 1428; 1433; 5.550882; ATGAAC; 1.92505; 1.33393;
Sequence; Oct-B1 [T00545]; 305; 312; 12.500000; ATTTGCTT; 0.57751; 0.34951;
Sequence; POU2F2 (Oct-2.1) [T00646]; 305; 312; 5.569054; ATTTGCTT; 0.07219; 0.04101;
Sequence; POU2F2 (Oct-2.1) [T00646]; 1274; 1281; 10.519488; ATTTCCGT; 0.19250; 0.10641;
Sequence; POU2F2 (Oct-2.1) [T00646]; 1358; 1365; 14.733084; GGGAAAAT; 0.33688; 0.21131;
Sequence; POU2F2 (Oct-2.1) [T00646]; 1411; 1418; 13.211194; ACCGAAAT; 0.12032; 0.07523;
Sequence; POU2F2B [T00662]; 305; 312; 12.500000; ATTTGCTT; 0.57751; 0.34951;
Sequence; C/EBPalpha [T00105]; 277; 282; 0.858813; GGGCAA; 1.54004; 1.60484;
Sequence; C/EBPalpha [T00105]; 307; 312; 0.644110; TTGCTT; 0.77002; 0.65392;
Sequence; C/EBPalpha [T00105]; 336; 341; 1.717627; TGGCAA; 2.31006; 2.02163;
Sequence; C/EBPalpha [T00105]; 503; 508; 0.858813; TTGCCC; 1.54004; 1.60484;
Sequence; C/EBPalpha [T00105]; 661; 666; 0.644110; TTGCTT; 0.77002; 0.65392;
Sequence; C/EBPalpha [T00105]; 805; 810; 1.073517; CAGCAA; 1.54004; 1.60484;
Sequence; C/EBPalpha [T00105]; 1229; 1234; 0.644110; GTGCAA; 0.77002; 0.65392;
Sequence; C/EBPalpha [T00105]; 1537; 1542; 1.717627; CTGCAA; 2.31006; 2.02163;
Sequence; C/EBPbeta [T00581]; 276; 282; 2.849631; AGGGCAA; 0.77002; 0.77272;
Sequence; C/EBPbeta [T00581]; 307; 313; 4.422621; TTGCTTC; 0.67377; 0.57037;
Sequence; C/EBPbeta [T00581]; 335; 341; 4.001351; TTGGCAA; 0.48126; 0.50084;
Sequence; C/EBPbeta [T00581]; 503; 509; 2.849631; TTGCCCC; 0.77002; 0.77272;
Sequence; C/EBPbeta [T00581]; 661; 667; 4.422621; TTGCTTC; 0.67377; 0.57037;
Sequence; C/EBPbeta [T00581]; 804; 810; 4.399718; TCAGCAA; 0.67377; 0.57037;
Sequence; C/EBPbeta [T00581]; 1228; 1234; 1.880632; CGTGCAA; 0.38501; 0.36226;
Sequence; C/EBPbeta [T00581]; 1536; 1542; 4.329855; GCTGCAA; 0.67377; 0.57037;
Sequence; FOXN2 [T04206]; 313; 323; 3.686082; CGTGTCCTCCG; 0.00677; 0.00751;
Sequence; FOXN2 [T04206]; 1278; 1288; 11.620398; CCGTTCCTCAT; 0.02030; 0.02235;
Sequence; NF-1 [T00539]; 70; 74; 0.000000; GCCAA; 1.54004; 1.71710;
Sequence; NF-1 [T00539]; 335; 339; 0.000000; TTGGC; 1.54004; 1.71710;
Sequence; NF-1 [T00539]; 1419; 1423; 0.000000; GCCAA; 1.54004; 1.71710;
Sequence; IRF-1 [T00423]; 388; 396; 4.389325; CTAGGGAAA; 0.09024; 0.07814;
Sequence; IRF-1 [T00423]; 452; 460; 7.503955; TTTCCAGAC; 0.30680; 0.28813;
Sequence; IRF-1 [T00423]; 640; 648; 8.452712; TTTCCACAA; 0.09024; 0.08814;
Sequence; IRF-1 [T00423]; 1275; 1283; 2.808808; TTTCCGTTC; 0.09625; 0.05966;
Sequence; IRF-1 [T00423]; 1355; 1363; 0.000000; AAAGGGAAA; 0.00602; 0.00389;
Sequence; IRF-1 [T00423]; 1364; 1372; 5.520843; ATGGGGAAA; 0.26469; 0.19993;
Sequence; IRF-1 [T00423]; 1514; 1522; 5.288112; TTTCCGTAC; 0.26469; 0.19993;
Sequence; IRF-1 [T00423]; 1566; 1574; 4.662185; TTTCCCTGA; 0.09024; 0.07814;
Sequence; IRF-3 [T04673]; 392; 398; 0.715278; GGAAAGG; 0.38501; 0.34750;

Sequence; IRF-3 [T04673]; 450; 456; 0.715278; CCTTTCC; 0.38501; 0.34750;
Sequence; IRF-3 [T04673]; 638; 644; 1.924527; TTTTTCC; 0.19250; 0.14471;
Sequence; IRF-3 [T04673]; 1273; 1279; 1.703218; CATTTCC; 0.28876; 0.25747;
Sequence; IRF-3 [T04673]; 1359; 1365; 0.732396; GGAAAAT; 0.38501; 0.34750;
Sequence; IRF-3 [T04673]; 1368; 1374; 1.209248; GGAAAAG; 0.38501; 0.29300;
Sequence; IRF-3 [T04673]; 1512; 1518; 0.732396; ATTTTCC; 0.38501; 0.34750;
Sequence; IRF-3 [T04673]; 1564; 1570; 0.000000; GCTTTCC; 0.19250; 0.17898;
Sequence; NF-AT1 [T00550]; 392; 400; 8.019368; GGAAAGGTA; 0.13836; 0.12013;
Sequence; NF-AT1 [T00550]; 448; 456; 8.019368; CACCTTTCC; 0.13836; 0.12013;
Sequence; NF-AT1 [T00550]; 636; 644; 6.325362; CCTTTTTCC; 0.07821; 0.06231;
Sequence; NF-AT1 [T00550]; 1271; 1279; 8.704758; CGCATTTCC; 0.21657; 0.17186;
Sequence; NF-AT1 [T00550]; 1359; 1367; 6.924484; GGAAAATGG; 0.09024; 0.07284;
Sequence; NF-AT1 [T00550]; 1368; 1376; 6.201000; GGAAAAGAA; 0.07219; 0.04657;
Sequence; NF-AT1 [T00550]; 1510; 1518; 6.676564; GAATTTTCC; 0.07821; 0.06231;
Sequence; NF-AT1 [T00550]; 1562; 1570; 8.449873; TGGCTTTCC; 0.21657; 0.17186;
Sequence; WT1 I [T01840]; 242; 248; 1.994344; CCCCCAC; 0.28876; 0.45038;
Sequence; WT1 I [T01840]; 283; 289; 9.125186; GTCTCAC; 0.38501; 0.48862;
Sequence; WT1 I [T01840]; 373; 379; 6.133670; AGCACAC; 0.67377; 0.91003;
Sequence; WT1 I [T01840]; 402; 408; 0.997172; GTGGGTG; 0.19250; 0.30787;
Sequence; WT1 I [T01840]; 406; 412; 8.510618; GTGGGCA; 0.38501; 0.41979;
Sequence; WT1 I [T01840]; 470; 476; 8.510618; TGCCCAC; 0.38501; 0.41979;
Sequence; WT1 I [T01840]; 505; 511; 6.133670; GCCCCAC; 0.67377; 0.91003;
Sequence; WT1 I [T01840]; 525; 531; 8.128015; GTCCCAC; 0.57751; 0.79947;
Sequence; WT1 I [T01840]; 613; 619; 8.128015; ATCCCAC; 0.57751; 0.79947;
Sequence; WT1 I [T01840]; 676; 682; 6.133670; GCCCCAC; 0.67377; 0.91003;
Sequence; WT1 I [T01840]; 770; 776; 6.133670; GCCCCAC; 0.67377; 0.91003;
Sequence; WT1 I [T01840]; 891; 897; 2.991516; CGCCCAC; 0.38501; 0.57830;
Sequence; WT1 I [T01840]; 910; 916; 8.128015; GCCGCAC; 0.57751; 0.79947;
Sequence; WT1 I [T01840]; 1077; 1083; 6.516274; TCCACAC; 0.19250; 0.21752;
Sequence; WT1 I [T01840]; 1081; 1087; 0.997172; CACCCAC; 0.19250; 0.30787;
Sequence; WT1 I [T01840]; 1100; 1106; 8.510618; TGCCCAC; 0.38501; 0.41979;
Sequence; WT1 I [T01840]; 1117; 1123; 8.510618; TCCTCAC; 0.38501; 0.41979;
Sequence; WT1 I [T01840]; 1146; 1152; 5.136498; GTGGGTC; 0.57751; 0.80635;
Sequence; WT1 I [T01840]; 1492; 1498; 5.136498; GTGGGTT; 0.57751; 0.80635;
Sequence; Pax-6 [T01122]; 412; 422; 13.409585; AGGTTAGTGAA; 0.09024; 0.07171;
Sequence; Pax-6 [T01122]; 729; 739; 9.073283; TTCACCAAAGA; 0.05001; 0.04068;
Sequence; SRY [T00997]; 266; 272; 2.154058; CTTGTTC; 0.57751; 0.43517;
Sequence; SRY [T00997]; 425; 431; 6.542222; GCACAAC; 0.77002; 0.87258;
Sequence; SRY [T00997]; 428; 434; 0.544384; CAACAAT; 0.38501; 0.26711;
Sequence; SRY [T00997]; 643; 649; 6.723683; CCACAAC; 0.77002; 0.87258;
Sequence; SRY [T00997]; 811; 817; 6.224664; TGACAAG; 0.96252; 0.83449;
Sequence; SRY [T00997]; 997; 1003; 5.698344; CTTGTAC; 0.57751; 0.40533;
Sequence; SRY [T00997]; 1084; 1090; 6.723683; CCACAAC; 0.77002; 0.87258;
Sequence; SRY [T00997]; 1132; 1138; 0.474868; TTTGTTC; 0.38501; 0.26711;
Sequence; SRY [T00997]; 1159; 1165; 0.045365; ATTGTTA; 0.38501; 0.20512;
Sequence; SRY [T00997]; 1466; 1472; 0.362923; GAACAAT; 0.38501; 0.26711;
Sequence; NF-AT2 [T01945]; 392; 400; 6.814008; GGAAAGGTA; 0.10227; 0.09439;
Sequence; NF-AT2 [T01945]; 448; 456; 8.895812; CACCTTTCC; 0.08422; 0.09572;
Sequence; NF-AT2 [T01945]; 636; 644; 6.276636; CCTTTTTCC; 0.15641; 0.11987;
Sequence; NF-AT2 [T01945]; 1271; 1279; 9.360839; CGCATTTCC; 0.04813; 0.05461;
Sequence; NF-AT2 [T01945]; 1359; 1367; 6.842892; GGAAAATGG; 0.10227; 0.09439;
Sequence; NF-AT2 [T01945]; 1368; 1376; 6.603507; GGAAAAGAA; 0.15641; 0.11987;
Sequence; NF-AT2 [T01945]; 1510; 1518; 4.729864; GAATTTTCC; 0.09024; 0.06078;

Sequence; NF-AT2 [T01945]; 1562; 1570; 7.092964; TGGCTTTCC; 0.10227; 0.09439;
Sequence; NF-AT1 [T01948]; 392; 399; 3.206881; GGAAAGGT; 0.24063; 0.21573;
Sequence; NF-AT1 [T01948]; 449; 456; 3.206881; ACCTTTCC; 0.24063; 0.21573;
Sequence; NF-AT1 [T01948]; 637; 644; 0.641376; CTTTTTCC; 0.04813; 0.03409;
Sequence; NF-AT1 [T01948]; 1272; 1279; 4.275841; GCATTTCC; 0.14438; 0.14053;
Sequence; NF-AT1 [T01948]; 1359; 1366; 1.496544; GGAAAATG; 0.26469; 0.19869;
Sequence; NF-AT1 [T01948]; 1368; 1375; 3.634465; GGAAAAGA; 0.21657; 0.19993;
Sequence; NF-AT1 [T01948]; 1511; 1518; 0.855168; AATTTTCC; 0.12032; 0.07519;
Sequence; NF-AT1 [T01948]; 1563; 1570; 3.206881; GGCTTTCC; 0.24063; 0.21573;
Sequence; HMG I(Y) [T02368]; 31; 37; 2.475749; GGAAGTC; 0.38501; 0.36321;
Sequence; HMG I(Y) [T02368]; 392; 398; 5.398478; GGAAAGG; 0.38501; 0.40999;
Sequence; HMG I(Y) [T02368]; 450; 456; 5.398478; CCTTTCC; 0.38501; 0.40999;
Sequence; HMG I(Y) [T02368]; 477; 483; 10.297523; TGATTCC; 0.19250; 0.15477;
Sequence; HMG I(Y) [T02368]; 514; 520; 4.899046; GGAATTC; 0.48126; 0.39014;
Sequence; HMG I(Y) [T02368]; 515; 521; 4.899046; GAATTCC; 0.48126; 0.39014;
Sequence; HMG I(Y) [T02368]; 638; 644; 3.535739; TTTTTCC; 0.28876; 0.21722;
Sequence; HMG I(Y) [T02368]; 828; 834; 10.297523; TCATTCC; 0.19250; 0.15477;
Sequence; HMG I(Y) [T02368]; 1059; 1065; 7.014009; CGCTTCC; 0.48126; 0.60984;
Sequence; HMG I(Y) [T02368]; 1198; 1204; 4.291053; CTCTTCC; 0.38501; 0.35731;
Sequence; HMG I(Y) [T02368]; 1211; 1217; 9.437306; GGAATCG; 0.57751; 0.61240;
Sequence; HMG I(Y) [T02368]; 1219; 1225; 6.153792; AGCTTCC; 0.57751; 0.52871;
Sequence; HMG I(Y) [T02368]; 1273; 1279; 0.860218; CATTTCC; 0.19250; 0.15477;
Sequence; HMG I(Y) [T02368]; 1278; 1284; 8.629540; CCGTTCC; 0.57751; 0.68914;
Sequence; HMG I(Y) [T02368]; 1344; 1350; 4.291053; GTCTTCC; 0.38501; 0.35731;
Sequence; HMG I(Y) [T02368]; 1359; 1365; 1.815304; GGAAAAT; 0.09625; 0.05980;
Sequence; HMG I(Y) [T02368]; 1368; 1374; 2.675522; GGAAAAG; 0.38501; 0.36321;
Sequence; HMG I(Y) [T02368]; 1395; 1401; 4.951498; TAGTTCC; 0.48126; 0.39014;
Sequence; HMG I(Y) [T02368]; 1436; 1442; 2.475749; GGAAGTG; 0.38501; 0.36321;
Sequence; HMG I(Y) [T02368]; 1450; 1456; 3.335966; GGAAGTA; 0.28876; 0.21722;
Sequence; HMG I(Y) [T02368]; 1462; 1468; 5.151271; GGAAGAA; 0.48126; 0.39014;
Sequence; HMG I(Y) [T02368]; 1509; 1515; 4.038828; GGAATTT; 0.28876; 0.26270;
Sequence; HMG I(Y) [T02368]; 1512; 1518; 1.815304; ATTTTCC; 0.09625; 0.05980;
Sequence; HMG I(Y) [T02368]; 1543; 1549; 7.874227; GGAAGGA; 0.38501; 0.40434;
Sequence; HMG I(Y) [T02368]; 1564; 1570; 5.398478; GCTTTCC; 0.38501; 0.40999;
Sequence; STAT4 [T01577]; 31; 36; 2.941176; GGAAGT; 2.31006; 2.19013;
Sequence; STAT4 [T01577]; 392; 397; 2.941176; GGAAAG; 2.31006; 2.19013;
Sequence; STAT4 [T01577]; 451; 456; 2.941176; CTTTCC; 2.31006; 2.19013;
Sequence; STAT4 [T01577]; 478; 483; 2.941176; GATTCC; 2.31006; 2.19013;
Sequence; STAT4 [T01577]; 514; 519; 1.470588; GGAATT; 1.54004; 1.28270;
Sequence; STAT4 [T01577]; 516; 521; 1.470588; AATTCC; 1.54004; 1.28270;
Sequence; STAT4 [T01577]; 639; 644; 1.470588; TTTTCC; 1.54004; 1.28270;
Sequence; STAT4 [T01577]; 829; 834; 4.411765; CATTCC; 1.54004; 1.65012;
Sequence; STAT4 [T01577]; 1060; 1065; 4.411765; GCTTCC; 1.54004; 1.65012;
Sequence; STAT4 [T01577]; 1199; 1204; 4.411765; TCTTCC; 1.54004; 1.65012;
Sequence; STAT4 [T01577]; 1211; 1216; 2.941176; GGAATC; 2.31006; 2.19013;
Sequence; STAT4 [T01577]; 1220; 1225; 4.411765; GCTTCC; 1.54004; 1.65012;
Sequence; STAT4 [T01577]; 1274; 1279; 0.000000; ATTTCC; 0.38501; 0.27363;
Sequence; STAT4 [T01577]; 1279; 1284; 4.411765; CGTTCC; 1.54004; 1.65012;
Sequence; STAT4 [T01577]; 1345; 1350; 4.411765; TCTTCC; 1.54004; 1.65012;
Sequence; STAT4 [T01577]; 1359; 1364; 1.470588; GGAAAA; 1.54004; 1.28270;
Sequence; STAT4 [T01577]; 1368; 1373; 1.470588; GGAAAA; 1.54004; 1.28270;
Sequence; STAT4 [T01577]; 1396; 1401; 1.470588; AGTTCC; 1.54004; 1.28270;
Sequence; STAT4 [T01577]; 1436; 1441; 2.941176; GGAAGT; 2.31006; 2.19013;

Sequence; STAT4 [T01577]; 1450; 1455; 2.941176; GGAAGT; 2.31006; 2.19013;
Sequence; STAT4 [T01577]; 1462; 1467; 4.411765; GGAAGA; 1.54004; 1.65012;
Sequence; STAT4 [T01577]; 1509; 1514; 1.470588; GGAATT; 1.54004; 1.28270;
Sequence; STAT4 [T01577]; 1513; 1518; 1.470588; TTTTCC; 1.54004; 1.28270;
Sequence; STAT4 [T01577]; 1543; 1548; 5.882353; GGAAGG; 0.38501; 0.46746;
Sequence; STAT4 [T01577]; 1565; 1570; 2.941176; CTTTCC; 2.31006; 2.19013;
Sequence; c-Ets-1 [T00112]; 29; 35; 6.471844; AGGGAAG; 0.38501; 0.41384;
Sequence; c-Ets-1 [T00112]; 390; 396; 8.462584; AGGGAAA; 0.57751; 0.55907;
Sequence; c-Ets-1 [T00112]; 452; 458; 7.229570; TTTCCAG; 0.09625; 0.07773;
Sequence; c-Ets-1 [T00112]; 479; 485; 10.368500; ATTCCAT; 0.57751; 0.55459;
Sequence; c-Ets-1 [T00112]; 512; 518; 8.387082; CCGGAAT; 0.57751; 0.55907;
Sequence; c-Ets-1 [T00112]; 517; 523; 4.968134; ATTCCTC; 0.28876; 0.23752;
Sequence; c-Ets-1 [T00112]; 640; 646; 7.552643; TTTCCAC; 0.28876; 0.27343;
Sequence; c-Ets-1 [T00112]; 830; 836; 10.530037; ATTCCAA; 0.57751; 0.55459;
Sequence; c-Ets-1 [T00112]; 1061; 1067; 6.633380; CTTCCCA; 0.38501; 0.41384;
Sequence; c-Ets-1 [T00112]; 1200; 1206; 4.065094; CTTCCGC; 0.28876; 0.33880;
Sequence; c-Ets-1 [T00112]; 1209; 1215; 4.645061; CAGGAAT; 0.48126; 0.43966;
Sequence; c-Ets-1 [T00112]; 1221; 1227; 3.742021; CTTCCGG; 0.09625; 0.13251;
Sequence; c-Ets-1 [T00112]; 1275; 1281; 6.217371; TTTCCGT; 0.38501; 0.39381;
Sequence; c-Ets-1 [T00112]; 1280; 1286; 4.304554; GTTCCTC; 0.48126; 0.43966;
Sequence; c-Ets-1 [T00112]; 1346; 1352; 0.484609; CTTCCTT; 0.28876; 0.25613;
Sequence; c-Ets-1 [T00112]; 1357; 1363; 8.462584; AGGGAAA; 0.57751; 0.55907;
Sequence; c-Ets-1 [T00112]; 1366; 1372; 8.301047; GGGGAAA; 0.57751; 0.55907;
Sequence; c-Ets-1 [T00112]; 1397; 1403; 9.866456; GTTCCAA; 0.38501; 0.36595;
Sequence; c-Ets-1 [T00112]; 1434; 1440; 3.742021; CCGGAAG; 0.09625; 0.13251;
Sequence; c-Ets-1 [T00112]; 1448; 1454; 0.000000; CAGGAAG; 0.09625; 0.10194;
Sequence; c-Ets-1 [T00112]; 1460; 1466; 0.000000; CAGGAAG; 0.09625; 0.10194;
Sequence; c-Ets-1 [T00112]; 1507; 1513; 8.710155; GCGGAAT; 0.57751; 0.55907;
Sequence; c-Ets-1 [T00112]; 1514; 1520; 6.217371; TTTCCGT; 0.38501; 0.39381;
Sequence; c-Ets-1 [T00112]; 1541; 1547; 0.484609; AAGGAAG; 0.28876; 0.25613;
Sequence; c-Ets-1 [T00112]; 1566; 1572; 8.462584; TTTCCCT; 0.57751; 0.55907;
Sequence; STAT1beta [T01573]; 451; 460; 14.492168; CTTTCCAGAC; 0.16243; 0.14056;
Sequence; STAT1beta [T01573]; 639; 648; 11.593735; TTTTCCACAA; 0.24364; 0.20226;
Sequence; STAT1beta [T01573]; 1274; 1283; 13.817926; ATTTCCGTTC; 0.02707; 0.02321;
Sequence; STAT1beta [T01573]; 1364; 1373; 14.492168; ATGGGGAAAA; 0.16243; 0.14056;
Sequence; STAT1beta [T01573]; 1565; 1574; 4.010530; CTTTCCCTGA; 0.02707; 0.02100;
Sequence; Smad3 [T04096]; 452; 461; 4.018497; TTTCCAGACA; 0.06918; 0.06074;
Sequence; Smad3 [T04096]; 573; 582; 2.567648; TGTCTGGCCT; 0.03459; 0.03835;
Sequence; ANF [T00025]; 451; 462; 13.199529; CTTTCCAGACAC; 0.12690; 0.10457;
Sequence; ANF [T00025]; 1309; 1320; 9.771221; TTACCCAGAGAA; 0.02538; 0.01695;
Sequence; Smad4 [T04292]; 452; 462; 12.563504; TTTCCAGACAC; 0.01805; 0.02042;
Sequence; Smad4 [T04292]; 572; 582; 4.775963; CTGTCTGGCCT; 0.01354; 0.01464;
Sequence; EIIaE-A [T00246]; 5; 11; 13.063953; GGGGGCA; 2.50256; 3.59757;
Sequence; EIIaE-A [T00246]; 28; 34; 10.118218; CAGGGAA; 1.73254; 2.62279;
Sequence; EIIaE-A [T00246]; 136; 142; 8.709302; GGGGGCG; 1.73254; 2.62019;
Sequence; EIIaE-A [T00246]; 275; 281; 5.763567; TAGGGCA; 0.57751; 0.86843;
Sequence; EIIaE-A [T00246]; 389; 395; 10.118218; TAGGGAA; 1.73254; 2.62279;
Sequence; EIIaE-A [T00246]; 406; 412; 13.063953; GTGGGCA; 2.50256; 3.59757;
Sequence; EIIaE-A [T00246]; 470; 476; 13.063953; TGCCCAC; 2.50256; 3.59757;
Sequence; EIIaE-A [T00246]; 504; 510; 14.472869; TGCCCCA; 2.50256; 3.60095;
Sequence; EIIaE-A [T00246]; 509; 515; 14.472869; CACCCGG; 2.50256; 3.60095;
Sequence; EIIaE-A [T00246]; 633; 639; 4.354651; TGCCCTT; 0.57751; 0.86801;
Sequence; EIIaE-A [T00246]; 776; 782; 10.118218; CTCCCGG; 1.73254; 2.62279;

Sequence; EIIaE-A [T00246]; 797; 803; 10.118218; CGCCCCA; 1.73254; 2.62279;
Sequence; EIIaE-A [T00246]; 840; 846; 8.709302; CGCCCCC; 1.73254; 2.62019;
Sequence; EIIaE-A [T00246]; 842; 848; 14.472869; CCCCCGG; 2.50256; 3.60095;
Sequence; EIIaE-A [T00246]; 845; 851; 14.472869; CCGGGCC; 2.50256; 3.60095;
Sequence; EIIaE-A [T00246]; 891; 897; 8.709302; CGCCCAC; 1.73254; 2.62019;
Sequence; EIIaE-A [T00246]; 930; 936; 14.472869; TGCCCAG; 2.50256; 3.60095;
Sequence; EIIaE-A [T00246]; 990; 996; 13.063953; GCGGGGG; 2.50256; 3.59757;
Sequence; EIIaE-A [T00246]; 1024; 1030; 8.709302; AGGGGCG; 1.73254; 2.62019;
Sequence; EIIaE-A [T00246]; 1040; 1046; 13.063953; TGCCCCC; 2.50256; 3.59757;
Sequence; EIIaE-A [T00246]; 1042; 1048; 14.472869; CCCCCGG; 2.50256; 3.60095;
Sequence; EIIaE-A [T00246]; 1066; 1072; 10.118218; CAGGGCC; 1.73254; 2.62279;
Sequence; EIIaE-A [T00246]; 1100; 1106; 13.063953; TGCCCAC; 2.50256; 3.59757;
Sequence; EIIaE-A [T00246]; 1238; 1244; 0.000000; GAGGGCG; 0.19250; 0.30599;
Sequence; EIIaE-A [T00246]; 1258; 1264; 14.472869; CACCCGA; 2.50256; 3.60095;
Sequence; EIIaE-A [T00246]; 1265; 1271; 8.709302; GGCCCTC; 1.73254; 2.62019;
Sequence; EIIaE-A [T00246]; 1303; 1309; 13.063953; CTCCCCT; 2.50256; 3.59757;
Sequence; EIIaE-A [T00246]; 1304; 1310; 13.063953; TCCCCTT; 2.50256; 3.59757;
Sequence; EIIaE-A [T00246]; 1356; 1362; 8.709302; AAGGGAA; 1.73254; 2.62019;
Sequence; EIIaE-A [T00246]; 1384; 1390; 13.063953; GCGGGCT; 2.50256; 3.59757;
Sequence; EIIaE-A [T00246]; 1553; 1559; 14.472869; CTGGGAG; 2.50256; 3.60095;
Sequence; EIIaE-A [T00246]; 1567; 1573; 10.118218; TTCCCTG; 1.73254; 2.62279;
Sequence; LF-A1 [T00467]; 5; 11; 1.561557; GGGGGCA; 0.86627; 1.21548;
Sequence; LF-A1 [T00467]; 41; 47; 8.920488; GATGGCA; 1.15503; 1.44036;
Sequence; LF-A1 [T00467]; 51; 57; 4.850633; GAAGGCA; 0.57751; 0.63147;
Sequence; LF-A1 [T00467]; 177; 183; 8.139709; CACGGCA; 0.19250; 0.23772;
Sequence; LF-A1 [T00467]; 275; 281; 0.780778; TAGGGCA; 0.57751; 0.80968;
Sequence; LF-A1 [T00467]; 334; 340; 9.701266; GTTGGCA; 1.73254; 2.14464;
Sequence; LF-A1 [T00467]; 406; 412; 1.561557; GTGGGCA; 0.86627; 1.21548;
Sequence; LF-A1 [T00467]; 421; 427; 4.850633; AAAGGCA; 0.57751; 0.63147;
Sequence; LF-A1 [T00467]; 470; 476; 1.561557; TGCCCAC; 0.86627; 1.21548;
Sequence; LF-A1 [T00467]; 504; 510; 1.561557; TGCCCCA; 0.86627; 1.21548;
Sequence; LF-A1 [T00467]; 537; 543; 8.139709; CATGGCA; 0.19250; 0.23772;
Sequence; LF-A1 [T00467]; 633; 639; 0.780778; TGCCCTT; 0.57751; 0.80968;
Sequence; LF-A1 [T00467]; 696; 702; 9.701266; TGCCACA; 1.73254; 2.14464;
Sequence; LF-A1 [T00467]; 930; 936; 0.780778; TGCCCAG; 0.57751; 0.80968;
Sequence; LF-A1 [T00467]; 1040; 1046; 1.561557; TGCCCCC; 0.86627; 1.21548;
Sequence; LF-A1 [T00467]; 1100; 1106; 1.561557; TGCCCAC; 0.86627; 1.21548;
Sequence; LF-A1 [T00467]; 1418; 1424; 9.701266; TGCCAAA; 1.73254; 2.14464;
Sequence; POU2F2C [T00665]; 489; 496; 11.705043; ATGCAGCT; 0.64970; 0.50927;
Sequence; POU2F2C [T00665]; 1417; 1424; 13.840971; ATGCCAAA; 1.08284; 0.70978;
Sequence; AP-4 [T00036]; 45; 55; 4.297334; GCAGCTGAAGG; 0.00752; 0.01020;
Sequence; AP-4 [T00036]; 488; 498; 14.985272; GATGCAGCTGG; 0.11844; 0.17123;
Sequence; Myf-5 [T00521]; 487; 499; 14.245790; TGATGCAGCTGGG; 0.09461; 0.12866;
Sequence; Sp3 [T02338]; 6; 14; 11.119741; GGGGCACCG; 0.18649; 0.32402;
Sequence; Sp3 [T02338]; 137; 145; 11.609185; GGGGCGTCA; 0.10227; 0.16877;
Sequence; Sp3 [T02338]; 501; 509; 6.880245; CCTTGCCCC; 0.12032; 0.21610;
Sequence; Sp3 [T02338]; 672; 680; 6.634728; CCTGGCCCC; 0.08422; 0.16757;
Sequence; Sp3 [T02338]; 742; 750; 10.420253; GGGGCTTTC; 0.09625; 0.16188;
Sequence; Sp3 [T02338]; 766; 774; 6.634728; CGCAGCCCC; 0.08422; 0.16757;
Sequence; Sp3 [T02338]; 794; 802; 1.010444; TCCCGCCCC; 0.04211; 0.11241;
Sequence; Sp3 [T02338]; 837; 845; 1.010444; ACCCGCCCC; 0.04211; 0.11241;
Sequence; Sp3 [T02338]; 993; 1001; 6.938589; GGGGCTTGT; 0.12032; 0.21610;
Sequence; Sp3 [T02338]; 1025; 1033; 8.731604; GGGGCGCTC; 0.01203; 0.02882;

Sequence; Sp3 [T02338]; 1037; 1045; 2.238026; TCCTGCCCC; 0.03609; 0.07382;
Sequence; Sp3 [T02338]; 1066; 1074; 9.461060; CAGGGCCCC; 0.05414; 0.10801;
Sequence; TFIIB [T00818]; 149; 156; 10.522345; GCTGAGGA; 0.62564; 0.78973;
Sequence; TFIIB [T00818]; 195; 202; 3.507448; CCTGAGAC; 0.14438; 0.15191;
Sequence; TFIIB [T00818]; 494; 501; 14.029794; GCTGGGAC; 0.86627; 1.08101;
Sequence; TFIIB [T00818]; 519; 526; 10.522345; TCCTCAGT; 0.62564; 0.78973;
Sequence; TFIIB [T00818]; 580; 587; 14.029794; CCTGCGTC; 0.86627; 1.08101;
Sequence; TFIIB [T00818]; 587; 594; 4.642258; CTCTCAGG; 0.14438; 0.15414;
Sequence; TFIIB [T00818]; 682; 689; 4.642258; CTCGCAGA; 0.14438; 0.15414;
Sequence; TFIIB [T00818]; 764; 771; 11.657154; ATCGCAGC; 0.62564; 0.80952;
Sequence; TFIIB [T00818]; 786; 793; 4.642258; ATCTCAGG; 0.14438; 0.15414;
Sequence; TFIIB [T00818]; 855; 862; 3.507448; TTCTCAGG; 0.14438; 0.15191;
Sequence; TFIIB [T00818]; 1003; 1010; 7.014897; CCTGCGAC; 0.43314; 0.46692;
Sequence; TFIIB [T00818]; 1029; 1036; 11.657154; CGCTCAGG; 0.62564; 0.80952;
Sequence; TFIIB [T00818]; 1062; 1069; 10.522345; TTCCCAGG; 0.62564; 0.78973;
Sequence; TFIIB [T00818]; 1122; 1129; 11.657154; ACCTCAGC; 0.62564; 0.80952;
Sequence; TFIIB [T00818]; 1214; 1221; 11.657154; ATCGCAGC; 0.62564; 0.80952;
Sequence; TFIIB [T00818]; 1235; 1242; 11.657154; GCTGAGGG; 0.62564; 0.80952;
Sequence; TFIIB [T00818]; 1310; 1317; 14.029794; TACCCAGA; 0.86627; 1.08101;
Sequence; TFIIB [T00818]; 1552; 1559; 11.657154; CCTGGGAG; 0.62564; 0.80952;
Sequence; AREB6 [T00625]; 59; 66; 6.503920; CAGGTTTA; 0.21657; 0.20859;
Sequence; AREB6 [T00625]; 411; 418; 6.389933; CAGGTTAG; 0.21657; 0.20859;
Sequence; AREB6 [T00625]; 527; 534; 0.664330; CCCACCTG; 0.07219; 0.09714;
Sequence; AREB6 [T00625]; 706; 713; 6.945873; CCAACCTG; 0.16844; 0.20528;
Sequence; AREB6 [T00625]; 790; 797; 7.978927; CAGGTCCC; 0.21657; 0.24110;
Sequence; AREB6 [T00625]; 859; 866; 6.678256; CAGGTCCA; 0.21657; 0.20859;
Sequence; AREB6 [T00625]; 937; 944; 6.392732; CAGGTTCA; 0.21657; 0.20859;
Sequence; AREB6 [T00625]; 999; 1006; 4.394059; TGTACCTG; 0.14438; 0.12808;
Sequence; AREB6 [T00625]; 1033; 1040; 7.870538; CAGGTCCT; 0.21657; 0.24110;
Sequence; AREB6 [T00625]; 1373; 1380; 7.585013; AGAACCTG; 0.12032; 0.10711;
Sequence; Ik-1 [T02702]; 488; 500; 11.871492; GATGCAGCTGGGA; 0.05083; 0.06568;
Sequence; Ik-1 [T02702]; 526; 538; 14.245790; TCCCACCTGCTCA; 0.09461; 0.11658;
Sequence; Ik-1 [T02702]; 1063; 1075; 10.408185; TCCCAGGGCCCCG; 0.00773; 0.01202;
Sequence; E12 [T00204]; 54; 66; 13.547281; GGCAGCAGGTTTA; 0.00790; 0.01111;
Sequence; E12 [T00204]; 527; 539; 7.332052; CCCACCTGCTCAT; 0.00522; 0.00742;
Sequence; POU6F1 [T04470]; 542; 552; 14.123853; CATACTCTAGA; 0.06053; 0.03048;
Sequence; POU6F1 [T04470]; 1156; 1166; 14.123853; CATATTGTTAG; 0.06053; 0.03048;
Sequence; Max [T05056]; 552; 562; 14.285384; ATACGTGGTTT; 0.22108; 0.21107;
Sequence; AIRE [T05990]; 556; 565; 10.667168; GTGGTTTCTA; 0.13535; 0.13097;
Sequence; Nrf2 [T01443]; 618; 628; 13.939071; ACATGCTTAAG; 0.10152; 0.06936;
Sequence; Nrf2 [T01443]; 898; 908; 13.939071; CTTAAGCATGA; 0.10152; 0.06936;
Sequence; Nrf2 [T01443]; 915; 925; 11.111955; ACATGCTGAAG; 0.06317; 0.04625;
Sequence; NF-AT4 [T01946]; 633; 644; 13.468561; TGCCCTTTTTCC; 0.02106; 0.01782;
Sequence; NF-AT4 [T01946]; 1507; 1518; 13.468561; GCGGAATTTTCC; 0.02106; 0.01782;
Sequence; Pu box binding factor [T00704]; 635; 644; 11.593735; CCCTTTTTCC; 0.24364; 0.18280;
Sequence; NF-AT3 [T02462]; 635; 644; 6.481482; CCCTTTTTCC; 0.04813; 0.03958;
Sequence; NF-AT3 [T02462]; 1359; 1368; 6.481482; GGAAAATGGG; 0.04813; 0.03958;
Sequence; NF-AT3 [T02462]; 1368; 1377; 6.481482; GGAAAAGAAC; 0.04813; 0.03958;
Sequence; NF-AT3 [T02462]; 1509; 1518; 5.555555; GGAATTTTCC; 0.07219; 0.05145;
Sequence; AML1 [T01067]; 638; 650; 11.351203; TTTTTCCACAACT; 0.03405; 0.02838;
Sequence; c-Myb [T00137]; 330; 337; 10.189227; GAGAGTTG; 0.28876; 0.21064;
Sequence; c-Myb [T00137]; 646; 653; 11.694333; CAACTCCT; 0.33688; 0.25025;
Sequence; c-Myb [T00137]; 656; 663; 6.539977; CCAAGTTG; 0.24063; 0.22108;

Sequence; c-Myb [T00137]; 865; 872; 11.694333; CAACTACA; 0.33688; 0.25025;
Sequence; BTEB3 [T05051]; 648; 656; 6.668514; ACTCCTCTC; 0.07219; 0.08767;
Sequence; BTEB3 [T05051]; 1302; 1310; 0.000000; ACTCCCCTT; 0.01203; 0.01468;
Sequence; BTEB3 [T05051]; 1543; 1551; 12.178270; GGAAGGAGT; 0.27673; 0.32454;
Sequence; NF-Y [T00150]; 687; 694; 1.003765; AGACCAAT; 0.16844; 0.14816;
Sequence; PPAR-alpha:RXR-alpha [T05221]; 493; 503; 10.172413; AGCTGGGACCT; 0.06768; 0.12948;
Sequence; PPAR-alpha:RXR-alpha [T05221]; 738; 748; 4.512070; GACTGGGGCTT; 0.02557; 0.03563;
Sequence; PPAR-alpha:RXR-alpha [T05221]; 751; 761; 10.172413; TCGGCCCAGCG; 0.06768; 0.12948;
Sequence; T3R-alpha1 [T01152]; 786; 796; 13.154760; ATCTCAGGTCC; 0.05414; 0.05456;
Sequence; T3R-alpha1 [T01152]; 855; 865; 13.333236; TTCTCAGGTCC; 0.14438; 0.11968;
Sequence; T3R-alpha1 [T01152]; 1029; 1039; 13.969556; CGCTCAGGTCC; 0.26169; 0.24354;
Sequence; DP-1 [T01548]; 791; 800; 11.593735; AGGTCCCGCC; 0.24364; 0.45222;
Sequence; DP-1 [T01548]; 818; 827; 14.492168; ACACCCCGCT; 0.16243; 0.34573;
Sequence; DP-1 [T01548]; 989; 998; 14.492168; GGCGGGGGCT; 0.16243; 0.34573;
Sequence; DP-1 [T01548]; 1383; 1392; 14.492168; AGCGGGCTTC; 0.16243; 0.34573;
Sequence; E2F-1 [T01542]; 794; 800; 0.000000; TCCCGCC; 0.19250; 0.34629;
Sequence; E2F-1 [T01542]; 821; 827; 5.126038; CCCCGCT; 0.19250; 0.36006;
Sequence; E2F-1 [T01542]; 837; 843; 5.944817; ACCCGCC; 0.38501; 0.81629;
Sequence; E2F-1 [T01542]; 888; 894; 3.397038; CCCCGCC; 0.57751; 0.93568;
Sequence; E2F-1 [T01542]; 909; 915; 9.608274; GGCCGCA; 0.77002; 1.08175;
Sequence; E2F-1 [T01542]; 989; 995; 3.397038; GGCGGGG; 0.57751; 0.93568;
Sequence; E2F-1 [T01542]; 1201; 1207; 3.663457; TTCCGCT; 0.57751; 0.93568;
Sequence; E2F-1 [T01542]; 1241; 1247; 7.879274; GGCGGAT; 0.96252; 1.68691;
Sequence; E2F-1 [T01542]; 1383; 1389; 7.673817; AGCGGGC; 0.38501; 0.62686;
Sequence; E2F-1 [T01542]; 1506; 1512; 1.934457; CGCGGAA; 0.38501; 0.60788;
Sequence; E2F-1:DP-1 [T05204]; 793; 801; 7.158536; GTCCCGCCC; 0.06016; 0.09820;
Sequence; E2F-1:DP-1 [T05204]; 836; 844; 9.856911; AACCCGCCC; 0.13836; 0.24543;
Sequence; E2F-1:DP-1 [T05204]; 887; 895; 10.250037; ACCCCGCCC; 0.15039; 0.26194;
Sequence; E2F-1:DP-1 [T05204]; 988; 996; 12.737425; CGGCGGGGG; 0.22860; 0.42430;
Sequence; E2F-1:DP-1 [T05204]; 1191; 1199; 8.217022; ATCACGCCT; 0.09024; 0.14811;
Sequence; E2F-1:DP-1 [T05204]; 1503; 1511; 9.297269; AGGCGCGGA; 0.09024; 0.15462;
Sequence; GCF [T00320]; 792; 802; 12.635787; GGTCCCGCCCC; 0.08798; 0.21406;
Sequence; GCF [T00320]; 886; 896; 13.898109; AACCCCGCCCA; 0.10490; 0.22898;
Sequence; MAZ [T00490]; 795; 805; 14.933281; CCCGCCCCATC; 0.06317; 0.11772;
Sequence; WT1 [T00899]; 840; 850; 11.537845; CGCCCCCGGGC; 0.06317; 0.18408;
Sequence; WT1 [T00899]; 988; 998; 11.883285; CGGCGGGGGCT; 0.08121; 0.19462;
Sequence; Egr-3 [T00243]; 398; 410; 9.093379; GTAAGTGGGTGGG; 0.01480; 0.02018;
Sequence; Egr-3 [T00243]; 889; 901; 13.640068; CCCGCCCACCTTA; 0.03299; 0.05288;
Sequence; Egr-3 [T00243]; 1079; 1091; 11.366723; CACACCCACAACC; 0.02270; 0.03493;
Sequence; Pbx1 [T06000]; 902; 913; 4.655113; AGCATGAGGCCG; 0.01288; 0.01007;
Sequence; GCMa [T02306]; 916; 924; 12.887507; CATGCTGAA; 0.20454; 0.20859;
Sequence; GCMa [T02306]; 1268; 1276; 14.599354; CCTCGCATT; 0.14438; 0.11867;
Sequence; PXR-1:RXR-alpha [T05671]; 935; 945; 14.688688; AGCAGGTTCAT; 0.01354; 0.00888;
Sequence; Sp1 [T00759]; 795; 803; 0.166549; CCCGCCCCA; 0.02406; 0.06949;
Sequence; Sp1 [T00759]; 838; 846; 0.000000; CCCGCCCCC; 0.02406; 0.06949;
Sequence; Sp1 [T00759]; 889; 897; 0.956058; CCCGCCCAC; 0.07219; 0.16994;
Sequence; Sp1 [T00759]; 986; 994; 9.525937; CTCGGCGGG; 0.16243; 0.32265;
Sequence; Sp1 [T00759]; 1238; 1246; 3.173774; GAGGGCGGA; 0.01203; 0.02176;
Sequence; WT1 I -KTS [T00900]; 839; 848; 10.053856; CCGCCCCCGG; 0.02406; 0.06684;
Sequence; WT1 I -KTS [T00900]; 990; 999; 1.833224; GCGGGGGCTT; 0.01203; 0.03300;
Sequence; WT1 I -KTS [T00900]; 1039; 1048; 12.803692; CTGCCCCCGG; 0.06016; 0.12270;
Sequence; WT1 I -KTS [T00900]; 1099; 1108; 14.759972; GTGCCCACGA; 0.05715; 0.10278;
Sequence; WT1 I -KTS [T00900]; 1264; 1273; 6.133634; AGGCCCTCGC; 0.01805; 0.03507;

Sequence; WT1 -KTS [T01839]; 839; 848; 10.053856; CCGCCCCCGG; 0.02406; 0.06684;
Sequence; WT1 -KTS [T01839]; 990; 999; 1.833224; GCGGGGGCTT; 0.01203; 0.03300;
Sequence; WT1 -KTS [T01839]; 1039; 1048; 12.803692; CTGCCCCCGG; 0.06016; 0.12270;
Sequence; WT1 -KTS [T01839]; 1099; 1108; 14.759972; GTGCCCACGA; 0.05715; 0.10278;
Sequence; WT1 -KTS [T01839]; 1264; 1273; 6.133634; AGGCCCTCGC; 0.01805; 0.03507;
Sequence; ER-beta [T04651]; 110; 118; 9.734206; TGACCCTTC; 0.04813; 0.05515;
Sequence; ER-beta [T04651]; 1016; 1024; 9.807616; CGAGGGTCA; 0.09625; 0.11403;
Sequence; ER-beta [T04651]; 1453; 1461; 6.884336; AGTAGGTCA; 0.09625; 0.08916;
Sequence; RAR-beta [T00721]; 110; 117; 3.404424; TGACCCTT; 0.16844; 0.18322;
Sequence; RAR-beta [T00721]; 1017; 1024; 3.501478; GAGGGTCA; 0.16844; 0.18322;
Sequence; RAR-beta [T00721]; 1454; 1461; 1.040300; GTAGGTCA; 0.12032; 0.09945;
Sequence; ER-alpha [T00261]; 109; 116; 1.577879; GTGACCCT; 0.31282; 0.32059;
Sequence; ER-alpha [T00261]; 1018; 1025; 2.671857; AGGGTCAG; 0.14438; 0.18561;
Sequence; ER-alpha [T00261]; 1455; 1462; 1.409838; TAGGTCAG; 0.31282; 0.32059;
Sequence; RelA [T00594]; 29; 38; 9.221808; AGGGAAGTCC; 0.04963; 0.05827;
Sequence; RelA [T00594]; 1058; 1067; 10.167859; GCGCTTCCCA; 0.04211; 0.04849;
Sequence; RelA [T00594]; 1357; 1366; 13.809119; AGGGAAAATG; 0.06918; 0.07018;
Sequence; RelA [T00594]; 1366; 1375; 14.742243; GGGGAAAAGA; 0.09625; 0.09985;
Sequence; RelA [T00594]; 1563; 1572; 7.720419; GGCTTTCCCT; 0.03008; 0.03370;
Sequence; EBF [T05427]; 1062; 1072; 4.120419; TTCCCAGGGCC; 0.01955; 0.03684;
Sequence; RAR-beta:RXR-alpha [T05420]; 1062; 1073; 12.463326; TTCCCAGGGCCC; 0.10321; 0.15724;
Sequence; USF1 [T00874]; 1094; 1102; 8.900438; AACCCGTGC; 0.22860; 0.33452;
Sequence; Pax-5 [T00070]; 140; 146; 4.979591; GCGTCAT; 0.67377; 0.80061;
Sequence; Pax-5 [T00070]; 583; 589; 2.739140; GCGTCTC; 0.38501; 0.46645;
Sequence; Pax-5 [T00070]; 759; 765; 4.979591; GCGTCAT; 0.67377; 0.80061;
Sequence; Pax-5 [T00070]; 954; 960; 11.542216; GCGTCGG; 0.38501; 0.54391;
Sequence; Pax-5 [T00070]; 1105; 1111; 7.061313; ACGACGC; 0.19250; 0.24039;
Sequence; Pax-5 [T00070]; 1191; 1197; 2.240452; ATCACGC; 0.28876; 0.39218;
Sequence; Pax-5 [T00070]; 1227; 1233; 7.762216; GCGTGCA; 0.19250; 0.30864;
Sequence; Pax-5 [T00070]; 1490; 1496; 8.803077; GCGTGGG; 0.19250; 0.31999;
Sequence; ERRalpha1 [T05682]; 1118; 1130; 13.621444; CCTCACCTCAGCA; 0.04695; 0.03878;
Sequence; HNF-4alpha [T03828]; 1126; 1138; 13.587440; CAGCACTTTGTTC; 0.02390; 0.01737;
Sequence; CUTL1 [T00100]; 429; 434; 0.925283; AACAAT; 1.92505; 1.51831;
Sequence; CUTL1 [T00100]; 689; 694; 0.000000; ACCAAT; 0.38501; 0.29066;
Sequence; CUTL1 [T00100]; 806; 811; 0.967341; AGCAAT; 1.92505; 1.51831;
Sequence; CUTL1 [T00100]; 1159; 1164; 0.925283; ATTGTT; 1.92505; 1.51831;
Sequence; CUTL1 [T00100]; 1390; 1395; 1.732064; TTCAAT; 1.15503; 0.75858;
Sequence; CUTL1 [T00100]; 1467; 1472; 0.925283; AACAAT; 1.92505; 1.51831;
Sequence; AhR:Arnt [T05394]; 1193; 1201; 9.185597; CACGCCTCT; 0.05414; 0.07277;
Sequence; AhR:Arnt [T05394]; 1223; 1231; 5.810987; TCCGGCGTG; 0.30079; 0.41298;
Sequence; AhR:Arnt [T05394]; 1486; 1494; 5.888384; CCAAGCGTG; 0.30079; 0.41298;
Sequence; c-Ets-2 [T00113]; 515; 522; 2.827022; GAATTCCT; 0.28876; 0.23872;
Sequence; c-Ets-2 [T00113]; 1210; 1217; 2.827022; AGGAATCG; 0.28876; 0.23872;
Sequence; c-Ets-2 [T00113]; 1278; 1285; 2.827022; CCGTTCCT; 0.28876; 0.23872;
Sequence; c-Ets-2 [T00113]; 1344; 1351; 4.629332; GTCTTCCT; 0.43314; 0.36366;
Sequence; c-Ets-2 [T00113]; 1449; 1456; 2.485451; AGGAAGTA; 0.14438; 0.13402;
Sequence; c-Ets-2 [T00113]; 1461; 1468; 4.629332; AGGAAGAA; 0.43314; 0.36366;
Sequence; c-Ets-2 [T00113]; 1542; 1549; 4.629332; AGGAAGGA; 0.43314; 0.36366;
Sequence; Arnt [T01346]; 1193; 1201; 10.241323; CACGCCTCT; 0.10227; 0.16708;
Sequence; Arnt [T01346]; 1223; 1231; 11.217784; TCCGGCGTG; 0.12032; 0.17704;
Sequence; Arnt [T01346]; 1486; 1494; 11.057524; CCAAGCGTG; 0.12032; 0.17704;
Sequence; AhR [T01795]; 1190; 1197; 1.711124; GATCACGC; 0.21657; 0.33487;

Sequence; AhR [T01795]; 1227; 1234; 2.493742; GCGTGCAA; 0.36095; 0.50033;
Sequence; AhR [T01795]; 1490; 1497; 1.766689; GCGTGGGT; 0.21657; 0.27714;
Sequence; MIF-1 [T01047]; 1249; 1261; 14.500958; TTAGAGTAACACC; 0.00940; 0.00714;
Sequence; SXR:RXR-alpha [T05670]; 33; 41; 7.175614; AAGTCCAGG; 0.24063; 0.18748;
Sequence; SXR:RXR-alpha [T05670]; 1297; 1305; 11.263007; CTTGGACTC; 0.09625; 0.08467;
Sequence; MZF-1 [T00529]; 240; 246; 0.056332; TCCCCCC; 0.38501; 0.62969;
Sequence; MZF-1 [T00529]; 438; 444; 2.220412; TCCCCGA; 0.38501; 0.48625;
Sequence; MZF-1 [T00529]; 703; 709; 2.107748; TCCCCAA; 0.38501; 0.48625;
Sequence; MZF-1 [T00529]; 1304; 1310; 1.166538; TCCCCTT; 0.38501; 0.46746;
Sequence; MZF-1 [T00529]; 1364; 1370; 1.053874; ATGGGGA; 0.38501; 0.46746;
Sequence; GABP-alpha [T01390]; 1197; 1208; 8.833550; CCTCTTCCGCTG; 0.00865; 0.00930;
Sequence; GABP-alpha [T01390]; 1343; 1354; 14.677905; AGTCTTCCTTTC; 0.02688; 0.02770;
Sequence; GABP-alpha [T01390]; 1432; 1443; 13.457706; ACCCGGAAGTGA; 0.01560; 0.01415;
Sequence; GABP-alpha [T01390]; 1446; 1457; 8.177756; AACAGGAAGTAG; 0.00790; 0.00790;
Sequence; GABP [T00268]; 378; 389; 14.847057; ACCACTTCAGCT; 0.03948; 0.03845;
Sequence; GABP [T00268]; 1196; 1207; 2.995493; GCCTCTTCCGCT; 0.00376; 0.00423;
Sequence; GABP [T00268]; 1271; 1282; 13.611252; CGCATTTCCGTT; 0.01579; 0.01518;
Sequence; GABP [T00268]; 1433; 1444; 3.041476; CCCGGAAGTGAT; 0.00376; 0.00423;
Sequence; CDX2 [T03246]; 1436; 1447; 14.892122; GGAAGTGATAAA; 0.05978; 0.03679;
Sequence; IRF-2 [T01491]; 258; 268; 6.873928; AAGAGTCACTT; 0.15227; 0.10956;
Sequence; IRF-2 [T01491]; 1438; 1448; 6.873928; AAGTGATAAAC; 0.15227; 0.10956;
Sequence; FOXJ2 (long isoform) [T04169]; 1440; 1449; 3.916738; GTGATAAACA; 0.03309; 0.01753;
Sequence; HNF-3beta [T02513]; 60; 66; 6.782764; AGGTTTA; 0.19250; 0.10620;
Sequence; HNF-3beta [T02513]; 1245; 1251; 6.557271; GATTTTA; 0.38501; 0.25181;
Sequence; HNF-3beta [T02513]; 1324; 1330; 2.442513; AGATTTA; 0.19250; 0.08011;
Sequence; HNF-3beta [T02513]; 1444; 1450; 4.950880; TAAACAG; 0.38501; 0.25226;
Sequence; HNF-3beta [T02513]; 1527; 1533; 3.053142; CGATTTA; 0.77002; 0.37300;
Sequence; NERF-1a [T05021]; 1448; 1459; 0.000000; CAGGAAGTAGGT; 0.00019; 0.00019;
Sequence; NERF-1a [T05021]; 1460; 1471; 7.088804; CAGGAAGAACAA; 0.00545; 0.00600;
Sequence; RORalpha2 [T01528]; 1450; 1461; 2.181595; GGAAGTAGGTCA; 0.00273; 0.00159;
Sequence; RORalpha1 [T01527]; 1452; 1461; 2.249512; AAGTAGGTCA; 0.04963; 0.04193;
Sequence; COUP-TF1 [T00149]; 1453; 1461; 1.930067; AGTAGGTCA; 0.03008; 0.02415;
Sequence; SF-1 [T02769]; 1452; 1462; 12.020287; AAGTAGGTCAG; 0.00602; 0.00615;
Sequence; AR [T00040]; 268; 275; 0.671565; TGTTCTGT; 0.19250; 0.14875;
Sequence; AR [T00040]; 1134; 1141; 5.899530; TGTTCGGC; 0.36095; 0.36043;
Sequence; AR [T00040]; 1463; 1470; 0.729340; GAAGAACA; 0.19250; 0.14875;
Sequence; HOXD9 [T01424]; 1470; 1479; 11.174228; AATAATAAGG; 0.14739; 0.08139;
Sequence; HOXD10 [T01425]; 1470; 1479; 11.174228; AATAATAAGG; 0.14739; 0.08139;
Sequence; AP-3 (2) [T00039]; 61; 67; 3.763516; GGTTTAG; 0.57751; 0.46789;
Sequence; AP-3 (2) [T00039]; 412; 418; 11.290546; AGGTTAG; 1.15503; 0.86689;
Sequence; AP-3 (2) [T00039]; 1325; 1331; 0.000000; GATTTAG; 0.28876; 0.19890;
Sequence; AP-3 (2) [T00039]; 1500; 1506; 8.971049; CTAAGGC; 0.48126; 0.40102;
Sequence; NF-kappaB [T00590]; 1508; 1518; 7.571839; CGGAATTTTCC; 0.00602; 0.00476;
Sequence; E2F-5 [T01607]; 1505; 1512; 13.735780; GCGCGGAA; 0.50533; 0.60821;
Sequence; HNF-1B [T01950]; 1472; 1479; 11.219398; TAATAAGG; 0.31282; 0.16536;
Sequence; RAR-alpha1 [T00719]; 1453; 1462; 7.753480; AGTAGGTCAG; 0.01203; 0.01443;
Sequence; RXR-alpha [T01345]; 1453; 1461; 2.099821; AGTAGGTCA; 0.15039; 0.14063;
Sequence; ELF-1 [T01113]; 1445; 1457; 5.072491; AAACAGGAAGTAG; 0.00113; 0.00076;
Sequence; Pbx1b [T02087]; 345; 354; 14.774452; TGATGAAGGA; 0.16243; 0.11646;
Sequence; Pbx1b [T02087]; 1285; 1294; 12.747416; TCATGACAGA; 0.11731; 0.10185;
Sequence; PU.1 [T02068]; 514; 523; 3.227625; GGAATTCCTC; 0.02256; 0.01920;
Sequence; PU.1 [T02068]; 1277; 1286; 3.941435; TCCGTTCCTC; 0.03158; 0.03628;
Sequence; R1 [T00711]; 1235; 1246; 9.864560; GCTGAGGGCGGA; 0.02331; 0.03759;

Sequence; TFII-I [T00824]; 86; 96; 6.742511; AGTGGATAGAC; 0.02406; 0.02392;
Sequence; TFII-I [T00824]; 220; 230; 9.416256; CTAGGATGGCC; 0.06317; 0.06314;
Sequence; TFII-I [T00824]; 1045; 1055; 6.759160; CCGGGATAGAC; 0.00602; 0.00674;
Sequence; T3R-beta1 [T00851]; 789; 799; 10.019726; TCAGGTCCCGC; 0.01542; 0.01712;
Sequence; T3R-beta1 [T00851]; 858; 868; 10.753334; TCAGGTCCAAC; 0.01955; 0.02161;
Sequence; T3R-beta1 [T00851]; 1032; 1042; 9.756265; TCAGGTCCTGC; 0.01542; 0.01712;
Sequence; CRF [T00170]; 690; 701; 12.356035; CCAATCTGCCAC; 0.05076; 0.06360;
Sequence; RFX1 [T01673]; 660; 668; 9.693213; GTTGCTTCA; 0.04813; 0.04405;
Sequence; POU2F1 [T00641]; 303; 312; 13.518184; TCATTTGCTT; 0.50533; 0.30063;
Sequence; POU2F2 [T00647]; 302; 312; 13.200917; ATCATTTGCTT; 0.31583; 0.20658;
Sequence; octamer-binding factor [T01225]; 302; 312; 14.796379; ATCATTTGCTT; 0.28425; 0.18062;

-- END ---

Distribution of the nucleotides over the given chain:

A	24.0%
C	30.4%
G	26.2%
T	19.5%
